# A multivariate Bayesian modeling strategy coupled with QTL analysis reveals genetic loci linking important sensory wine quality attributes with their corresponding wine aroma compounds

**DOI:** 10.3389/fpls.2026.1851889

**Published:** 2026-06-26

**Authors:** Tom Heinekamp, Armin Schüttler, Franco Röckel, Annemarie Siebert, Jörg Gottmann, Jochen Vestner, Lena Frenzke, Torsten Wenke, Stefan Wanke, Ulrich Fischer, Reinhard Töpfer, Florian Schwander

**Affiliations:** 1 Julius Kuehn-Institut, Federal Research Centre for Cultivated Plants, Institute for Grapevine Breeding Geilweilerhof, Siebeldingen, Germany; 2 Dienstleistungszentrum Ländlicher Raum - Rheinpfalz, Institute for Viticulture and Enology, Neustadt, Germany; 3 Technische Universität Dresden, Fakultät Biologie, Professur für Botanik, Dresden, Germany; 4 Goethe-Universität Frankfurt, Institut für Ökologie, Evolution und Biodiversität, Frankfurt am Main, Germany; 5 Senckenberg Forschungsinstitut und Naturmuseum, Frankfurt am Main, Germany; 6 Instituto de Biología, Universidad Nacional Autónoma de México, Mexico City, Mexico

**Keywords:** grapevine breeding, metabolomics, monoterpenes, multivariate genotype by environment modeling, QTL analysis, sensory evaluation, *Vitis vinifera* L., wine quality prediction

## Abstract

Wine quality assessment is the most time-consuming and labor-intensive task in grapevine breeding. Breeding programs worldwide would benefit by accelerating this process, regardless of their primary objectives, such as climate adaptation or disease resistance, because wine quality ultimately determines consumer acceptance. In this study, an F1 mapping population derived from ‘Calardis Musqué’ × ‘Villard Blanc’, segregating for perceived wine quality, was comprehensively characterized for key aroma-relevant metabolites and sensory attributes. A total of 25,284 sensory evaluations were conducted on 1,635 micro-vinified wine samples, which were obtained from 147 genotypes grown at two locations over six vintages. A multivariate Bayesian multilevel modeling approach was applied to disentangle the effects of genotype, environment, and individual taster subjectivity on eight sensory attributes and the total quality score (TQS). Quantitative trait locus (QTL) analysis based on a dense genetic map was performed using the modeled sensory phenotypes as well as quantification data of volatile monoterpenes acquired with a targeted SIDA–SPE–GC–MS. This integrative approach identified overlapping genetic loci linking aroma-active metabolites with their sensory quality attributes. Two major pleiotropic QTL clusters associated with wine quality were identified. The first, located on chromosome 2, simultaneously controlled all major quantified monoterpenes (e.g., linalool: LOD 10.1, PVE 19.7%) and is co-localized with QTLs for the most discriminating sensory attributes, including “floral” (LOD 14.8, PVE 14.3%), “fruity” (LOD 14.4, PVE 17.2%), and “tropical fruit” (LOD 13.1, PVE 16.3%). A second major QTL cluster on chromosome 11 harbored strong QTLs for terpene concentrations as well as quality-related aroma attributes (e.g., floral: LOD 11.1, PVE 10.9%). In addition, QTLs for TQS and the attribute “off-flavor” were identified on several chromosomes, indicating further genomic targets relevant for breeding on wine quality. This integrated workflow combines sensory phenotyping with genotype × environment modeling and estimation of taster bias for subsequent genomic analysis. The approach represents a significant advancement in grapevine quality assessment and provides a framework to accelerate the breeding of new grapevine cultivars that are agronomically valuable, consumer-accepted, and both ecologically and economically sustainable.

## 1 Introduction

### 1.1 Challenges for new cultivars

The German wine sector faces complex challenges. Winemakers are increasingly confronted with the impacts of climate change (Santos et al., 2020), a continuing decline in wine consumption (Mian et al., 2022), and growing economic pressures such as labor shortages and increasing bureaucratic demands, resulting in vineyard abandonment and the closure of wine estates (Vecchio et al., 2022; Eisenmann et al., 2023; The German Wine Institute, 2024). Fungus-resistant grapevine cultivars, commonly referred to as PIWIs, have been developed by breeding programs in countries such as Switzerland, France, Germany, Hungary, and Italy to mitigate the challenges caused by biotic stress. However, these cultivars have not achieved broad market acceptance yet (Töpfer and Trapp, 2022; Di Vita et al., 2024; Piccoli and Viganò, 2025).

New grapevine cultivars are expected to match or exceed the quality of traditional *Vitis vinifera* cultivars in order to gain acceptance among both winegrowers and consumers, who often exhibit strong loyalty toward established varieties (Trapp et al., 2025). Wine quality is determined by a balanced composition of volatile aroma compounds, taste, and mouthfeel and is strongly influenced by environmental conditions during grape development and ripening as well as by enological practices during vinification. Objective phenotyping of this highly complex trait remains one of the most labor-intensive and time-consuming bottlenecks in grapevine breeding, considerably slowing the development of new high-quality cultivars.

Consumer expectations further complicate the quality assessment. Even simple changes in wine color can bias sensory perception to the extent that trained experts may be misled (Morrot et al., 2001). Because sensory attributes arise from a complex metabolic background, the identification of metabolite markers, such as volatile monoterpenes, has become a more tangible and higher-throughput approach than a direct holistic sensory evaluation. Although metabolomic markers are promising tools, they still require grapes and resulting wines for screening, which are only available 3 to 5 years after planting a new grapevine.

The development and application of marker-assisted selection (MAS) has accelerated the selection process for quantitatively inherited traits in modern breeding programs. MAS is a cost-effective approach that uses genetic markers linked to traits of interest at early developmental stages. In biparental populations, quantitative trait locus (QTL) analysis is commonly used to identify genomic regions underlying trait variation, thereby facilitating the discovery of candidate genes and associated molecular markers (Töpfer et al., 2011). During the last two decades, numerous QTLs conferring resistance to major grapevine pathogens have been identified and characterized (as reviewed in Vezzulli et al. (2022)). An updated overview currently lists 32 resistance loci against downy mildew and 13 loci against powdery mildew (www.vivc.de/loci). This knowledge has enabled the targeted pyramiding of resistance loci through MAS, resulting in more durable and enhanced resistances in newly developed cultivars (Eibach et al., 2007). In contrast, reliable MAS markers connected to wine-quality-related traits are rare.

### 1.2 Genetic markers to select for quality traits

Genetic studies have identified QTLs associated with a range of viticultural and oenological traits, including berry weight (Doligez et al., 2002), wine aroma (Duchêne et al., 2009), berry acidity (Houel et al., 2015; Bayo-Canha et al., 2019), and the onset of berry ripening (veraison), which was previously mapped in the same F1 population used in the present study (Zyprian et al., 2015; Frenzke et al., 2024). Nevertheless, elucidating the genetic basis of complex sensory wine quality traits, which depend on numerous odor and taste properties, remains a major challenge.

A central task is the identification of genes encoding enzymes involved in the biosynthesis of volatile aroma compounds or their precursors. This is complicated by the fact that many grape-derived aroma compounds occur in berries as odorless precursors, including glycosides and cysteine conjugates, which must be enzymatically or chemically released during vinification to become sensory active (Ruiz et al., 2019).

The importance of such compounds is exemplified by monoterpenes, whose concentrations contribute substantially to the classification of grape cultivars into muscat, non-muscat aromatic, and neutral types (Rapp, 1998; Gómez-Plaza et al., 2007). Representative monoterpenes such as geraniol, linalool, and *cis*-rose oxide are highly potent aroma compounds with sensory thresholds in the low microgram-per-liter range (Ferreira et al., 2000). Wines rich in these compounds are typically characterized by pronounced floral and fruity aromas (Robinson, 2005). One parental genotype of the F1 population investigated here, ‘Calardis Musqué’, exhibits intense muscat-like and Gewürztraminer-like aroma characteristics (Institut für Rebenzüchtung Geilweilerhof, 2021), suggesting that isoprenoid and monoterpenoid compounds contribute substantially to the segregation observed within the population.

### 1.3 Scope of this study

The objective of this study was to establish an integrated workflow for the processing and exploitation of sensory data obtained from a trained human panel in order to identify genomic loci associated with sensory perception and wine quality. Standardized micro-vinifications were performed using grapes from 147 F1 individuals of the white wine progeny ‘Calardis Musqué’ × ‘Villard Blanc’ cultivated at two locations, which segregates for wine quality attributes and aroma intensity. Across multiple vintages, this resulted in a unique dataset comprising 1,635 authentic experimental wines.

A specifically trained sensory panel conducted a comprehensive descriptive analysis of all wines. The resulting extensive dataset was subsequently analyzed using a Bayesian multivariate genotype × environment modeling approach, allowing a realistic estimation of the effects attributable to genotype, environment (defined as the interaction of location and vintage), and individual tasters. This framework relaxed statistical assumptions, which can be frequently violated in sensory studies, due to human subjectivity and individual bias. The established workflow enables comprehensive processing and exploitation of sensory intensity and wine quality data for subsequent QTL analysis.

Volatile monoterpenes, known to be important factors in wine quality perception, were quantified using SIDA–SPE–GC–MS and were additionally included in the QTL analysis. Therewith, the established workflow enables comprehensive processing and exploitation of sensory intensity and wine quality data for subsequent QTL analysis. The identification of overlapping confidence intervals between sensory QTLs associated with fruity and floral and QTLs for corresponding aroma compounds highlights the genetic regions of relevance for wine quality. The obtained results provide novel insights into the metabolic and genetic basis of sensory wine quality traits and contribute to the identification of genomic regions controlling aroma perception. Ultimately, these findings pave the way for the development of reliable molecular markers for integrating wine quality traits into MAS. This will improve the precision and efficiency of grapevine breeding through the early prediction of wine quality potential in individual genotypes.

## 2 Materials and methods

### 2.1 White wine F1 mapping population

The biparental F1 mapping population of ‘Calardis Musqué’ (VIVC-No. 4549) × ‘Villard Blanc’ (VIVC-No. 13081) (CM×VB) with 147 genotypes was used for the investigation. The experimental vineyards were established in 2010 in two locations: at the JKI Geilweilerhof, Siebeldingen (SB), Germany (49°1254″ N, 8°0248″ E), consisting of two plots, and at the DLR, Neustadt/Weinstrasse (NW) (49°3573″ N 8°1671″ E), consecutively planted with four vines in SB and five at NW, each within one vineyard and grafted on rootstock ‘Selection Oppenheim 4’ (SO4, VIVC 11473). The vines were planted in a vertical shoot positioning (VSP, north–south-oriented) trellis system and a plant density of 5,000 vines per hectare (2 × 1 m spacing). Pruning was conducted on a flat arch with approximately 10–12 buds per shoot. Parental varieties were co-cultivated in the same plots. Foliage pruning was performed 3 weeks after flowering. Defoliation in the cluster zone was carried out later manually on the eastern side of the canopy to achieve better aeration, reducing the risk of mildew and *Botrytis* infections. The evaluation results of six vintages, namely, 2016, 2017, 2018, 2019, 2020, and 2021, harvested at the location in Siebeldingen and four vintages, namely, 2018, 2019, 2020, and 2021, harvested in Neustadt were micro-vinificated and included in this study. Discrepancies in the number of vintages between both locations are a consequence of capacity constraints (2016) in NW and unrecoverable yield losses caused by a late frost event in 2017.

### 2.2 Sample processing and microvinification

Due to the variation of up to 6 weeks within the ripening processes of the F1 individuals, the maturation of the berries was closely monitored with a refractometer or by FTIR spectroscopic (Foss Winescan, Hamburg, Germany) measurements and the evaluation of ripeness indicators. The decision for optimal harvest date was mainly based on must weight (aim for sugar content of 190 g/L), total acidity, seed ripeness, health status, and berry taste. All grapes of each genotype were hand-harvested, and the eight grapevines in SB and five at NW were separately pooled, resulting in a genotype- and location-specific sample. They were subsequently mixed, destemmed, and pressed. The resulting musts were clarified overnight at 5 °C with 1.0 g/L bentonite (Erbslöh, Geisenheim GmbH, Geisenheim, Germany) and treated with 50 mg/L sulfurous acid to inhibit microbial growth. The clarified supernatants were enriched with 0.3 g/L yeast nutrients (Lalvin^®^ Go-Ferm; Lallemand Inc., Ontario, Canada) and 0.2 g/L pure cultured yeasts (Oenoferm^®^ Freddo F3; Erbslöh Geisenheim GmbH, Geisenheim, Germany). All fermentations were carried out at 18 °C to 19 °C in a 4-L scale within 5-L glass containers and an additional treatment of 0.5 g/L diammonium phosphate (Eaton Industries GmbH, Bonn, Germany) after three days to ensure optimal nutrient supply of the yeasts. After complete fermentation, the wines were separated from the lees and supplied with 100 mg/L sulfur dioxide. The individual wines were deacidified to reach 7 g/L total acids by adding 0.67g/L potassium bicarbonate (Erbslöh, Geisenheim, Germany) or calcium carbonate (Erbslöh, Geisenheim) for the reduction of 1 g/L total acids to the wines, depending on the dimension of deacidification necessary. After 3 months of storage, the wines were filtered with Ø22-cm K 200 depth filter sheets (Pall GmbH, Dreieich, Germany), and the free sulfur dioxide concentration was adjusted to 50 mg/L. Technical replicates of wine per genotype × location × vintage specific samples were not systematically produced due to the quantity constraints of grape material. However, exemplary samples have been replicated in order to demonstrate the reproducibility and robustness of the microvinification procedure. Bottled wines were stored at 8 °C until sensory evaluation. The wine samples were aliquoted at the time point of sensory evaluation and stored at -18 °C until gas chromatography analysis.

### 2.3 Sensory evaluation

The sensory analysis of those six vintages (2016–2021) was conducted with 10 to 17 trained judges per session in a sensory lab equipped according to EN ISO 8589:2010. In total, 56 different trained judges assessed the experimental wines across all sessions and years. The number of evaluated wines differed from 24 to 1,542, depending on the availability per judges over the 6 years. Moreover, 19.6% of the dataset consists of complete trials, in which one judge evaluated every wine from 147 genotypes from both locations across all six vintages.

For sensory evaluation, 20 mL of each wine was presented to the judges at room temperature in transparent wine glasses (DIN 15005). The rating was performed on an unstructured line scale from 0 (lowest possible score) to 10 (highest possible score) with decimal numbers to obtain continuous data. A total of 31 wines were evaluated per session in a randomized order. At least one Calardis Musqué wine of the respective vintage was included in each session as anchor wine. Deviating from fully repeated QDA, only 20% of all experimental wines were evaluated in replicates in order to handle the high number of experimental wines per year. FIZZ software (FIZZ network, version 2.51 c 02, Biosystems, Courtenon, France) was used for data acquisition.

The panel was trained and calibrated to the descriptive attributes by aroma standards in terms of aroma detection and intensity assessment (descriptive analysis) (**Supplementary Table 1**). In a next step, the panelists were specifically trained with a set of experimental wines from the F1 population which exhibit extreme attribute intensities to familiarize the judges with the unusually broad range of wine qualities among the set of the unselected grapevine F1 individuals. The sensory evaluation methodology was developed as a combination of quantitative descriptive evaluation of sensory traits and qualitative assessment of wine quality for both olfactory and taste attributes. Quantitative evaluation was performed as modified quantitative descriptive analysis (QDA) after specific training (Lawless and Heymann, 2010) including the aroma descriptors “fruity”, “tropical fruit”, “floral”, “green/green grassy”, and “off-flavor” and taste descriptors “sweet”, “sour”, and “bitter”. For wines presenting an “off-flavor”, the judges were asked to characterize the type of defect using an integrated “check-all-that-apply” (CATA) approach offering the choice of the classic off-flavors “foxy”, “strawberry”, “atypical aging (ATA)”, “ethyl acetate”, “reductive off-flavor”, “oxidation”, “musty”, “earthy”, and “other”. The qualitative evaluation methodology was adapted from Resolution OIV-OENO 671A-2025 Revision of the "OIV standard for internationa wine and spirituous verages of vitivinicultural orgin competitions^1^ including comprehensive evaluation for aroma “genuineness”, “intensity”, “quality” and taste “genuineness”, “intensity”, “persistence”, and “quality” as well as a holistic smell and taste evaluation “overall judgment”. In order to provide one metric, overall qualitative assessment within the population, the “total quality score” (TQS) was calculated as the weighted *w_i_* average ${\underline{x}}_{w}$ of all qualitative median *x_i_* attribute scores per genotype and per vintage. Both values for quality—smell and taste—were weighted by a factor two.


$$\text{TQS}\;=\;{\underline{x}}_{w}=\;\frac{\sum_{i\;=\;8}^{n}\left(w_{i}\;*\;x_{i}\right)}{\sum_{i\;=\;8}^{n}\left(w_{i}\right)}$$

### 2.4 Gas chromatography quantification

Wines of the CM×VB F1 individuals from the years 2019, 2020, and 2021 and for both experimental vineyards were analyzed using gas chromatography (GC) to quantify *cis*−rose oxide, citronellol, geraniol, *cis-*/*trans-*linalool oxide, linalool, nerol, *α*−terpineol, nerol oxide, and *β*−damascenone. Analytical replicates of the wines were analyzed according to the replications of the wines during the respective sensory evaluation undergoing the full analytical sample processing procedure. The substances utilized for the analysis were obtained from Phenomenex, (Aschaffenburg, Germany), Sigma-Aldrich KGaA (Darmstadt, Germany), and Carl Roth GmbH + Co. KG (Karlsruhe, Germany). The deuterated monoterpenes used as internal standard (IS) were synthesized at the DLR Rheinpfalz, according to Kotseridis et al. (1998) for d4−*β*−damascenone and for d5-linalool as described in Ganss et al. (2011) (see **Supplementary Table 2**). A modified solid-phase extraction (SPE) method in combination with a stable isotope dilution assay (SIDA) coupled to gas chromatography–mass spectrometry (GC–MS) was applied using the instrumentation according to Schober et al. (2023). Deviating from that described procedure, conditioning of the SPE cartridge (styrene-divinylbenzene; particle size 100 µm; Strata SDB-L) with 50 mg of sorbent was achieved with 1 mL each of hexane/dichloromethane (2:1, v:v), methanol, and deionized water. A 10 mL wine sample was spiked with 25 μL IS solution containing 40 mg/L n-dodecane, 12 mg/L d4-β-damascenone, 30 mg/L d5-linalool in Ethanol. This corresponds to a final concentration of 100 μg/L n-dodecane, 30 μg/L d4-β-damascenone, and 75 μg/L d5-linalool in the wine sample, which was subsequently loaded onto the cartridge at a flow rate of one to two drops per second. The cartridge was then rinsed with 1% (w/v) sodium hydrogen carbonate, followed by drying of the sorbent under argon flow for 10 to 15 min. Monoterpenes were eluted with 1 mL of hexane/dichloromethane (2:1, v:v), and 11 µL was directly used for large-volume injection. For improved selectivity and sensitivity, mass spectrometric detection was applied in single-ion monitoring mode with two m/z values per analyte and its stable isotope-labeled internal standard, one serving as a quantifier and one as a qualifier (**Supplementary Table 2**). Calibration curves were generated for the quantification of monoterpenes by analyzing standard mixture solutions prepared in 10 mL of synthetic wine (1 g/L 2,3-butandiol, 15 g/L D-glucose, 15 g/L D-fructose, 1.5 g/L D-malic acid, 1.5 g/L L-tartaric acid, 6 g/L glycerine, and 12% (v/v) ethanol, adjusted to pH 3.5 with 1 molar potassium hydroxide solution). Calibration curves were obtained by calculating the peak surface ratios of the respective analytes to those of their IS as a dependency of analyte concentration. The analytes *cis*−rose oxide, citronellol, geraniol, *cis-*/*trans-*linalool oxide, linalool, nerol, α−terpineol, and nerol oxide were quantified against isotopically labeled d5-linalool and β−damascenone against isotopically labeled d4-β-damascenone. The analytical standard for linalool oxide consisted of *cis-*/*trans*-linalool oxide in their respective pyranoside and furanoside forms, resulting in four distinctive isomers. Isomeric ratio was detected upon the respective peak surfaces of TIC chromatograms and subsequently integrated in calibration, whereas final quantification in this study was limited to the furanoid forms.

### 2.5 Statistical analysis

#### 2.5.1 Genetic map

The genetic map was constructed using haplotype-based markers (HBMs) following Frenzke et al. (2024). Compared to pure SNP markers, this marker type provides phase information for up to four alleles. Genotyping-by-sequencing (GBS) libraries were generated by restriction enzyme digestion with *Psi*I, followed by standard Illumina library preparation and paired-end sequencing (150 bp). Raw reads were then quality-trimmed, filtered for the restriction motif, and standardized to 100 bp. Parental reads were *de novo* clustered (90% identity) to define putative loci and alleles, while low-quality and singleton sequences were removed. Clean reads from all genotypes were then mapped to these loci using exact matching to determine allele presence and absence. HBMs with excessive missing data were discarded, and the remaining HBMs were anchored to the *V. vinifera* PN40024 reference genome 12X.v2 (Canaguier et al., 2017) as well as to the T2T PN40024 reference genome (Shi et al., 2023) (v5) for the different downstream genetic analyses. A genetic map was constructed using the *onemap* package (Margarido et al., 2007), with the markers coded, filtered for nonredundancy, and tested for segregation distortion, retaining only markers with near-Mendelian distribution (*χ*²< 65). The markers were grouped by linkage and constrained to single chromosomes, and a representative set with high data completeness and low distortion was selected to achieve even genome coverage. Final mapping to the 19 grapevine chromosomes was performed assisted by a reference-guided order and the Kosambi mapping function. For a more detailed description, refer to Frenzke et al. (2024).

#### 2.5.2 Estimation of environmental and taster influences

To increase the reliability of the QTL analysis results, an estimation of the environmental effects is crucial, especially in this case, in which diverse vintages from a segregating population meet tasters with their individual underlying preferences. Data from the sensory evaluations and GC were dissected thoroughly to draw conclusions for the development of an adapted genotype-by-environment (G×E) model to compensate for the tasters’ effect in sensory evaluations of wine.

All analyses were performed via R statistical software v4.2.2 (R Core Team, 2022) in *RStudio* v2024.4.0.735 (Posit team, 2024). The F1 individuals were classified in three quality groups: “best”, “average”, and “worst”. Those were characterized through the 20th and 80th quantile of the TQS density plot across all years and locations based on the sensory evaluation data. Those groups were used to analyze universal relations in quality perception and chemical compounds. Pearson correlation coefficients with corresponding *p*-values were computed with the R core package *stats*. Group differences were calculated using the *FSA* package (Ogle et al., 2015), performing a Kruskal–Wallis test followed by Dunn’s *post-hoc* test. The plots were designed using *ggplot2* (Wickham, 2016) and *ggdist* (Kay, 2023, 20). Principal component analysis (PCA) was calculated using *FactoMineR* (Lê et al., 2008). The missing days of the veraison from the dataset provided by Frenzke et al. (2024) were interpolated with *mice* (Buuren and Groothuis-Oudshoorn, 2011).

To model the GC data from 2019 to 2021, the *sommer* package (Covarrubias-Pazaran, 2016) was used, setting the day of the veraison and environment as fixed effects. In the sensory analysis, the impact of ripening was found to be very little (<7%) and excluded from the *brms* model for simplification, but it was significant for the terpene measurement from the wine. For *sommer*, the hierarchy within the data matrix of the sensory evaluation data was too extensive to handle; thus, another approach was taken. To analyze the eight sensory attributes and the TQS simultaneously, a Bayesian multivariate multilevel model created with the *brms* package (Bürkner, 2017) was used. It provides a high-level interface for the programming language Stan (Carpenter et al., 2017) and adapts the lme4 syntax for accessibility. While the tasters worked with a slider on a bounded scale from 0 to 10 and were able to choose decimal numbers, the data were treated as continuous for simplicity. Categorical and bounded scales were not yet supported in a multivariate approach.


$$mvbind\left(Y_{traits}\right)∼1+environment+(1|taster)+(1|gr\left(genotype,cov=G\right))+\ldots \ldots (1|genotype:environment),\sigma ∼1+(1|taster)$$

The multivariate response (mvbind) enabled the joint modeling of all sensory attributes (fruity, sour, etc.), to account for residual correlations among descriptors. This acknowledged that perceptual errors are likely correlated—for instance, a high rating in floral positively biases specific attribute ratings like fruity. The years and the locations were concluded to a single environment-variable (e.g., year 2016 in location Siebelding - 2016_SB) to reduce overparameterization. Genotypic effects were included through a kinship matrix calculated using *r/QTL* (Broman et al., 2003). The genotype × environment interaction was modeled as a random effect, capturing the specific combining ability of each genotype–environment pair, that deviated from the additive main effects. To relax the assumption of homoscedasticity for every taster, the formula contained σ, calculating residual standard deviation for each individual. This accommodated differences in panelist reliability and consistency, assuming varying skill levels and perception sensitivities. The dispersion parameter was modeled using a log-link function. This formulation explicitly modeled the precision of each panelist, down-weighting observations from tasters with higher residual variance (lower consistency) without removing data. The day of the year (DOY) for the onset of ripening (veraison) was included as a variable first. As this did not improve the model, the veraison parameter was excluded afterward to reduce the number of parameters.

All parameters were estimated using full Bayesian inference. The default weakly informative priors provided by *brms* were set. These included flat, improper uniform priors for population-level effects, an LKJ(1) prior (a uniform distribution over all valid correlation matrices) for all correlation matrices and half-Student-*t* priors for all standard deviation parameters. The model was fitted using four independent Markov chains, each run for 3,000 iterations with 1,000 warmup iterations, yielding a total of 8,000 posterior samples. To eliminate divergent transitions common in hierarchical models with sparse data, the adapt_delta parameter was increased to 0.90, and the maximum tree depth was set to 12 to ensure model convergence. For the evaluation of every trait, *R*² was calculated and a leave-one-out cross-validation (LOO) was conducted using 3,000 draws in *loo* (Vehtari et al., 2017). For the monoterpenes, the univariate approach using *sommer* with its default parameters was applied:


trait∼1+DOY_veraison+environment+(1|gr(genotype,cov=G))

#### 2.5.3 QTL analysis

Interval mapping (IM) and composite interval mapping (CIM) were performed on the estimated best linear unbiased predictors (BLUPs) for each genotype. This was performed for all attributes of the sensory model, as well as for the GC-data model, using the packages *r/QTL* for IM and *fullsibQTL* for CIM (Gazaffi et al., 2020). Statistical thresholds were determined using 10,000 permutations. Genotype probabilities were calculated at 1-cM intervals with an assumed genotyping error rate of 0.0001. Significance thresholds for the logarithm of the odds (LOD) scores were established through both genome-wide and chromosome-specific permutation tests to control the experiment-wise error rate. Cofactors were selected via multiple linear regression functions from the *fullsibQTL* package. For each significant locus, 1-LOD and 2-LOD drop confidence intervals, as well as 95% and 99% Bayes credible intervals, were calculated for downstream analysis. The proportion of phenotypic variance explained by each detected QTL was quantified.

## 3 Results

### 3.1 Sensory evaluation of wines from the ‘Calardis Musqué’ × ‘Villard Blanc’ population

#### 3.1.1 Classification into sensory wine quality groups

**Figure 1:**
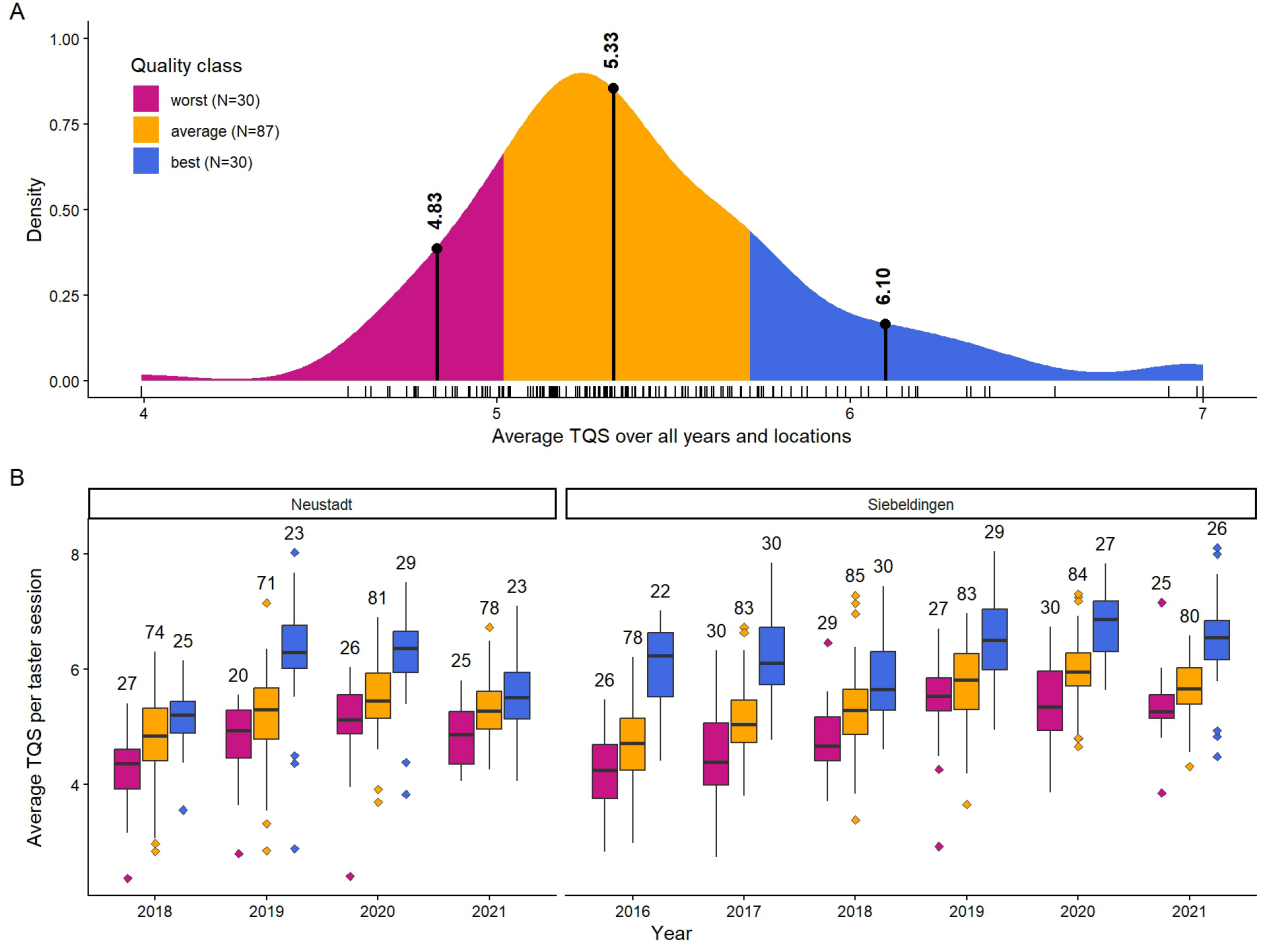
**(A)** Segregation of wine quality represented by the average total quality score (TQS) of each genotype within the population CM×VB, colored by the 20th and 80th quantile and the average value for the formed quality groups. **(B)** Quality group performance separated by the vintage and location, with the number of the genotypes indicated above the boxplot.

To investigate genetic mechanisms responsible for sensory wine quality, experimental wines produced from grapevine F1 individuals of the CM×VB cross-population were screened for descriptive and qualitative sensory attributes in a comprehensive evaluation. In total, 25,284 sensory profiles were determined based on 1,635 wine samples produced in 10 environments (six vintages in Siebeldingen and four in Neustadt). The score card of the tasters was adapted to the broad distribution of wine quality traits resulting from the high variance in the investigated population segregating for wine quality perception. The TQS, representative for the wine quality of the 147 F1 genotypes across the environments (all vintages and locations), showed an approximate normal distribution (**Figure 1A**). The 20th and 80th quantiles of the density curve were used to divide all genotypes into three quality groups (worst, average, and best). The parental cultivars were rated with an average (over all years and locations) TQS of 7.1 for CM and 5.3 for VB. Therewith, VB was rated comparable to the average of the F1 population, and CM has highest quality ratings. Within the “worst” group, only a few genotypes performed very poorly, and in the “best” group a small number of genotypes stand out for their particularly good quality rating. The genotypes within the defined quality groups performed reliably under the varying environmental influences of each vintage and location (**Figure 1B**).

#### 3.1.2 Role of the sensory attributes for wine quality

**Figure 2:**
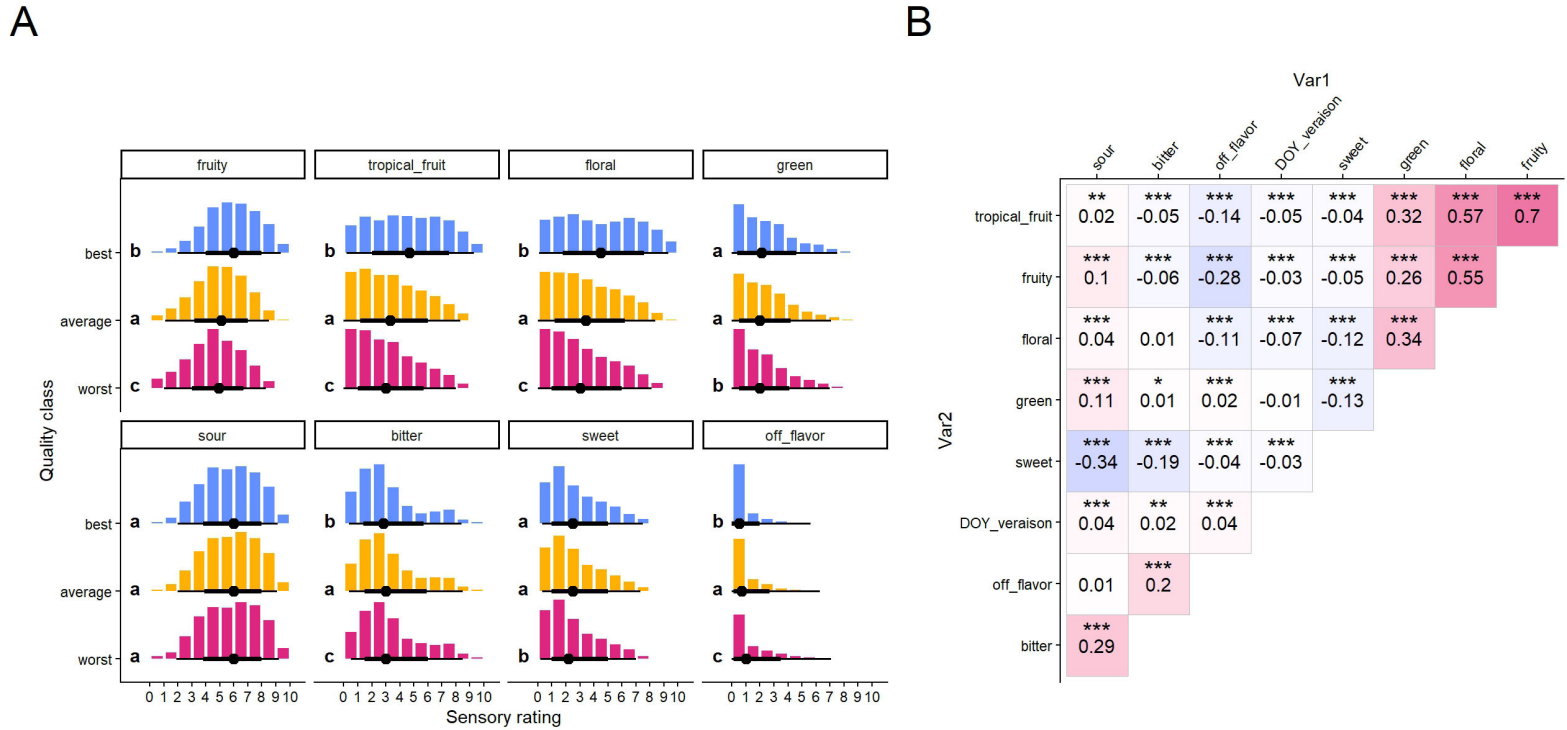
**(A)** Distribution of sensory attributes within the quality groups and their statistical differences indicated by the letters a, b, and c. The black dot indicates the mean value, and the varying line thickness represents the 66% and 95% intervals. **(B)** Pearson correlation of the sensory attributes and their significance from all collected sample evaluations (*p <.05, **p <.01, ***p <.001), complemented with the day of the veraison within the year (DOY).

In the course of the sensory evaluation, the focus was set on the eight descriptive and qualitative sensory attributes: fruity, tropical fruit, floral, green, sour, bitter, sweet, and off-flavor. For each attribute, the distribution of the rating appeared relatively similar within the three quality groups (**Figure 2A**), though, a Kruskal–Wallis test followed by a post-hoc Dunn’s test demonstrated significant differences between at least two of the quality groups for each attribute except sour (**Figure 2A**), indicating their importance in differentiating wine quality. Especially the attributes fruity, tropical fruit, floral, and off-flavor proved to be decisive for the classification. Some sensory attributes were found to be highly correlated with each other, suggesting that they may be measuring similar aspects of wine quality perception or are metabolically linked (**Figure 2B**). This emphasizes the underlying issue of non-independence between variables, giving reason for choosing the more complex Bayesian model. While most of the correlations according to Pearson are highly significant due to the large sample size, differences in the strength of interaction were observed. The day of veraison, which is very variable within this population, resulting in a harvest range of about 6 weeks between individuals, was found to not improve the sensory model, indicating a low impact on the final aroma perception in the produced wines.

**Figure 3:**
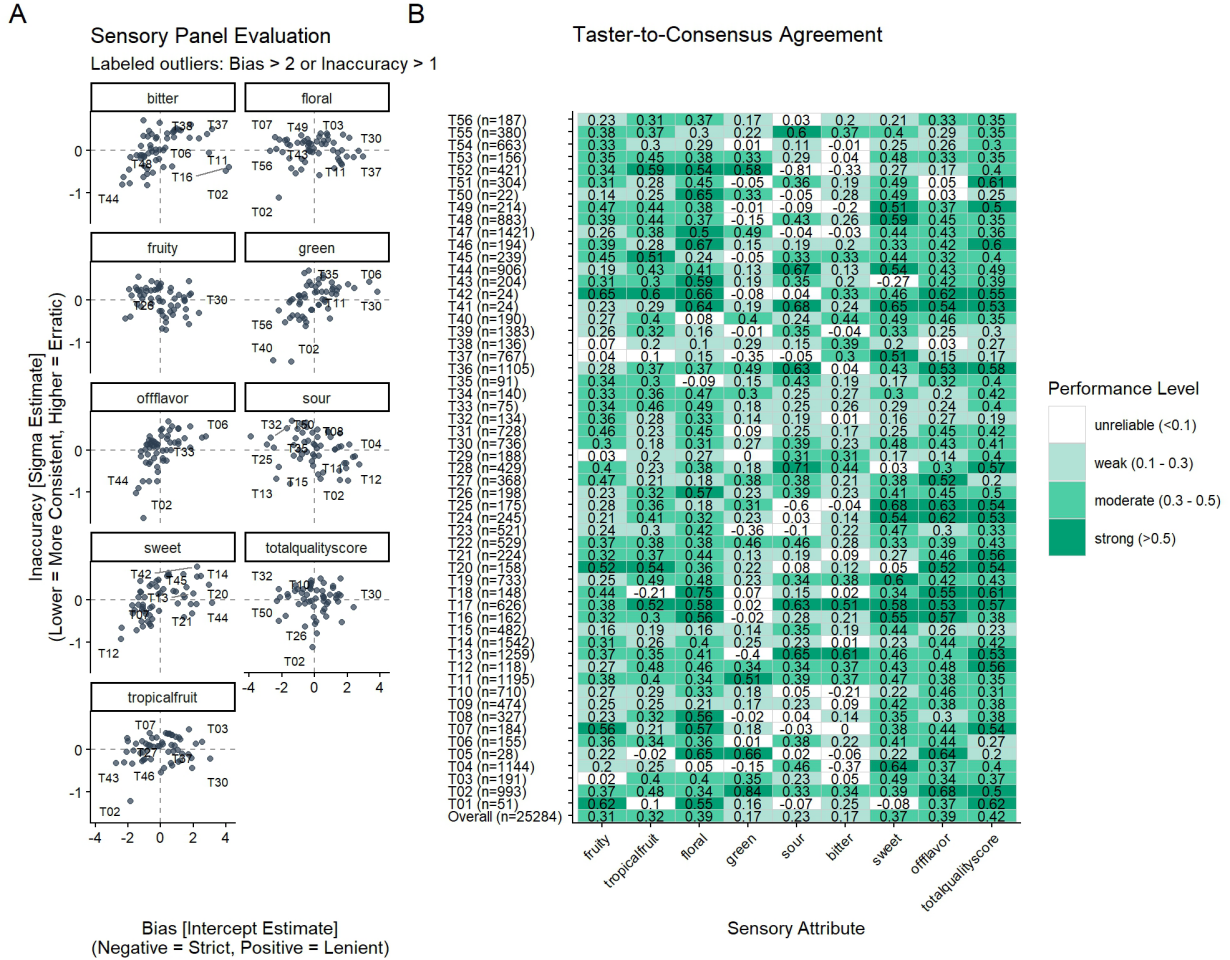
**(A)** Comparison of the taster estimation of the eight sensory attributes and the TQS of all 25,284 evaluations by the location and distributional part of the *brms* model. The x-axis shows the bias of the individual tasters of the panel (T01–T56). While tasters with negative x-values have a tendency toward lower ratings, others rate higher. The y-axis illustrates the varying accuracy of the tasters. **(B)** Taster-to-consensus agreement based on the Pearson correlation between the consensus score of each environment and each individual taster’s performance (*n* = number of wines evaluated by the respective taster over all sessions).

To verify the reliability of the sensory results, the obtained datasets were statistically analyzed through the *brms* model over the sessions and years. The intercept and sigma estimates illustrated the difficulties caused by internal bias and imprecision for each individual taster (**Figure 3A**). It is well known that tasters differ, even after thorough training, in the use of the intensity scales: some tasters rate, on average, two or more points lower or higher on the scale compared to the panel mean. The sigma estimate for each taster showed a wide range, indicating that some of them are less consistent within their ratings than others, thus harder to predict. The model predicted a consensus score for every environment (each sensory attribute for each wine in every session) based on all of the tasters’ assessments (**Figure 3B**). The taster-to-consensus agreement expressed the accordance of each individual taster for each attribute and each wine, in respect to each session they participated in, with this consensus score (Pearson correlation). To classify the correlations regarding sensory science, Cohen’s convention was utilized assuming that *r* ≈ 0.1 is weak, 0.3 is moderate, and 0.5 is strong (Cohen, 2009). The results show that there is a clear difference in the tasters’ accuracy. Some sensory attributes were identified to be more comprehensible for tasters to recognize and evaluate (e.g., floral, off-flavor, and sweet).

#### 3.1.3 Sensory-based G×E model

The multivariate Bayesian multilevel model was fitted to the eight descriptive sensory attributes and the TQS to separate and estimate the effects of genotype, environment, and individual taster’s subjectivity. The Markov Chain Monte Carlo (MCMC) convergence was assessed by visually inspecting trace plots and computing the Gelman–Rubin convergence diagnostic ( $\hat{R}$) for all parameters. The trace plots exhibited good mixing and stationarity (**Supplementary Files 3**). The model demonstrated excellent convergence, with all potential scale reduction factors at $\hat{R}>1.01$ and effective sample sizes exceeding 1,000 for all parameters. The model fit was assessed using Bayesian *R*², which estimates the proportion of variance explained by the fixed (environment) and random effects (genotype, taster, G×E). The model demonstrated a strong explanatory power, expressed as explained variance, for sensory attributes with the highest *R*² observed for sweet (0.578), sour (0.536), and bitter (0.511). Some sensory attributes showed moderate explanatory power, ranging from 0.309 (fruity) to 0.454 (green). The lower *R*² observed for off-flavor (0.223) suggested that the tasters’ perceptions were largely driven by random residual variation rather than systematic genotypic or environmental factors. All estimates were precise, with standard errors lower than 0.005. Furthermore, the predictive validity of the multivariate Bayesian model was calculated via leave-one-out cross-validation (LOO-CV) with 3,000 draws. Pareto-k diagnostics indicated a highly robust model fit. For the majority of sensory attributes, including fruity, tropical fruit, floral, green, and bitter, 100% of observations fell within the reliable predictive range (*k* < 0.7), indicating that no influential outliers were present. Minor deviations were observed for sweet (*n* = 6 outliers, 0.02%) and off-flavor (*n* = 16 outliers, 0.06%), where *k*-values exceeded the 0.7 threshold. The posterior predictive checks are robust across the scale for each attribute but also showed the trade-off from the Gaussian approximation necessary for the multivariate functionality. Due to the calculation methodology, the model predicted values outside of the given scale, so some estimations reached values under zero and over 10. We also observed the human bias to favor integers, known as “whole number bias” (Ni and Zhou, 2005), which is not reproduced by the model, so the calculated curve is smoother than the original data.

Variance decomposition revealed that the subjective influence of the tasters was the dominant source of variation across all attributes in this study, resulting in significantly outweighing genotypic effects. The identified standard deviations for the taster random intercepts ranged from 0.97 (off-flavor) to 1.52 (sour). Despite the previously mentioned assessor noise (σ ~ taster), distinct genotypic variance was detected, most notably for floral (σ ~ genotype = 0.39) and tropical fruit (σ ~ genotype = 0.38) aroma. The variance attributable to the G×E interaction was present but consistently lower than the observed main genotypic variance (e.g., for fruity, σ ~ genotype × enviroment = 0.04 vs. σ ~ genotype = 0.29). Analysis of residual correlations provided insight into the underlying associations of perceived quality. The TQS was strongly positively correlated with fruity (rho = 0.59), tropical fruit (rho = 0.52), and floral (rho = 0.48) intensities. Conversely, the perception of off-flavor showed a strong negative correlation with the TQS (rho = -0.54) and negative associations with the fruity descriptors. Interestingly, sour and bitter showed a moderate positive correlation with each other (rho = 0.23) but had negligible or weak negative correlations with the TQS, suggesting that these traits were secondary to fruity in driving the overall preference in this panel (all results and summaries are included in the **Supplementary Folder 3**).

### 3.2 Gas chromatography analysis

#### 3.2.1 Variance of volatile compounds in the population

**Figure 4:**
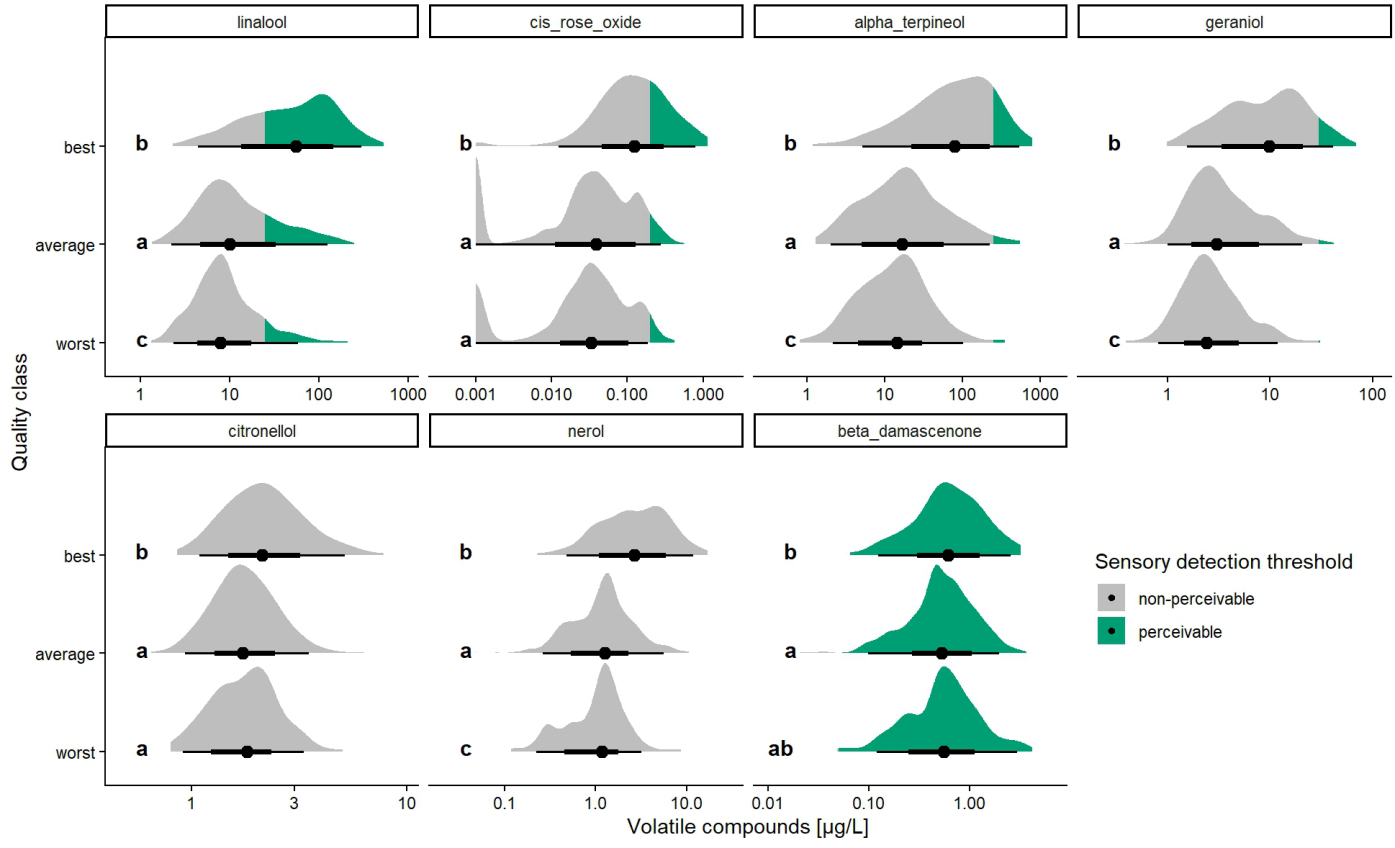
Density plots of concentration of volatiles, separated for the quality groups “best”, “average”, and “worst”, across all years and locations. The black dot represents the mean value, and the varying line thickness represents the line 66% and 95% intervals. The letters indicate statistically significant differences in compound concentrations among the groups (Kruskal–Wallis and Dunn’s *post-hoc*). The green-shaded area indicates the range of concentrations that are above the limit of detection perceivable by humans.

The density distribution of the concentration of volatiles measured by SIDA–GC–MS and separated by the three quality groups across all years and locations revealed that the highest concentrations of all monoterpenes were found to be in the quality group “best” (**Figure 4**). β-Damascenone is equally present in each group, in accordance with its role as a general aroma enhancer. In context to sensory detection thresholds (Waterhouse et al., 2024), the quality groups “average” and “worst” show sub-threshold levels for most of the investigated individual volatile compounds. Linalool and, to a lower extent, *cis*-rose oxide appeared to be the main monoterpenic drivers for the intensity of the floral attribute within the analyzed population, being abundant in the quality group “best” in concentrations above the threshold. On the other hand, citronellol and nerol appeared to be not perceivable as a single substance in the identified concentrations.

**Figure 5:**
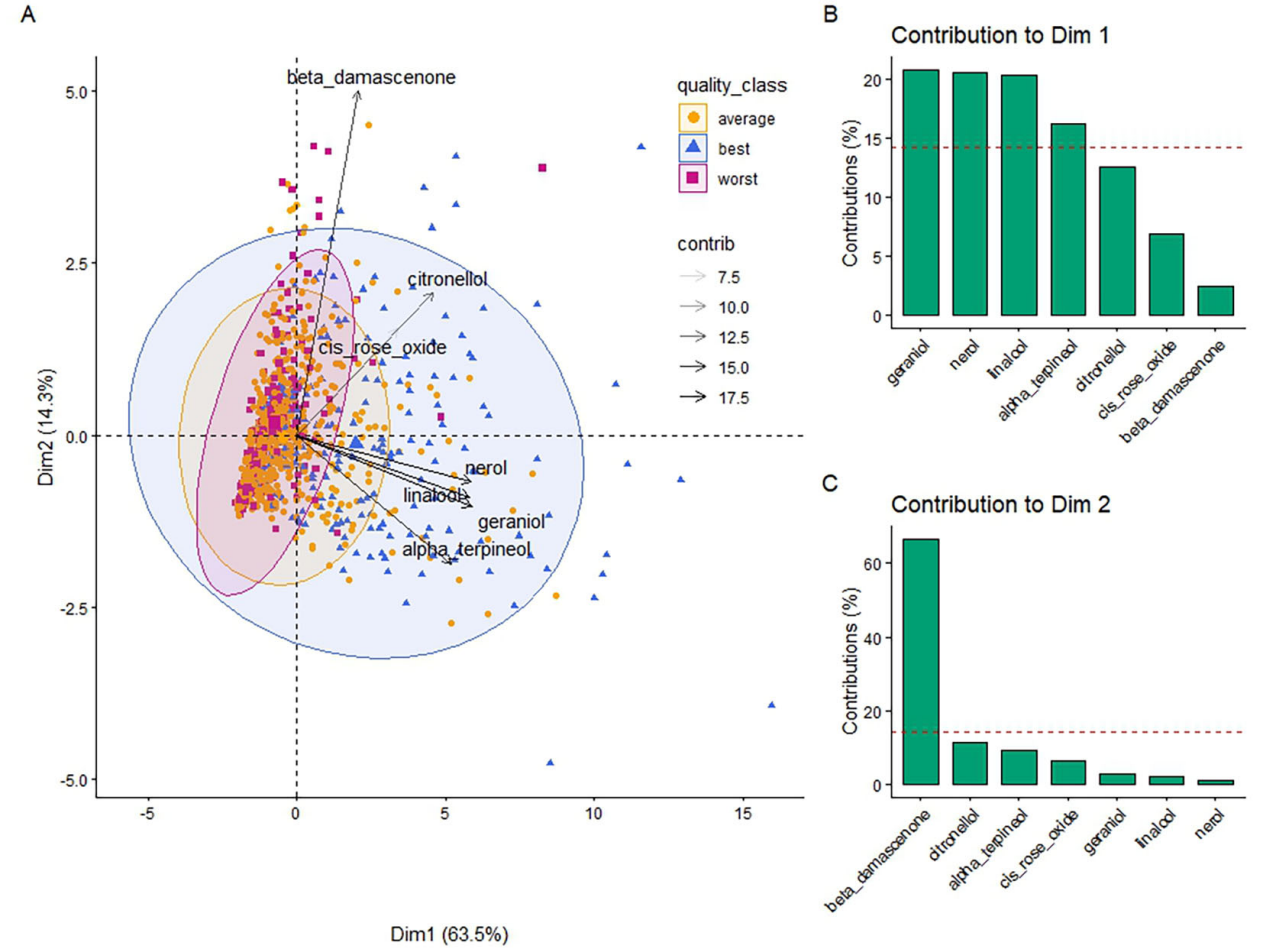
**(A)** Principal component analysis (PCA) biplot of volatile compound concentrations (SIDA-GC-MS data), complemented by information of the quality groups based on sensory data on the individuals of the CM×VB population. **(B)** Scree plot showing the contribution of variables to Dim 1 of the PCA. **(C)** Scree plot showing the contribution of variables to Dim 2.

**Figure 5A** shows the principal component analysis (PCA) biplot, which visualizes the relationships among all of the volatile aroma compounds measured in the wines of the CM×VB population. It also highlights how these compounds behave within the three quality groups. The first dimension (Dim1) captures the largest sources of variation for the genotypes, with 63.5% in the combined dataset, with the highest contribution given by geraniol—20.8%, linalool—20.3%, nerol—20.6%, and α-terpineol—16.2%. The sensory quality groups according to TQS, as indicated by the shifts in 95% ellipses, showed that the population mainly discriminates on this dimension along with the monoterpene concentrations. Dimension 2, with 14.3% explained variance, was mainly determined by β-damascenone (66.7%) and was found to be mostly independent from perceived sensory quality (**Figure 5B, C**).

#### 3.2.2 GC-based G×E model

The accumulation of monoterpenes highly depends on the length of ripening and weather conditions. Additionally, the Z-ratio for the genotype variance component from the *sommer* model exceeds the 1.92 significance threshold (ranging from 3.76 to 8.13), indicating a significant contribution of the genotype to the variation in the concentration of these aroma compounds and β-damascenone. Linalool and α-terpineol showed the largest absolute genotypic variance, while *cis*-rose oxide and β-damascenone showed the lowest values (**Table 1**). The 2019 vintage (especially 2019_SB) consistently resulted in increased concentrations for many monoterpenes, notably α-terpineol, geraniol, nerol oxide, and linalool. The site in Siebeldingen (SB) consistently promoted higher linalool concentrations. Specifically, the 2017_SB and 2019_SB environments showed significant positive effects, whereas the corresponding Neustadt (NW) environments for those years showed no significant deviation from the baseline. Conversely, the NW location in 2020 and 2021 was associated with significantly diminished monoterpene concentrations, suggesting that environmental factors specific to the NW site in these years may have limited their biosynthesis. *cis*-Rose-oxide, *trans*-linalool oxide, nerol oxide, citronellol, *β*-damascenone, linalool, and *α*-terpineol all show a negative coefficient. This indicates that a later veraison is associated with a lower concentration of these compounds, though the effect is often small and not significant for every compound. Later veraison is linked to cooler vintages, which may be linked to less sunshine hours, which have a negative effect on monoterpene biosynthesis in the grapes.

**Table 1:** Explained variances for the aroma compound concentrations from the *sommer* model and the calculated phenotype variance explained (PVE).

Aroma compound	Genotype variance	Residual variance	Z-ratio	PVE (%)
Linalool	6,642.1	768.91	7.94	0.90
α-Terpineol	12,706.26	2,653.21	7.58	0.83
*trans*-Linalool oxide (furanoid)	516.43	64.67	7.9	0.89
*cis*-Linalool oxide (furanoid)	286.72	21.16	8.13	0.93
Geraniol	97.87	17.26	7.7	0.85
Nerol oxide	30.28	4.14	7.85	0.88
Nerol	7.51	1.05	7.84	0.88
Citronellol	0.28	0.32	5.81	0.47
*cis*-Rose oxide	0.021	0.004	7.59	0.84
β-Damascenone	0.041	0.21	3.76	0.16

### 3.3 QTL analysis results

Based on the genotype effect standard deviations (SD) from the Bayesian multivariate multilevel model, the sensory attributes sour (SD = 0.03), bitter (SD = 0.03), and green (SD = 0.06) showed a very small variation attributable to the genotype in the CM×VB population. They were therefore excluded from the QTL analysis. The sensory attributes with the largest genotype-explained variation were found to be floral (SD = 0.39), tropical fruit (SD = 0.38), and fruity (SD = 0.29).

**Figure 6:**
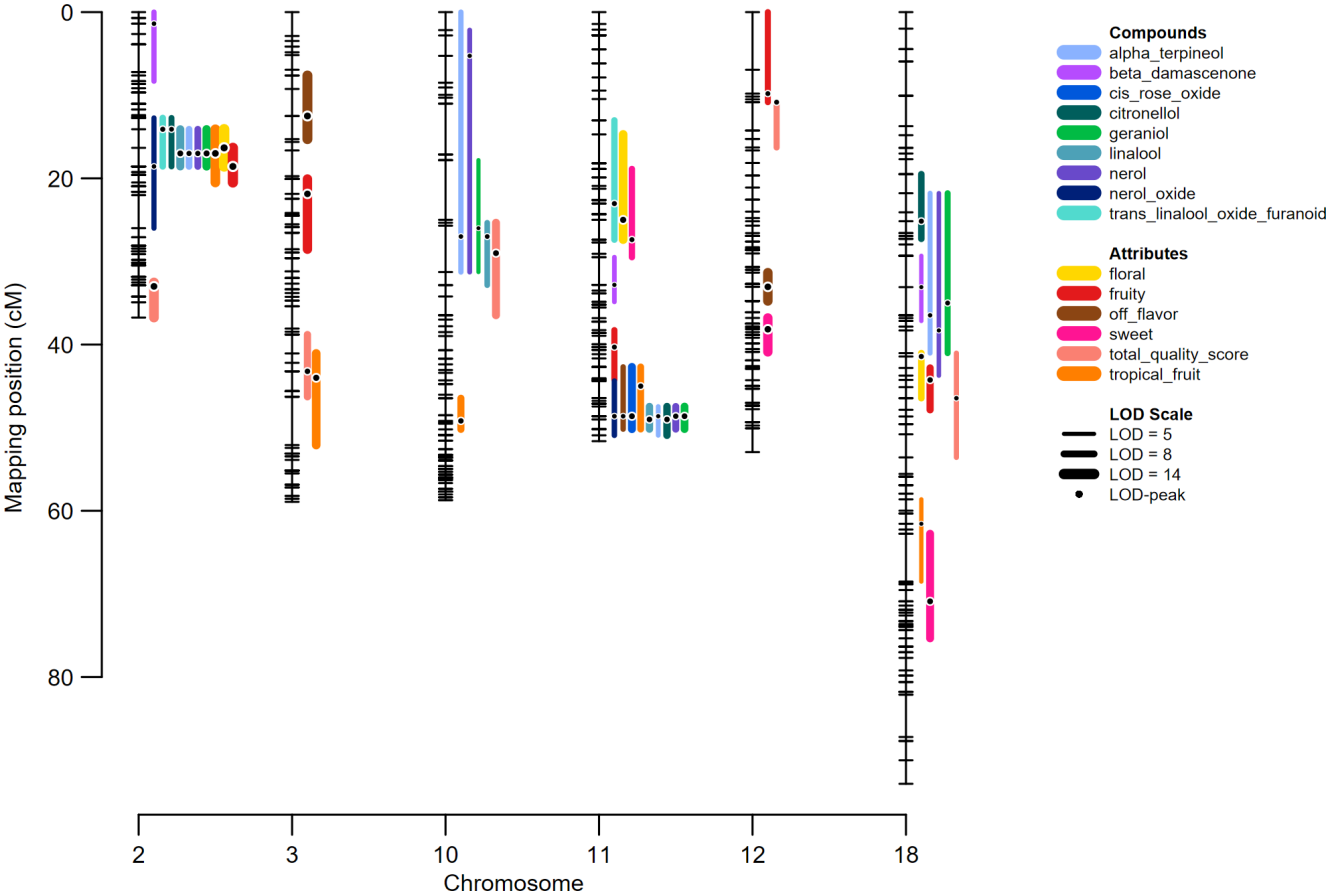
Curated QTLs for sensory attributes and volatile aroma compounds in the population CM×VB. The length of the segments are marking the QTL intervals (LOD-1) from the CIM, with their width indicating the strength of the LOD_max_ value (logarithm of the odds).

Composite interval mapping (CIM) analysis was carried out based on the HBM genetic map with the best linear unbiased predictors (BLUPs) calculated using the *brms* (sensory data) and *sommer* models (SIDA-GC-MS data) as phenotypic data. In total, 33 QTLs for volatile compounds and 41 QTLs for sensory attributes were identified across nearly all chromosomes (chr) (**Supplementary Files 4**). QTLs with overlapping confidence intervals for sensory attributes and metabolite concentrations were identified, revealing genetic loci of particular interest that control an important part of the complex trait of wine quality. Specifically, two major QTL clusters or QTL stacks were observed on chromosome 2 and chromosome 11, indicating regions with a significant, pleiotropic impact, likely on terpene biosynthesis (**Figure 6**).

Chr2 hosts a highly significant QTL stack (LOD_max_ up to 14.8/PVE up to 19.7%) for all of the major monoterpenes quantified, namely: geraniol (10.6/17.4%), linalool (10.1/19.7%), nerol (8.3/14.5%), α-terpineol (8.2/16.8%), *trans*-linalool oxide (furanoid) (7.9/18.3%), citronellol (7.3/11%), and nerol oxide (6.5/15.1%). Crucially, these QTLs overlap directly with QTLs identified for the most discriminating sensory attributes, namely: floral (14.8/14.3%), fruity (14.4/17.2%), tropical fruit (13.1/16.3%), and the TQS (13.13/12.2%). A second major QTL stack was identified on chr11, which also harbors strong QTLs for several measured monoterpenes and quality-related sensory attributes, including floral (11.1/10.9%), tropical fruit (8.3/8.9%), and fruity (7.5/6.0%). Additionally, this region includes QTLs for sweet (7.7/9.2%) and off-flavor (6.7/9.0%), suggesting a broader genetic influence on overall wine quality. On further chromosomes, additional QTLs were identified—some with overlapping intervals and some isolated—for example, QTLs for off-flavor were detected on chr3 (13.7/11.7%) and on chr12 (12.8/13.0%), the latter overlapping with QTLs for sweet (12.6/14.1%). For the total quality score, QTLs were identified on chr3 (8.9/8.4%), chr10 (10.0/8.5%), and chr18 (6.5/6.8%).

### 3.4 Candidate gene search

For candidate gene screening within the identified QTL regions, the functional annotation by The Grapevine Genomics Encyclopedia (grapedia.org 2026) using PANTHER (Thomas et al., 2022) based on the v5.1 T2T PN40024 reference genome (Shi et al., 2023) was used. The developed markers were aligned to the updated reference genome, and the positions were included in **Supplementary File 4**. Screening was based solely on positional annotation. Due to the abundance of identified QTLs, the candidate gene search was restricted to the two regions of the very prominent QTL stacks on chr2 and chr11 (**Table 2**). The interval of the chr2 QTL stack (LOD-1, position in T2T PN40024: 3201643–3919651 bp) harbors 102 genes in total. Among these, a cluster of seven genes—*Vitvi05_01chr02g04810*, *Vitvi05_01chr02g04840*, *Vitvi05_01chr02g04860*, *Vitvi05_01chr02g04950*, *Vitvi05_01chr02g05320*, *Vitvi05_01chr02g05330* and *Vitvi05_01chr02g05310*—with similarities to CYTOCHROME P450, FAMILY 76 stood out. Another candidate gene, *Vitvi05_01chr02g05160*, putatively encodes a MYB PROTEIN-RELATED-MYB TRANSCRIPTION FACTOR. The interval of the QTL stack on chr11 (LOD-1, position in T2T PN40024: 16812781–20026417 bp) holds 319 genes in total. Three candidate genes—*Vitvi05_01chr11g18380*, *Vitvi05_01chr11g19030*, and *Vitvi05_01chr11g19050*—are discussed later in detail for the putative function of 1-deoxy-D-xylulose-5-phosphate-synthase.

**Table 2:** Candidate genes within the LOD-1 confidence intervals of the QTL stacks for volatile aroma compounds and sensory attributes on chromosome 2 and chromosome 11.

Gene ID	Chr.	Gene position in v5.1 T2T PN40024 [bp]	PANTHER ID
*Vitvi05_01chr02g04810*	2	3336902-3339276	PTHR47950 (‘CYTOCHROME P450, FAMILY 76, SUBFAMILY C, POLYPEPTIDE 5-RELATED’)
*Vitvi05_01chr02g04840*		3347447-3349841	
*Vitvi05_01chr02g04860*		3358045-3360328	
*Vitvi05_01chr02g04950*		3425113-3427593	PTHR24286 (‘CYTOCHROME P450 26’)
*Vitvi05_01chr02g05160*		3570423-3578782	PTHR45614 (‘MYB PROTEIN-RELATED-MYB TRANSCRIPTION FACTOR’)
*Vitvi05_01chr02g05310*		3667591-3669398	PTHR47950 (‘CYTOCHROME P450, FAMILY 76, SUBFAMILY C, POLYPEPTIDE 5-RELATED’)
*Vitvi05_01chr02g05320*		3672000-3673812	
*Vitvi05_01chr02g05330*		3675480-3675881	
*Vitvi05_01chr11g18380*	11	19205407-19210544	PTHR43322 (‘1-D-DEOXYXYLULOSE 5-PHOSPHATE SYNTHASE-RELATED-DEOXYXYLULOSE-5-PHOSPHATE SYNTHASE’)
*Vitvi05_01chr11g19030*		19775082-19780556	
*Vitvi05_01chr11g19050*		19818146-19822100	

The complete list of annotated genes in the QTL stacks can be found in **Supplementary Files 4**.

## 4 Discussion

### 4.1 Quality-determining factors in the ‘Calardis Musqué’ × ‘Villard Blanc’ population

Wines of Calardis Musqué are characterized by intense floral, fruity, and tropical fruit flavors as well as pronounced acidity, whereas wines of Villard Blanc are nearly neutral in their aroma with subtle notes of citrus. The wines of the F1 individuals segregate for quality traits, especially muscat flavor (fruity and floral) with the responsible monoterpenes, making the cross-population CM×VB a perfect resource to study underlying genetic determinants. In this study, the attributes fruity, tropical fruit, and floral and the critical off-flavor strongly separates the population in terms of quality, while attributes like sour, bitter, and green had no discernible discriminating impact. The attributes fruity, tropical fruit, and floral are perceived as pleasant (Khan et al., 2007) and are clearly associated with high levels of monoterpene concentration (**Figure 5**). As the F1 individuals widely vary within the veraison timeframe and the monoterpenes are known to rise with maturity (Gunata et al., 1985), close monitoring of berry maturity and the criterion of equal maturity for determining harvest dates allowed the impact on monoterpene concentrations in the wine samples to be minimized. Thus, timing of the veraison had a minimal effect on the floral and fruity sensory perception (**Figure 2B**), and monoterpenes were only slightly diminished in later ripening individuals. Additionally, despite the significant impact of vintage and location on absolute monoterpene concentrations and their sensory perception, the relative ranking of the best and worst genotypes remained stable across the different environments. This confirms the high genetic predisposition for the most relevant quality parameters. To dissect the role of monoterpenes further, the SIDA–GC–MS analysis provided a quantitative basis for the sensory observations by measuring 10 key volatile compounds, including monoterpenes and β-damascenone, which served as the molecular basis to confirm the sensory-derived QTLs.

Not all measured concentrations of aromatic compounds were above the respective sensory detection threshold. Nevertheless, it is known that some monoterpenes show synergistic effects at individual sub-threshold levels in wine with other monoterpenes (Ribereau-Gayon et al., 1975) and with other aroma compound classes of fruity descriptors (Garbay et al., 2025). While geraniol, nerol, and linalool showed the highest contribution in dividing the quality groups, only linalool concentrations were measured above the sensory detection thresholds. This finding confirmed linalool as the most relevant metabolomic marker of aroma quality in the studied CM×VB population. β-Damascenone is known for showing interactive sensory effects with a wine’s aromatic matrix, especially with monoterpenes and fruity esters formed during fermentation (Garbay et al., 2025; Pineau et al., 2009). Therefore, it was quantified also and was found to be well above the sensory threshold in all quality groups. Thus, variations in β-damascenone concentration seem to have no direct impact on the wine quality gradient in this specific study. While the model calculated with *sommer* was sufficient for the exploration of the GC data, the sensory dataset profited from an additional approach.

### 4.2 Benefits of a multivariate Bayesian modeling for processing sensory data

Implementing a Bayesian multivariate multilevel model using the *brms* package (Bürkner, 2017) provided a significant methodological advantage over traditional linear modeling. The latter typically assumes independence and constant variance, which are not applicable for the present dataset. Additionally, the *brms* package was able to handle population structures like the kinship matrix. A taster’s individual sensitivity toward descriptive attributes and immanent subjective evaluation of quality parameters were identified as the dominant source of variation across all attributes, consistently outweighing genotypic effects. This underscores the critical need for advanced statistical methods to account for human bias in sensory studies.

A standard linear model, such as a linear regression or ANOVA for each trait individually, fails to capture the intricate structure of sensory data such as high correlation among attributes, hierarchical grouping, and systematic taster biases (Schielzeth et al., 2020; Kuznetsova et al., 2015). By contrast, the multivariate model allowed for the joint modeling of all nine sensory traits, including the TQS, which not only estimated the true relationships between these results but also substantially improved the overall statistical power of the analysis. The specification of a sub-model for the residual standard deviation explicitly accounts for differences in taster consistency and perception sensitivity, thus the heteroscedasticity. This is an important feature to handle the varying skill levels and sensory accuracy within human panels. Furthermore, estimating a full residual correlation matrix captures the non-independence and correlations between the nine sensory attributes, which persists even after all fixed and random effects have been factored out (Mathew et al., 2018).

Such complex models are practical today thanks to open-source tools like *brms*, the probabilistic programming language Stan, and the increased computational capacity and ease of use of modern statistical software. Finally, the results generated from the Bayesian multivariate multilevel model can be effectively utilized as a direct feedback loop for both the individual tasters and the improvement of the sensory methodology—for example, the analysis of residual correlations revealed perceived relationships between the attributes. The strong positive correlations identified between the sensory descriptors fruity (rho = 0.59), tropical fruit (rho = 0.52), and floral (rho = 0.48) indicate a high degree of co-dependence or a perceptual overlap among these key quality attributes. This insight can be used to refine the descriptive analysis, revealing areas where sensory perception is impacted by psychological contrasts or molecular masking effects and highlighting the structural advantages of sensory data over traditional univariate models.

### 4.3 QTL analysis and potential gene candidates

Gaining knowledge about the underlying genetic factors controlling the biosynthesis of wine-quality-related aromatics is a rather sophisticated task. Difficulties due to a noticeable number of initially odorless grape-derived aroma precursor compounds and the huge impact of the winemaking and fermentation process complicate the investigations. Finally, the high number of identified QTLs for the measured volatile monoterpenes and the sensory attributes distributed across nearly all chromosomes of grapevine in this study emphasizes the complexity of genetic control of wine quality and at the same time demonstrates the successful experimental approach.

Several QTLs were detected for the total quality score (e.g., on chr3, chr10, and chr18). Identification of genetic loci contributing to an overall positive wine quality perception is a novel approach and opens up new prospects for breeding. Multiple QTLs (chr3, chr11, and chr12) were identified for the attribute off-flavor, suggesting genetic determinants with potential negative impact in the CM×VB population. Studying the genetic basis of off-flavor is particularly challenging without knowledge of its specific nature and the responsible compounds. On the one hand, this attribute is shaped by exogenous factors, like contaminated natural cork closures or smoke taint; on the other hand, it is influenced by endogenous sources originating from grapes and winemaking, such as untypical aging flavor, petrol notes, excessive volatile acidity, or reductive off-flavors (Waterhouse et al., 2024). Thus, identifying QTLs linked to the off-flavor attribute reveals genetically influenced factors and opens up new approaches to further research. A more detailed descriptive analysis to specify the off-flavors in this population in combination with an untargeted metabolomic approach could identify and pinpoint underlying compounds.

For breeding purposes, genetic regions with a pleiotropic impact on several favorable traits can be particularly interesting. The QTL stack on chromosome 2 demonstrates clear co-localization for all major quantified monoterpenes, overlapping with QTLs for the most discriminating sensory attributes: floral, fruity, tropical fruit, and the total quality score. A second major QTL stack on chromosome 11 similarly showed strong co-localization for measured monoterpenes and quality-related sensory traits (floral, tropical fruit, and fruity) and also included QTLs for sweet and off-flavor, indicating a possible sensoric masking effect. These QTL stacks reveal regions with genetic control of monoterpene biosynthesis, leading to the perception of favorable sensory attributes. Detection of the same regions in respect to monoterpene concentrations and sensory attributes validates the robustness of the processed sensory evaluation data. In other studies, QTLs for muscat score have been found on chr1, chr 5, and chr 7 (Doligez et al., 2006) with a co-located QTL for monoterpene content on chr1 and chr5. The QTLs for nerol and geraniol were found on chr13 and for linalool on chr2 (Doligez et al., 2006). In addition to chr5 for monoterpene content, chr10 also seems to be prevalent in other studies, especially those mentioning linalool (Battilana et al., 2009; Duchêne et al., 2009). The confirmation of some QTL regions in this study reinforces their impact on monoterpene biosynthesis.

On chromosome 11, three candidate genes (*Vitvi05_01chr11g18380*, *Vitvi05_01chr11g19030*, and *Vitvi05_01chr11g19050*) putatively code for a 1-deoxy-D-xylulose-5-phosphate-synthase. This enzyme plays a key role in the metabolic flux control in plastidial monoterpene biosynthesis at the beginning of the MEP pathway in plants (Tholl, 2015). Two five-carbon (C_5_) units of isopentenyl pyrophosphate (IPP) and its isomer dimethylallyl diphosphate (DMAPP) form the backbone of all monoterpenes and sesquiterpenes. In plants, isoprenoid units are synthesized by two independent pathways. While the mevalonic acid (MVA) pathway takes place in the cytoplasm of the berry cells, the methylerythritol 4-phosphate (MEP) pathway is located in the plastid (McGarvey and Croteau, 1995; Lichtenthaler, 1999). Previous analyses of muscat × neutral mapping populations revealed that the expression of the *V. vinifera* 1-deoxy-D-xylulose-5-phosphate-synthase gene (*VviDXS*) is associated with monoterpene biosynthesis in muscat varieties and aromatic muscat-like mutants. This gene was linked to a QTL on chromosome 5 which described 17%–93% of the variation found for the monoterpenes linalool, nerol, and geraniol (Emanuelli et al., 2010). Furthermore, the genome−wide annotation and functional characterization of the *V. vinifera* terpenoid synthase genes (*VvTPS*) by Martin et al. (2010) led to the prediction of 69 putative *VvTPS* genes across the grapevine genome. Overall, the authors demonstrated the large potential of the *VvTPS* gene family for variation in the terpene metabolism and provided strong evidence for the complexity of wine quality patterns. The Myb transcription factor predicted for *Vitvi05_01chr02g05160* could also play a role in terpene accumulation, as MYB24 was suggested to bind to the promoters of 22 terpene synthases in grapevine (Zhang et al., 2023). In the interval of the chr2 QTL stack, a cluster of seven genes (*Vitvi05_01chr02g04810*, *Vitvi05_01chr02g04840*, *Vitvi05_01chr02g04860*, *Vitvi05_01chr02g04950*, *Vitvi05_01chr02g05320*, *Vitvi05_01chr02g05330*, and *Vitvi05_01chr02g05590*) was predicted as cytochrome P450, sub-family 76. Bicyclic monoterpene lactones are derived from the crucial precursor (*E*)-8-carboxylinalool during wine aging (Giaccio et al., 2011; Ilc et al., 2017). The synthesis of (*E*)-8-carboxylinalool involves multiple steps (hydroxylation, dehydrogenation, and carboxylation), which are catalyzed by a cytochrome P450 enzyme VvCYP76F14 in grapevine (Lin et al., 2019; Peng et al., 2024). The CYP76 family enzymes act as a multifunctional monooxygenase capable of catalyzing multiple substrates (Paine et al., 2005). A comparable enzyme in *Arabidopsis thaliana*, AtCYP76C1, demonstrates this versatility by converting linalool into various oxidized derivatives and lilac compounds (Boachon et al., 2015; Kunert et al., 2023). Further investigation should focus on the functional analysis of the discovered candidate genes by expression studies to prove their impact. The identification of strong candidate genes within the QTLs underline their functional importance for monoterpene formation and quality relevance. This knowledge allows breeders to select high-aroma genotypes at the earliest seedling stages and to accelerate the whole breeding process.

## 5 Conclusions

This study established a robust, data-driven basis for the next generation of grapevine breeding with a focus on quality traits. The key achievement is the translation of complex, human-perceived intensity of sensory attributes and holistic wine quality ratings of different environments (location and year) into precise phenotypic data. This was reached by applying a Bayesian multivariate multilevel model using the *brms* package to discover new genetic loci in a subsequent QTL analysis. Several reliable QTLs for the assessed sensory attributes and quantified monoterpenes were identified in the genome of grapevine. The two prominent QTL stacks on chromosomes 2 and 11 represent exceptional targets for marker-assisted selection, as they are associated with the core quality-driving traits: floral, fruity, tropical fruit, and the TQS, as well as important volatile monoterpenes. In a novel approach, genetic regions associated with an overall positive perception of wine quality, as calculated by the TQS, were identified. This allowed us to move beyond the selection of single compounds to a more integrated phenotyping approach. Using TQS-related molecular markers in marker-assisted selection maximizes the overall wine quality potential of grapes in selected breeding lines and greatly increases breeding efficiency.

## Data Availability

The datasets presented in this study can be found in online repositories. The names of the repository/repositories and accession number(s) can be found in the article/**Supplementary Material**.

## Footnotes

^1^ Resolution OIV-OENO 671A-2025 Revision of the “OIV standard for internationa wine and spirituous verages of vitivinicultural orgin competitions“. (2025). Available online at: https://www.oiv.int/index.php/node/4087 (Accessed June 15, 2026).
