# Supplementary material for: A multivariate Bayesian modeling strategy coupled with QTL analysis reveals genetic loci linking important sensory wine quality attributes with their corresponding wine aroma compounds
Source: Front Plant Sci. 2026 Jun 26;17:1851889. doi: 10.3389/fpls.2026.1851889 (PMC13350352; doi:10.3389/fpls.2026.1851889)

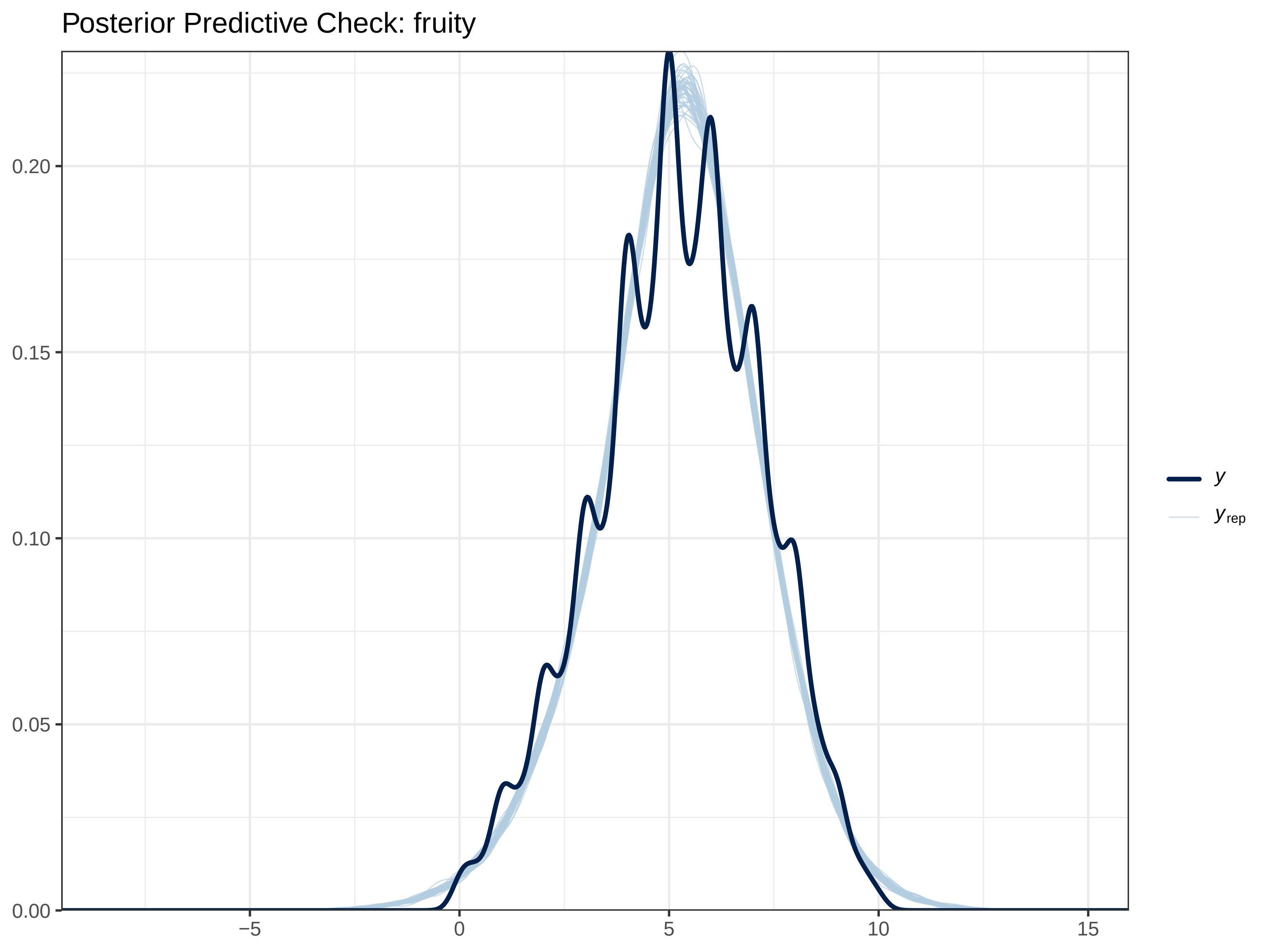
Posterior Predictive Check: fruity
0.00
0.05
0.10
0.15
0.20
−5
0
5
10
15
y
y rep

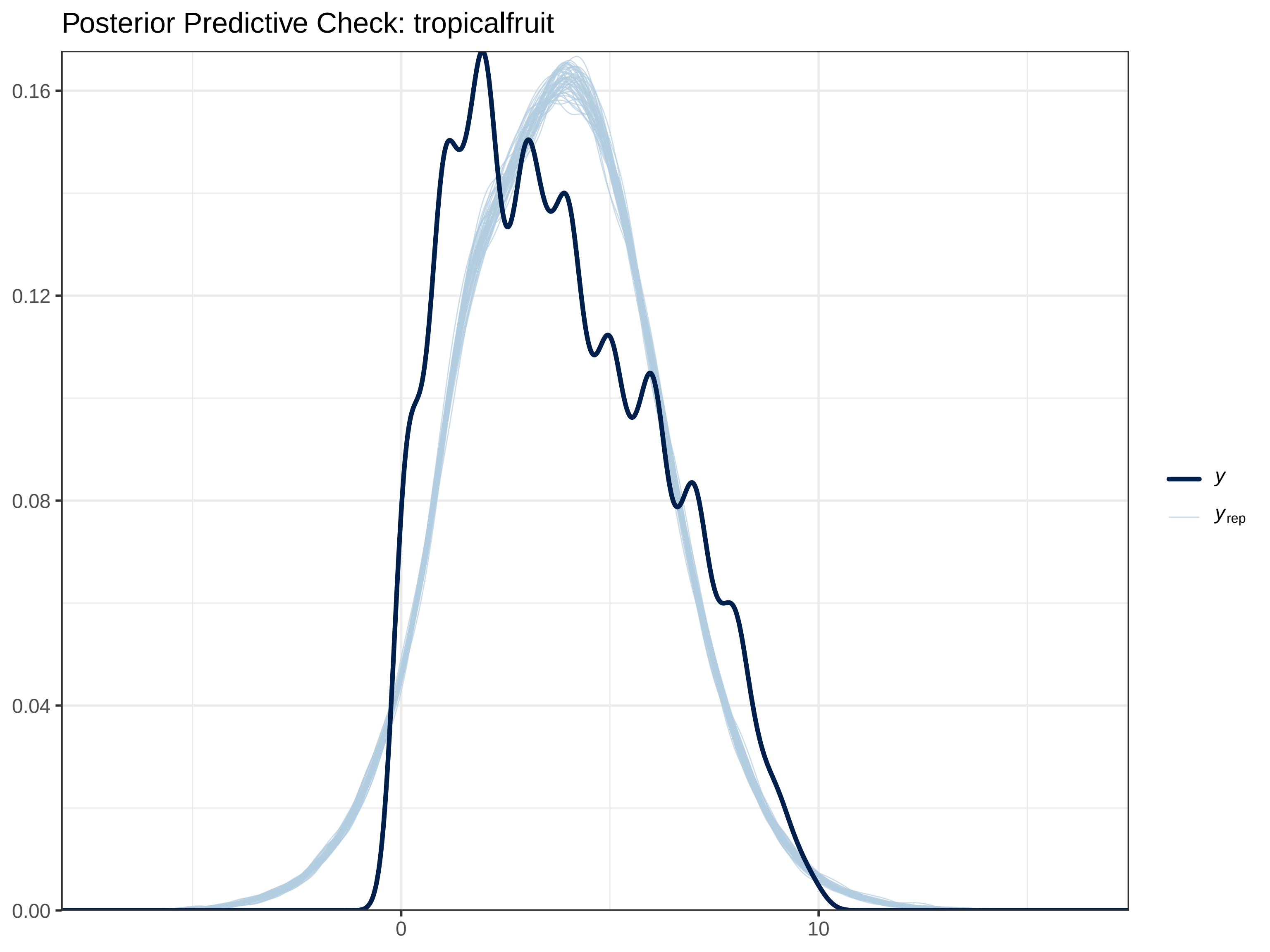

Posterior Predictive Check: tropicalfruit
0.16
0.12
0.08
0.04
0.00
0
10
y
y rep

Posterior Predictive Check: floral

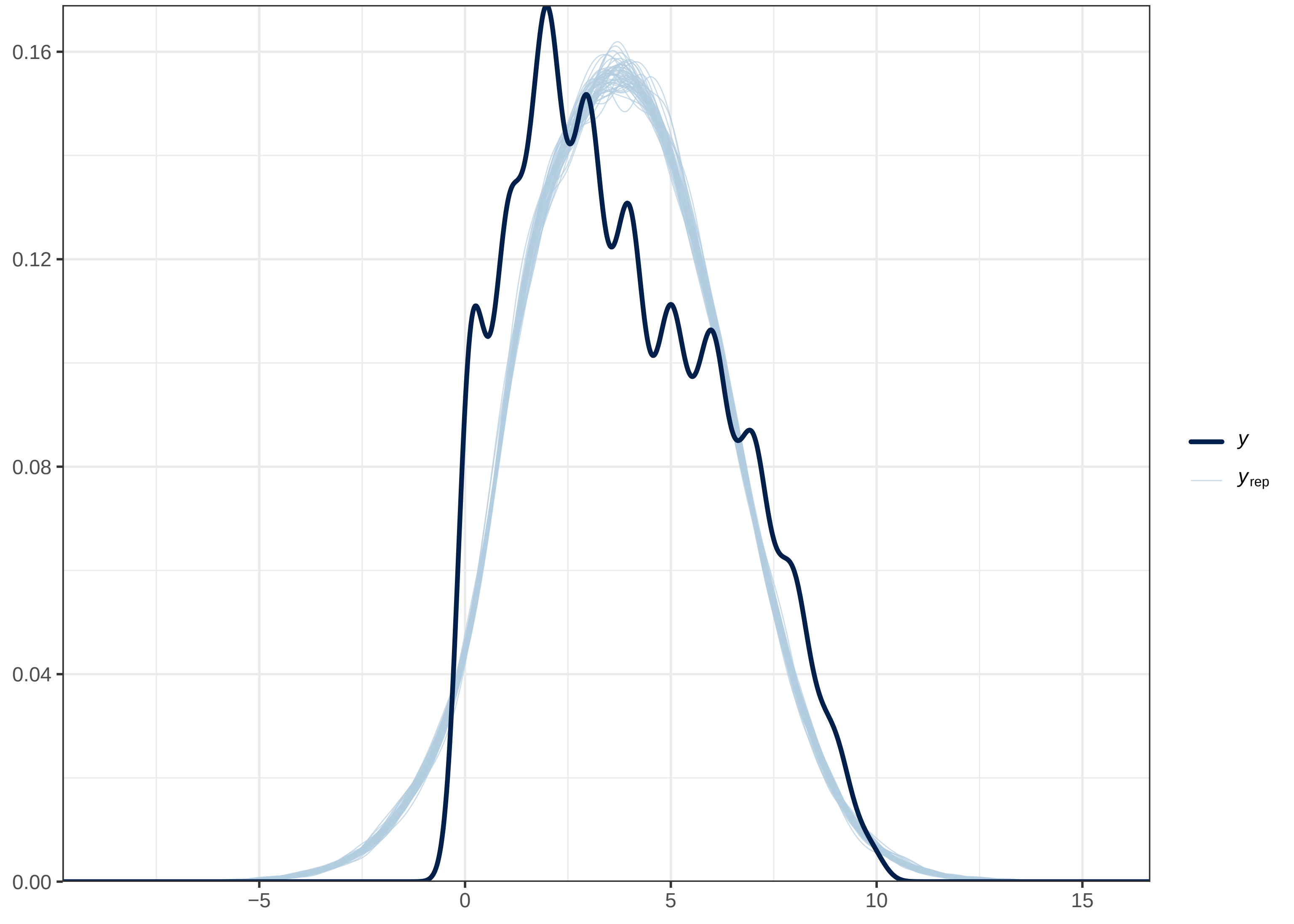

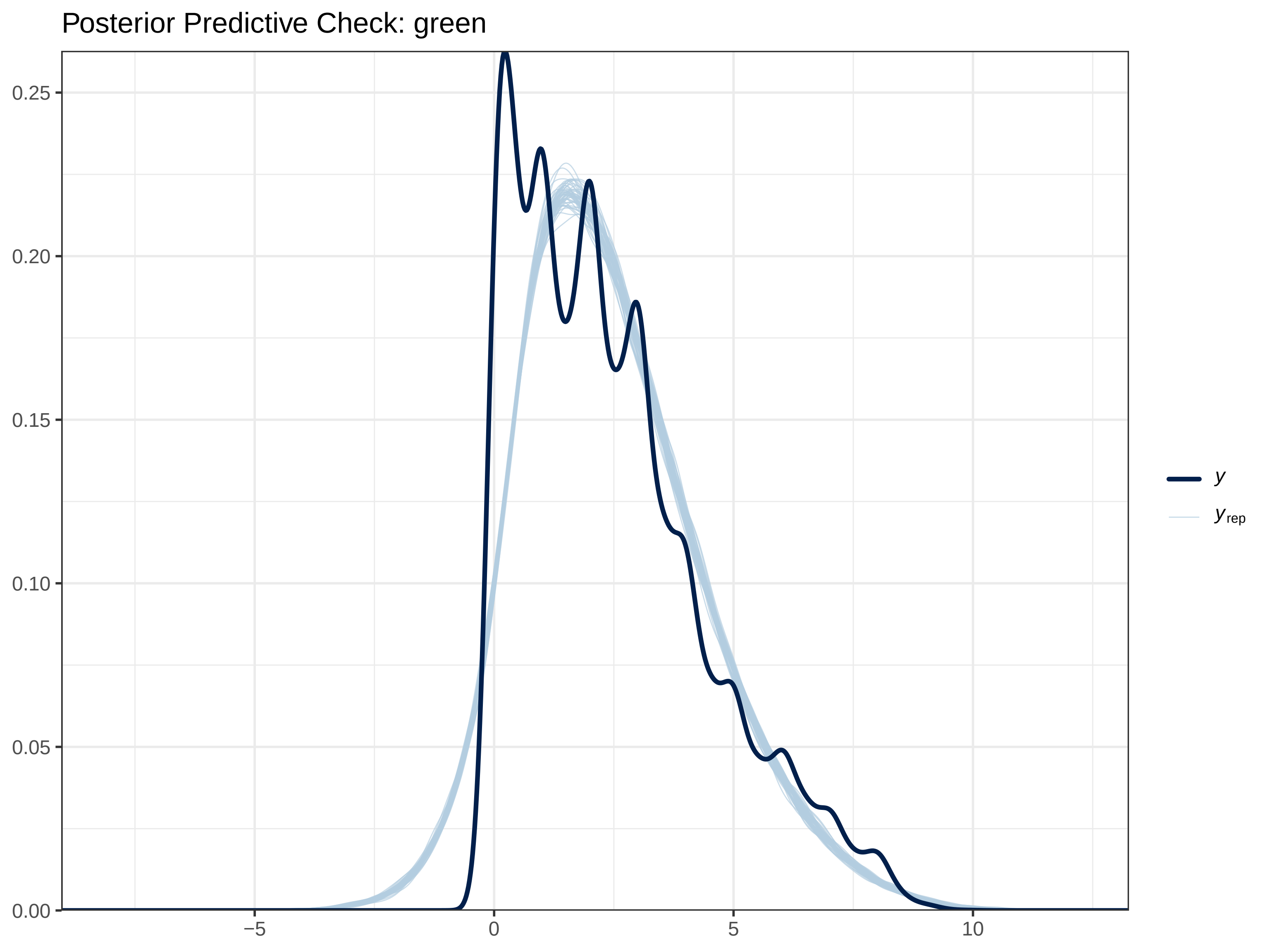
Posterior Predictive Check: green
0.25
0.20
0.15
0.10
0.05
0.00
−5
0
5
10
y
y rep

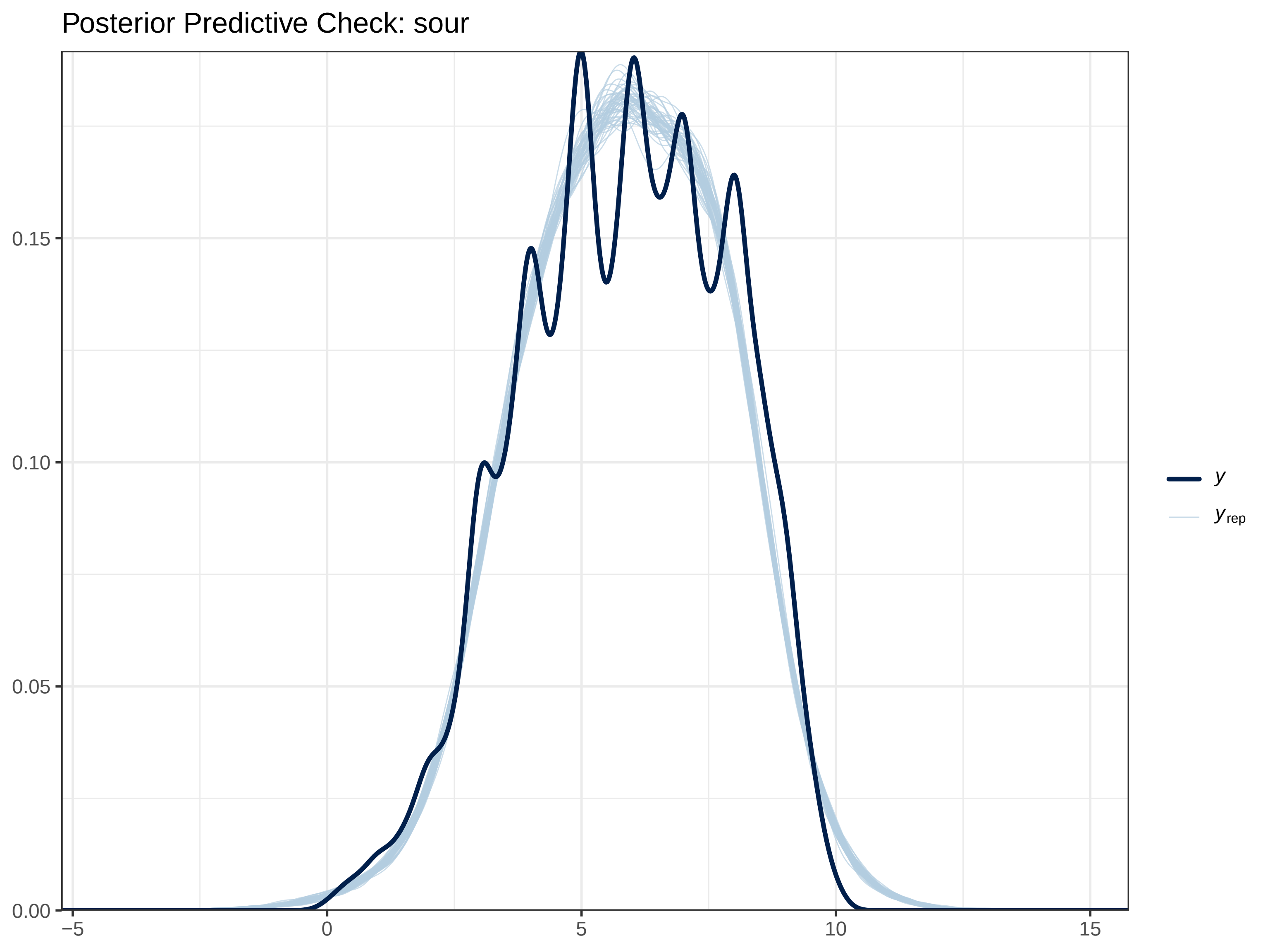
Posterior Predictive Check: sour
0.00
0.05
0.10
0.15
−5
0
5
10
15
y
y rep

Posterior Predictive Check: bitter

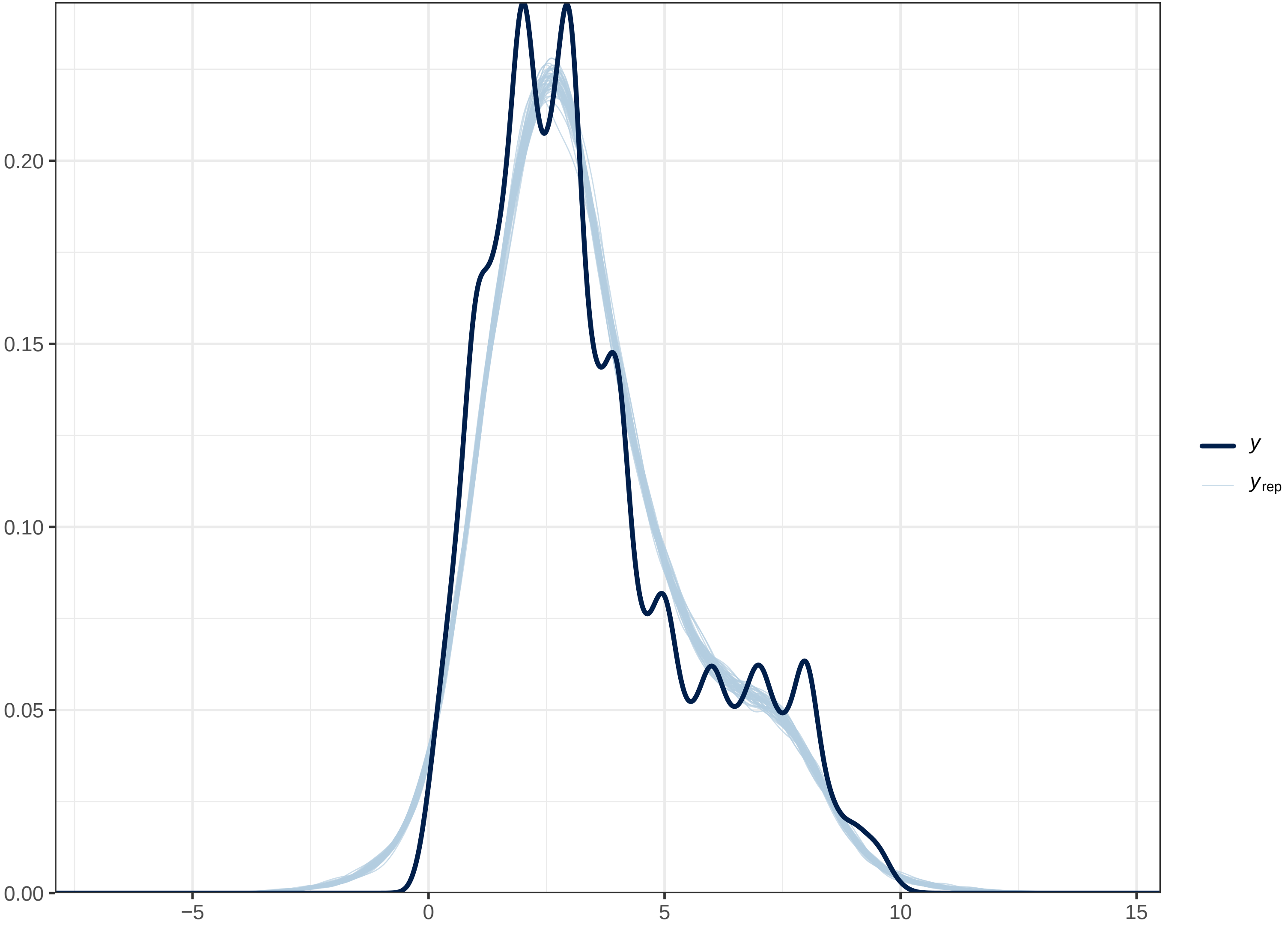

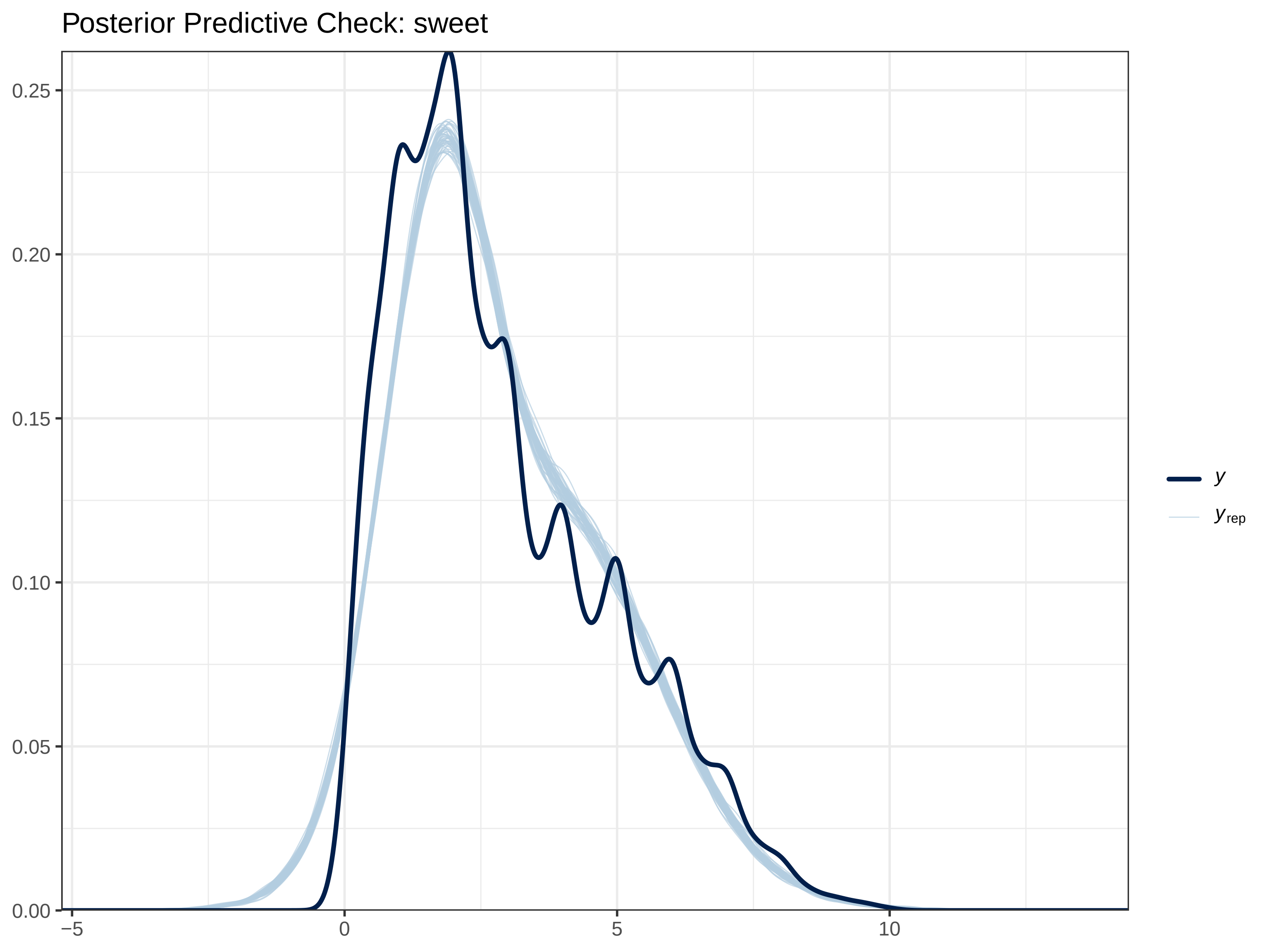
Posterior Predictive Check: sweet
0.25
0.20
0.15
0.10
0.05
0.00
−5
0
5
10
y
y rep

Posterior Predictive Check: offflavor

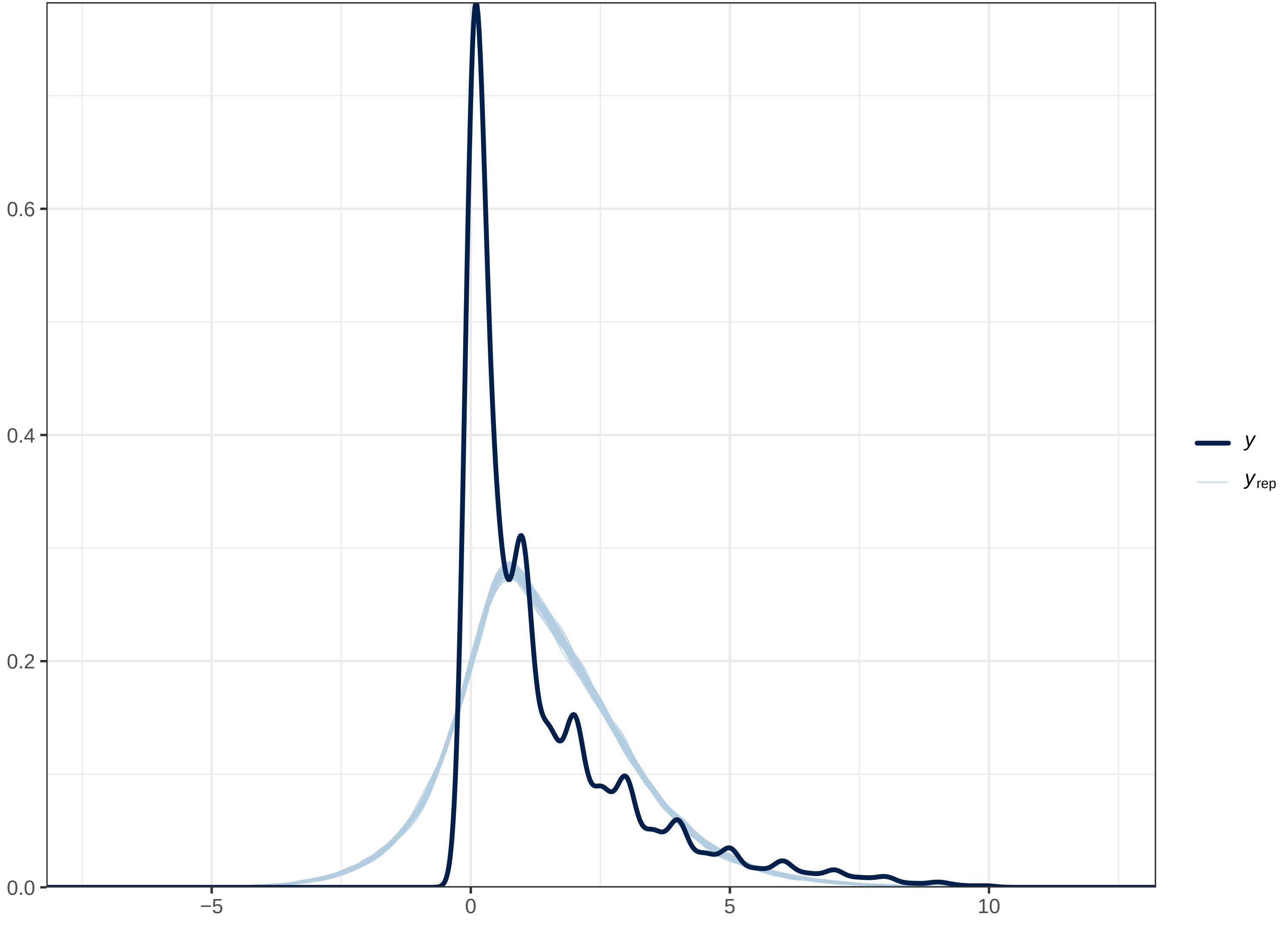

Posterior Predictive Check: totalqualityscore

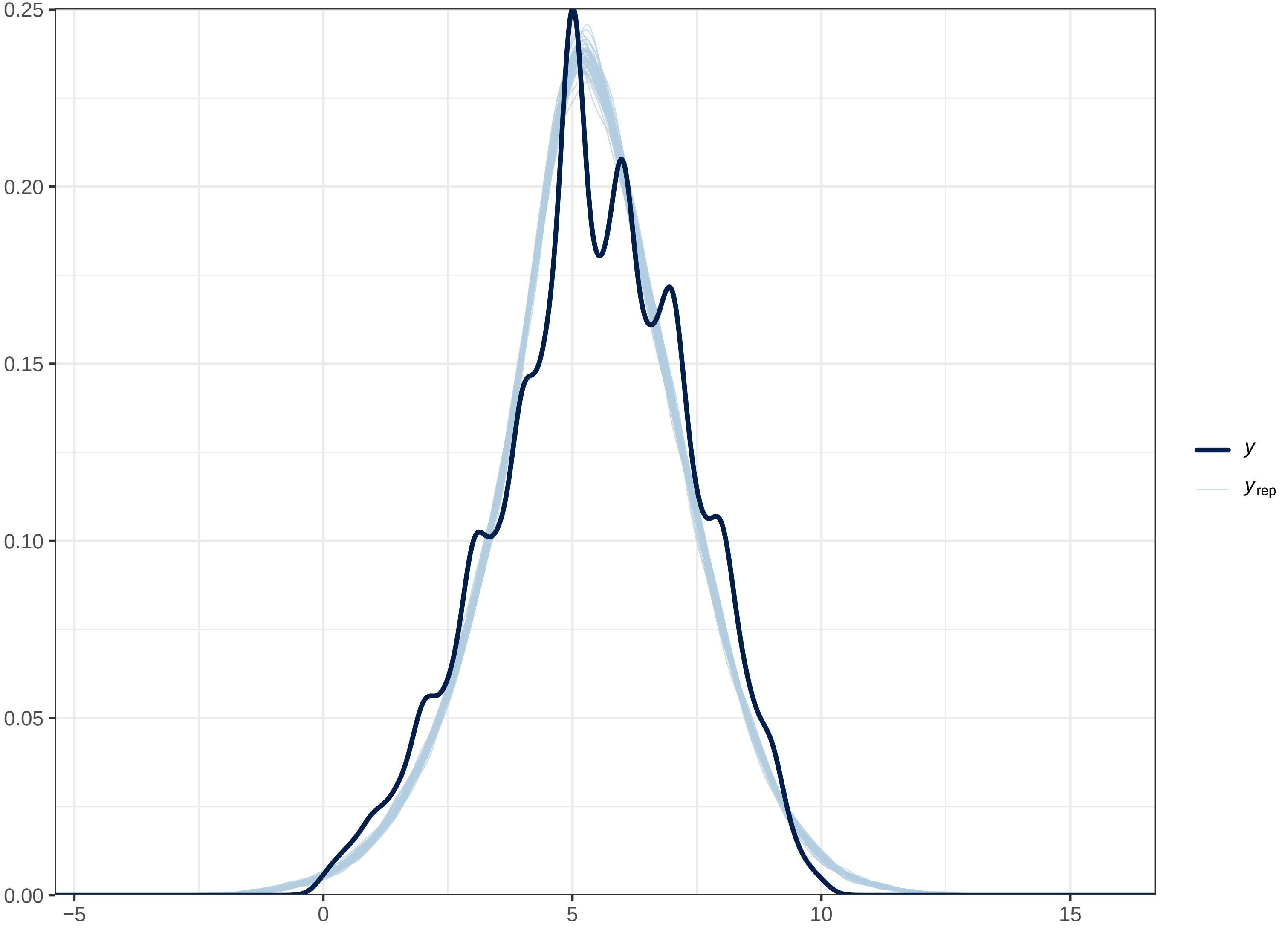

Supplement: Supplementary file 1 [file DataSheet1.zip › Supplementary_Files_3/brms_files/posterior_predictive_checks.pdf]
