# Supplementary figures and images for: A multivariate Bayesian modeling strategy coupled with QTL analysis reveals genetic loci linking important sensory wine quality attributes with their corresponding wine aroma compounds (part 1 of 3)
Source: Front Plant Sci. 2026 Jun 26;17:1851889. doi: 10.3389/fpls.2026.1851889 (PMC13350352; doi:10.3389/fpls.2026.1851889)

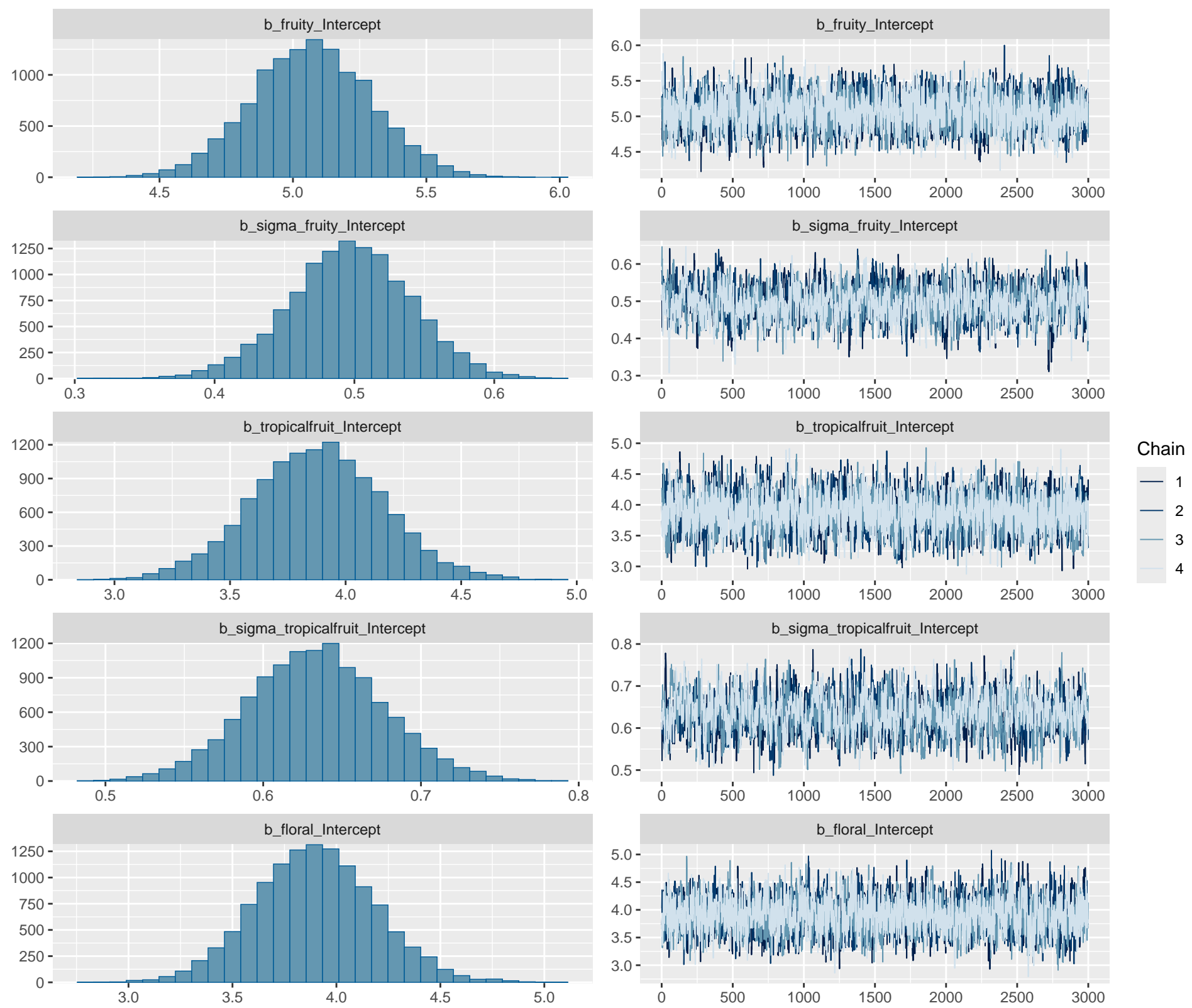

Supplement: Supplementary file 1 [file DataSheet1.zip › Supplementary_Files_3/brms_files/trace_plot_1.pdf]

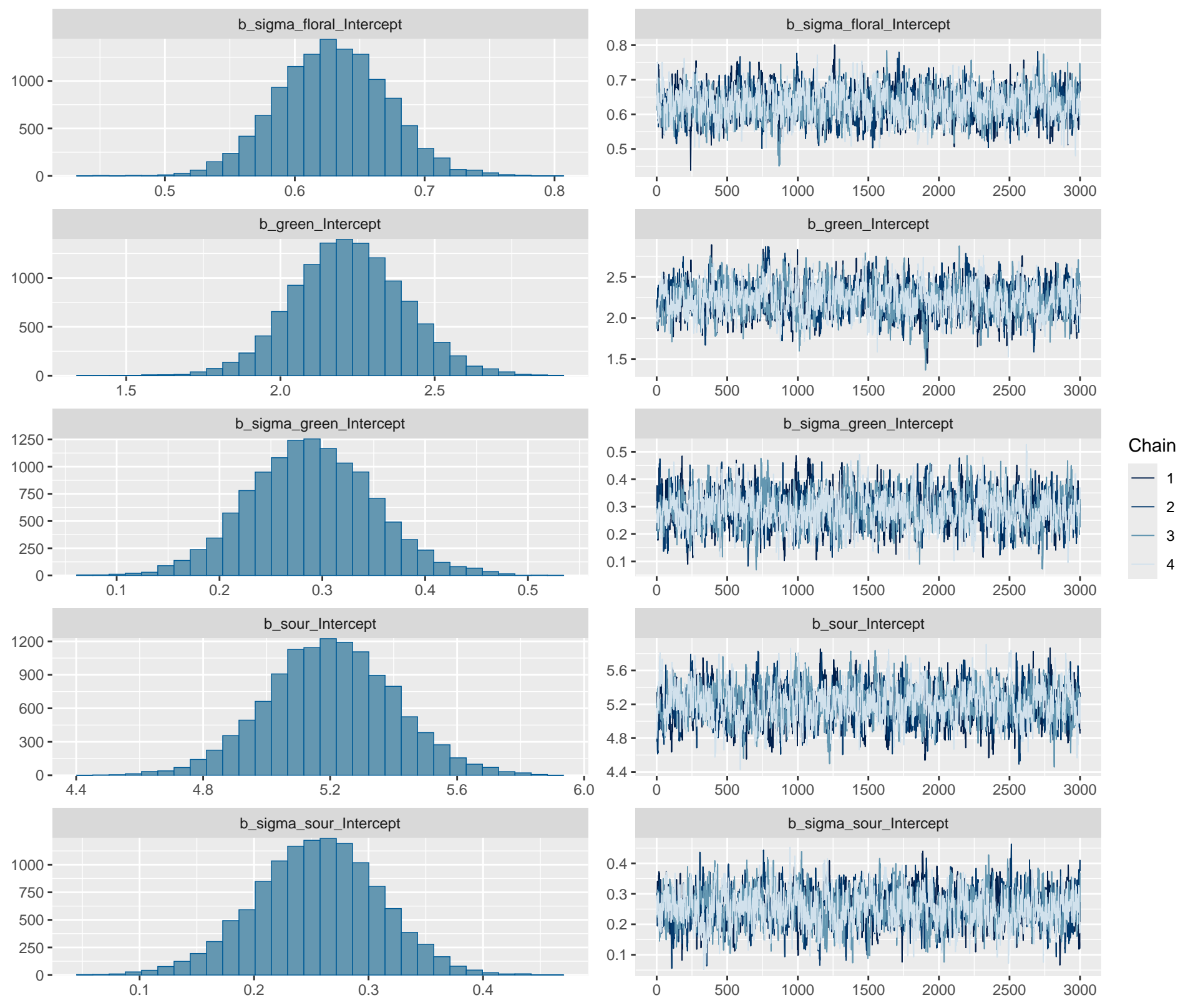

Supplement: Supplementary file 1 [file DataSheet1.zip › Supplementary_Files_3/brms_files/trace_plot_2.pdf]

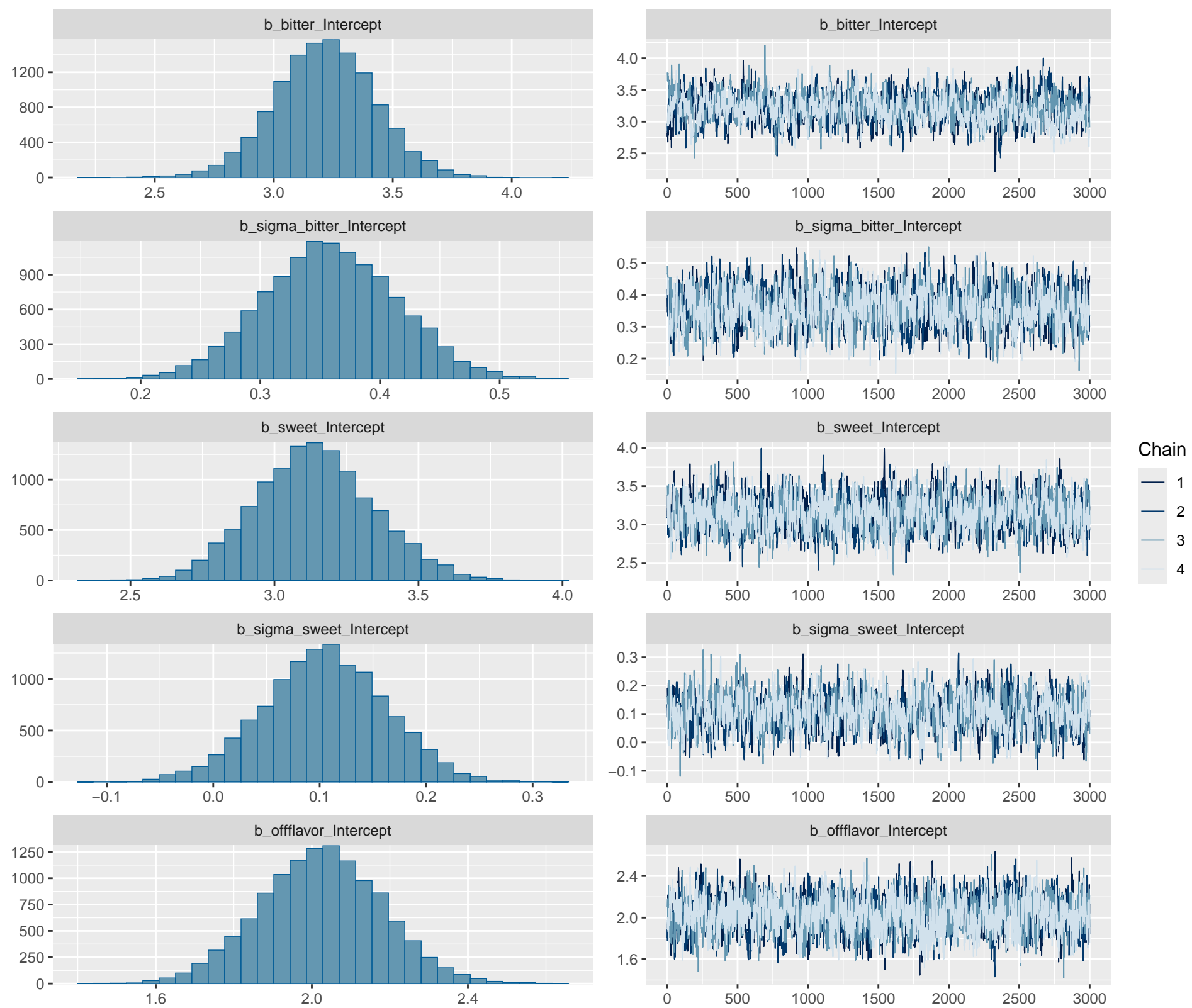

Supplement: Supplementary file 1 [file DataSheet1.zip › Supplementary_Files_3/brms_files/trace_plot_3.pdf]

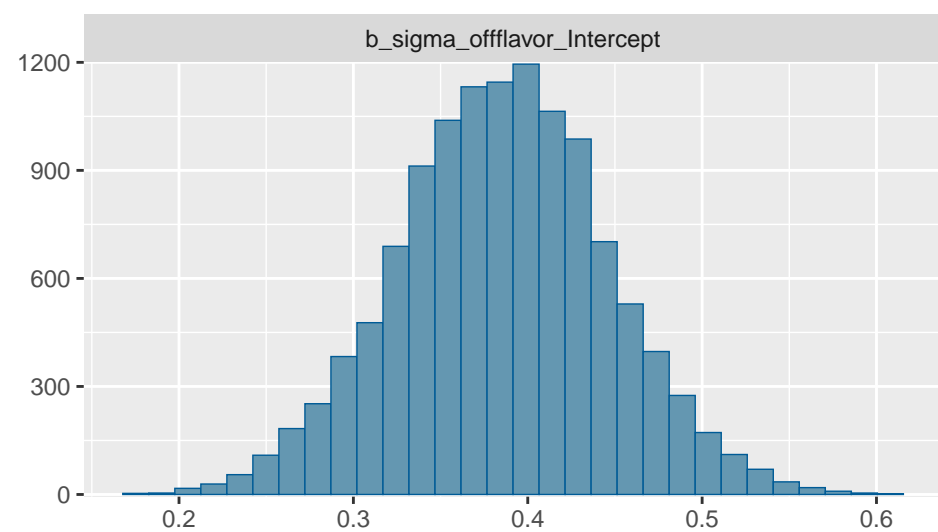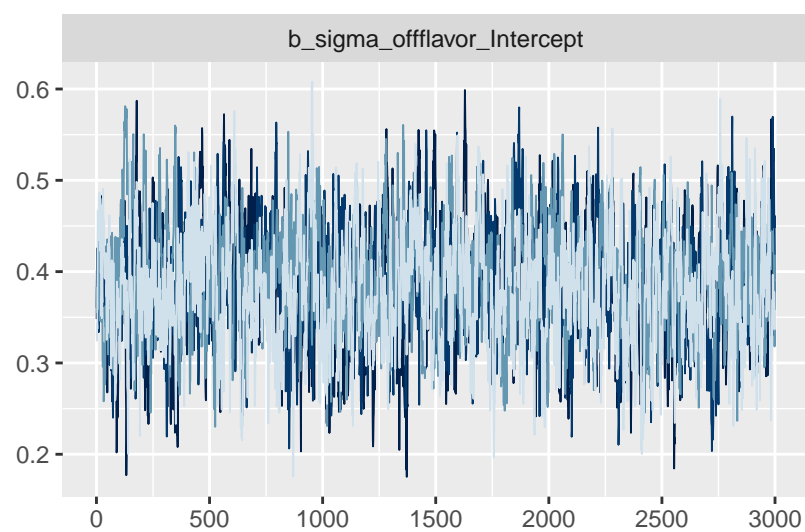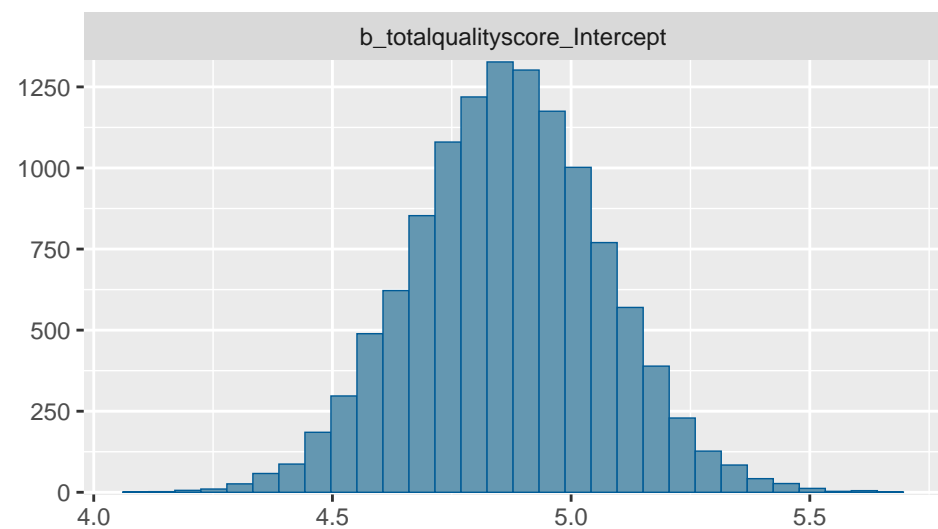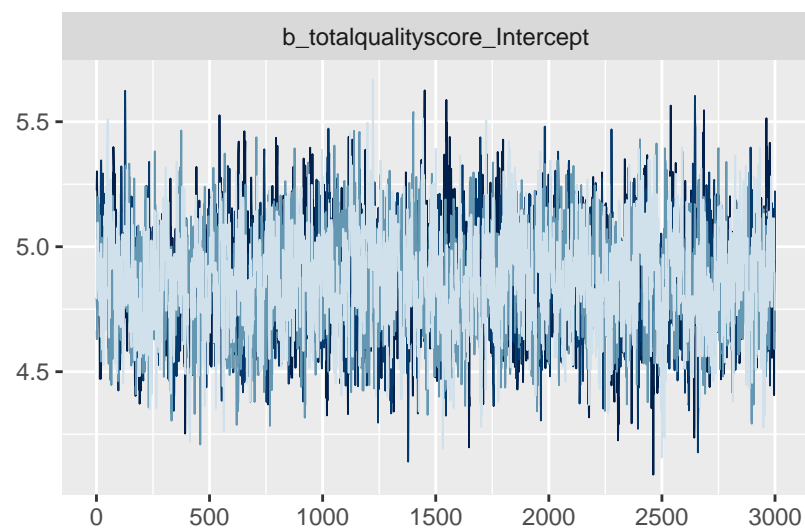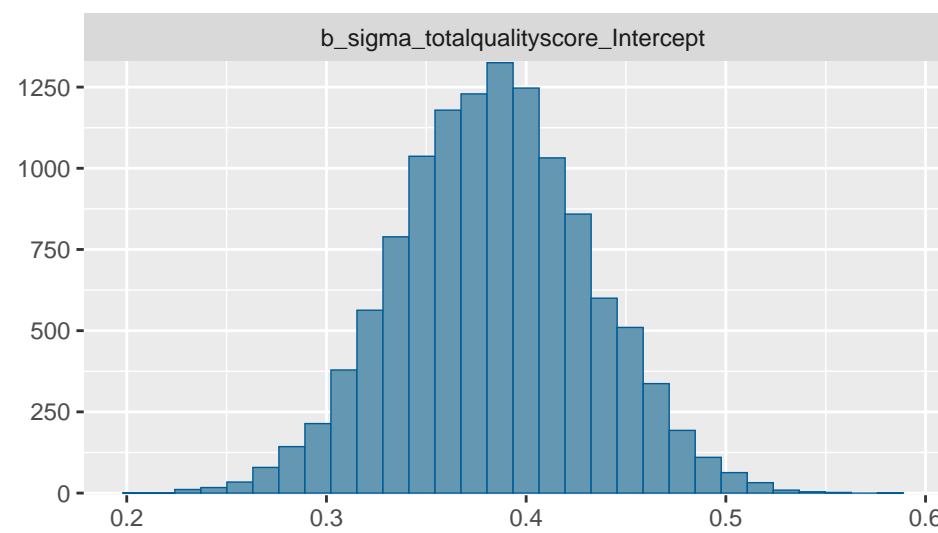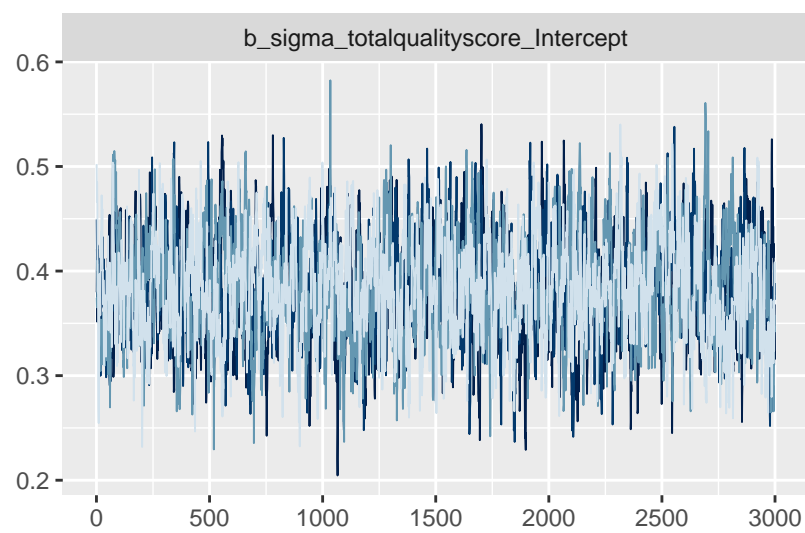

Chain

1

2

3

4

Supplement: Supplementary file 1 [file DataSheet1.zip › Supplementary_Files_3/brms_files/trace_plot_4.pdf]

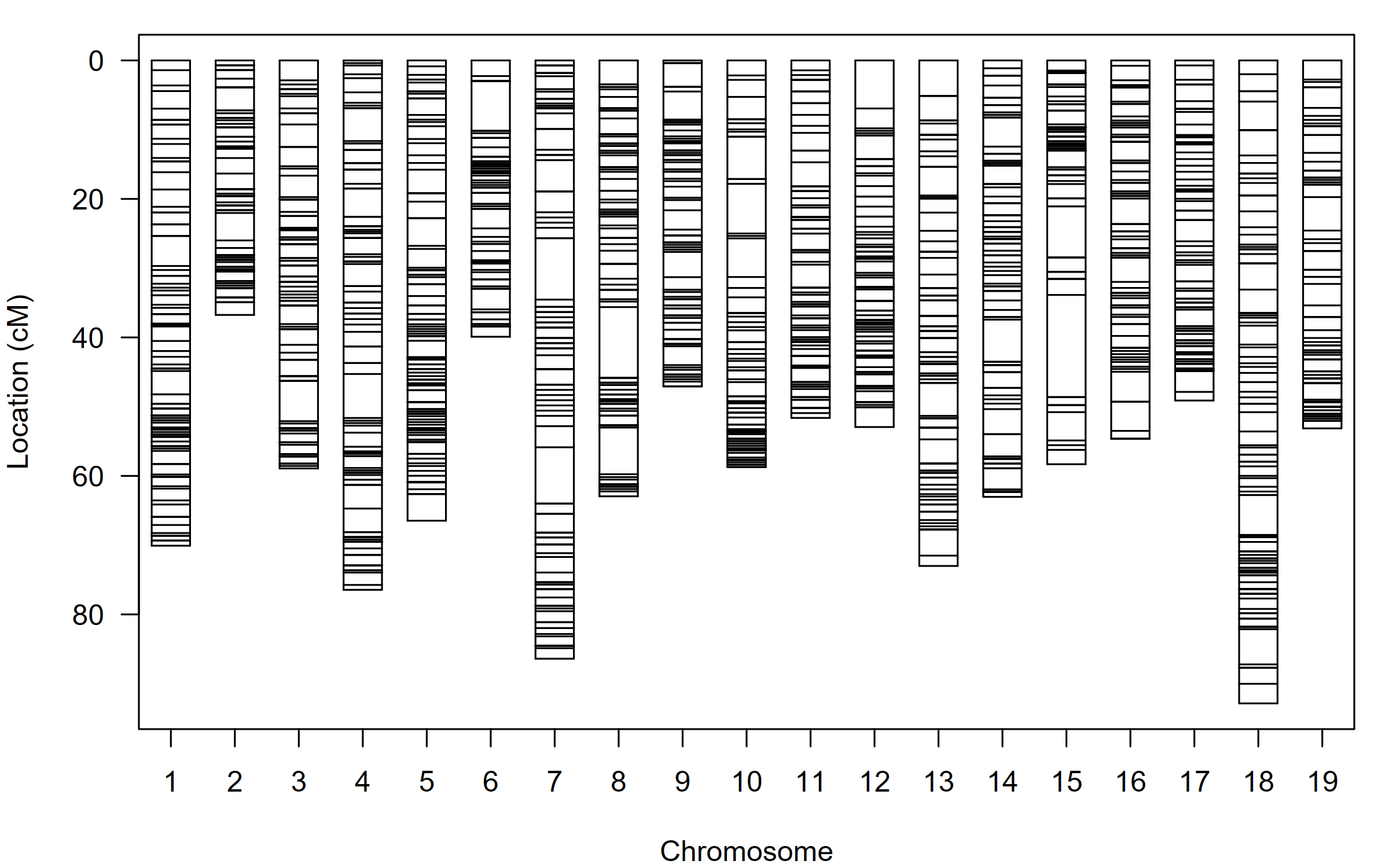

Supplement: Supplementary file 2 [file DataSheet2.zip › Supplementary_Files_4/genetic_map_coverage.tiff]

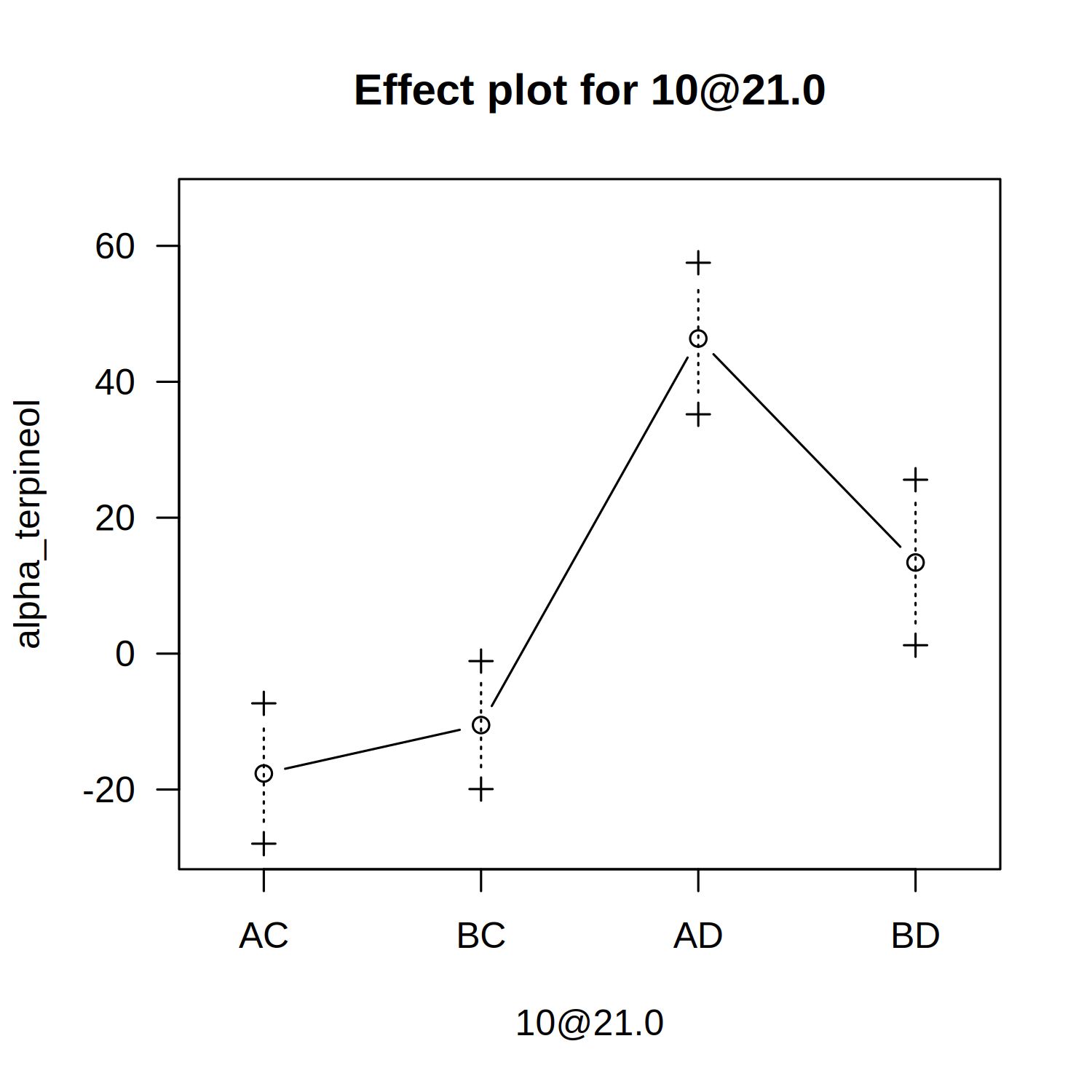

Supplement: Supplementary file 2 [file DataSheet2.zip › Supplementary_Files_4/QTL_analysis/alpha_terpineol/alpha_terpineol_eff_chr10.jpg]

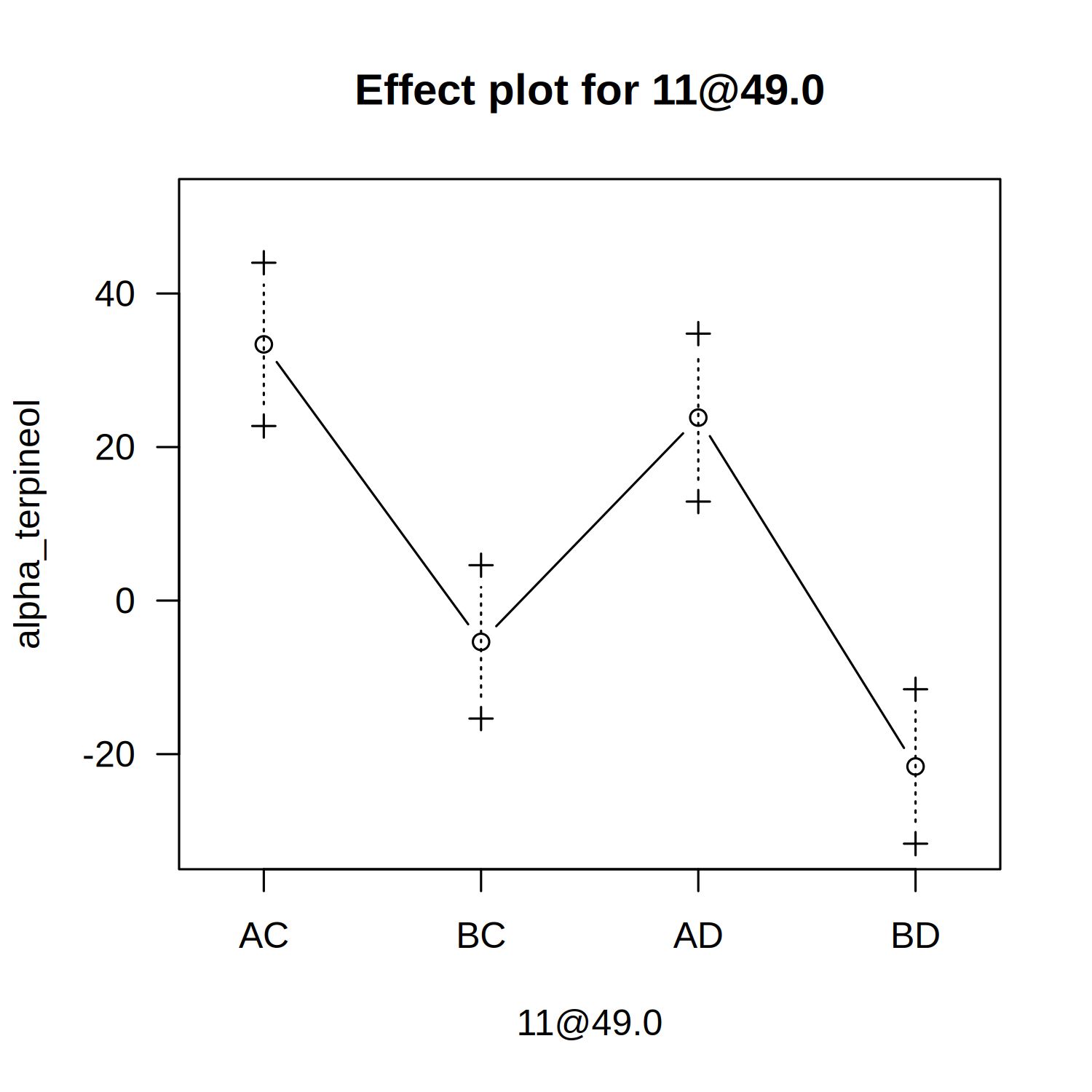

Supplement: Supplementary file 2 [file DataSheet2.zip › Supplementary_Files_4/QTL_analysis/alpha_terpineol/alpha_terpineol_eff_chr11.jpg]

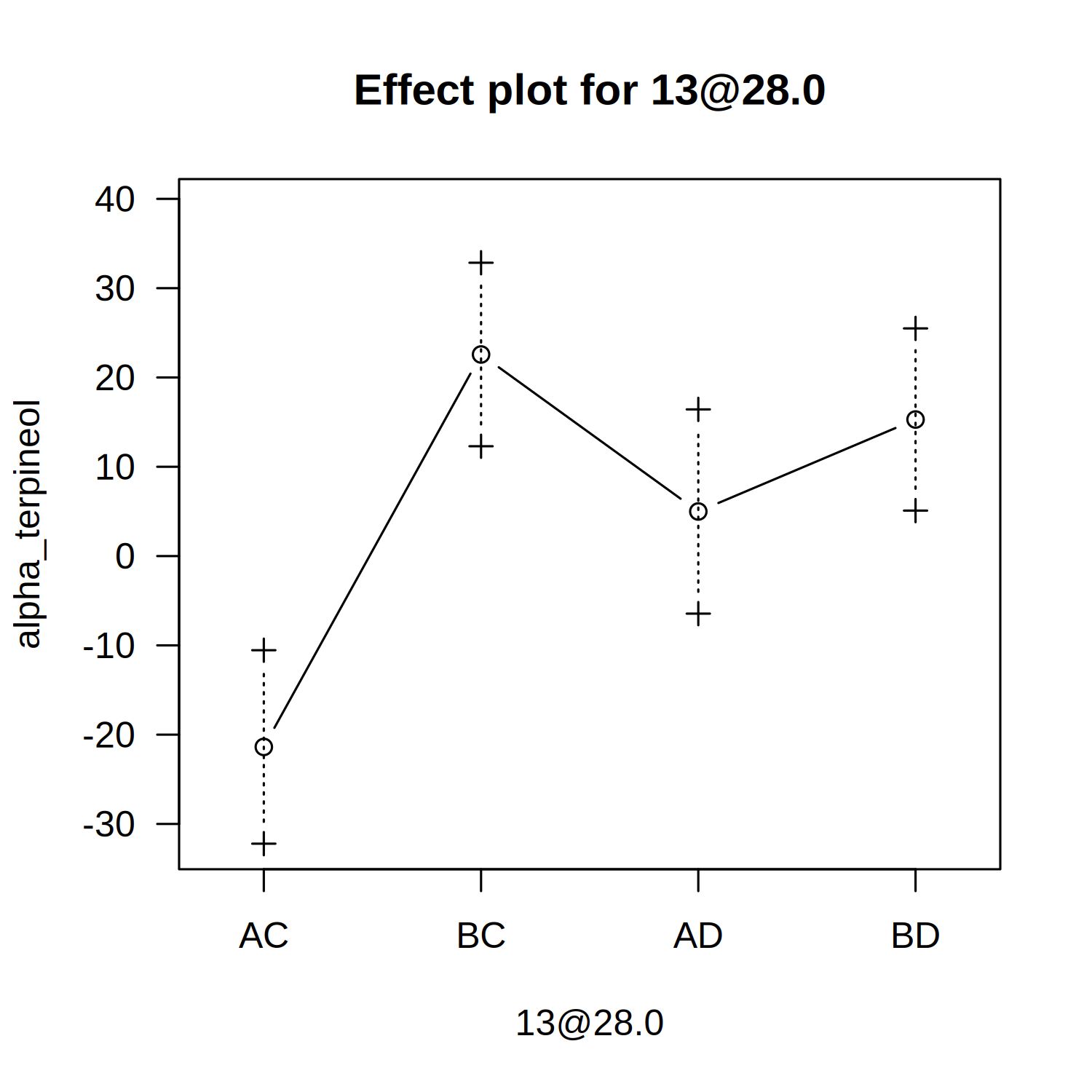

Supplement: Supplementary file 2 [file DataSheet2.zip › Supplementary_Files_4/QTL_analysis/alpha_terpineol/alpha_terpineol_eff_chr13.jpg]

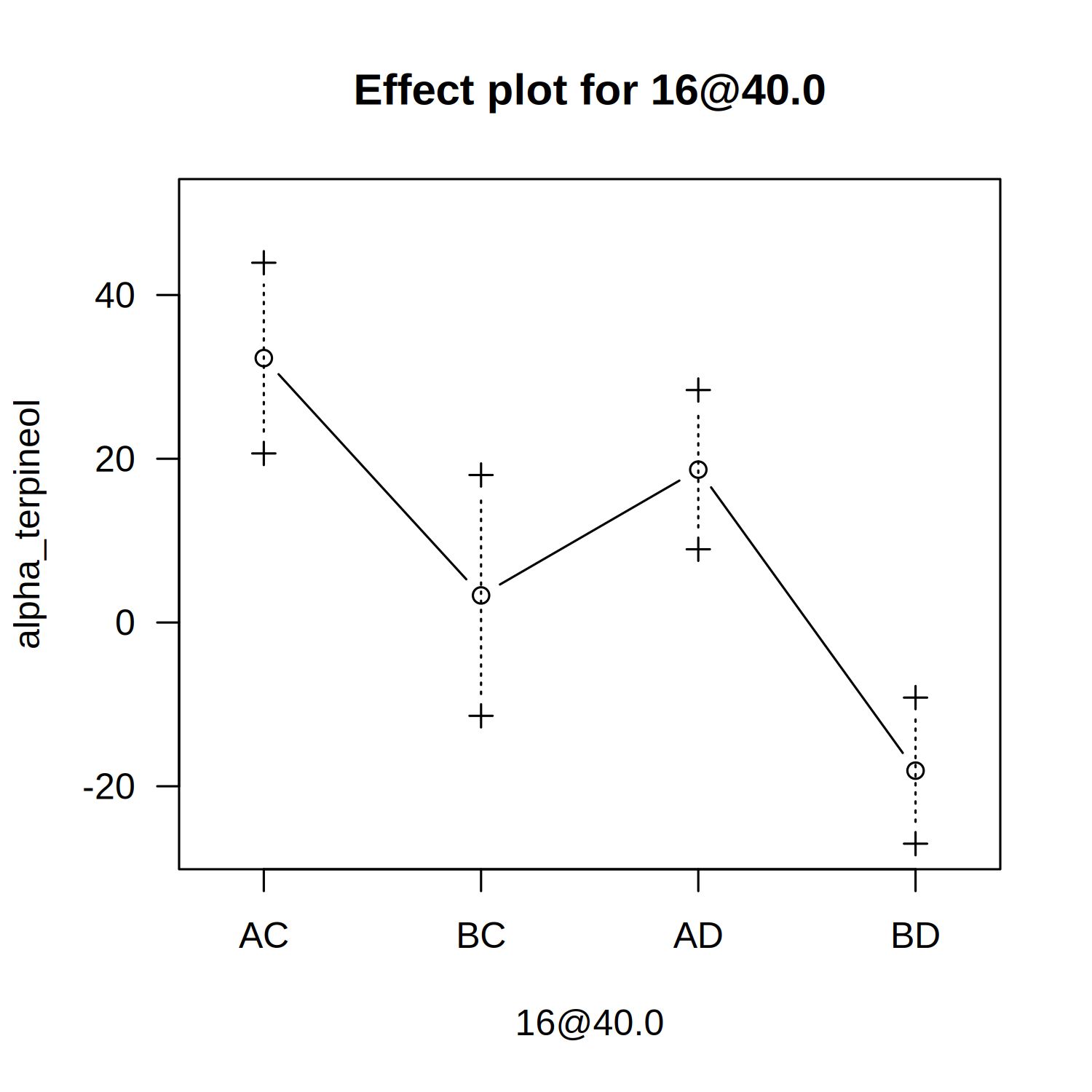

Supplement: Supplementary file 2 [file DataSheet2.zip › Supplementary_Files_4/QTL_analysis/alpha_terpineol/alpha_terpineol_eff_chr16.jpg]

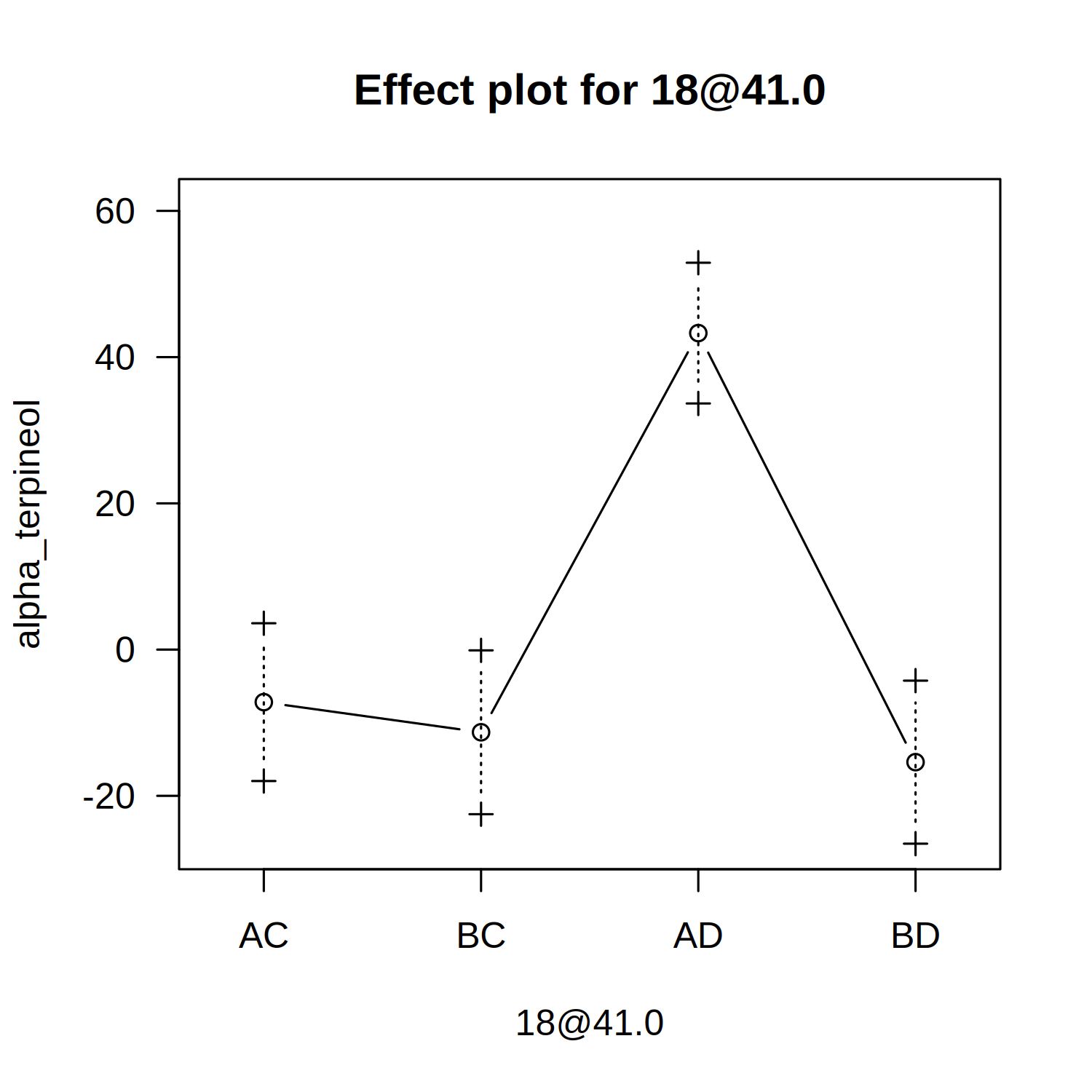

Supplement: Supplementary file 2 [file DataSheet2.zip › Supplementary_Files_4/QTL_analysis/alpha_terpineol/alpha_terpineol_eff_chr18.jpg]

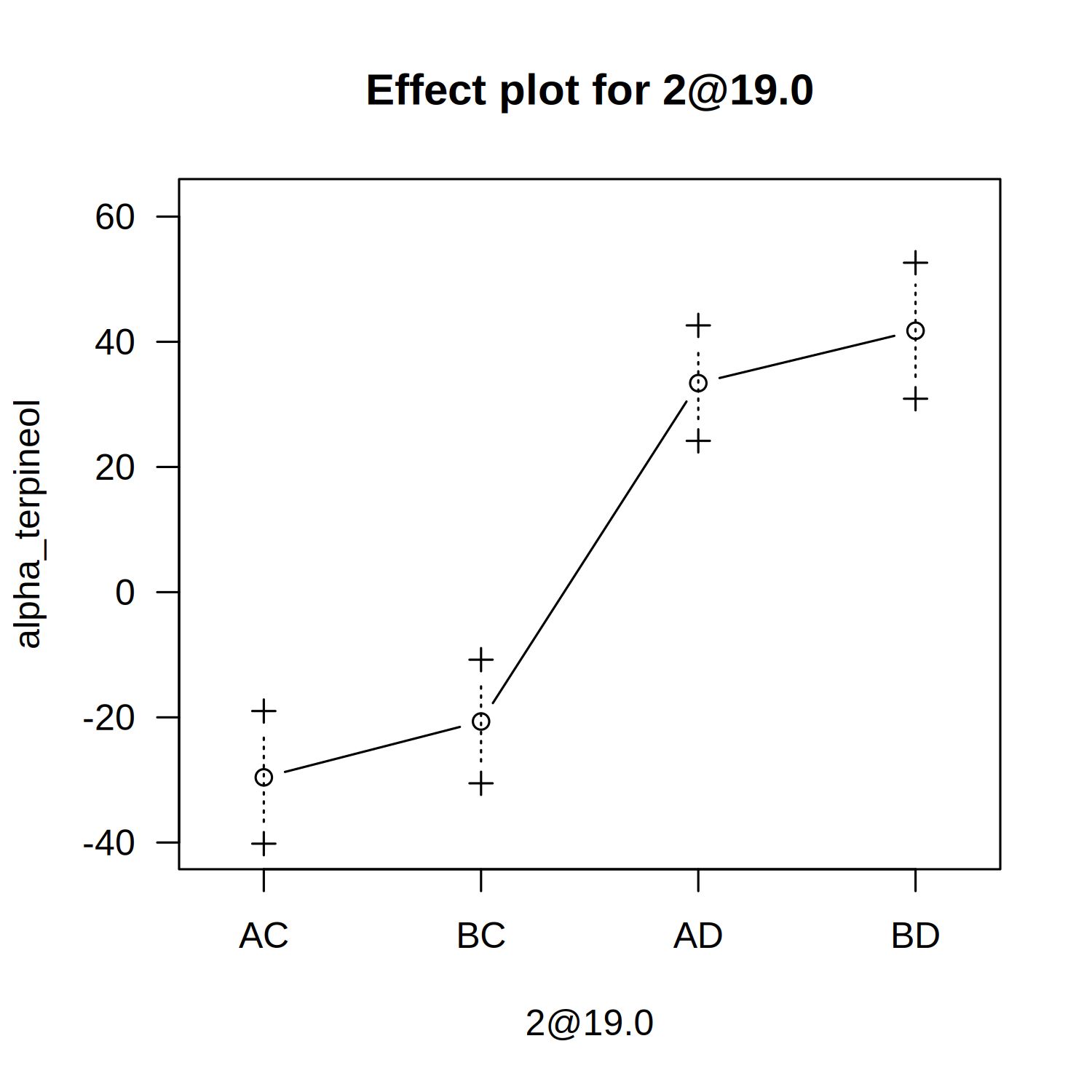

Supplement: Supplementary file 2 [file DataSheet2.zip › Supplementary_Files_4/QTL_analysis/alpha_terpineol/alpha_terpineol_eff_chr2.jpg]

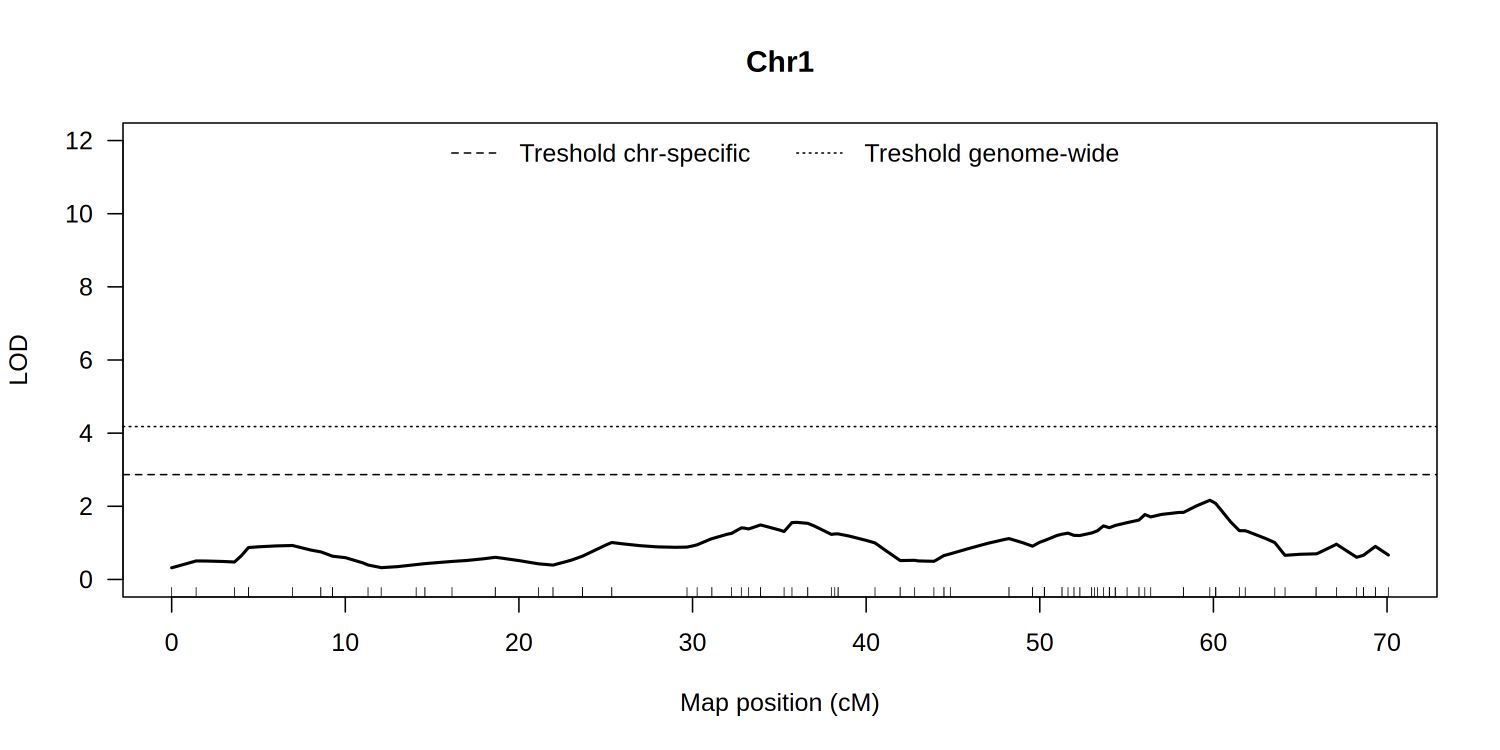

Supplement: Supplementary file 2 [file DataSheet2.zip › Supplementary_Files_4/QTL_analysis/alpha_terpineol/alpha_terpineol_LODplot_chr1.jpg]

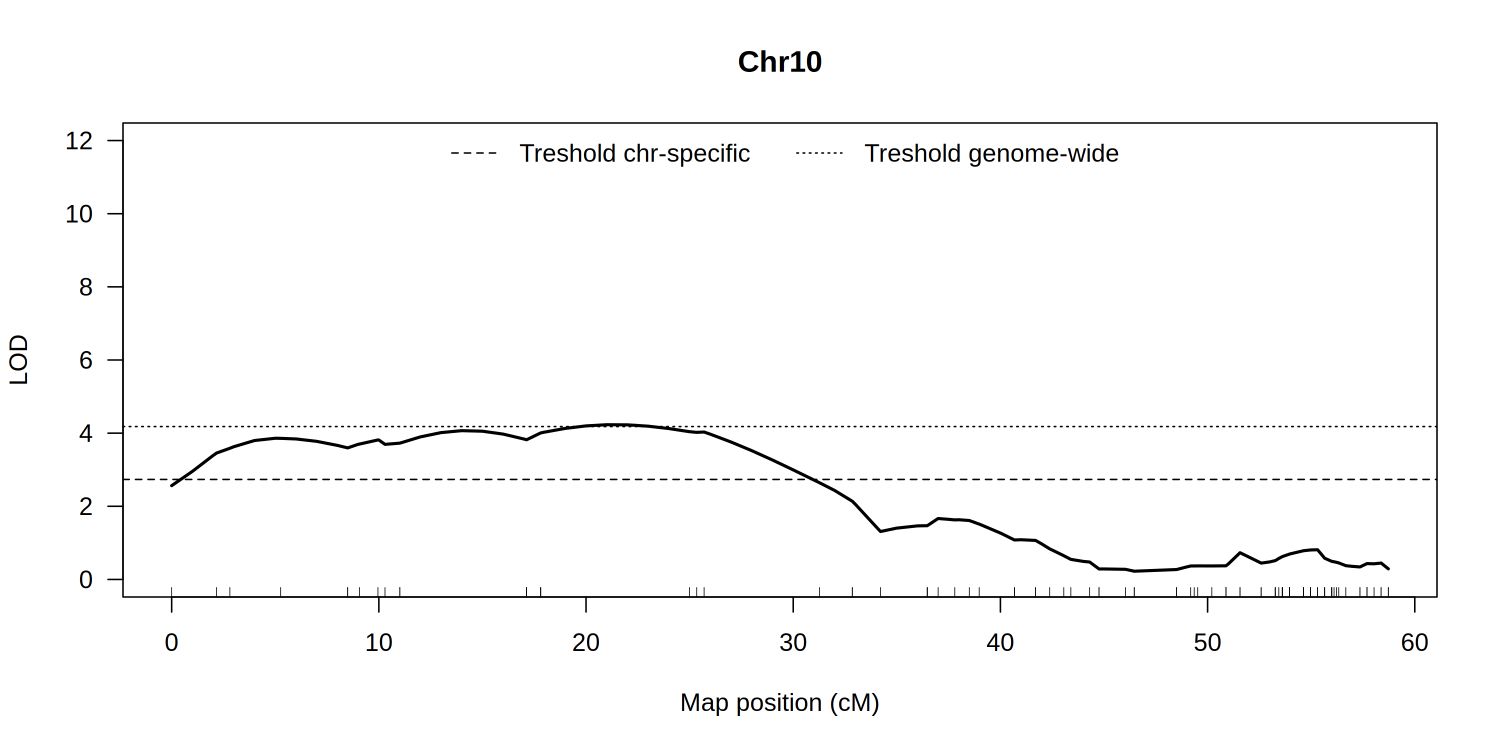

Supplement: Supplementary file 2 [file DataSheet2.zip › Supplementary_Files_4/QTL_analysis/alpha_terpineol/alpha_terpineol_LODplot_chr10.jpg]

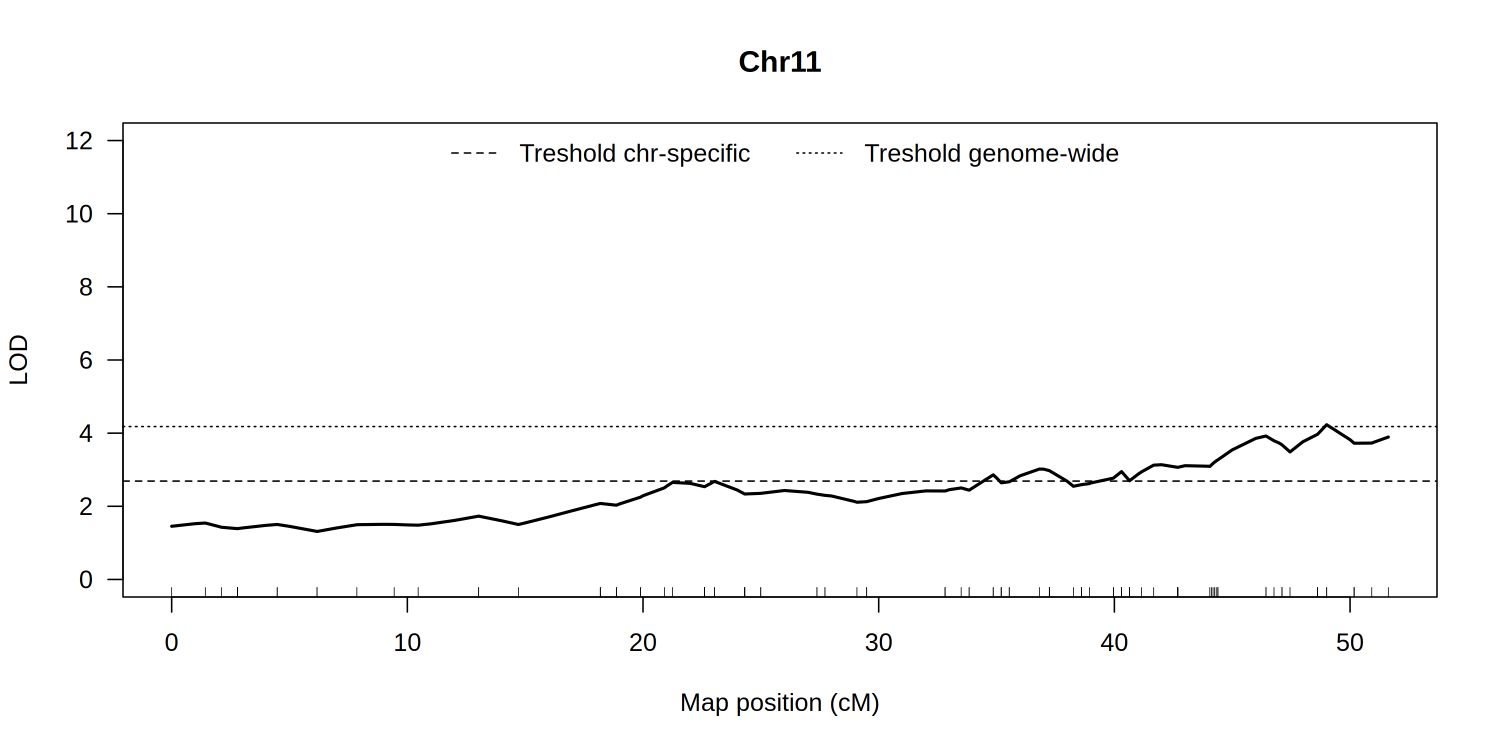

Supplement: Supplementary file 2 [file DataSheet2.zip › Supplementary_Files_4/QTL_analysis/alpha_terpineol/alpha_terpineol_LODplot_chr11.jpg]

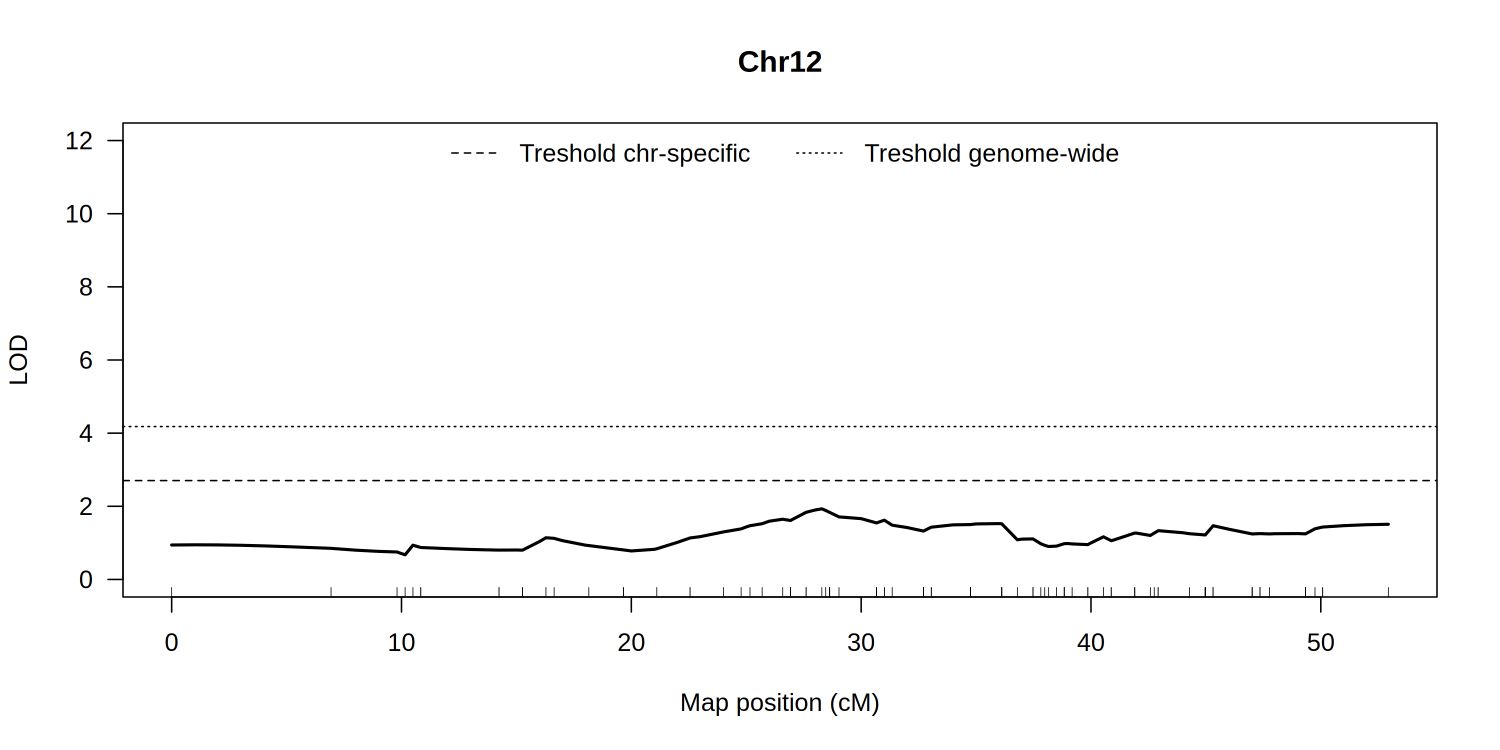

Supplement: Supplementary file 2 [file DataSheet2.zip › Supplementary_Files_4/QTL_analysis/alpha_terpineol/alpha_terpineol_LODplot_chr12.jpg]

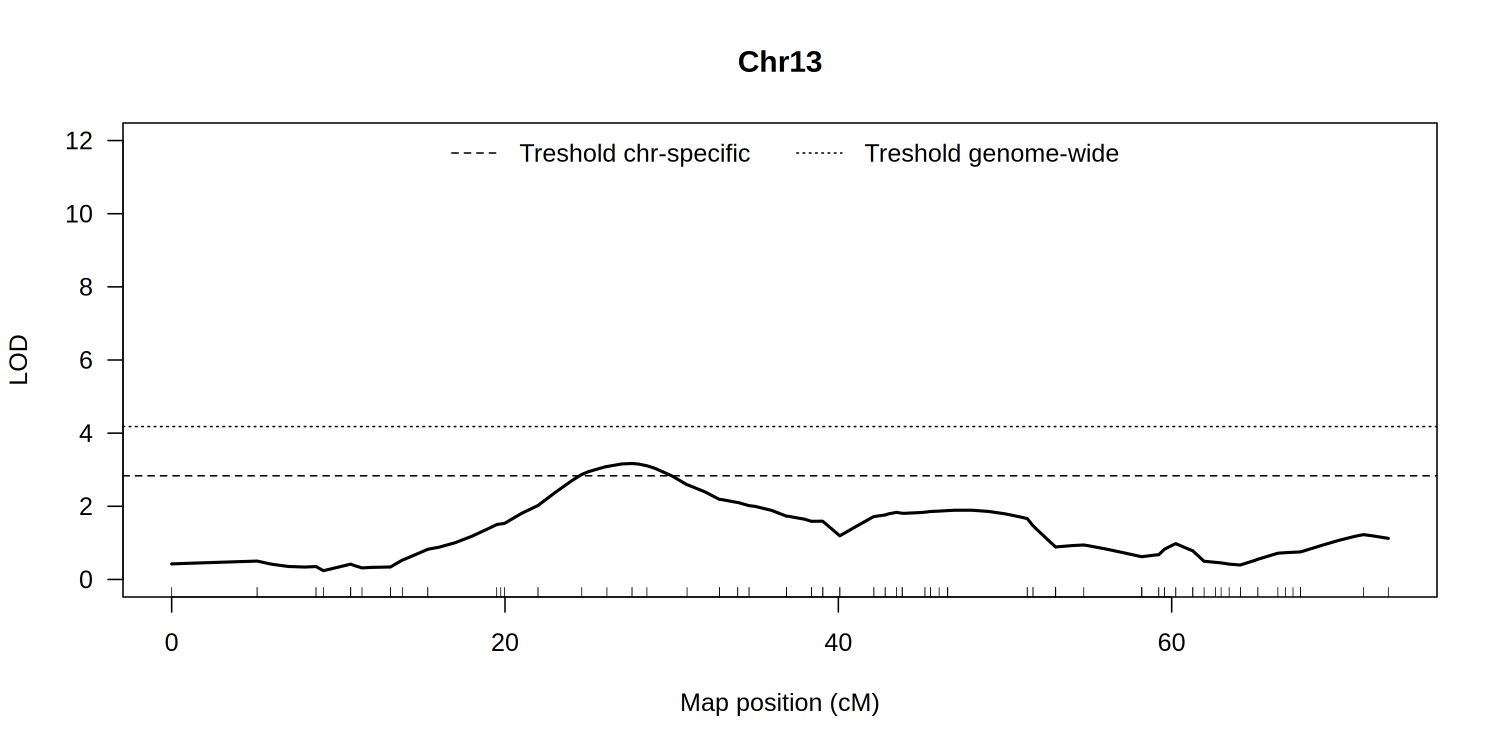

Supplement: Supplementary file 2 [file DataSheet2.zip › Supplementary_Files_4/QTL_analysis/alpha_terpineol/alpha_terpineol_LODplot_chr13.jpg]

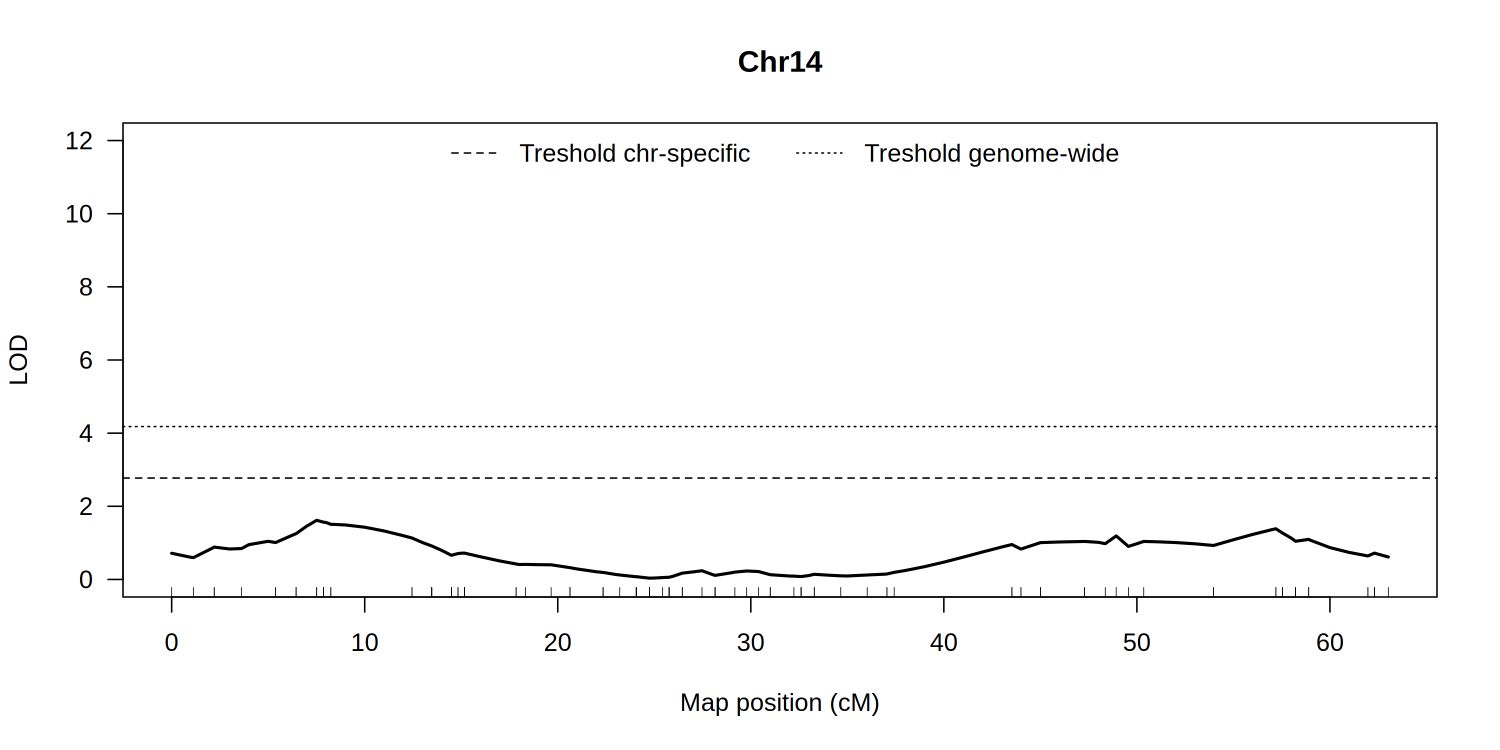

Supplement: Supplementary file 2 [file DataSheet2.zip › Supplementary_Files_4/QTL_analysis/alpha_terpineol/alpha_terpineol_LODplot_chr14.jpg]

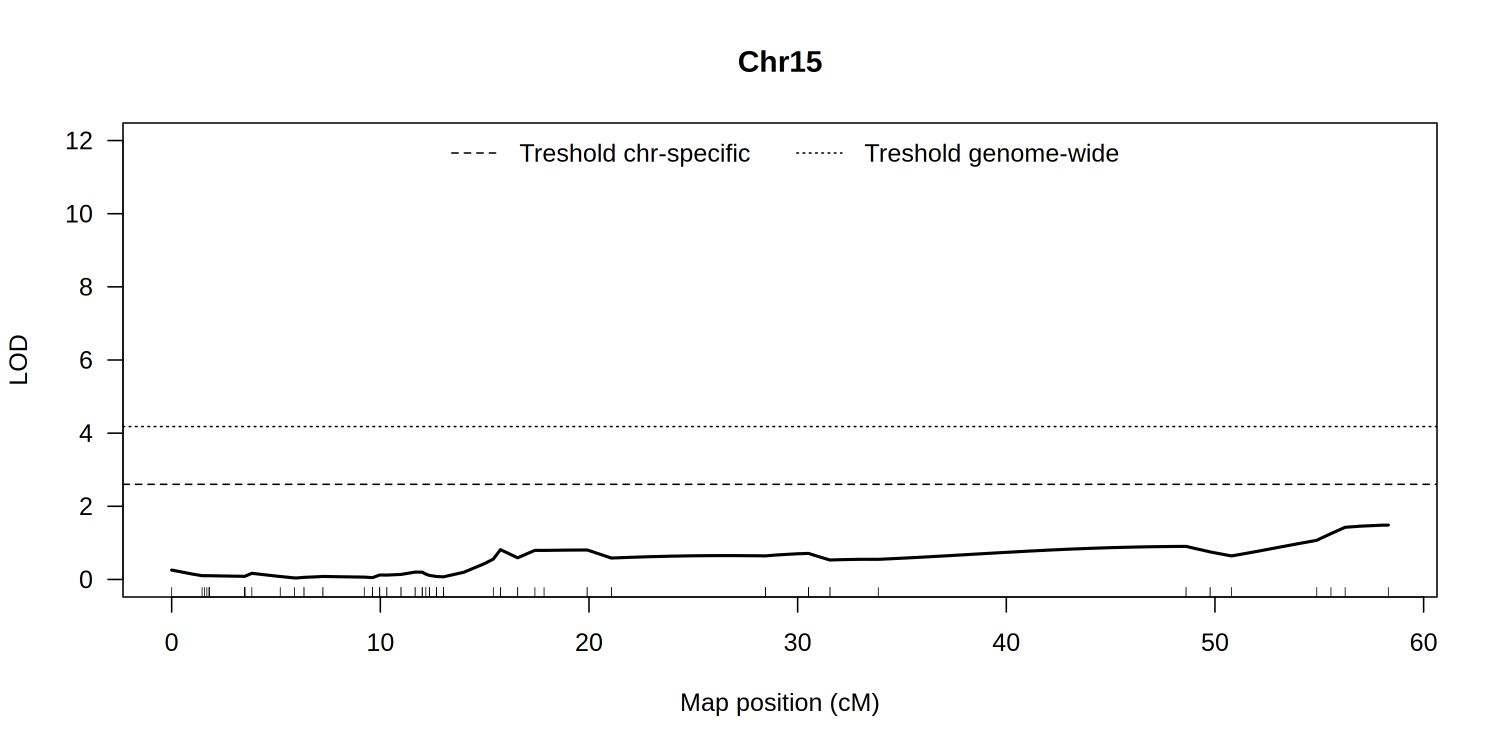

Supplement: Supplementary file 2 [file DataSheet2.zip › Supplementary_Files_4/QTL_analysis/alpha_terpineol/alpha_terpineol_LODplot_chr15.jpg]

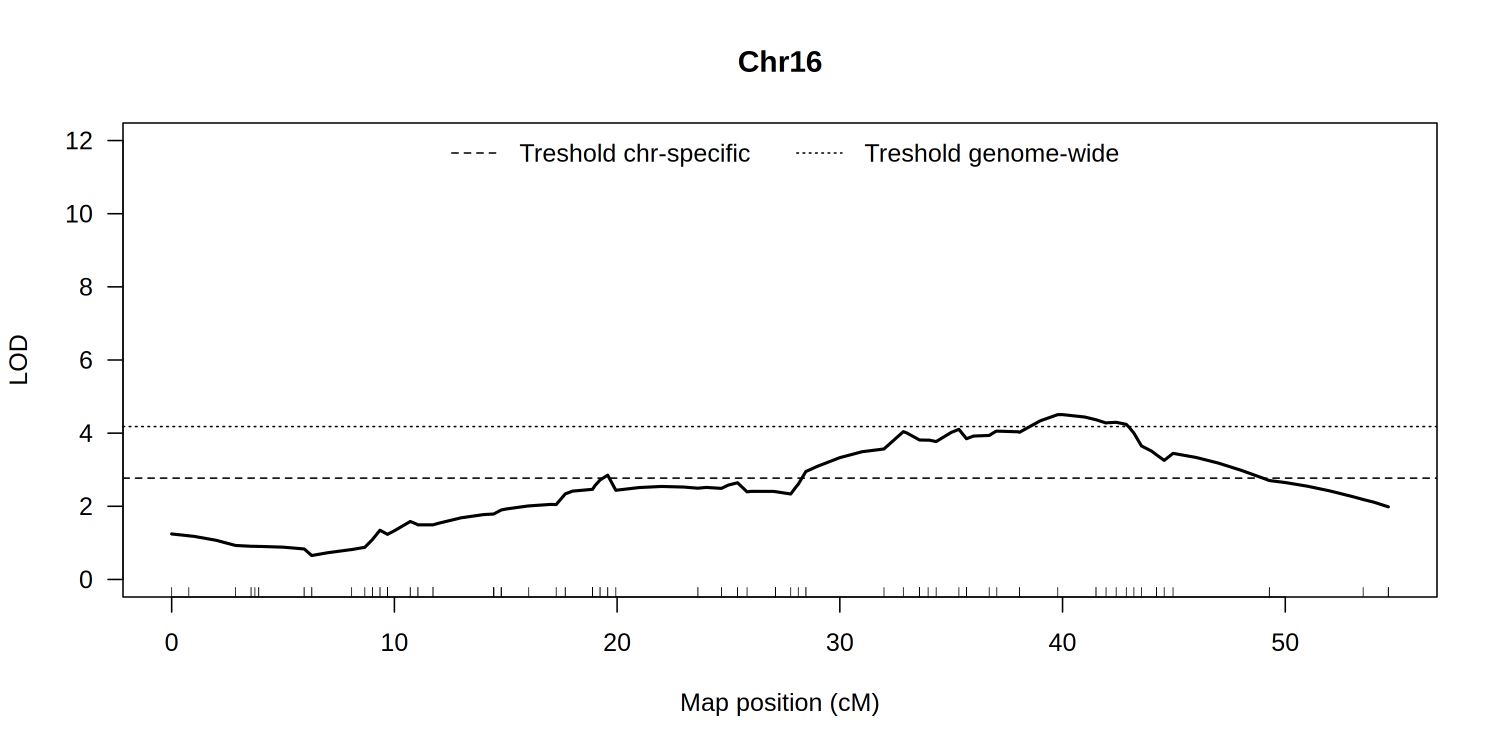

Supplement: Supplementary file 2 [file DataSheet2.zip › Supplementary_Files_4/QTL_analysis/alpha_terpineol/alpha_terpineol_LODplot_chr16.jpg]

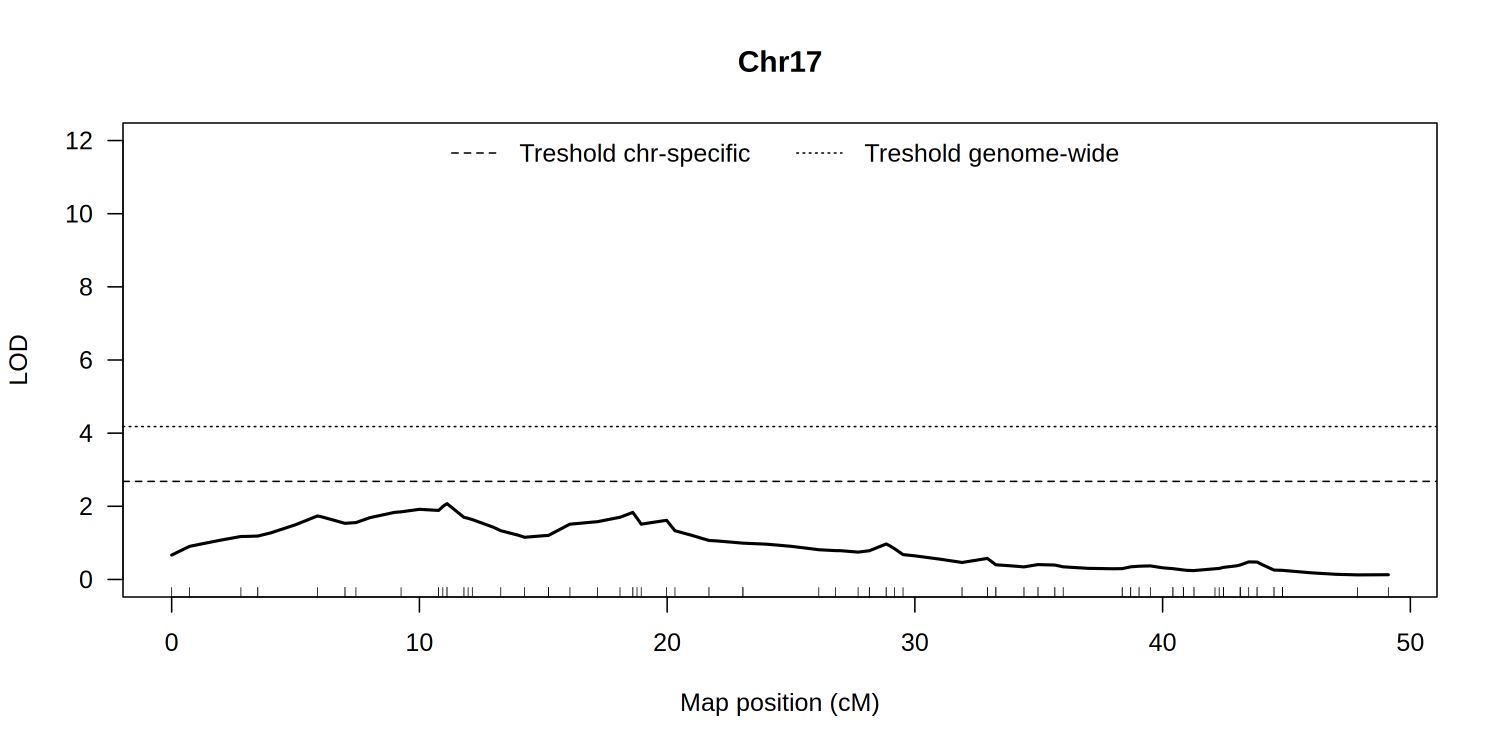

Supplement: Supplementary file 2 [file DataSheet2.zip › Supplementary_Files_4/QTL_analysis/alpha_terpineol/alpha_terpineol_LODplot_chr17.jpg]

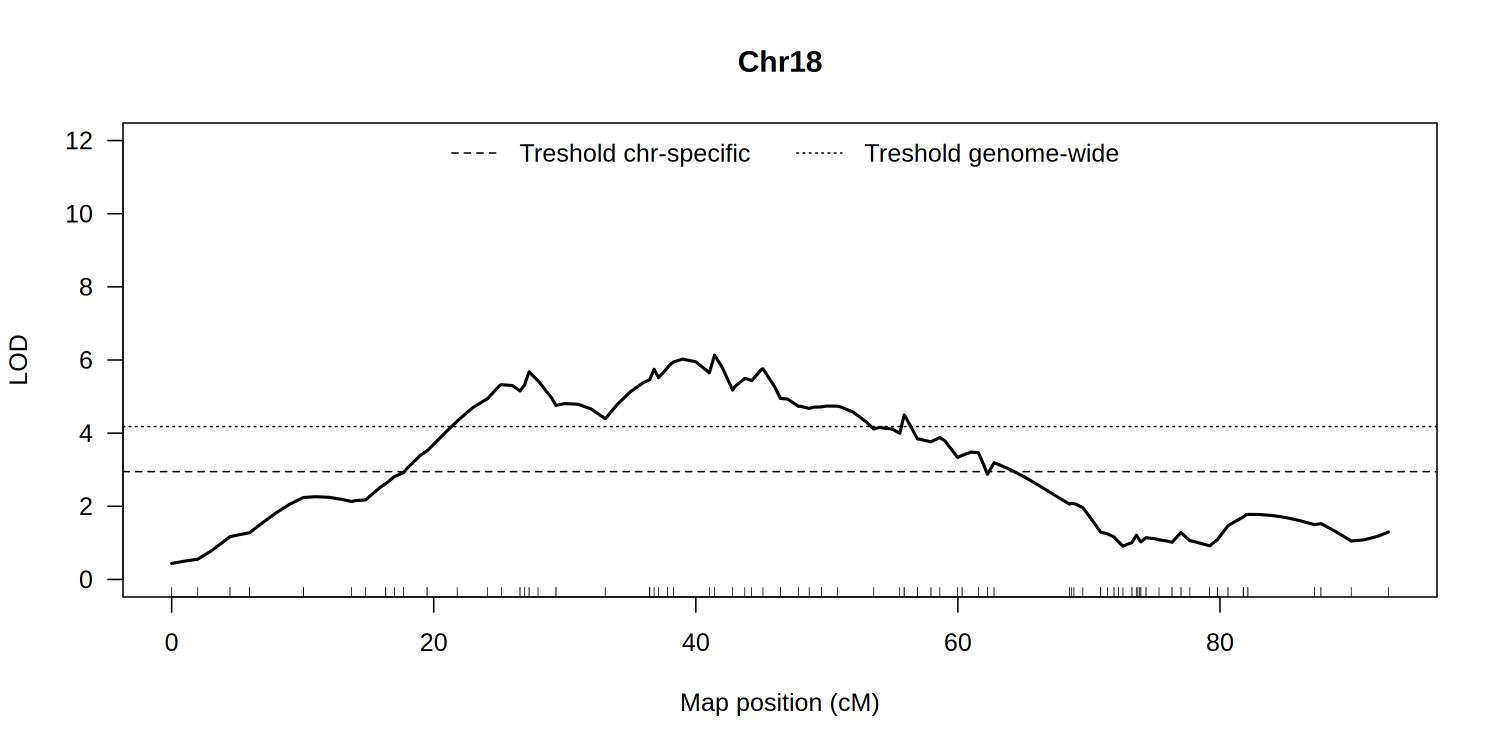

Supplement: Supplementary file 2 [file DataSheet2.zip › Supplementary_Files_4/QTL_analysis/alpha_terpineol/alpha_terpineol_LODplot_chr18.jpg]

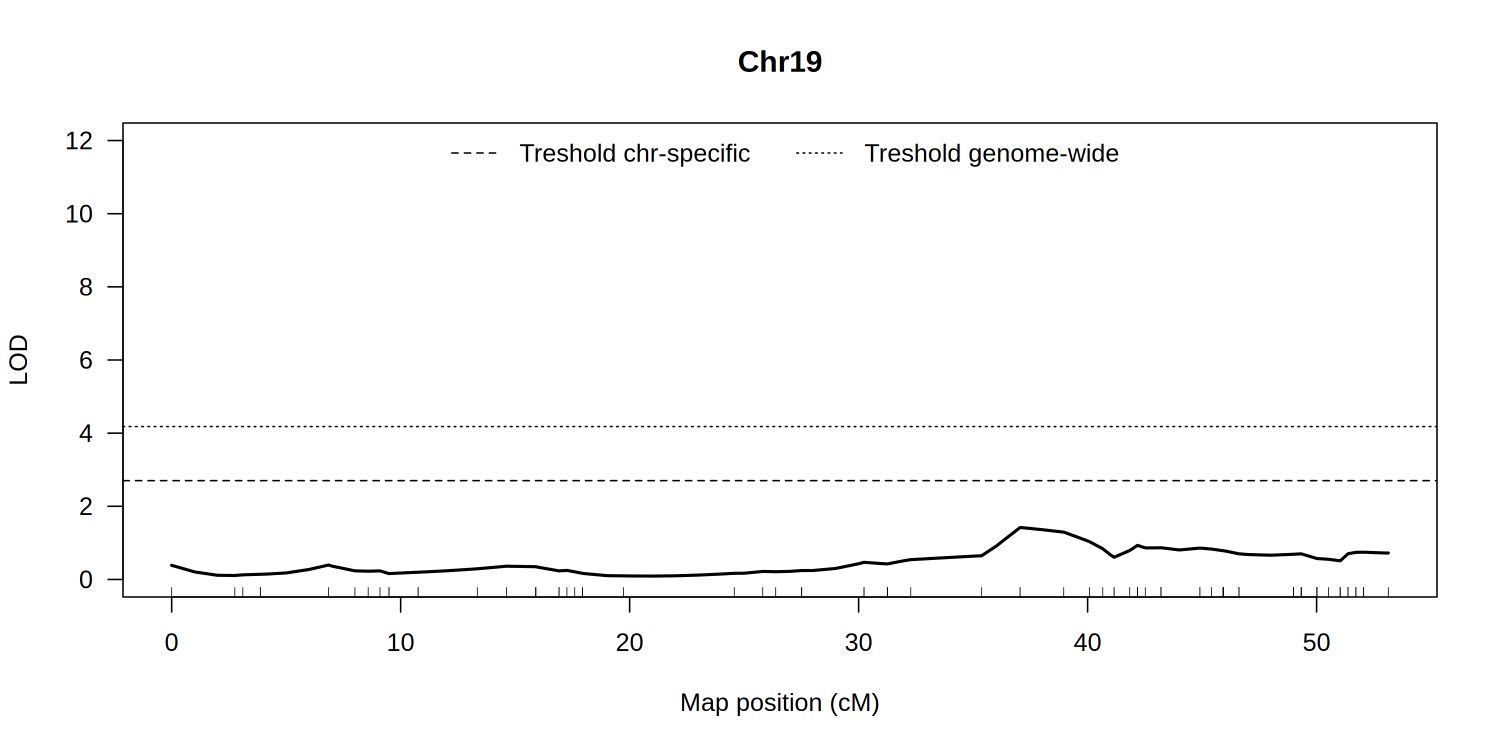

Supplement: Supplementary file 2 [file DataSheet2.zip › Supplementary_Files_4/QTL_analysis/alpha_terpineol/alpha_terpineol_LODplot_chr19.jpg]

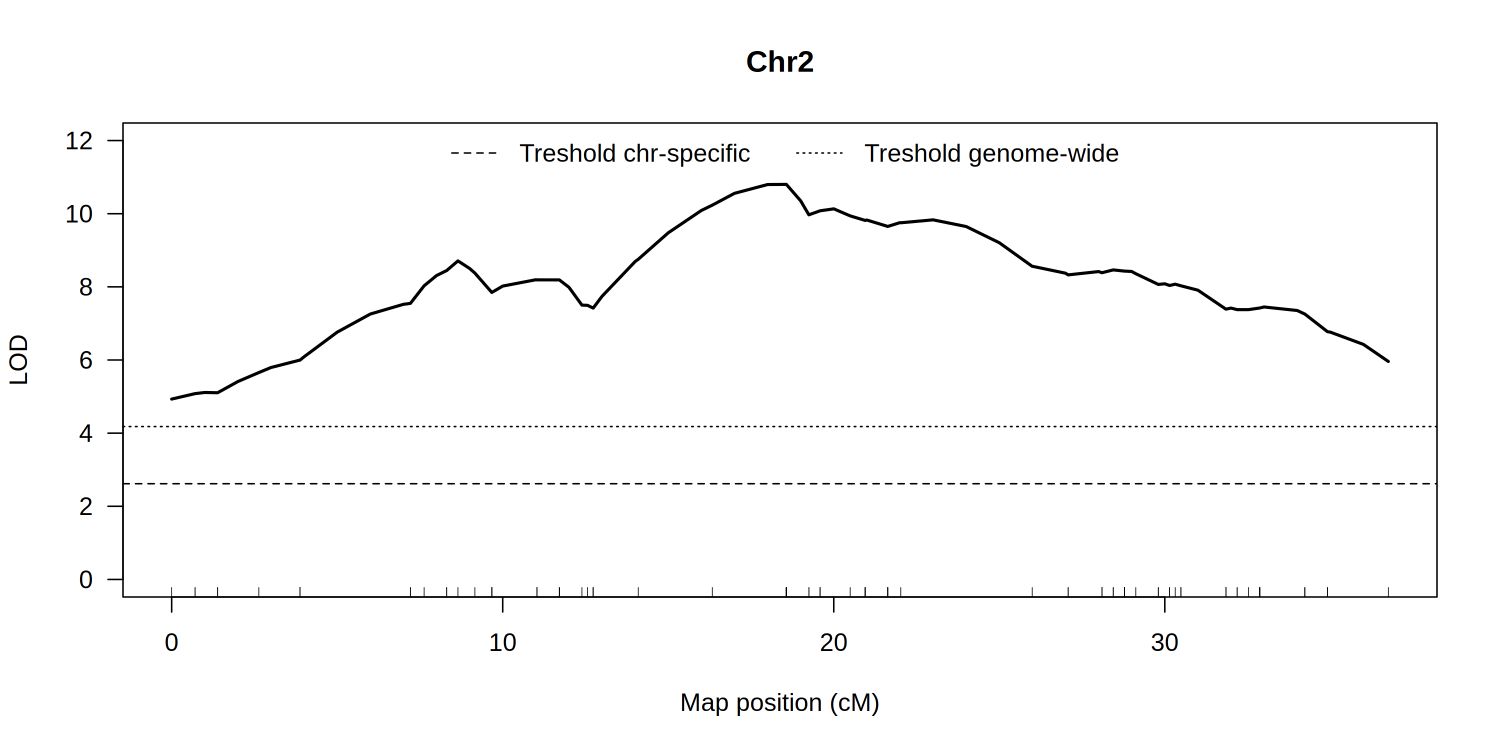

Supplement: Supplementary file 2 [file DataSheet2.zip › Supplementary_Files_4/QTL_analysis/alpha_terpineol/alpha_terpineol_LODplot_chr2.jpg]

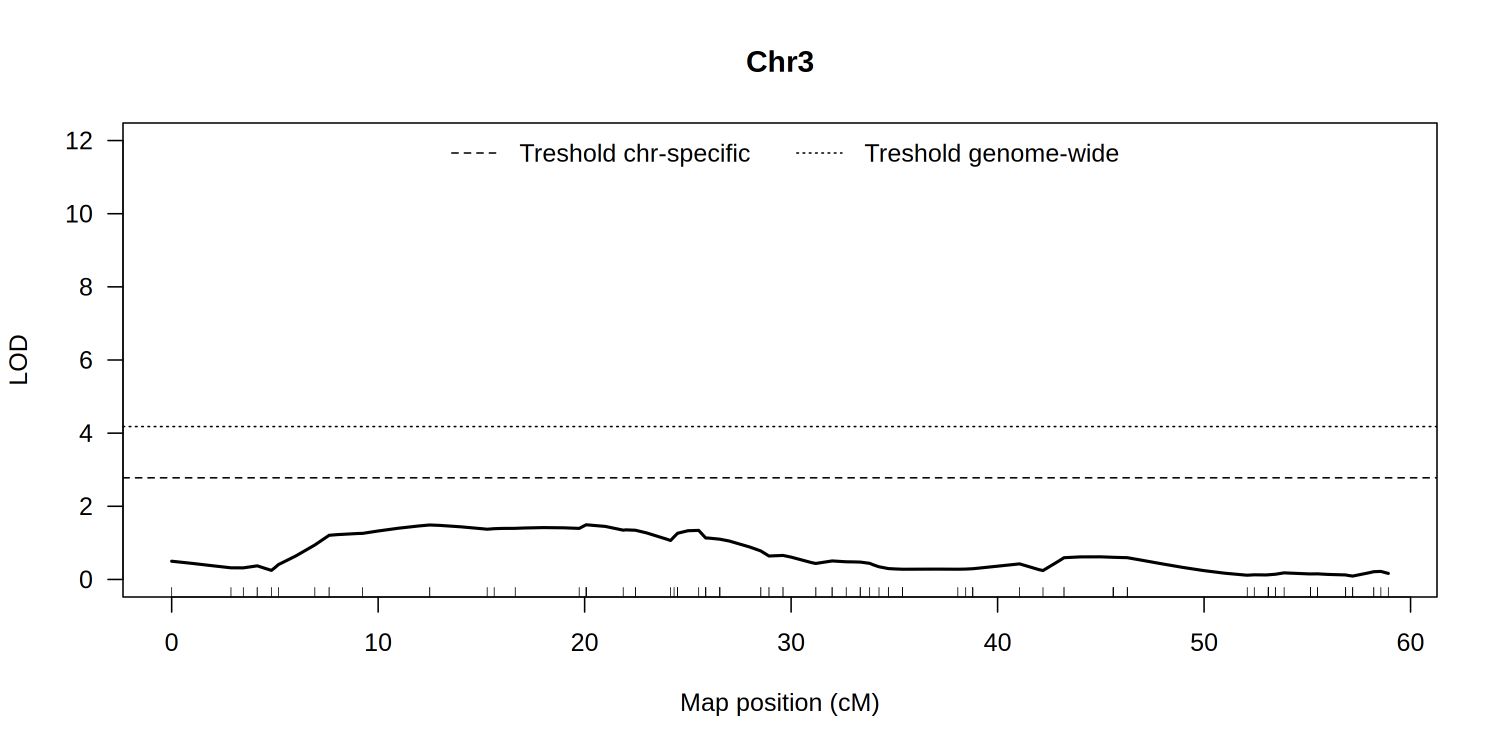

Supplement: Supplementary file 2 [file DataSheet2.zip › Supplementary_Files_4/QTL_analysis/alpha_terpineol/alpha_terpineol_LODplot_chr3.jpg]

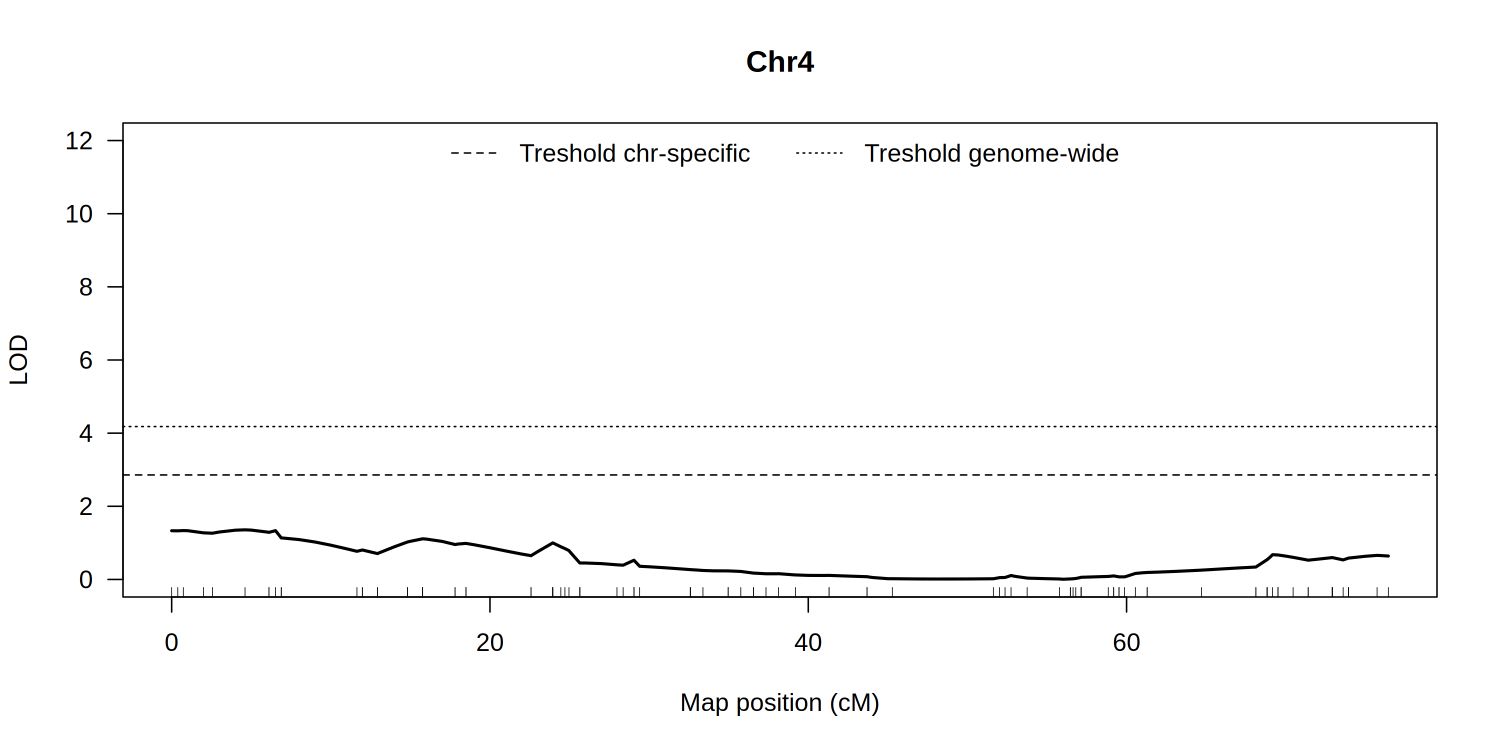

Supplement: Supplementary file 2 [file DataSheet2.zip › Supplementary_Files_4/QTL_analysis/alpha_terpineol/alpha_terpineol_LODplot_chr4.jpg]

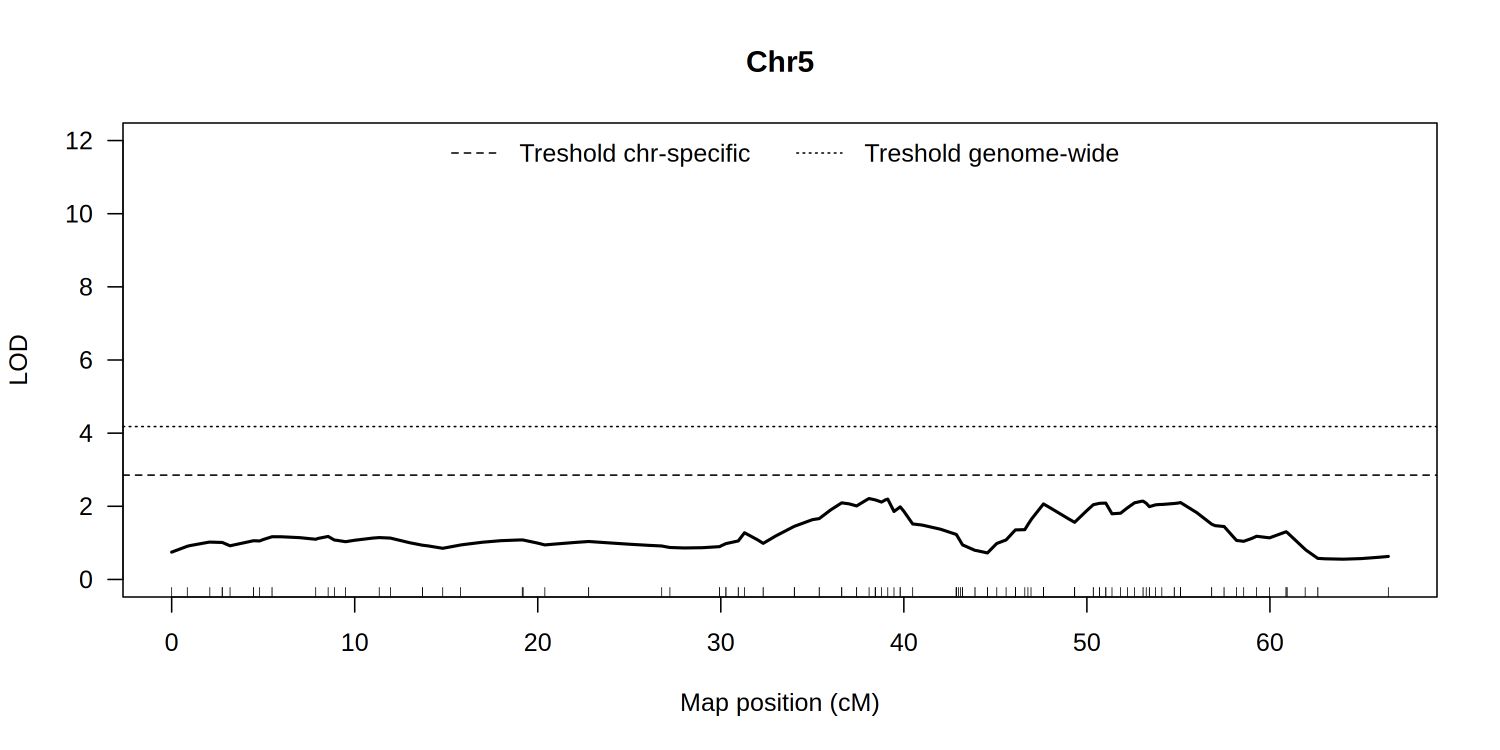

Supplement: Supplementary file 2 [file DataSheet2.zip › Supplementary_Files_4/QTL_analysis/alpha_terpineol/alpha_terpineol_LODplot_chr5.jpg]

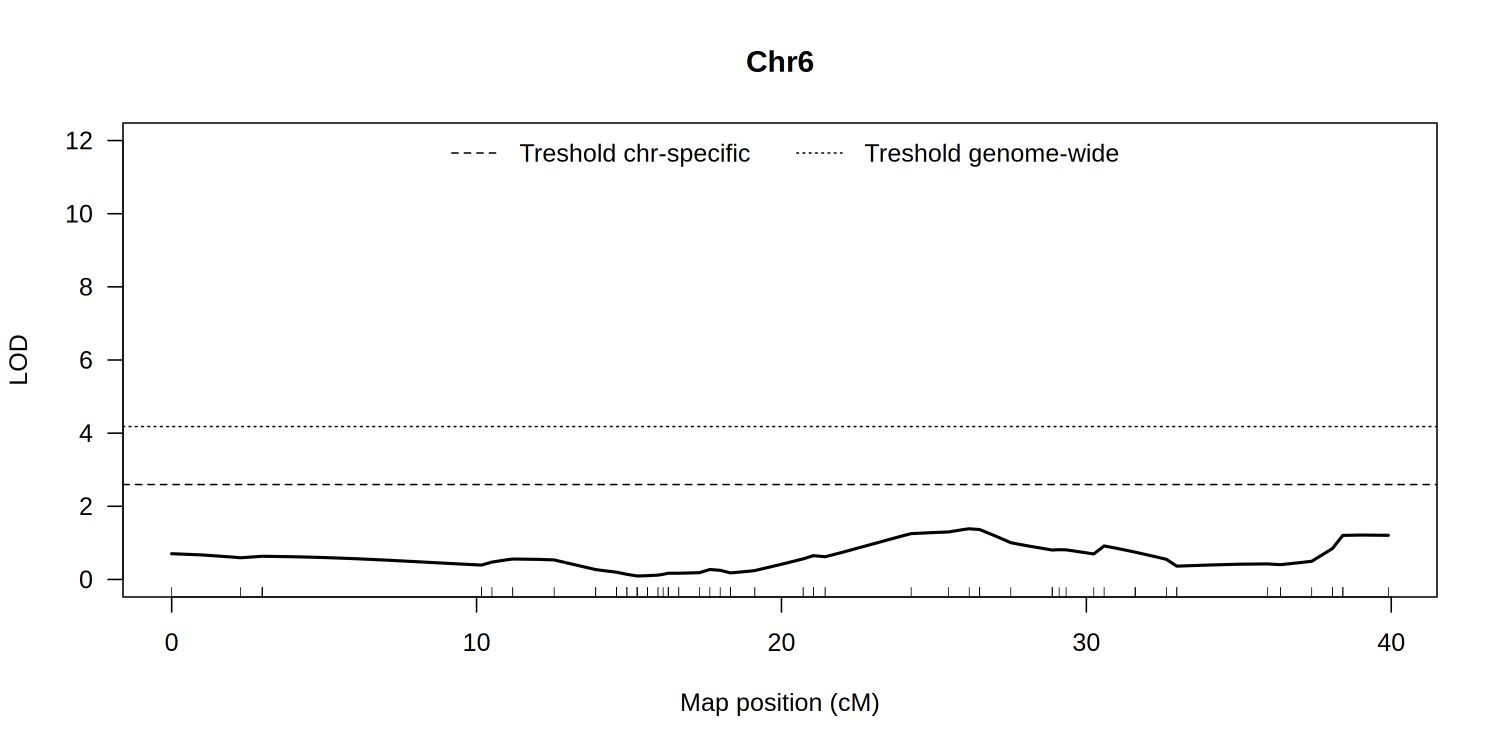

Supplement: Supplementary file 2 [file DataSheet2.zip › Supplementary_Files_4/QTL_analysis/alpha_terpineol/alpha_terpineol_LODplot_chr6.jpg]

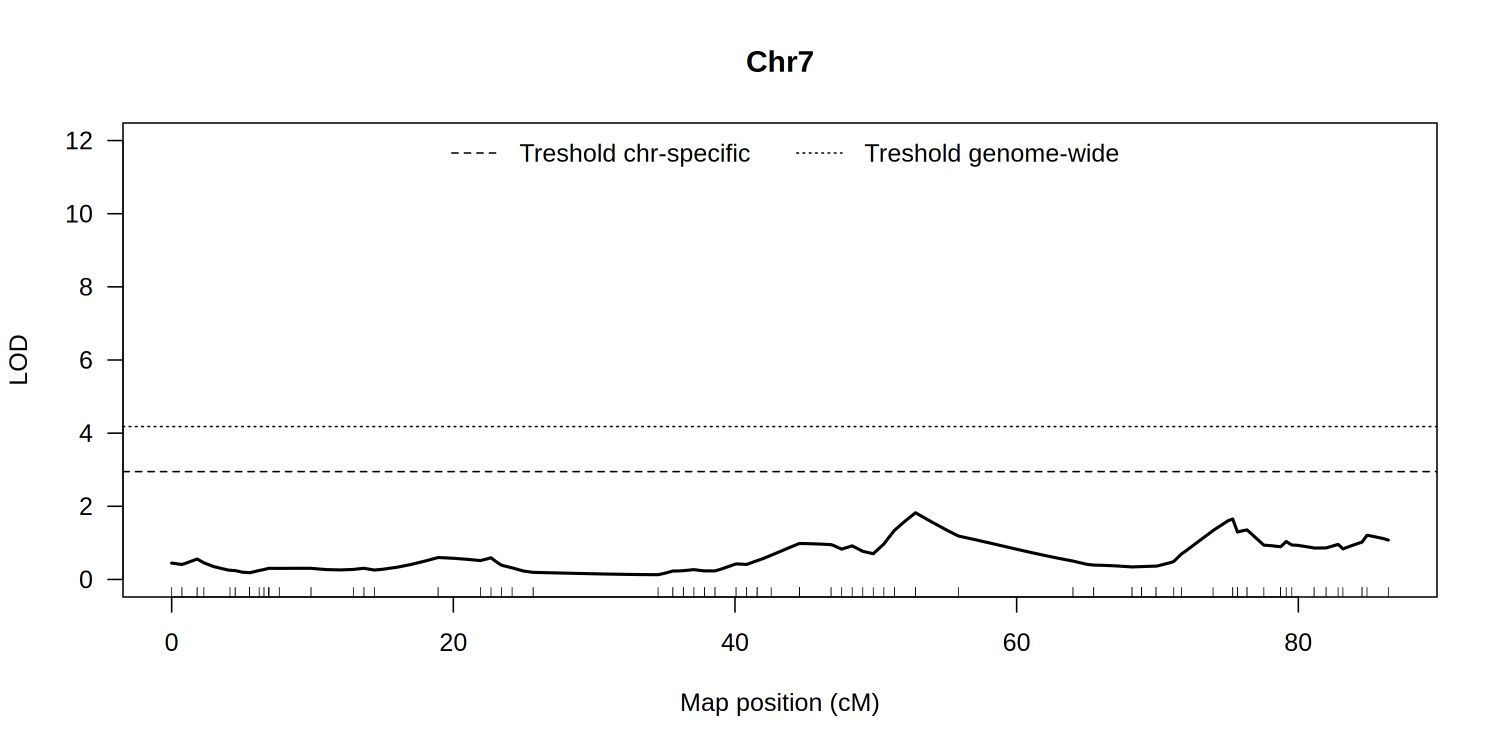

Supplement: Supplementary file 2 [file DataSheet2.zip › Supplementary_Files_4/QTL_analysis/alpha_terpineol/alpha_terpineol_LODplot_chr7.jpg]

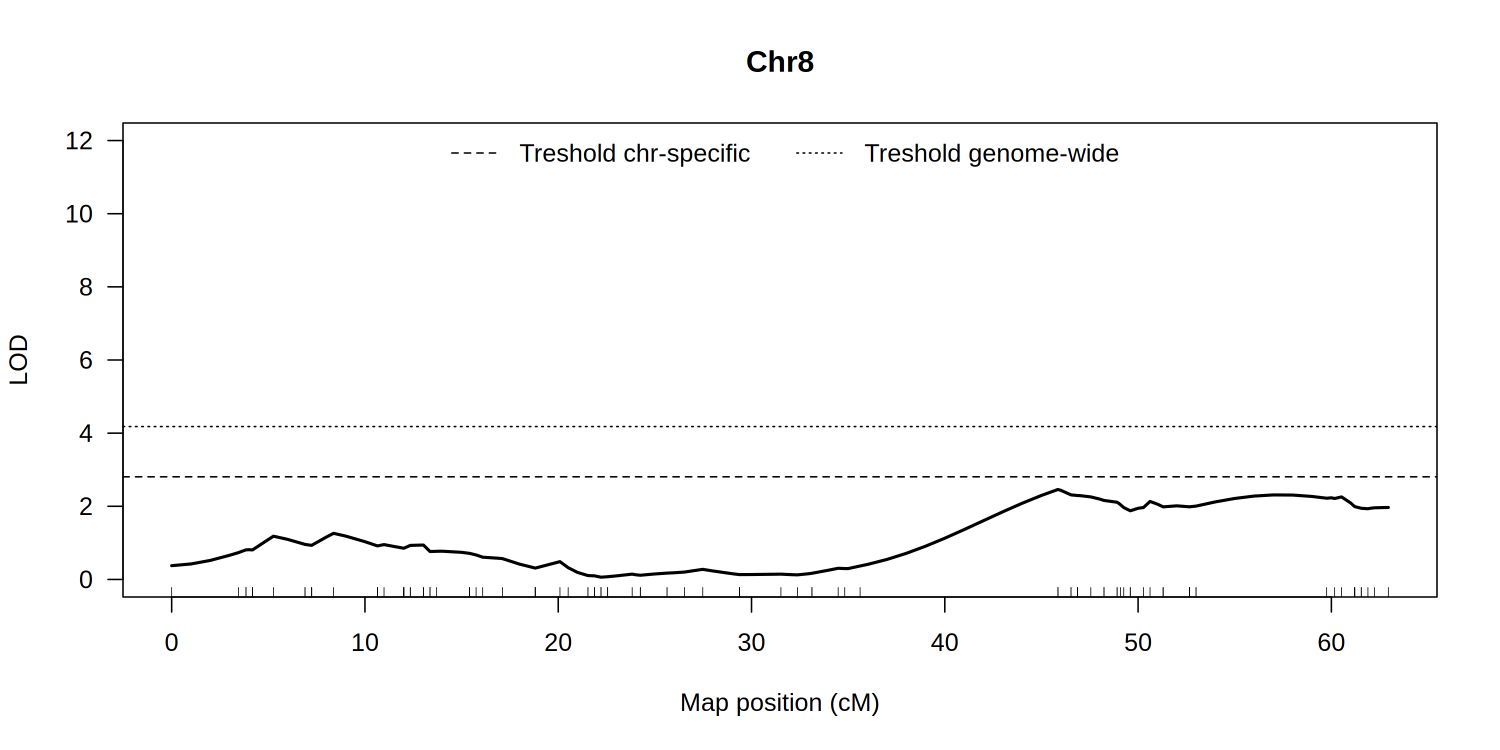

Supplement: Supplementary file 2 [file DataSheet2.zip › Supplementary_Files_4/QTL_analysis/alpha_terpineol/alpha_terpineol_LODplot_chr8.jpg]

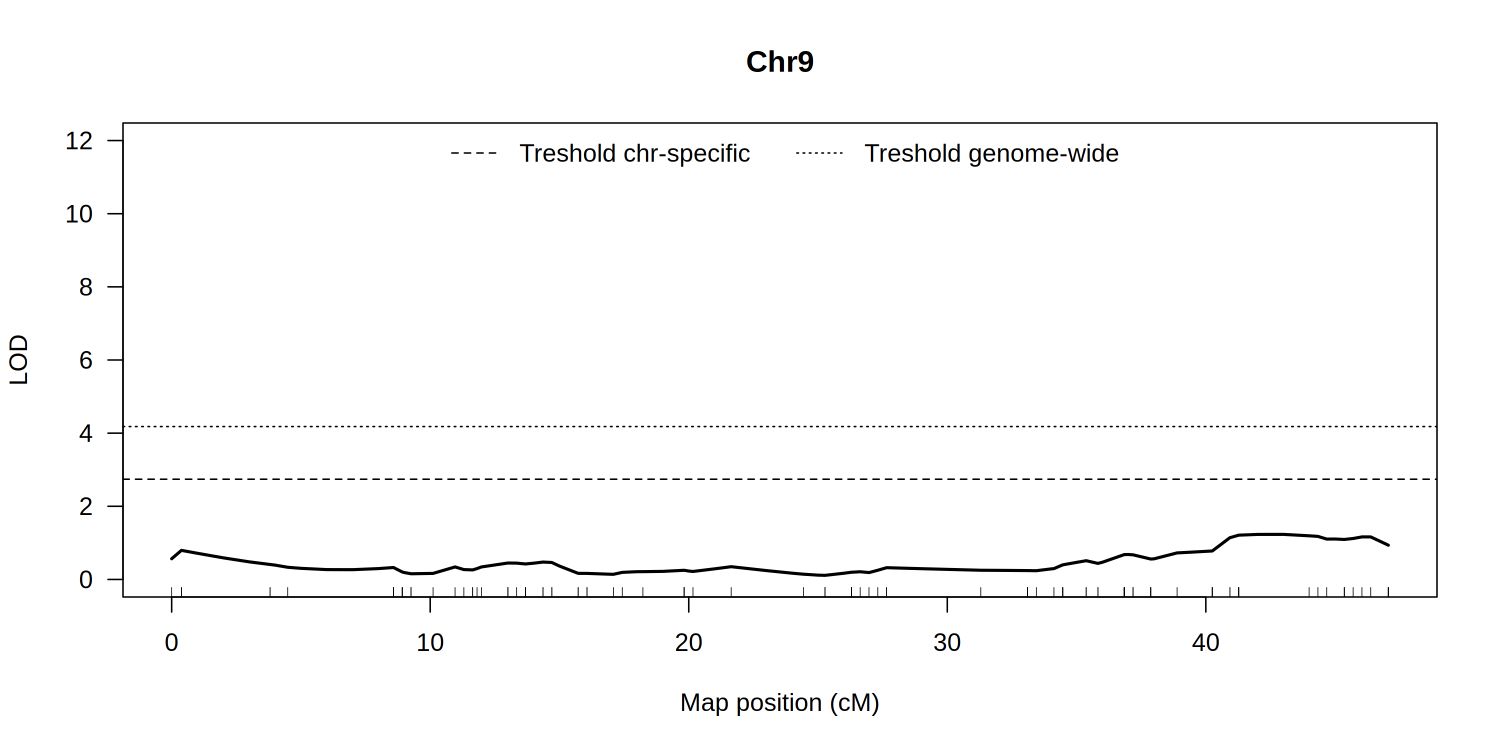

Supplement: Supplementary file 2 [file DataSheet2.zip › Supplementary_Files_4/QTL_analysis/alpha_terpineol/alpha_terpineol_LODplot_chr9.jpg]

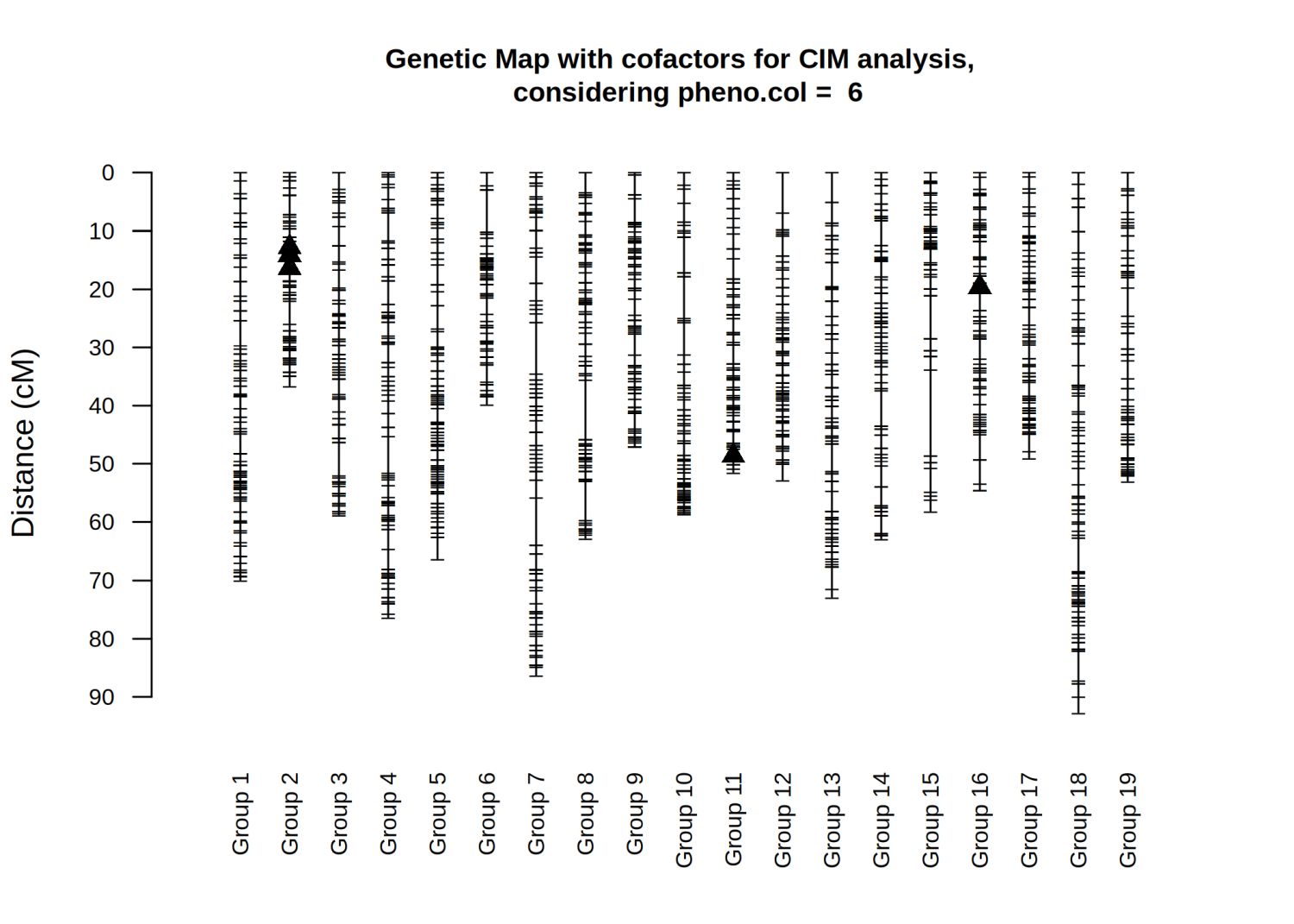

Supplement: Supplementary file 2 [file DataSheet2.zip › Supplementary_Files_4/QTL_analysis/alpha_terpineol/CIM analysis/Cofactors_pos.jpg]

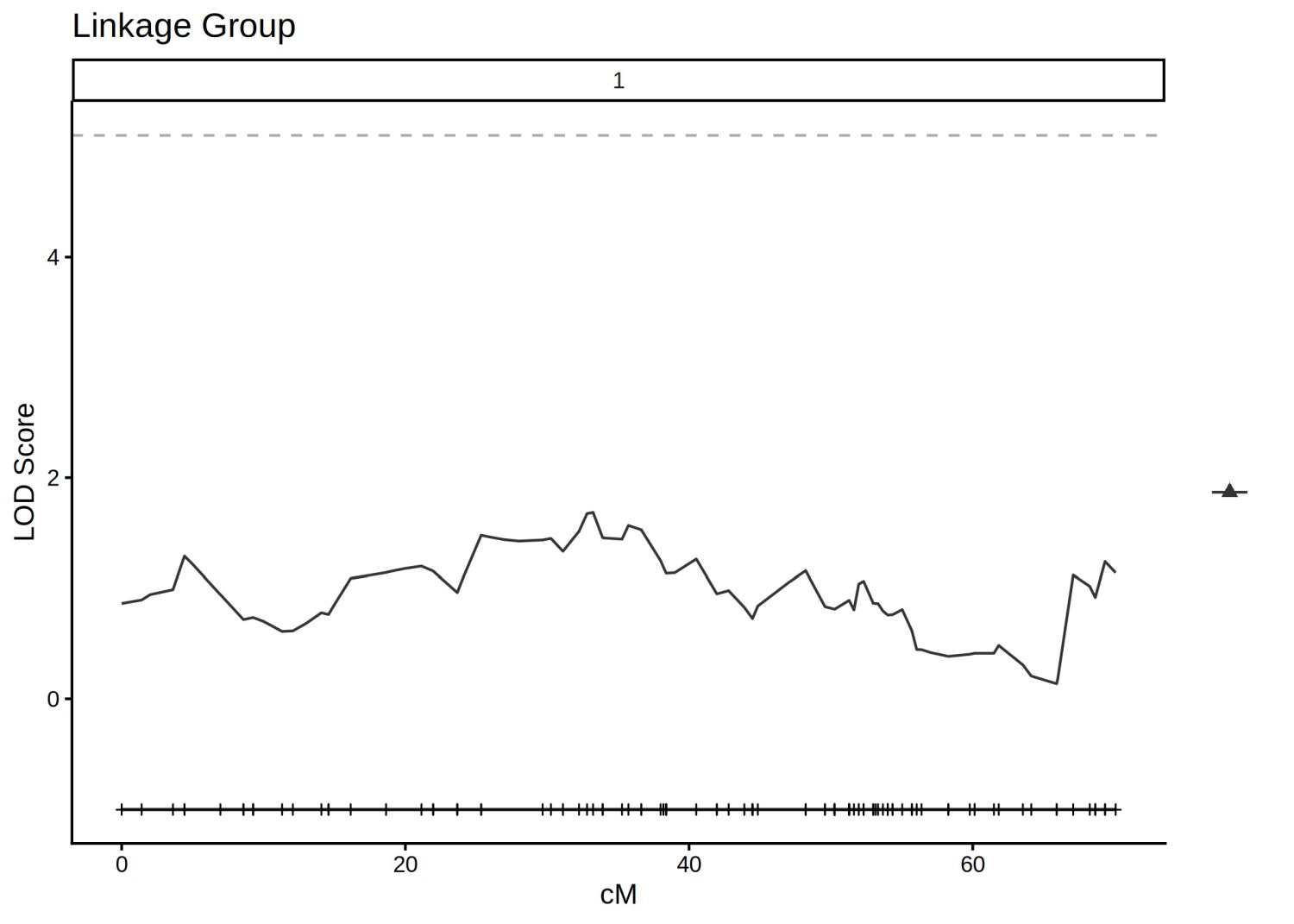

Supplement: Supplementary file 2 [file DataSheet2.zip › Supplementary_Files_4/QTL_analysis/alpha_terpineol/CIM analysis/LODplot_chr1.jpg]

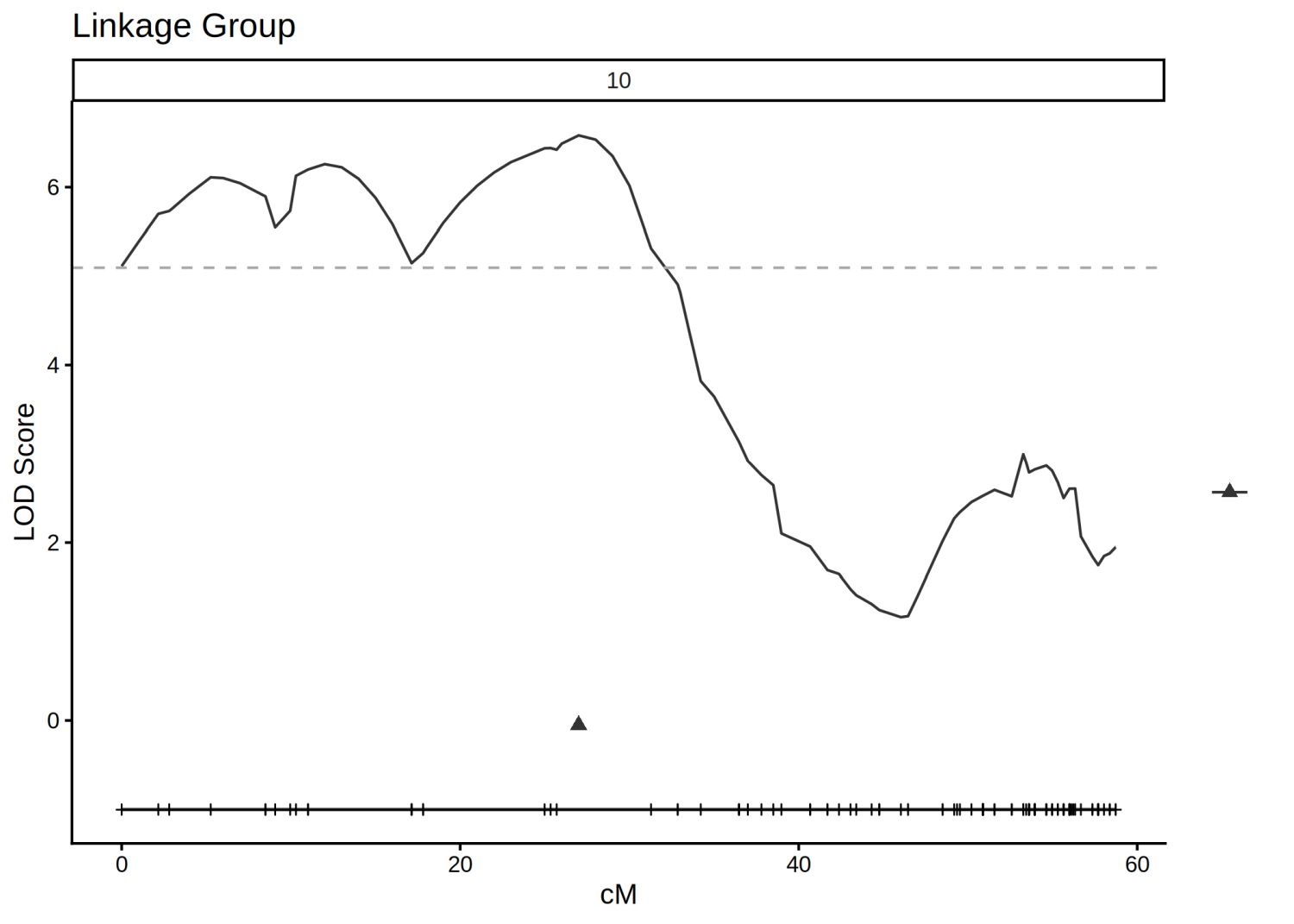

Supplement: Supplementary file 2 [file DataSheet2.zip › Supplementary_Files_4/QTL_analysis/alpha_terpineol/CIM analysis/LODplot_chr10.jpg]

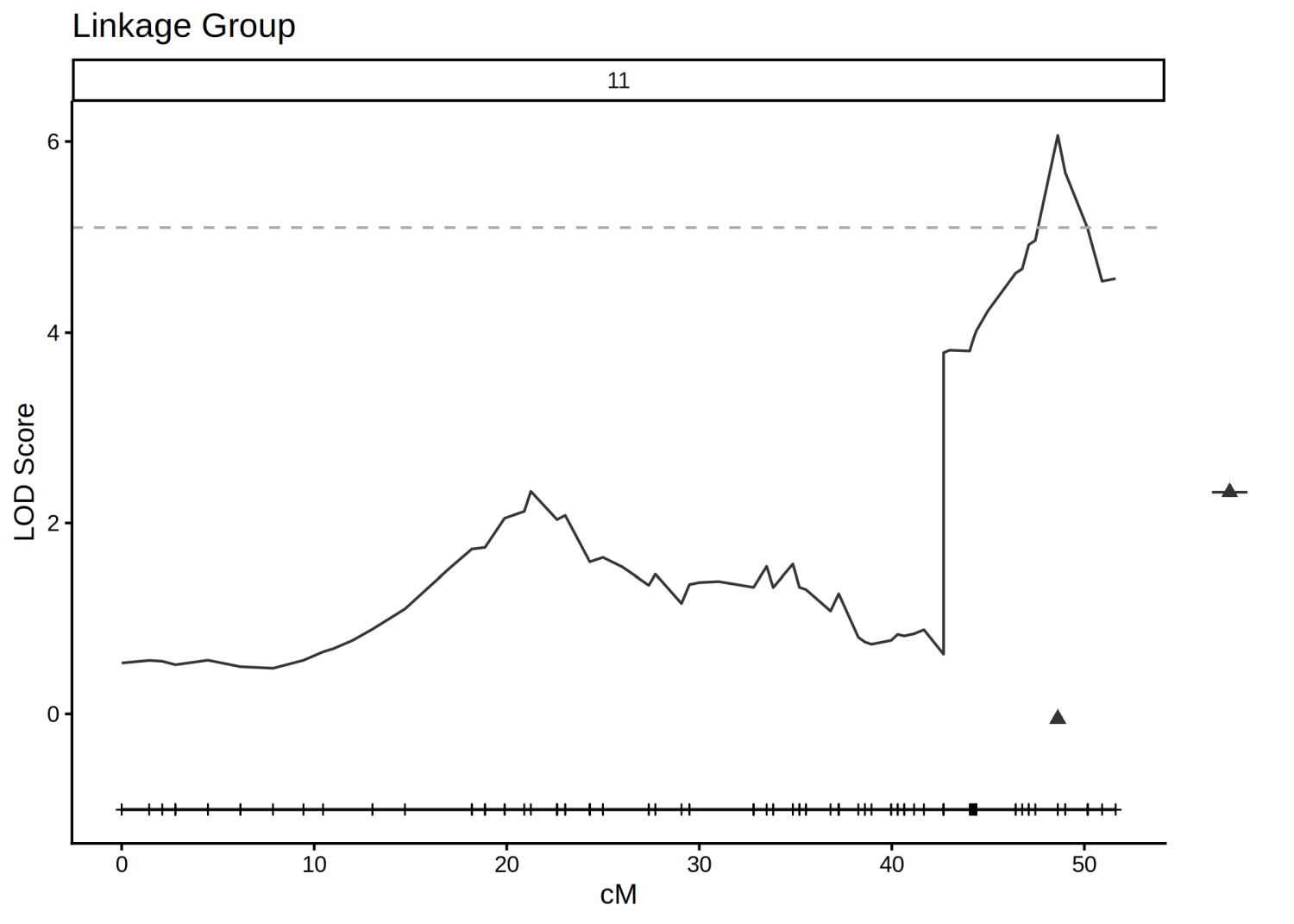

Supplement: Supplementary file 2 [file DataSheet2.zip › Supplementary_Files_4/QTL_analysis/alpha_terpineol/CIM analysis/LODplot_chr11.jpg]

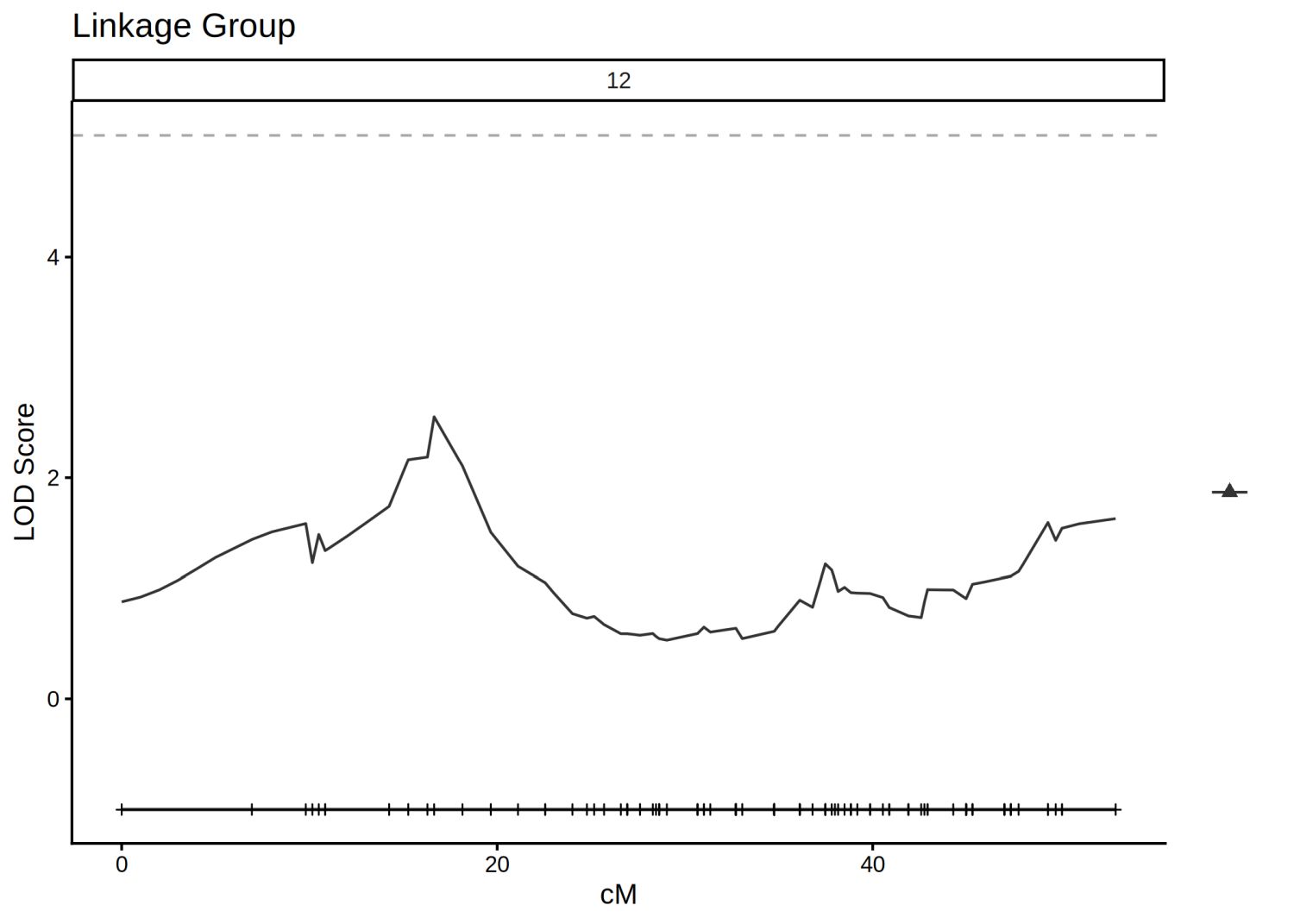

Supplement: Supplementary file 2 [file DataSheet2.zip › Supplementary_Files_4/QTL_analysis/alpha_terpineol/CIM analysis/LODplot_chr12.jpg]

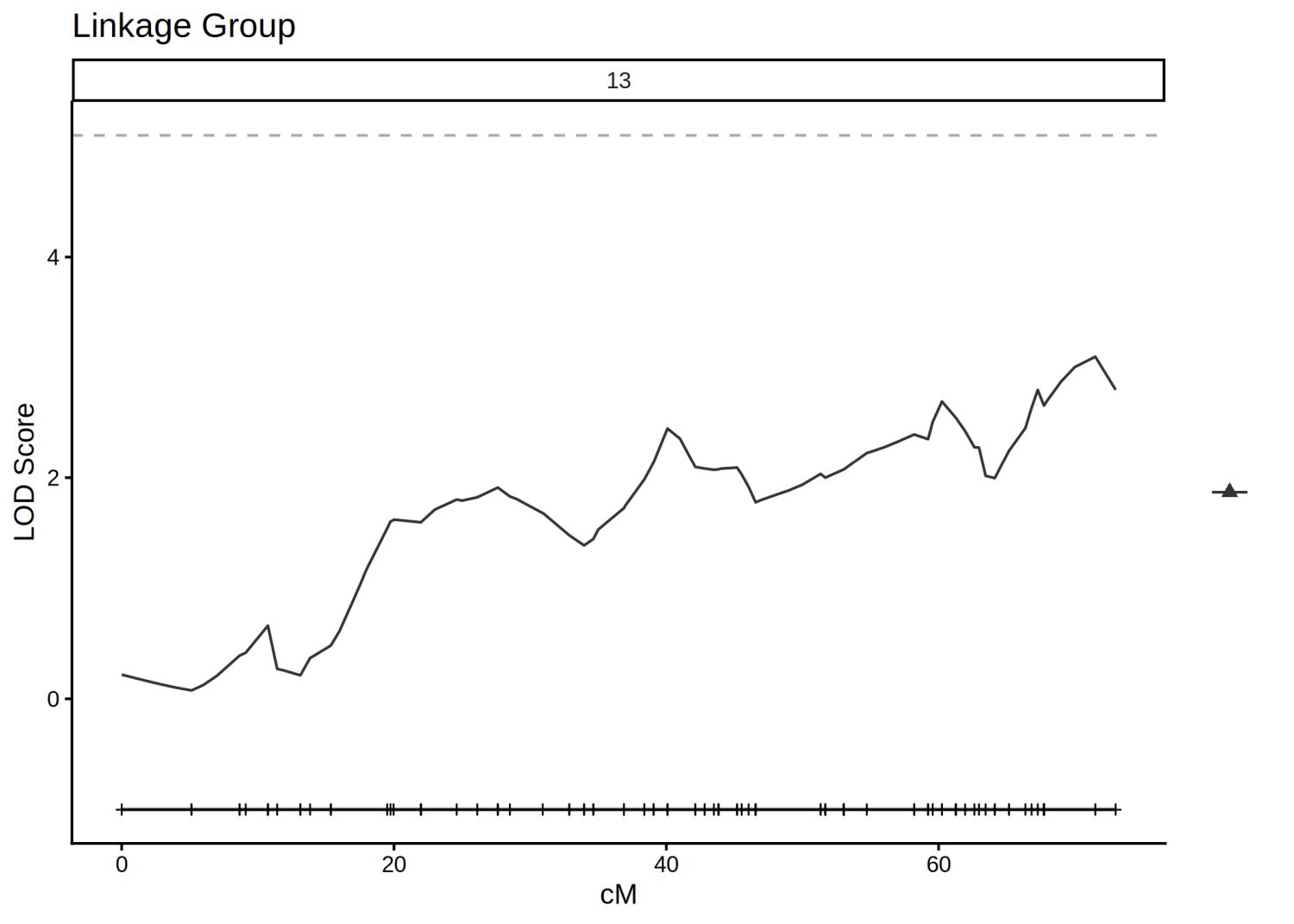

Supplement: Supplementary file 2 [file DataSheet2.zip › Supplementary_Files_4/QTL_analysis/alpha_terpineol/CIM analysis/LODplot_chr13.jpg]

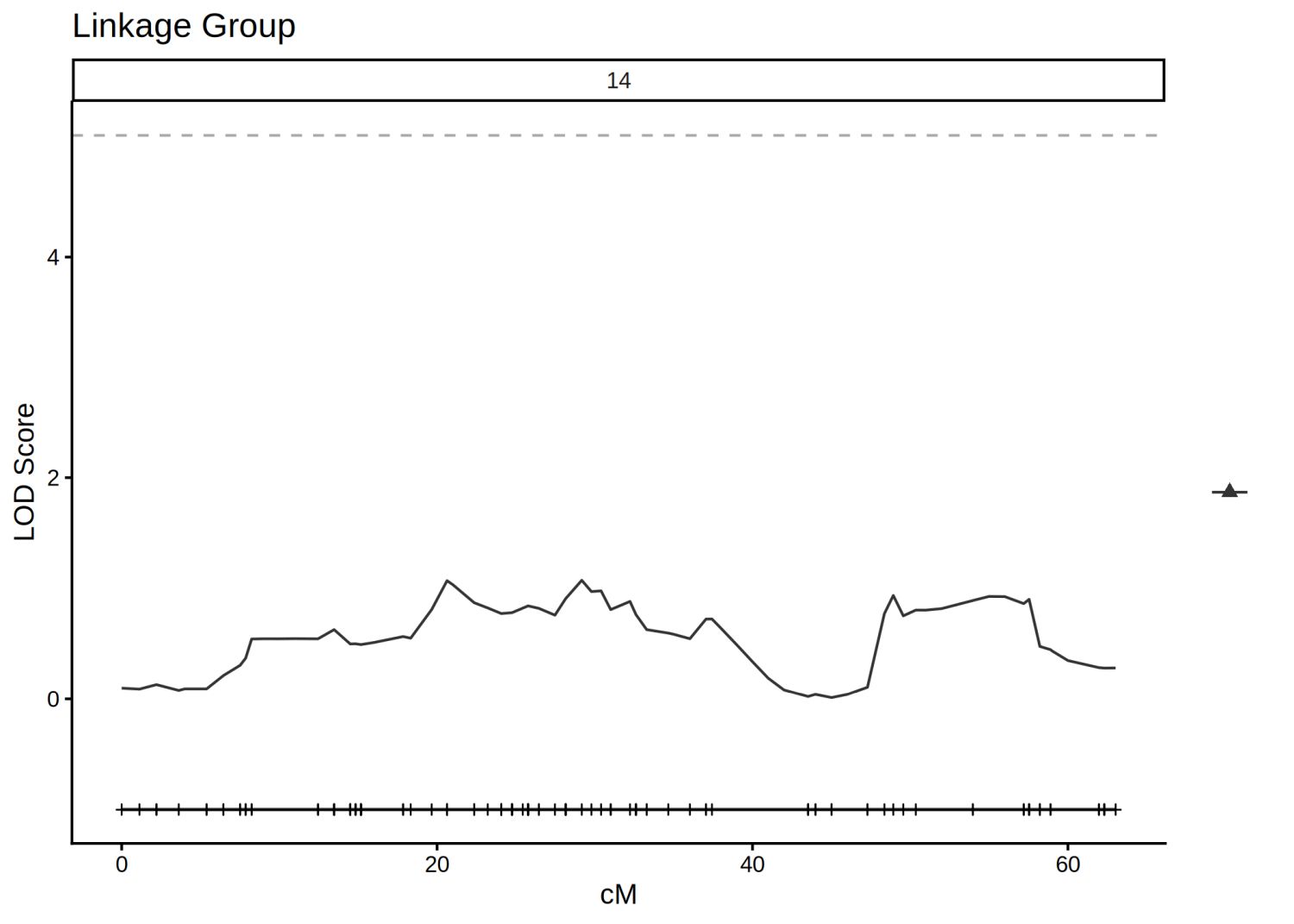

Supplement: Supplementary file 2 [file DataSheet2.zip › Supplementary_Files_4/QTL_analysis/alpha_terpineol/CIM analysis/LODplot_chr14.jpg]

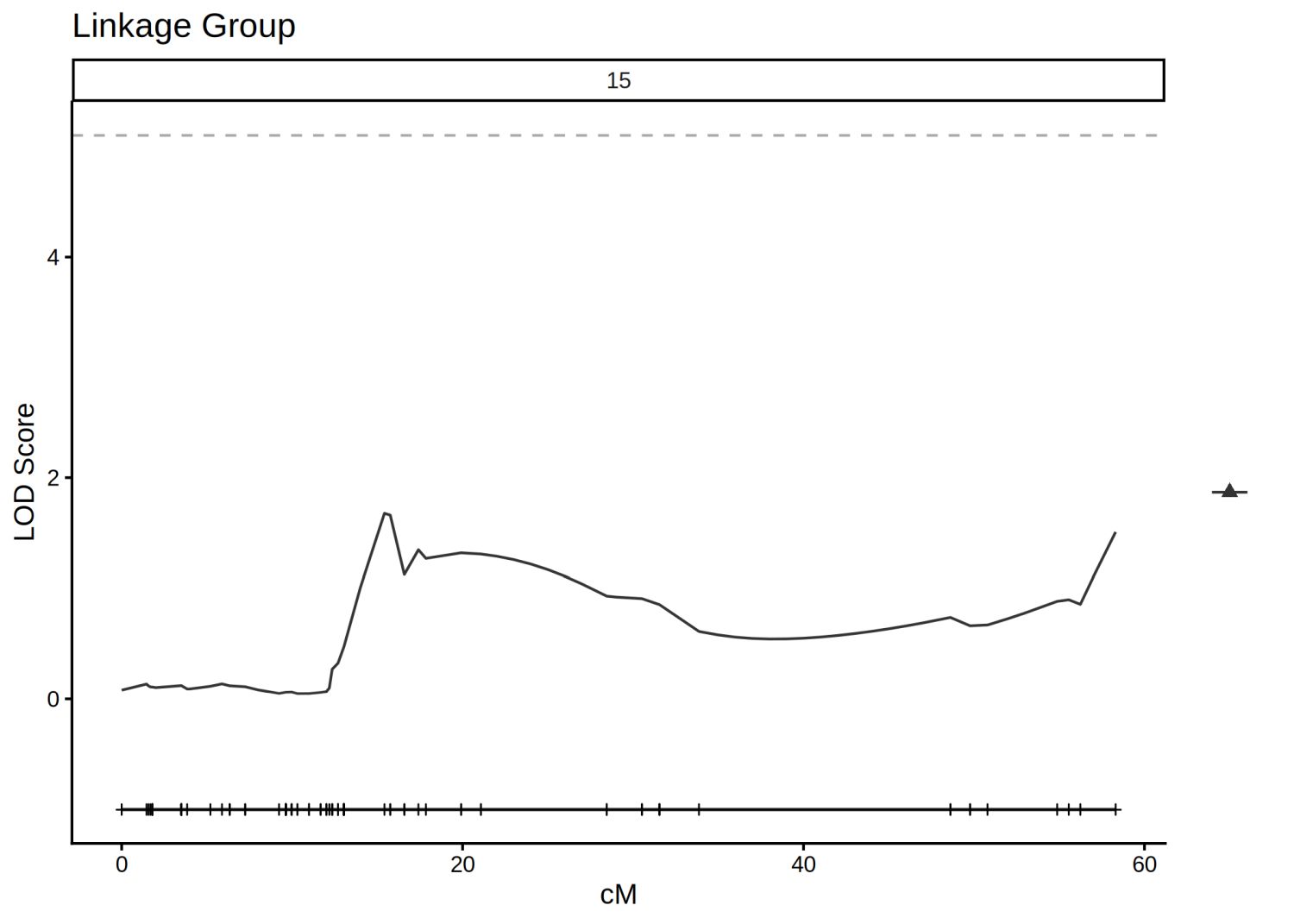

Supplement: Supplementary file 2 [file DataSheet2.zip › Supplementary_Files_4/QTL_analysis/alpha_terpineol/CIM analysis/LODplot_chr15.jpg]

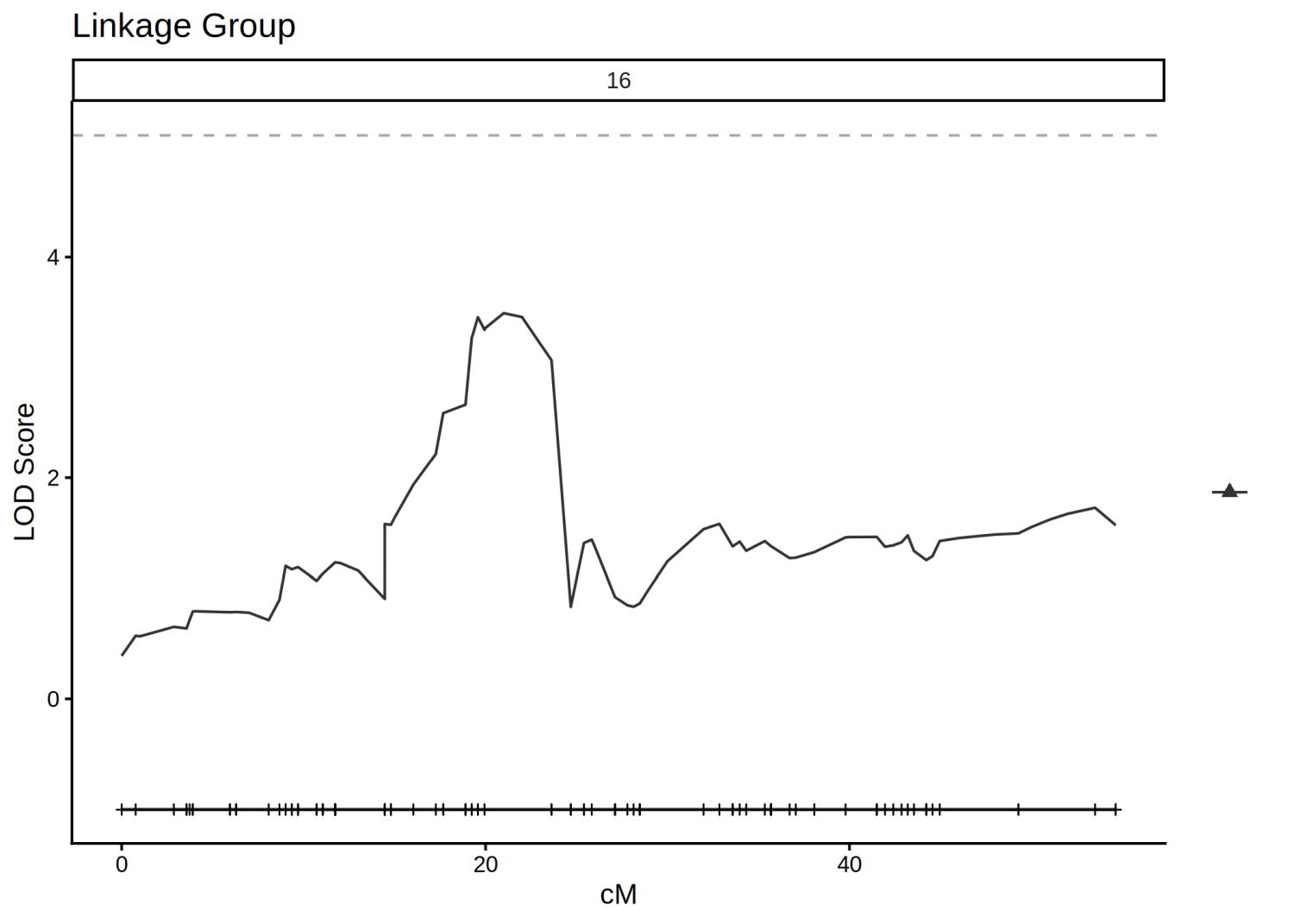

Supplement: Supplementary file 2 [file DataSheet2.zip › Supplementary_Files_4/QTL_analysis/alpha_terpineol/CIM analysis/LODplot_chr16.jpg]

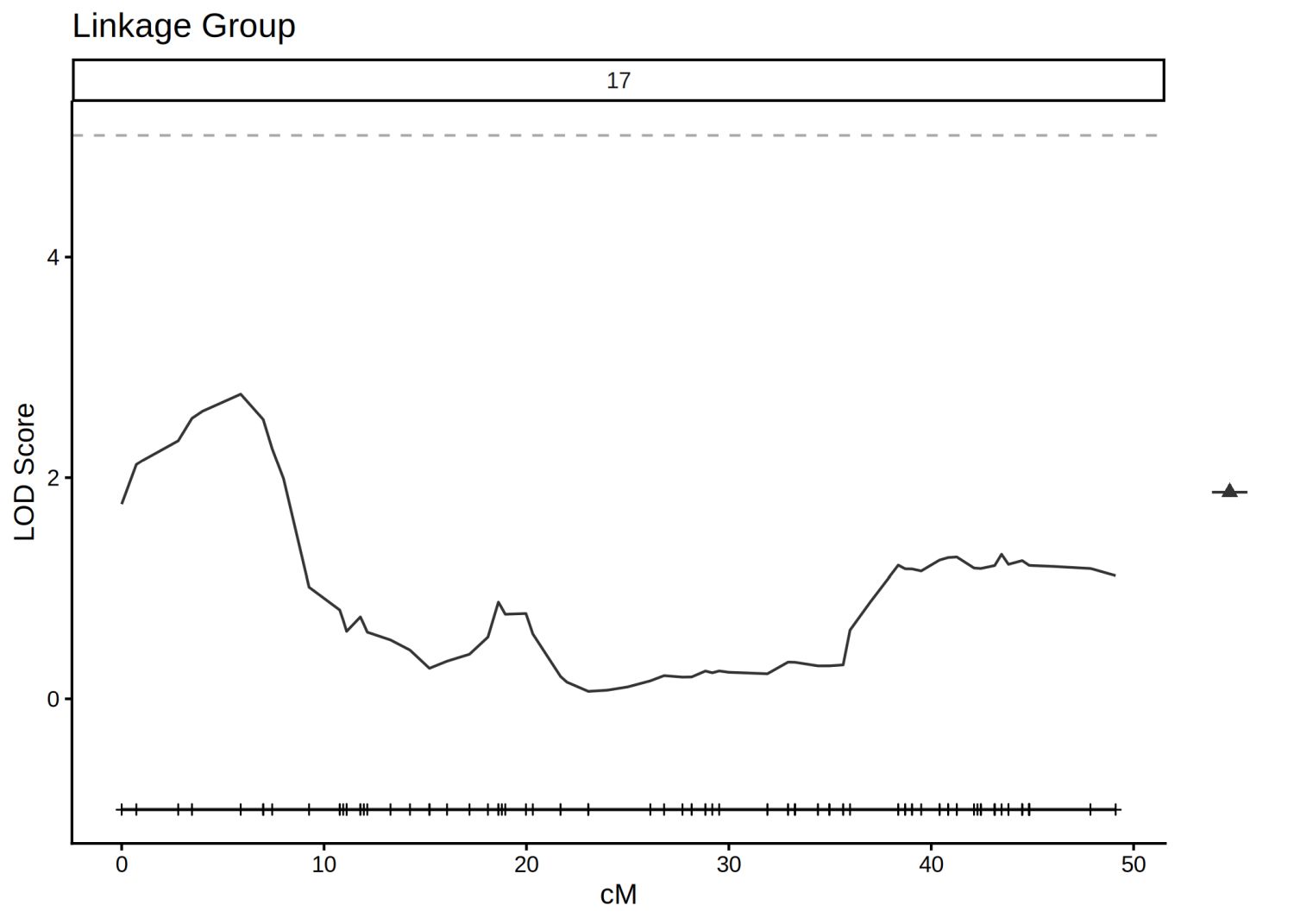

Supplement: Supplementary file 2 [file DataSheet2.zip › Supplementary_Files_4/QTL_analysis/alpha_terpineol/CIM analysis/LODplot_chr17.jpg]

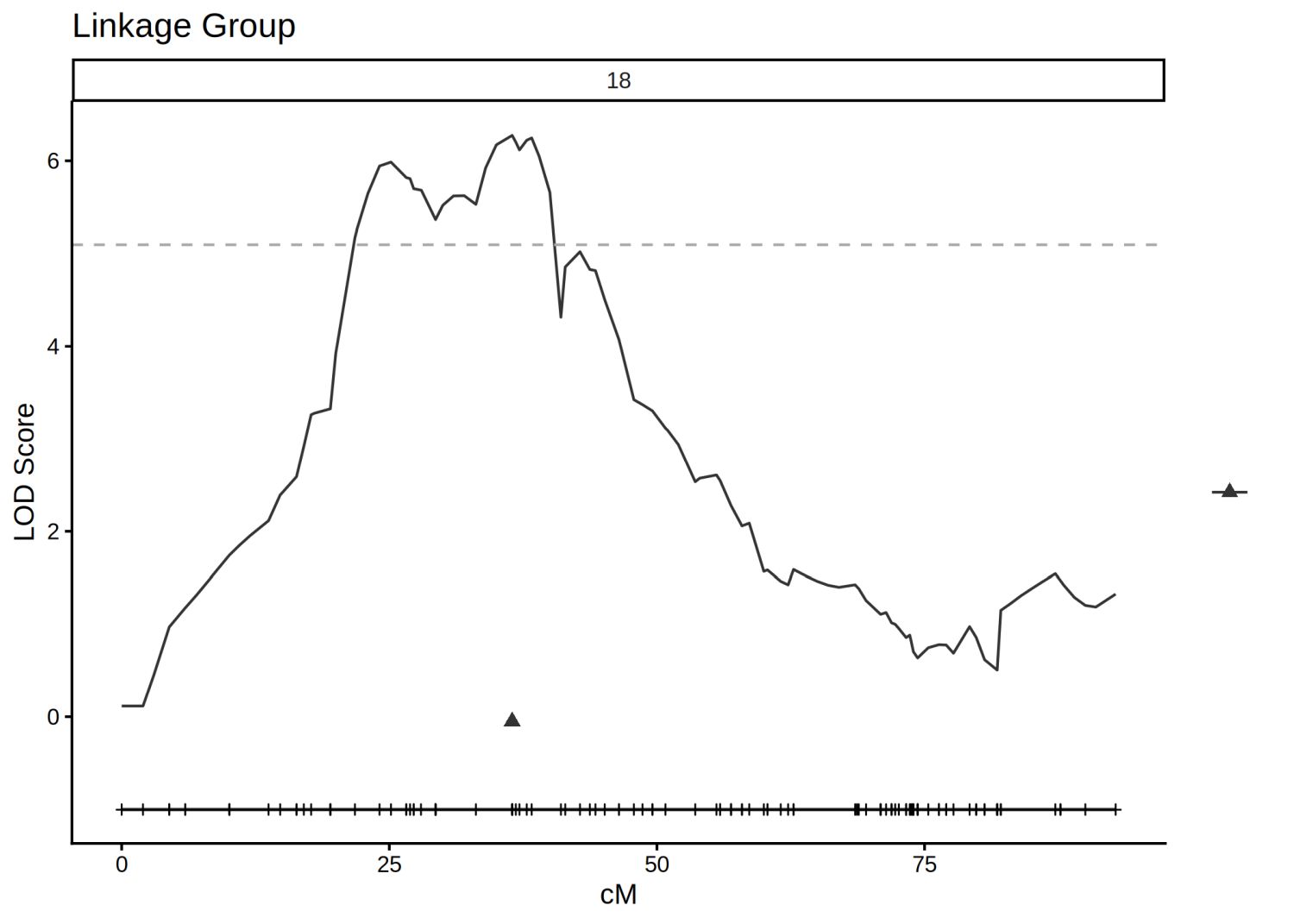

Supplement: Supplementary file 2 [file DataSheet2.zip › Supplementary_Files_4/QTL_analysis/alpha_terpineol/CIM analysis/LODplot_chr18.jpg]

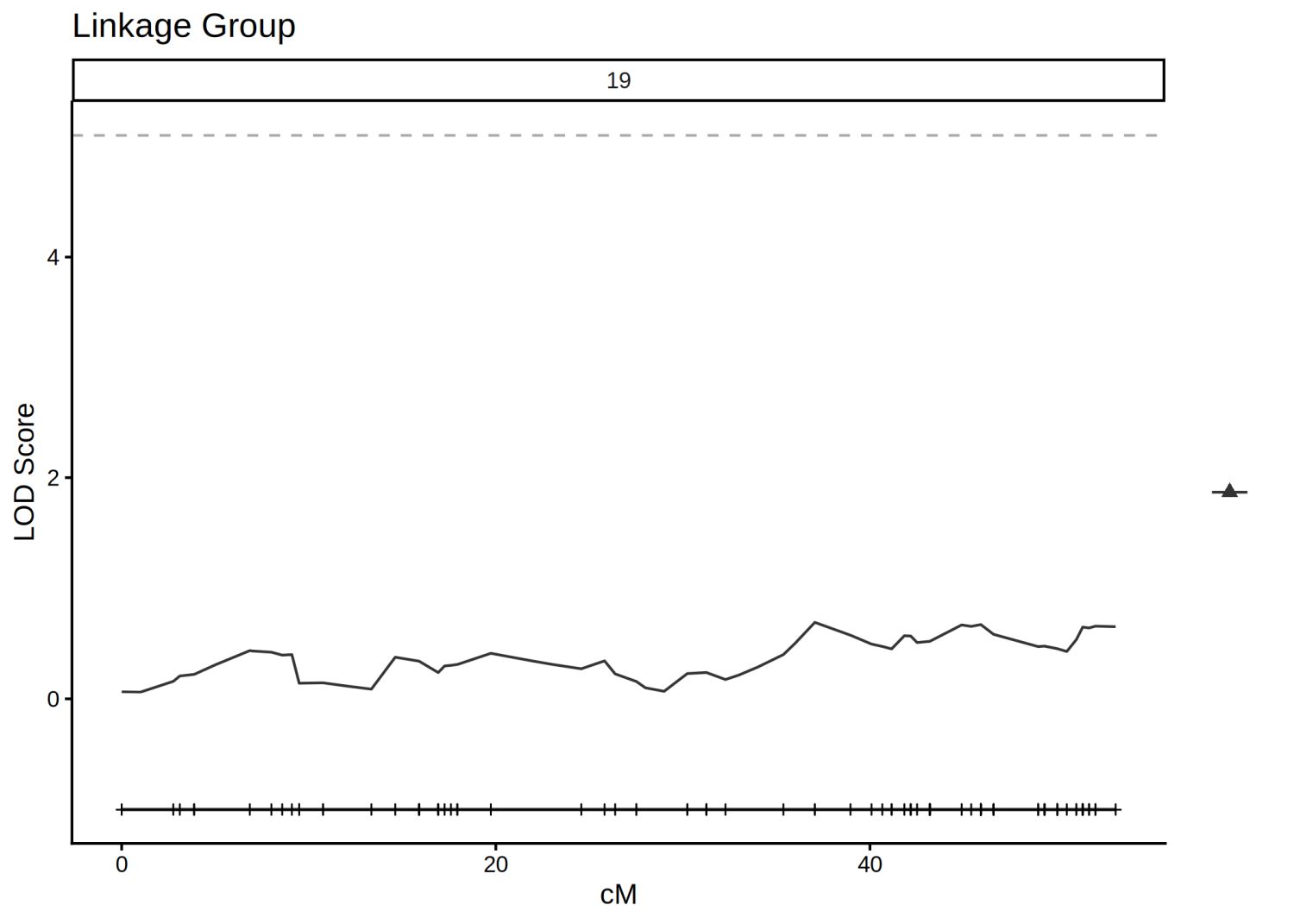

Supplement: Supplementary file 2 [file DataSheet2.zip › Supplementary_Files_4/QTL_analysis/alpha_terpineol/CIM analysis/LODplot_chr19.jpg]

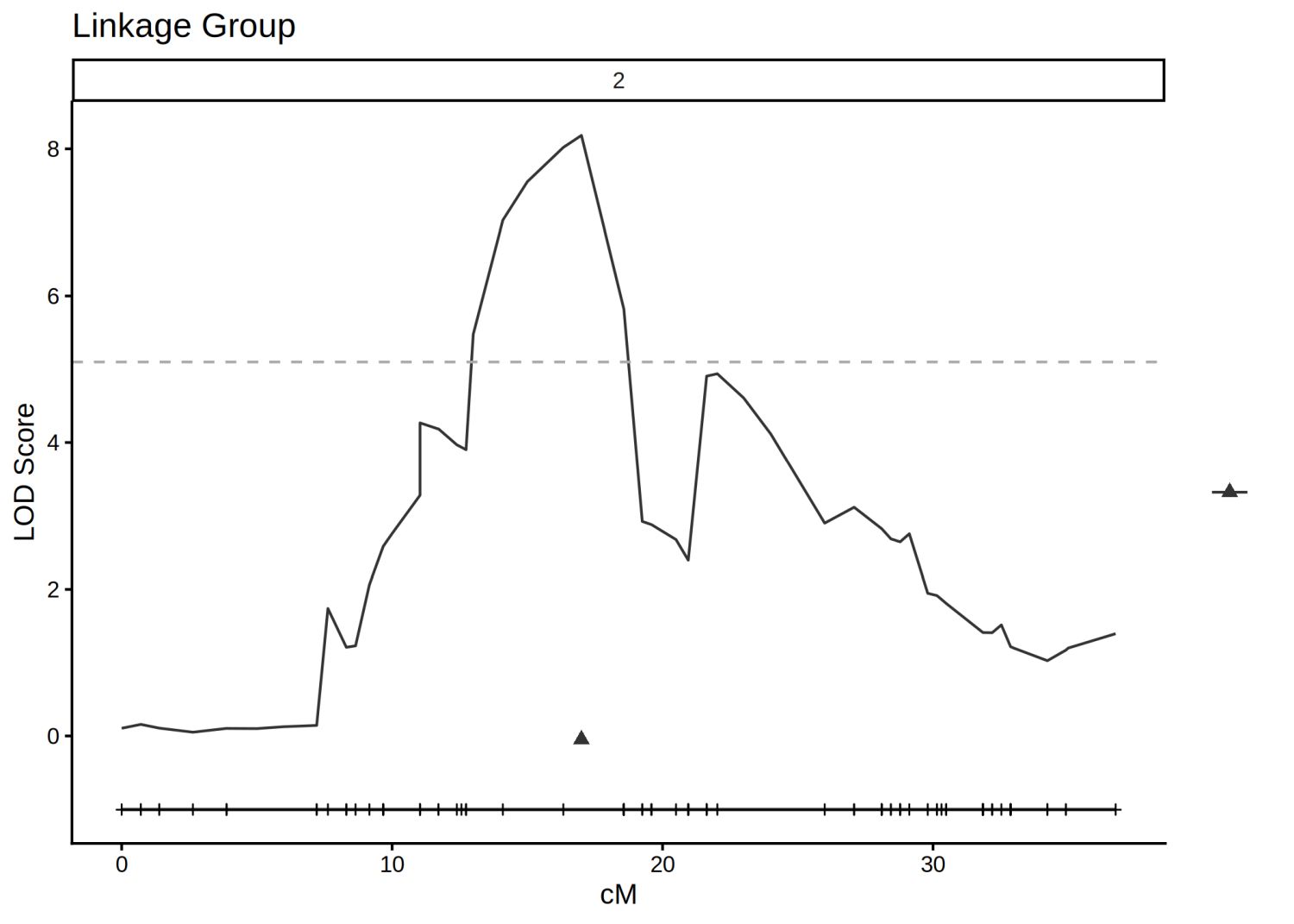

Supplement: Supplementary file 2 [file DataSheet2.zip › Supplementary_Files_4/QTL_analysis/alpha_terpineol/CIM analysis/LODplot_chr2.jpg]

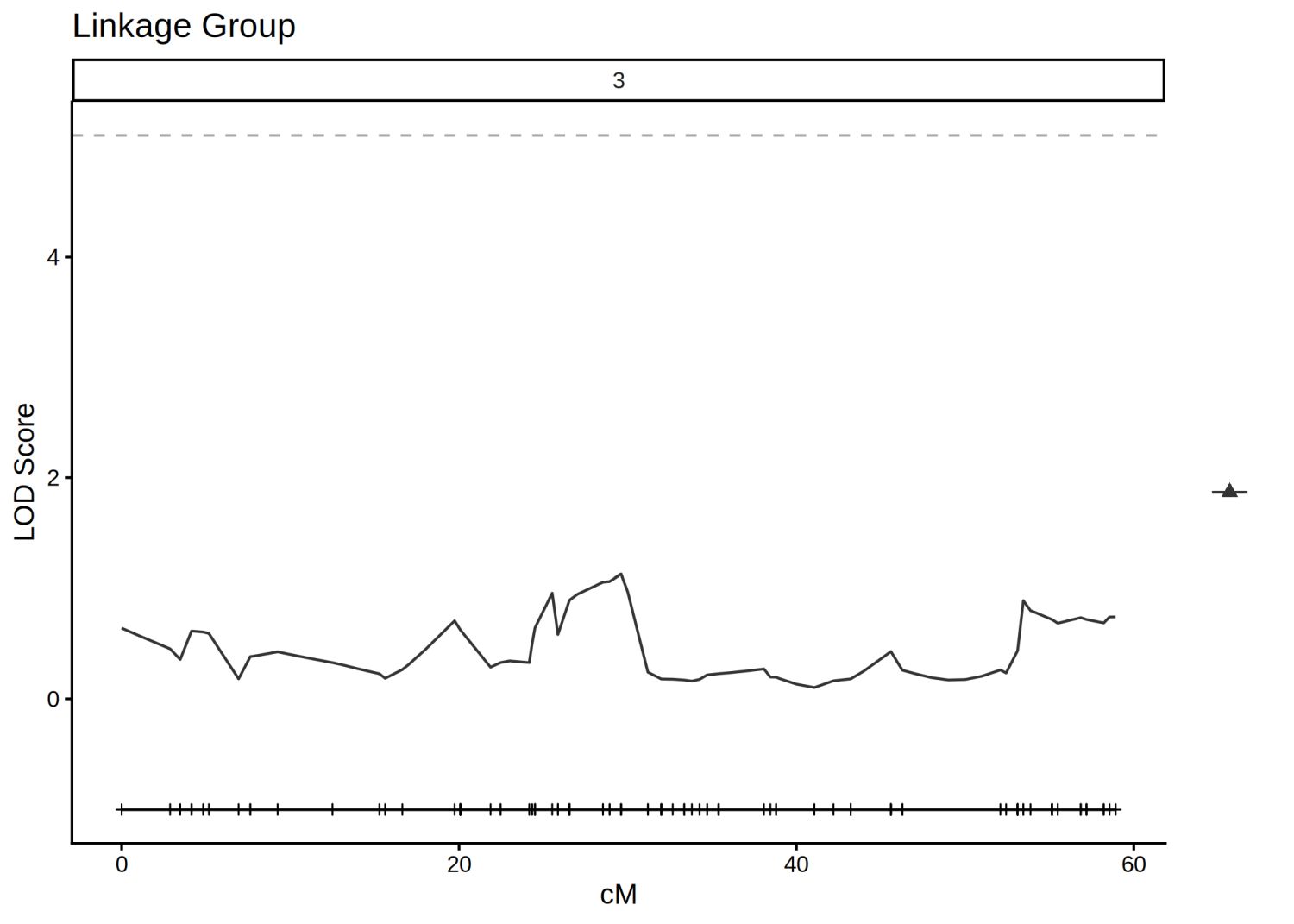

Supplement: Supplementary file 2 [file DataSheet2.zip › Supplementary_Files_4/QTL_analysis/alpha_terpineol/CIM analysis/LODplot_chr3.jpg]

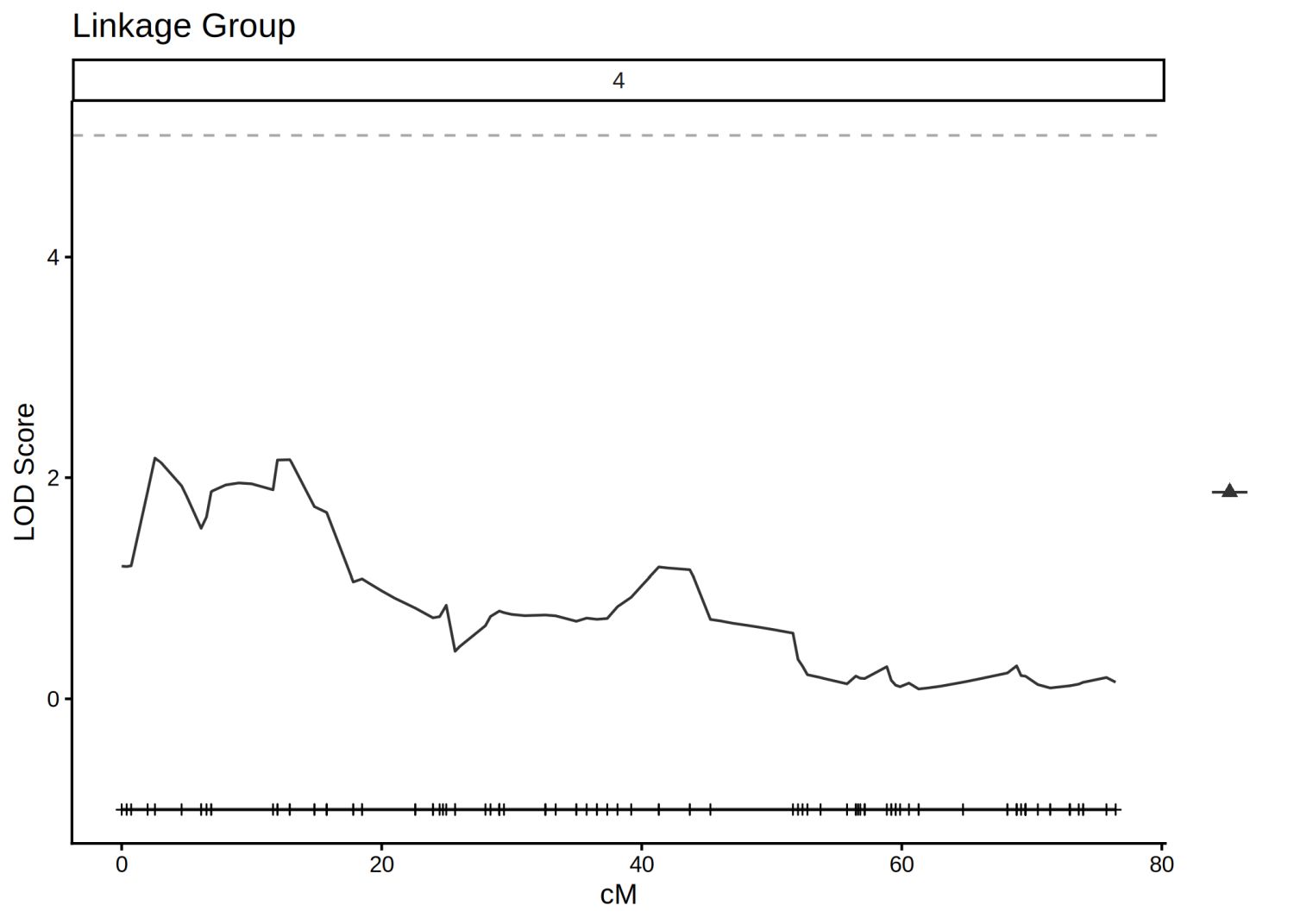

Supplement: Supplementary file 2 [file DataSheet2.zip › Supplementary_Files_4/QTL_analysis/alpha_terpineol/CIM analysis/LODplot_chr4.jpg]

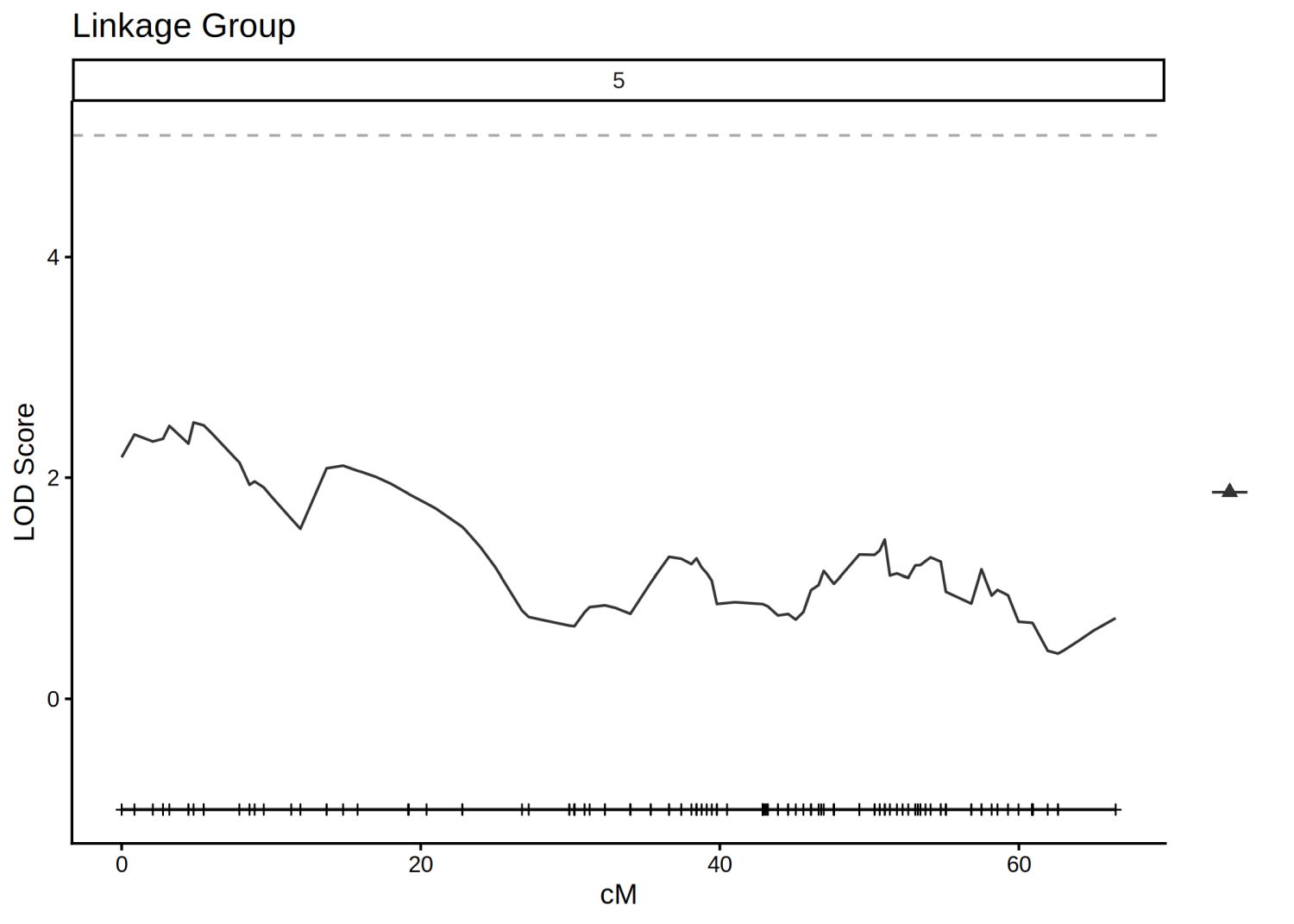

Supplement: Supplementary file 2 [file DataSheet2.zip › Supplementary_Files_4/QTL_analysis/alpha_terpineol/CIM analysis/LODplot_chr5.jpg]

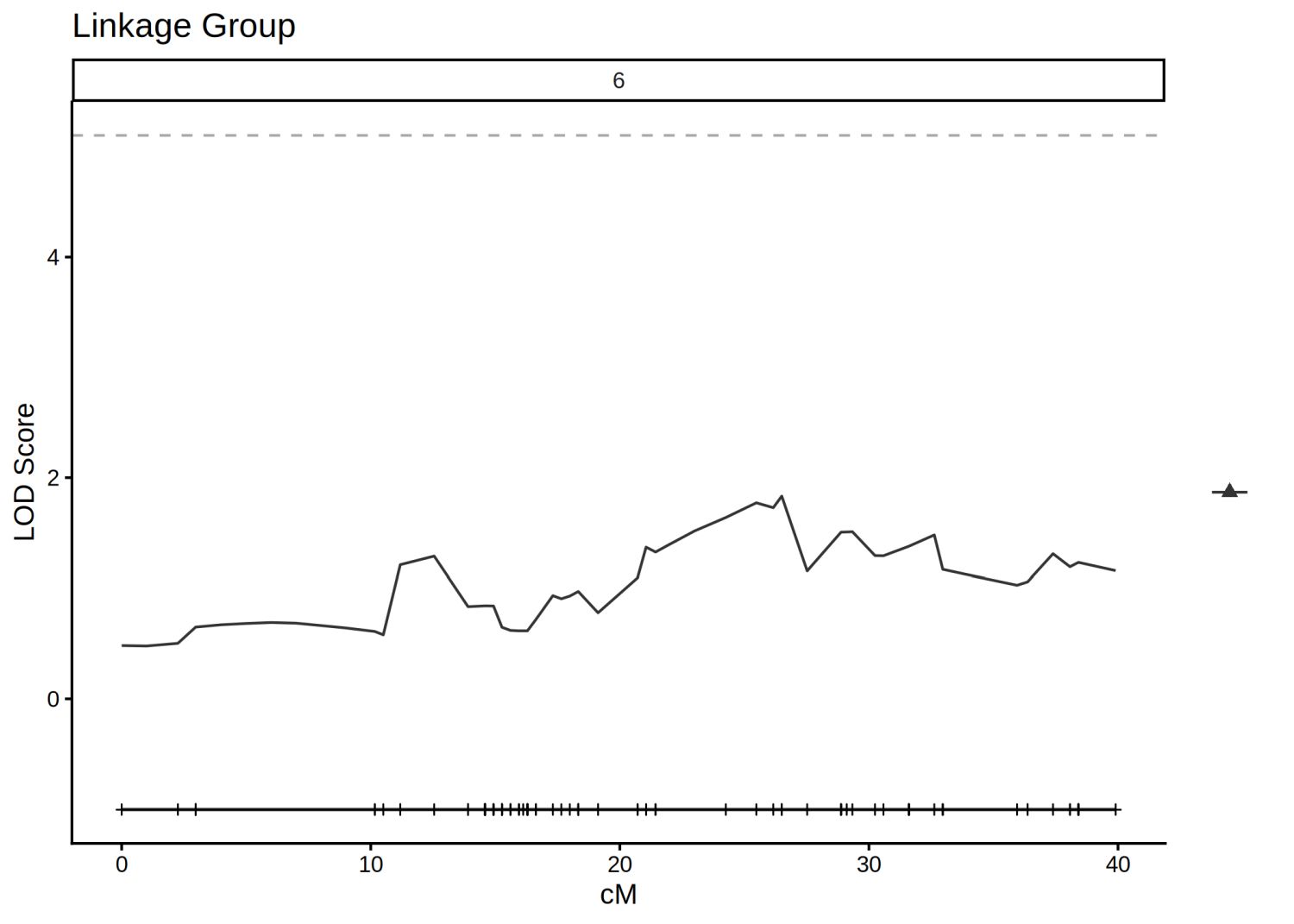

Supplement: Supplementary file 2 [file DataSheet2.zip › Supplementary_Files_4/QTL_analysis/alpha_terpineol/CIM analysis/LODplot_chr6.jpg]

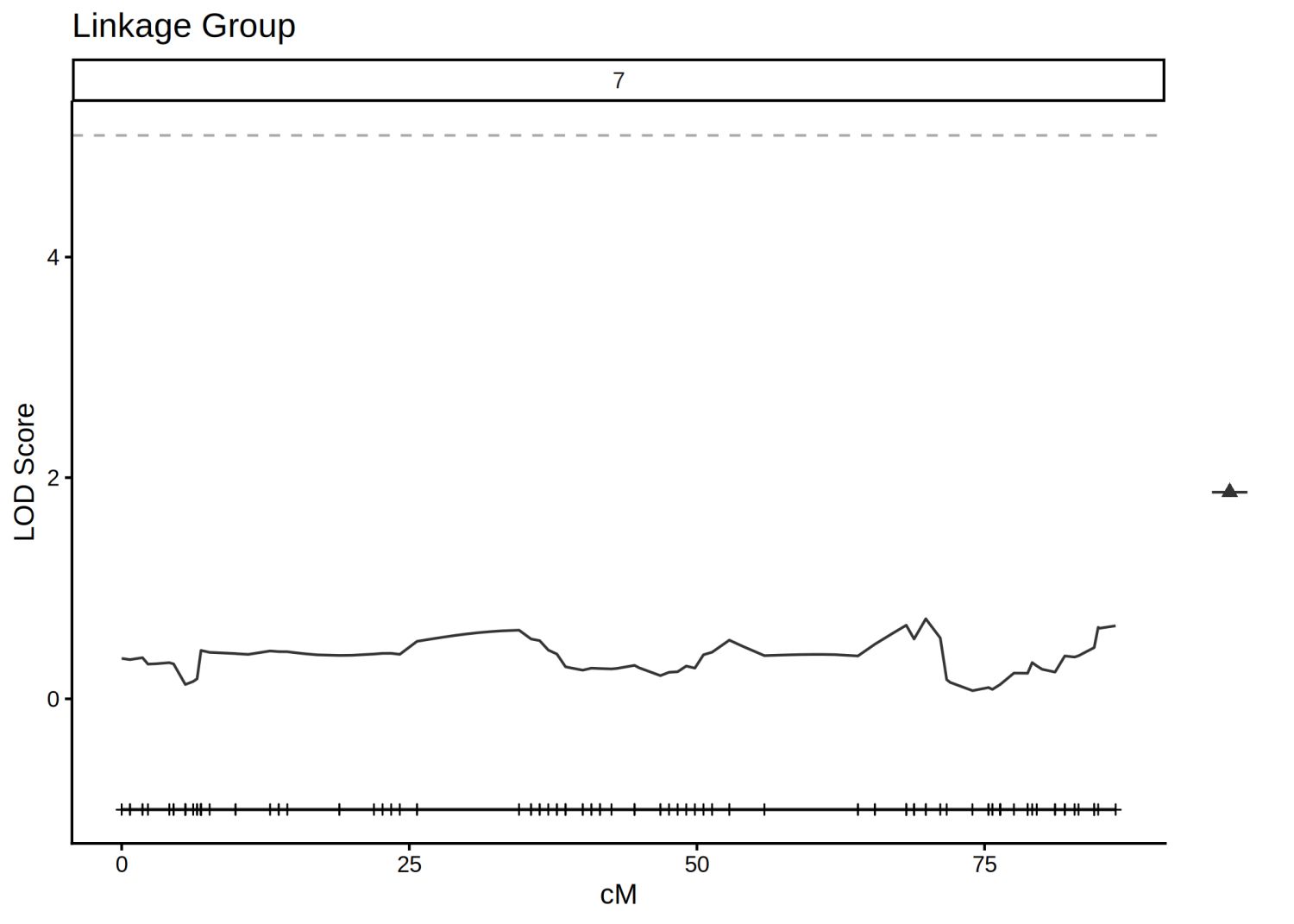

Supplement: Supplementary file 2 [file DataSheet2.zip › Supplementary_Files_4/QTL_analysis/alpha_terpineol/CIM analysis/LODplot_chr7.jpg]

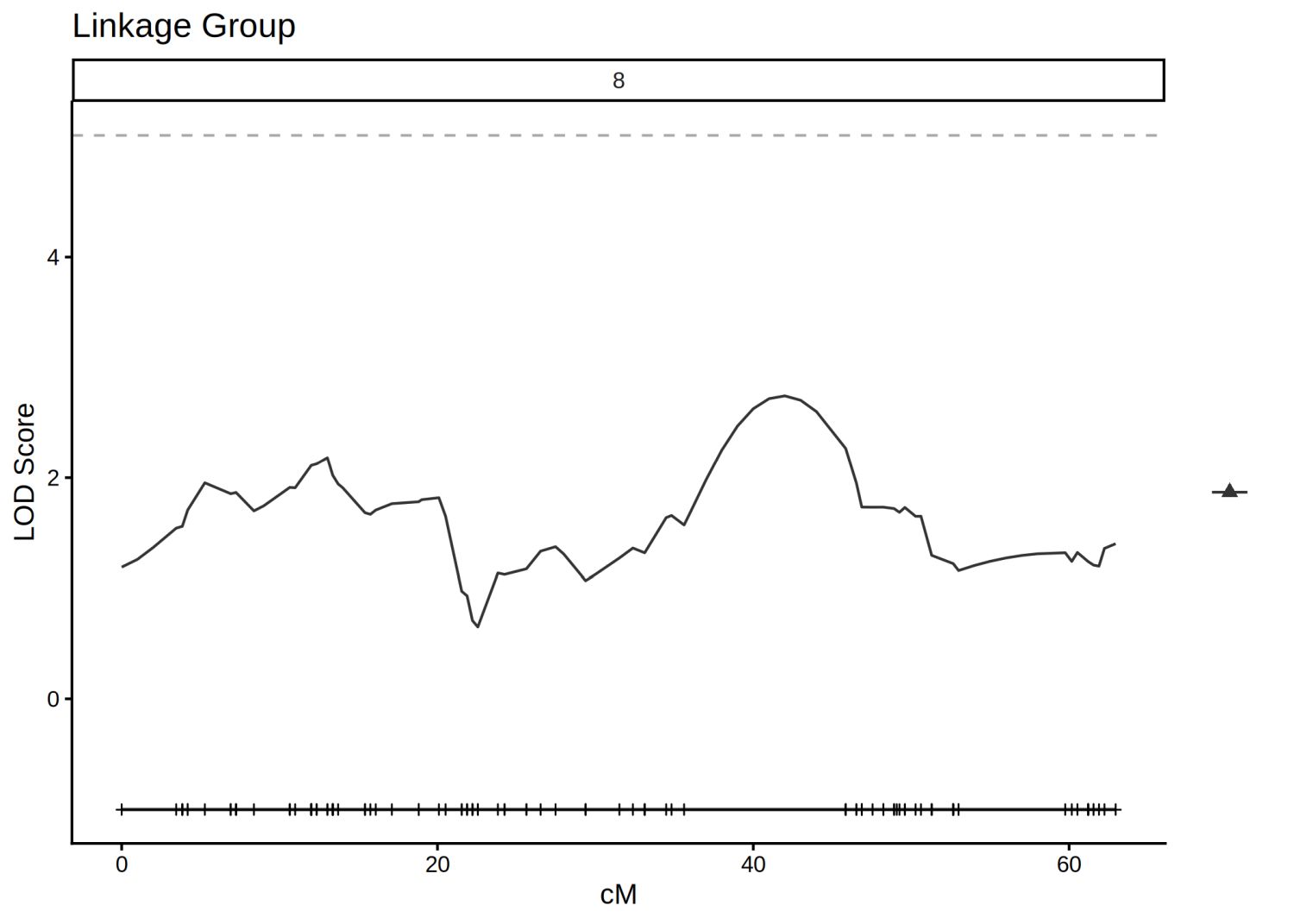

Supplement: Supplementary file 2 [file DataSheet2.zip › Supplementary_Files_4/QTL_analysis/alpha_terpineol/CIM analysis/LODplot_chr8.jpg]

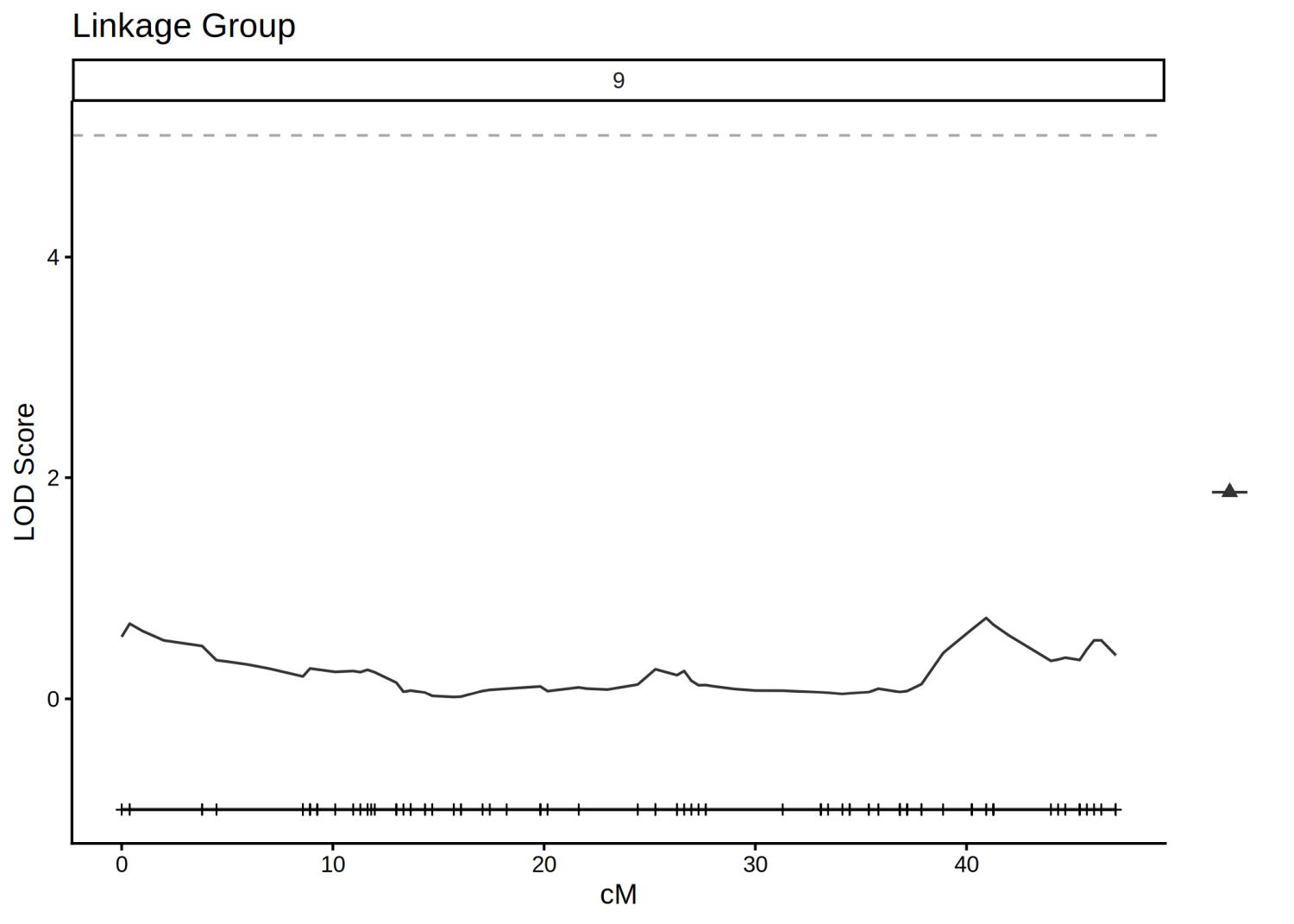

Supplement: Supplementary file 2 [file DataSheet2.zip › Supplementary_Files_4/QTL_analysis/alpha_terpineol/CIM analysis/LODplot_chr9.jpg]

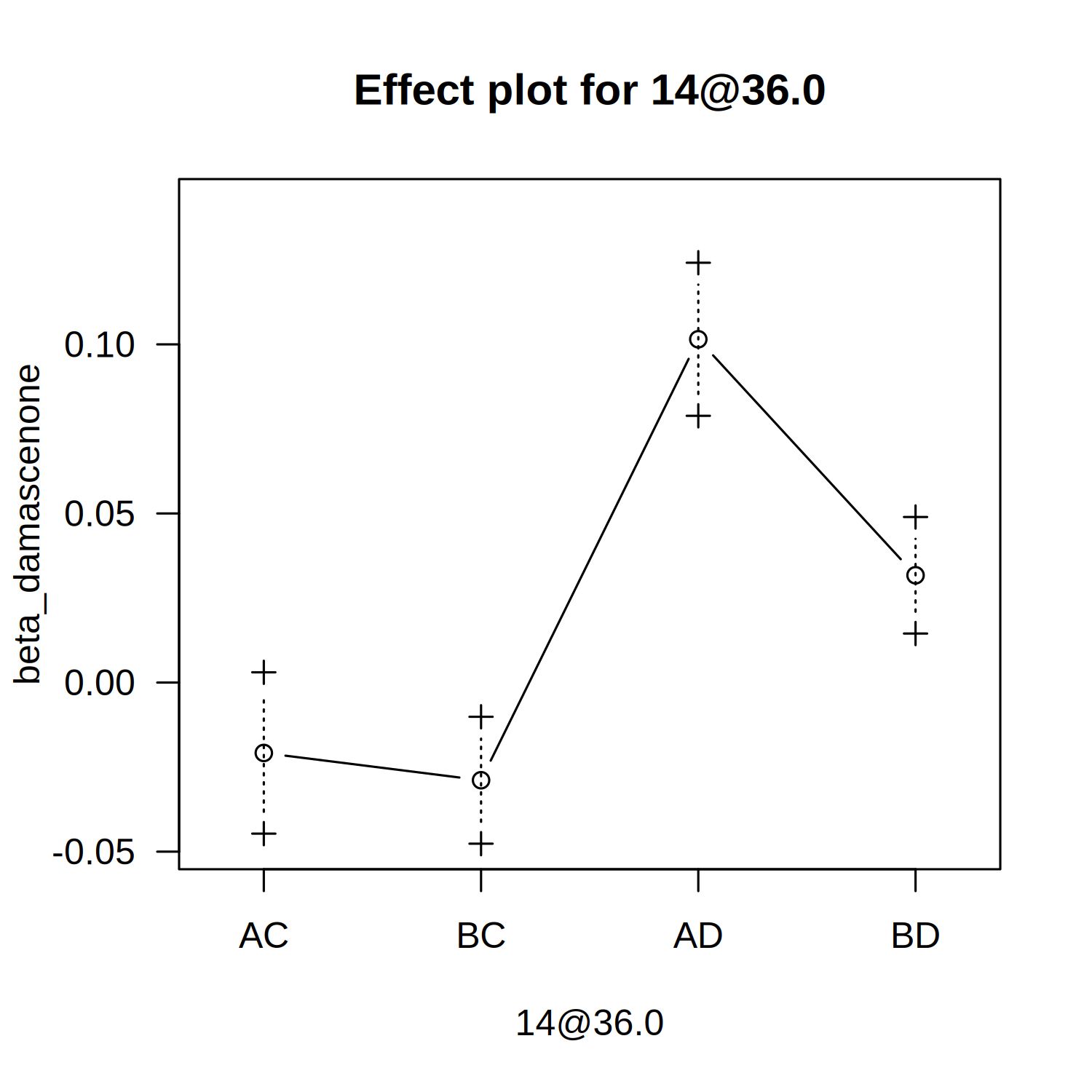

Supplement: Supplementary file 2 [file DataSheet2.zip › Supplementary_Files_4/QTL_analysis/beta_damascenone/beta_damascenone_eff_chr14.jpg]

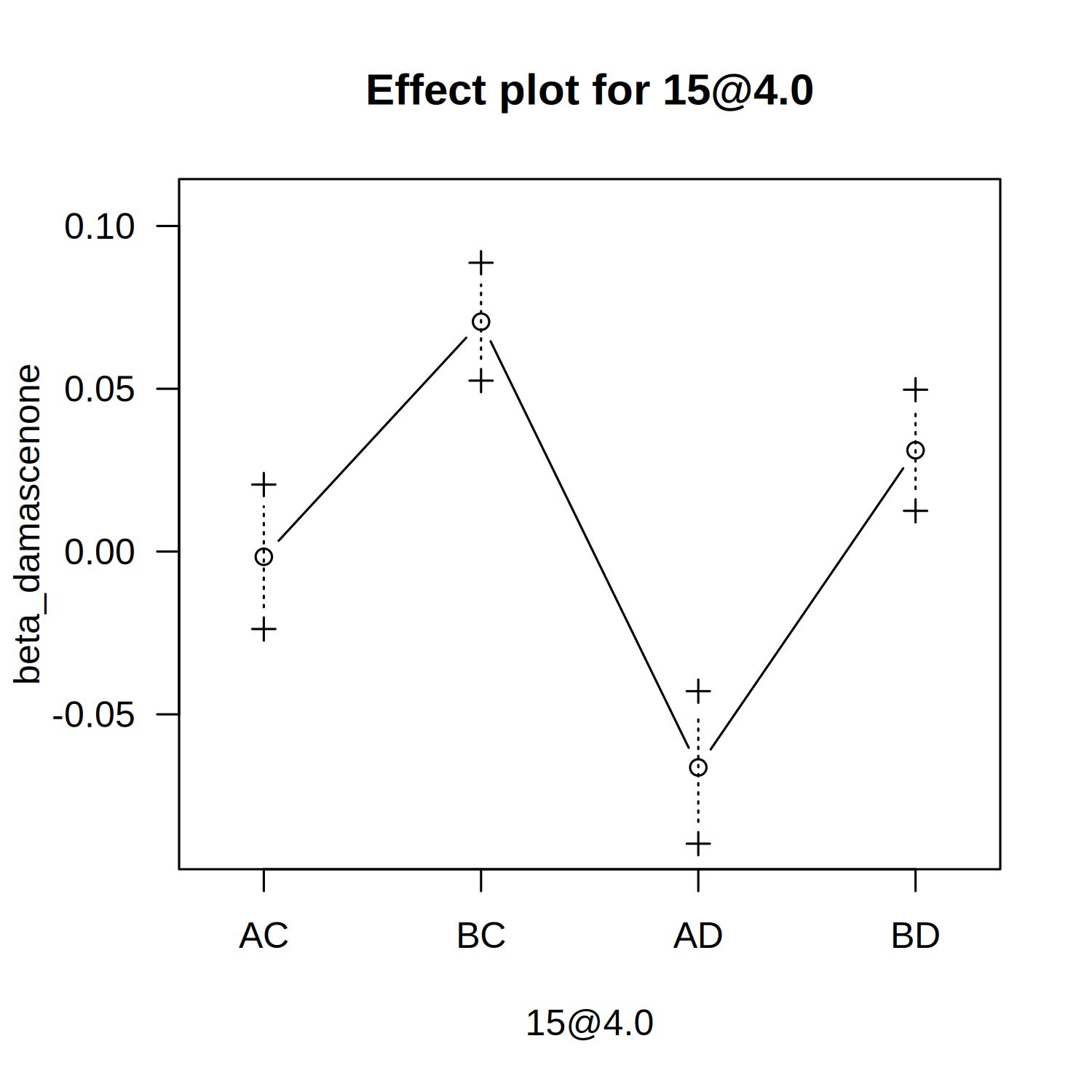

Supplement: Supplementary file 2 [file DataSheet2.zip › Supplementary_Files_4/QTL_analysis/beta_damascenone/beta_damascenone_eff_chr15.jpg]

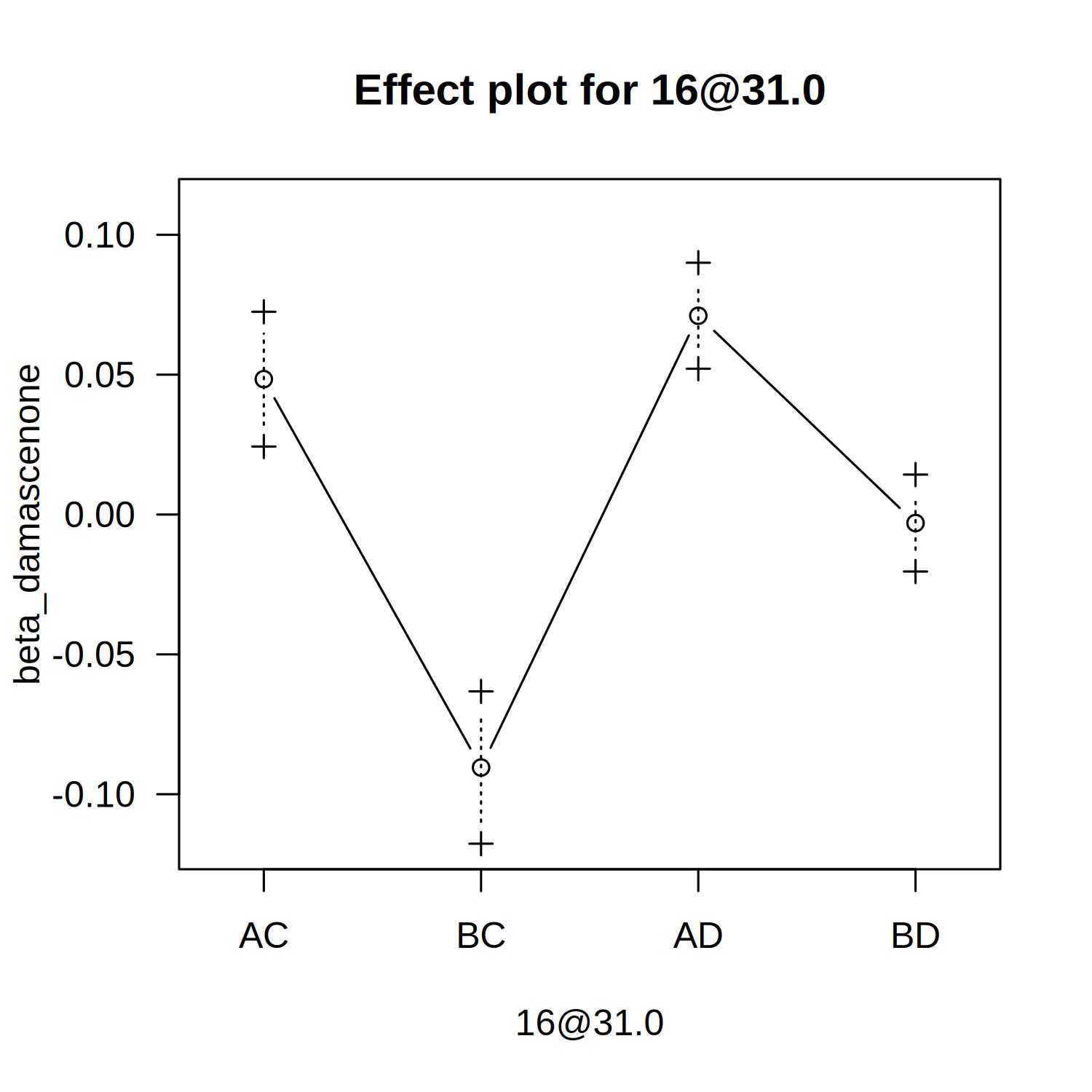

Supplement: Supplementary file 2 [file DataSheet2.zip › Supplementary_Files_4/QTL_analysis/beta_damascenone/beta_damascenone_eff_chr16.jpg]

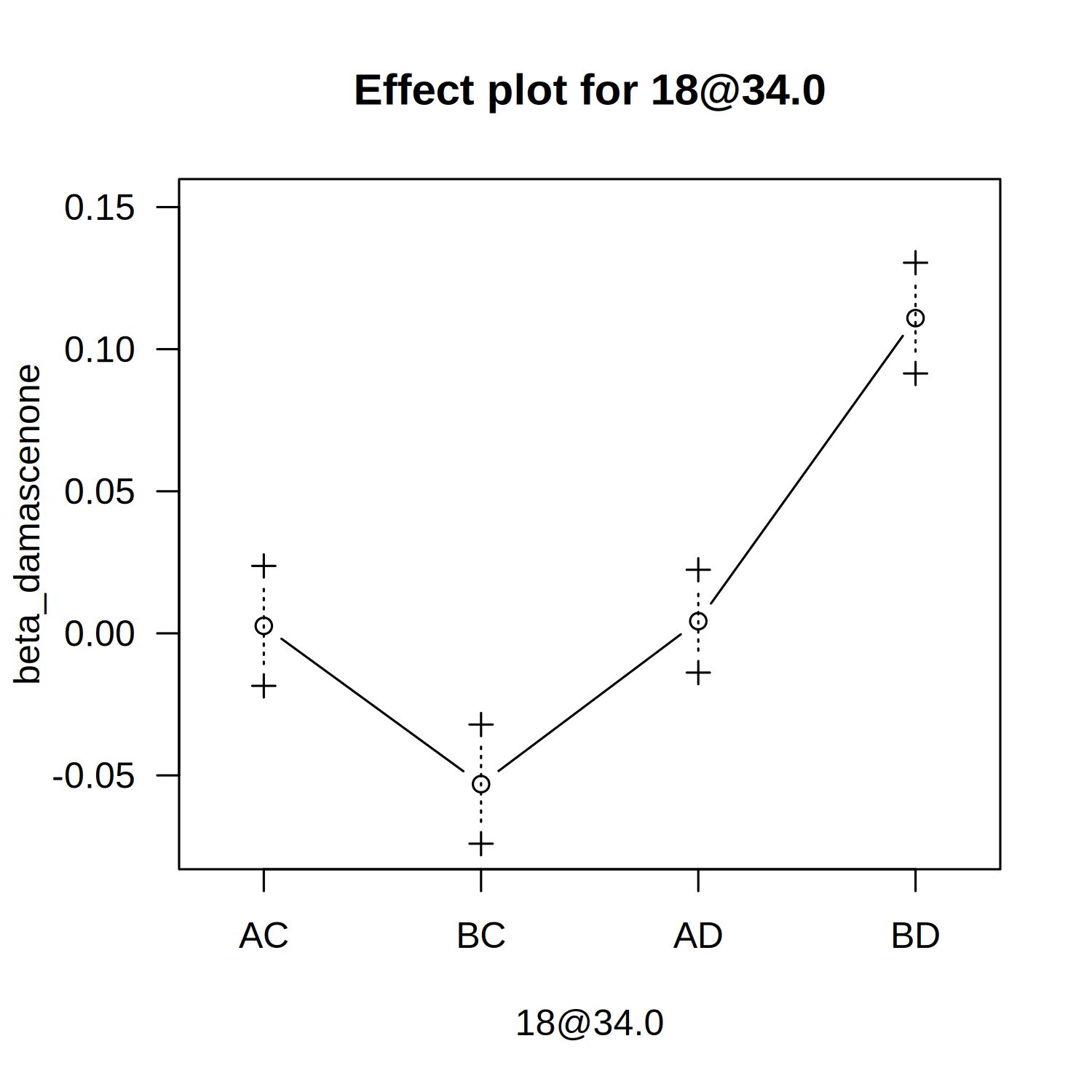

Supplement: Supplementary file 2 [file DataSheet2.zip › Supplementary_Files_4/QTL_analysis/beta_damascenone/beta_damascenone_eff_chr18.jpg]

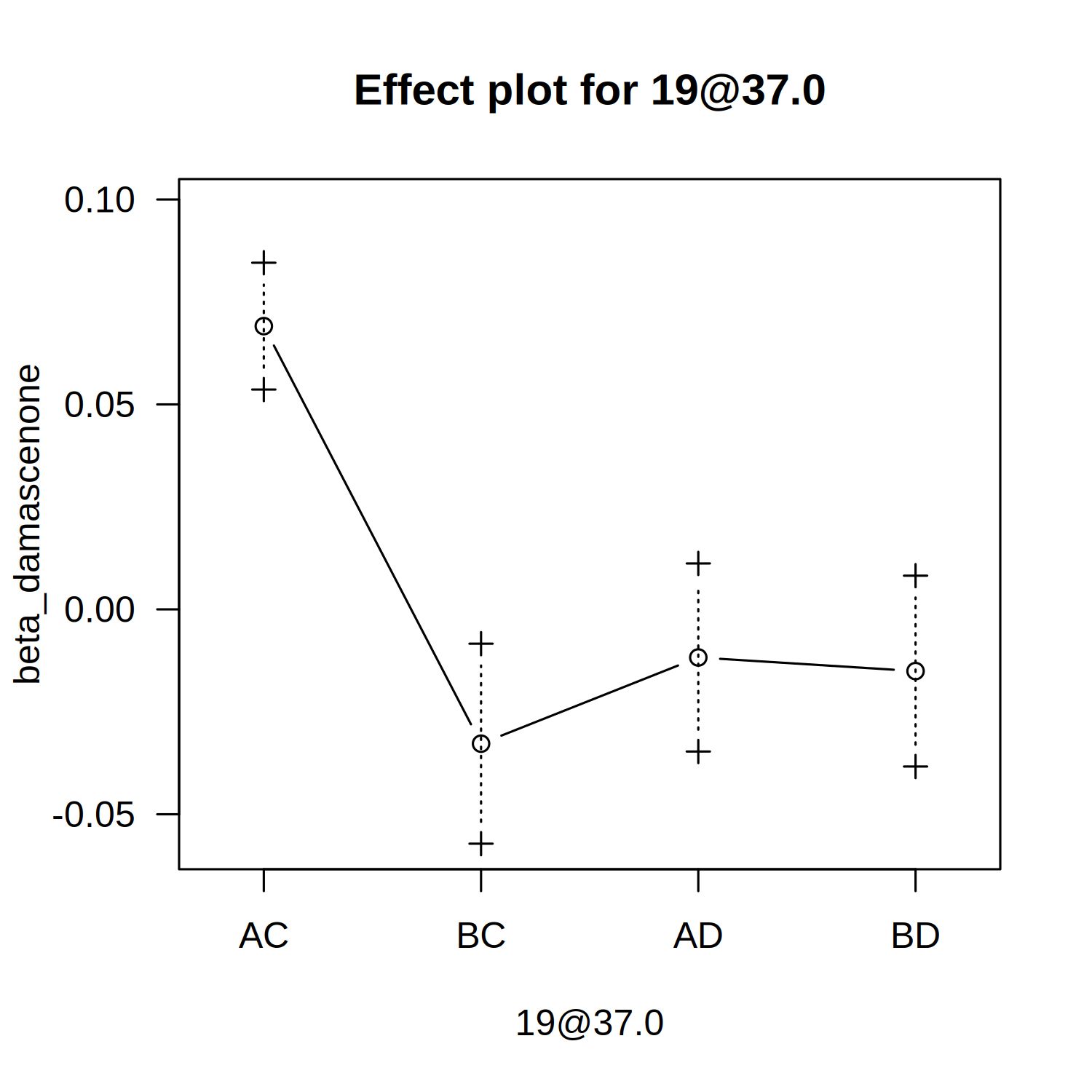

Supplement: Supplementary file 2 [file DataSheet2.zip › Supplementary_Files_4/QTL_analysis/beta_damascenone/beta_damascenone_eff_chr19.jpg]

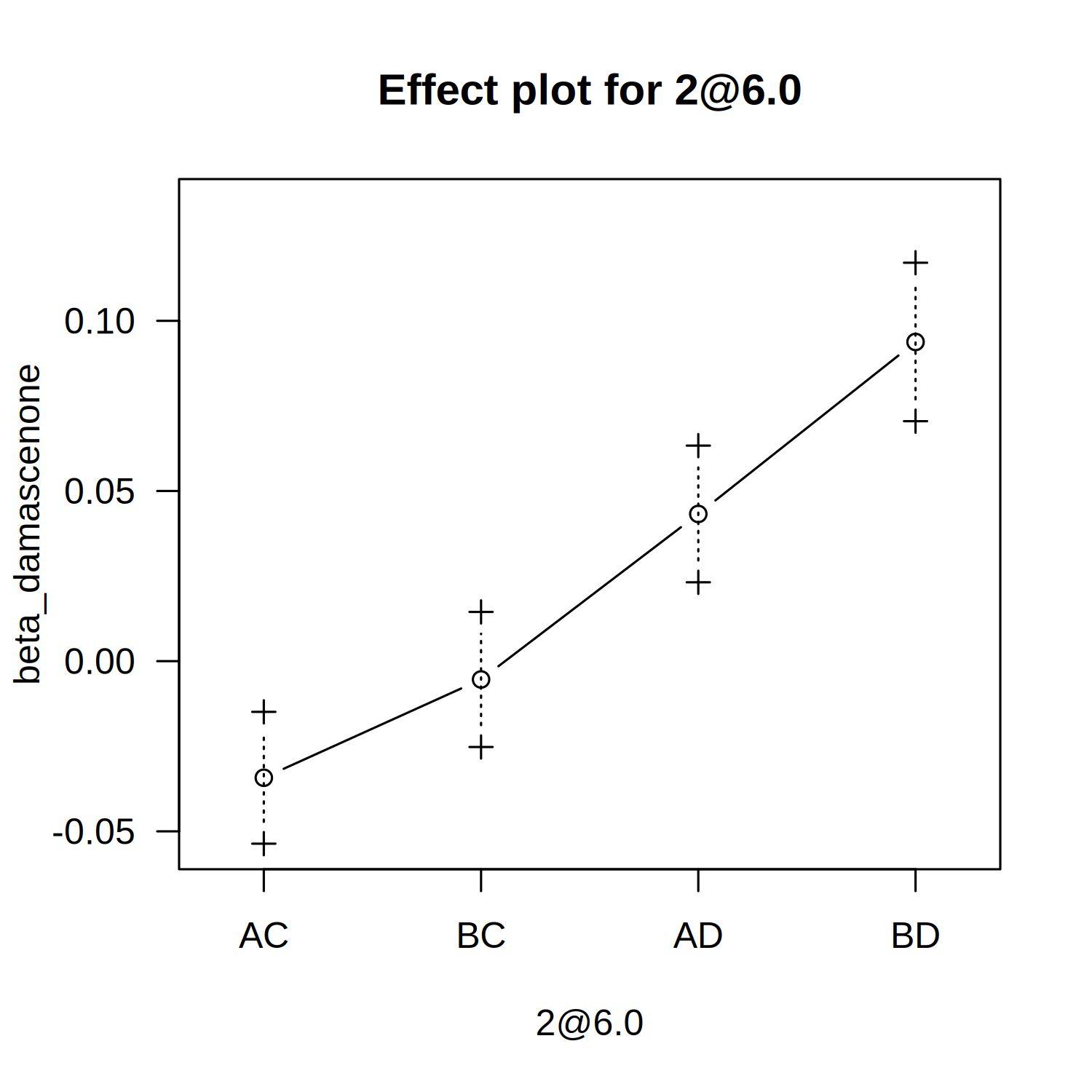

Supplement: Supplementary file 2 [file DataSheet2.zip › Supplementary_Files_4/QTL_analysis/beta_damascenone/beta_damascenone_eff_chr2.jpg]

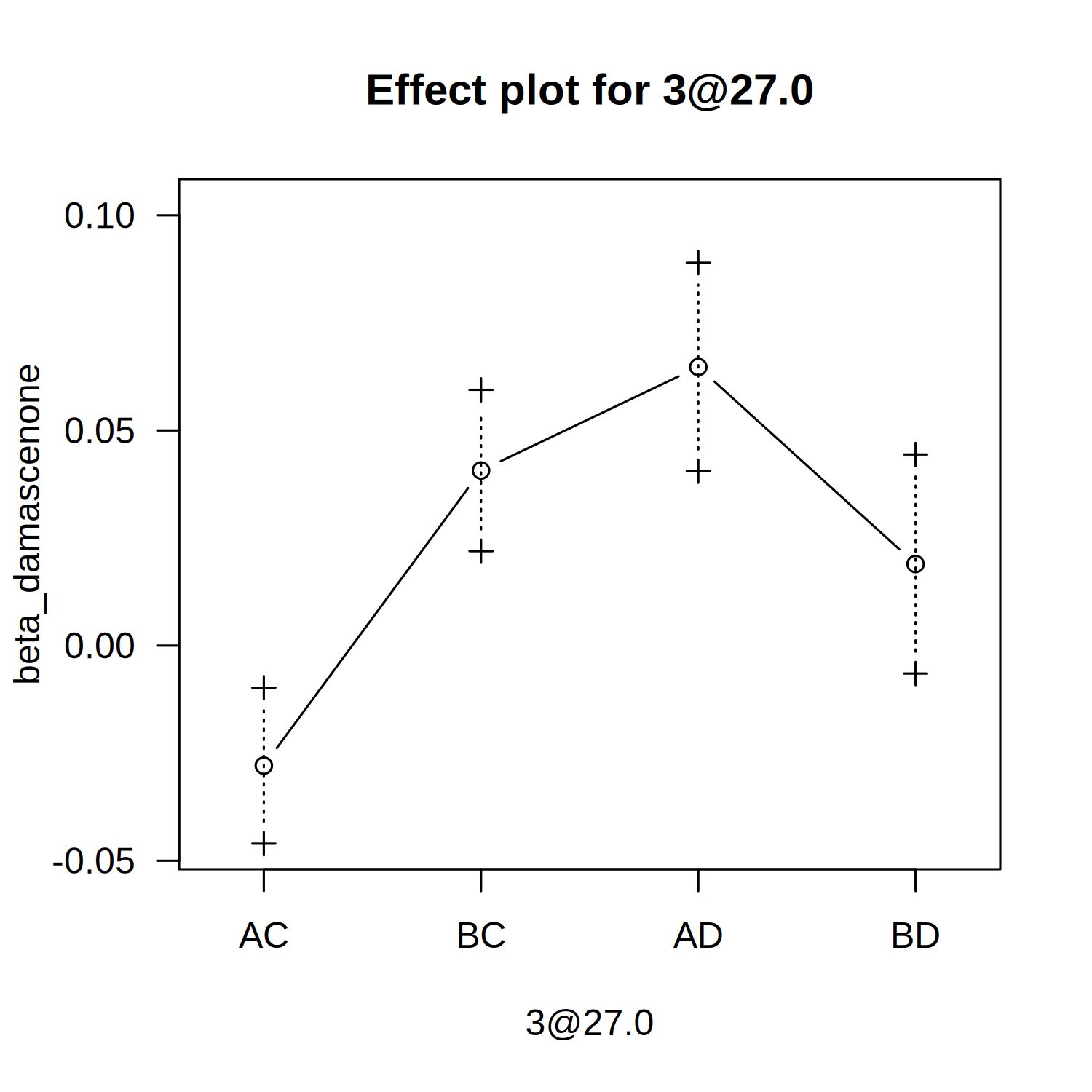

Supplement: Supplementary file 2 [file DataSheet2.zip › Supplementary_Files_4/QTL_analysis/beta_damascenone/beta_damascenone_eff_chr3.jpg]

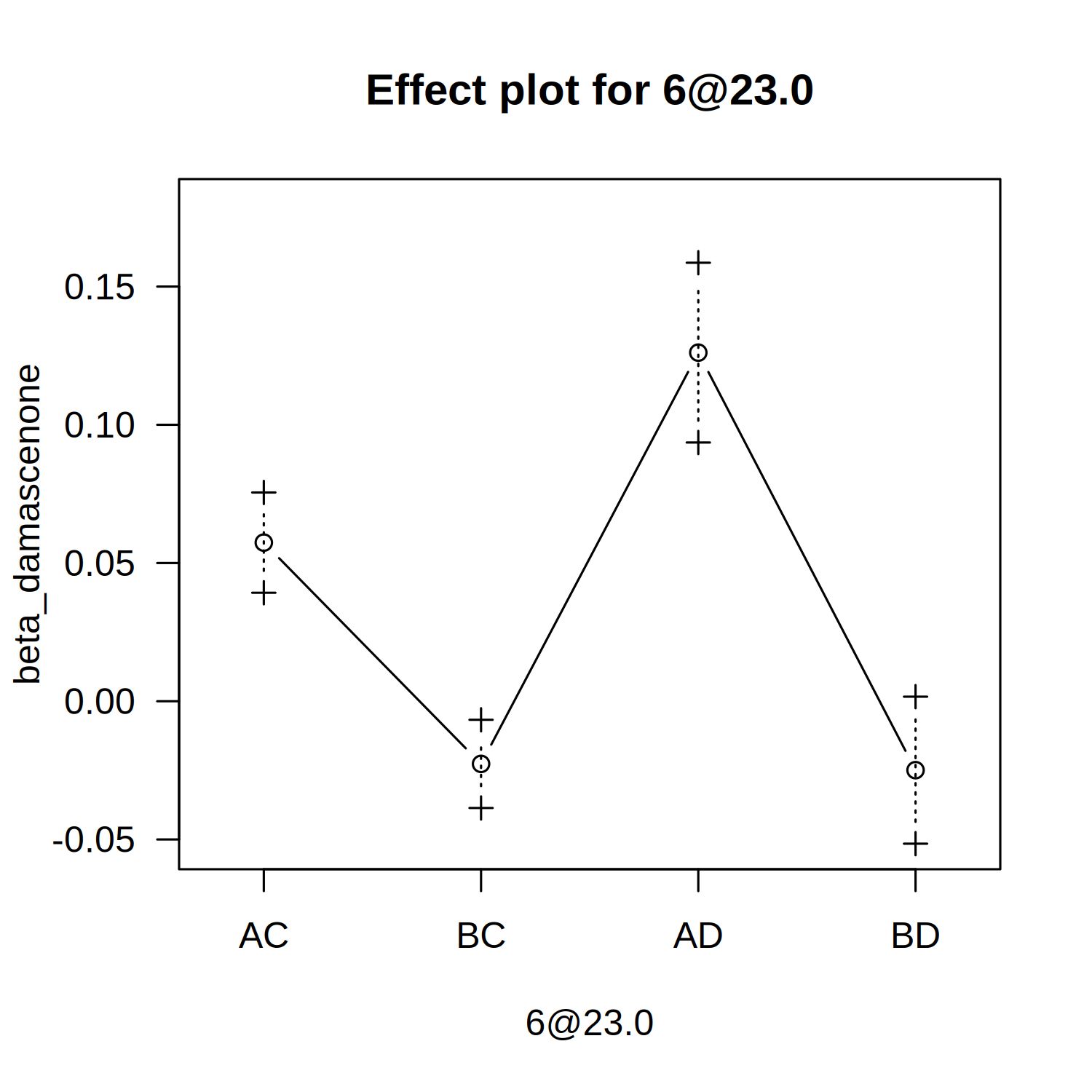

Supplement: Supplementary file 2 [file DataSheet2.zip › Supplementary_Files_4/QTL_analysis/beta_damascenone/beta_damascenone_eff_chr6.jpg]

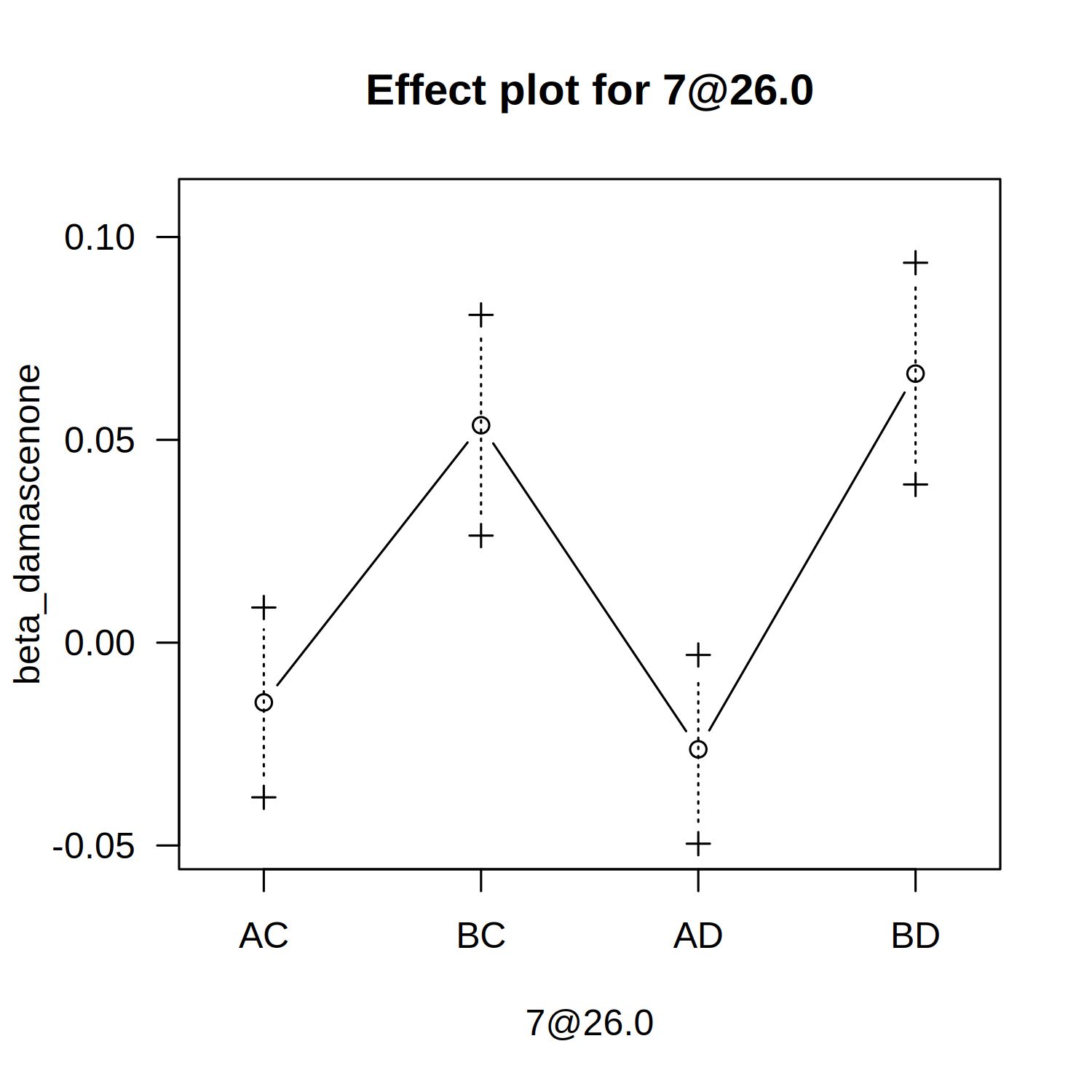

Supplement: Supplementary file 2 [file DataSheet2.zip › Supplementary_Files_4/QTL_analysis/beta_damascenone/beta_damascenone_eff_chr7.jpg]

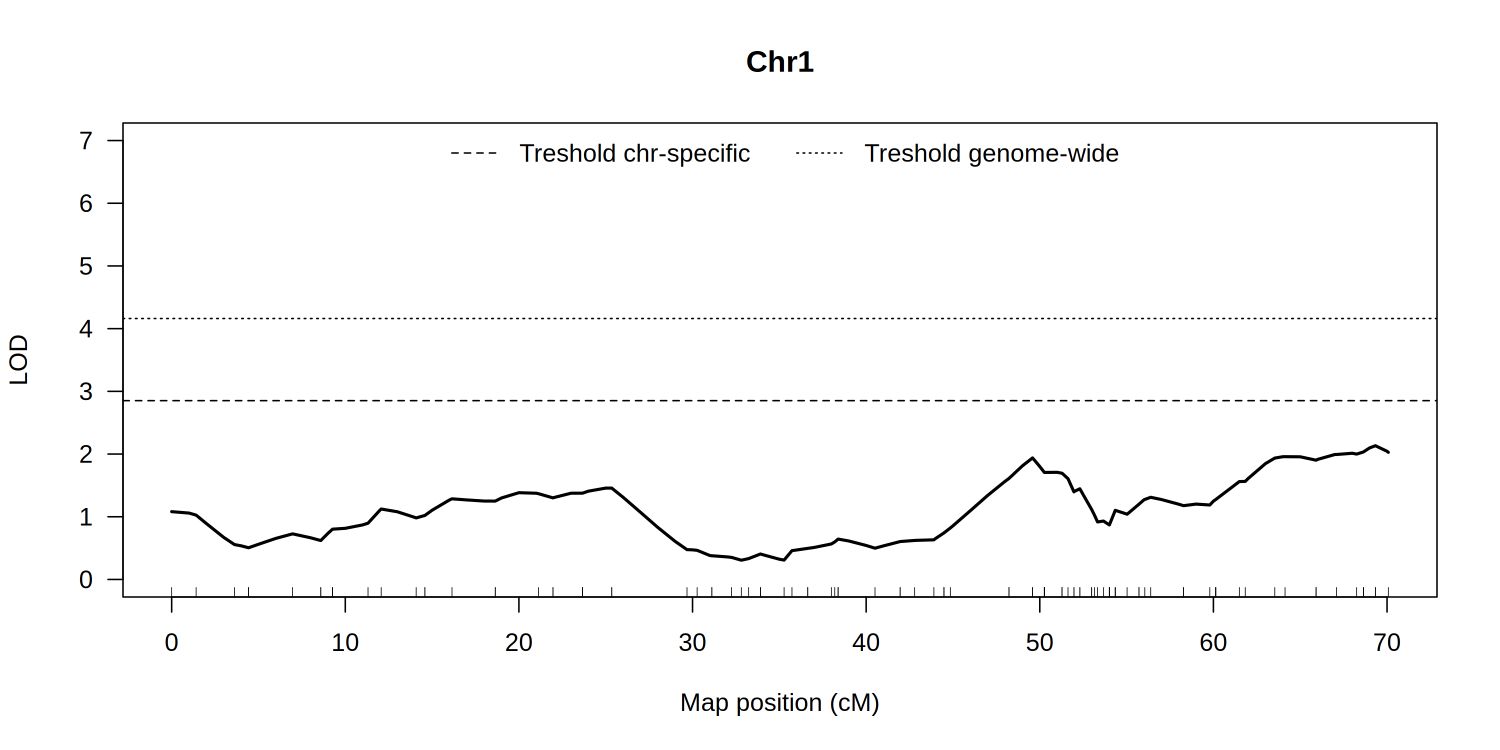

Supplement: Supplementary file 2 [file DataSheet2.zip › Supplementary_Files_4/QTL_analysis/beta_damascenone/beta_damascenone_LODplot_chr1.jpg]

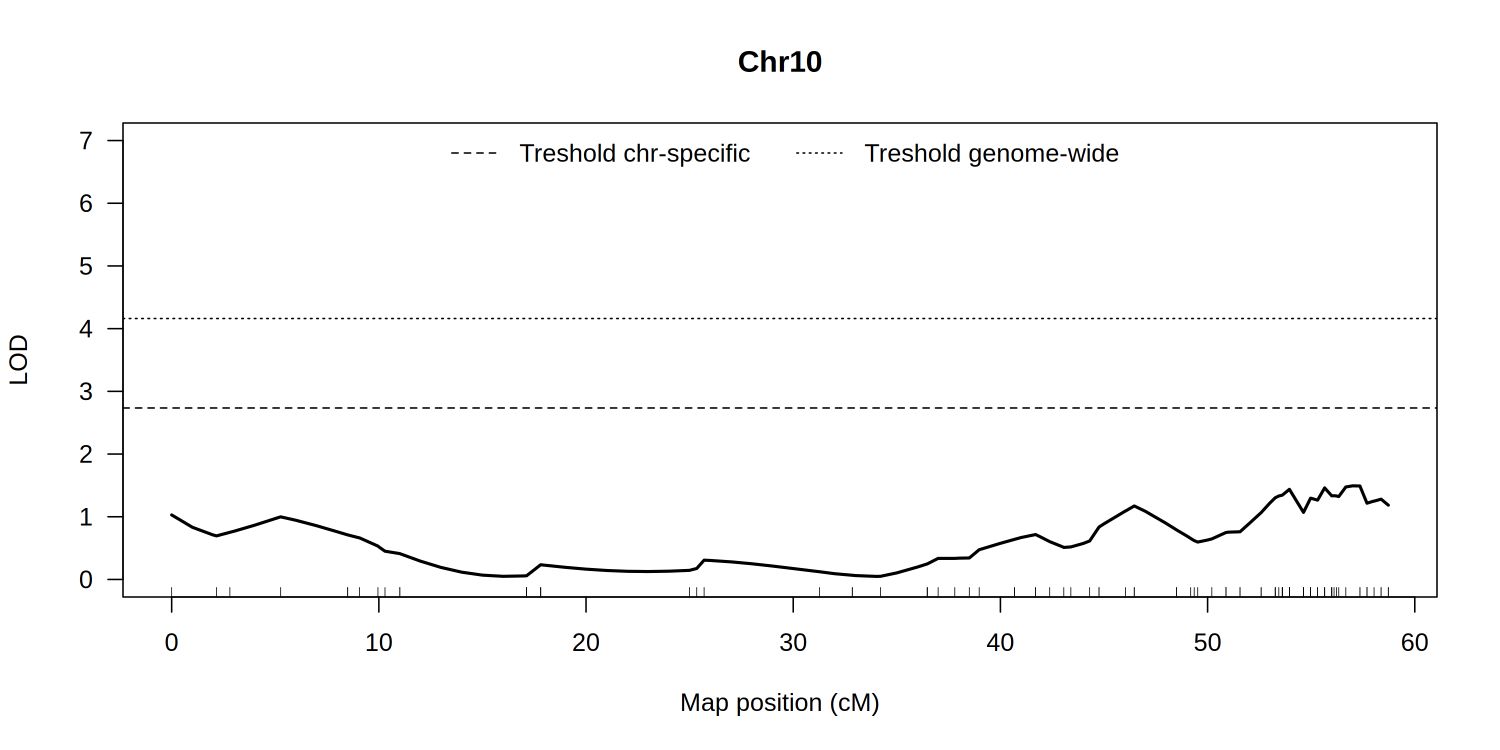

Supplement: Supplementary file 2 [file DataSheet2.zip › Supplementary_Files_4/QTL_analysis/beta_damascenone/beta_damascenone_LODplot_chr10.jpg]

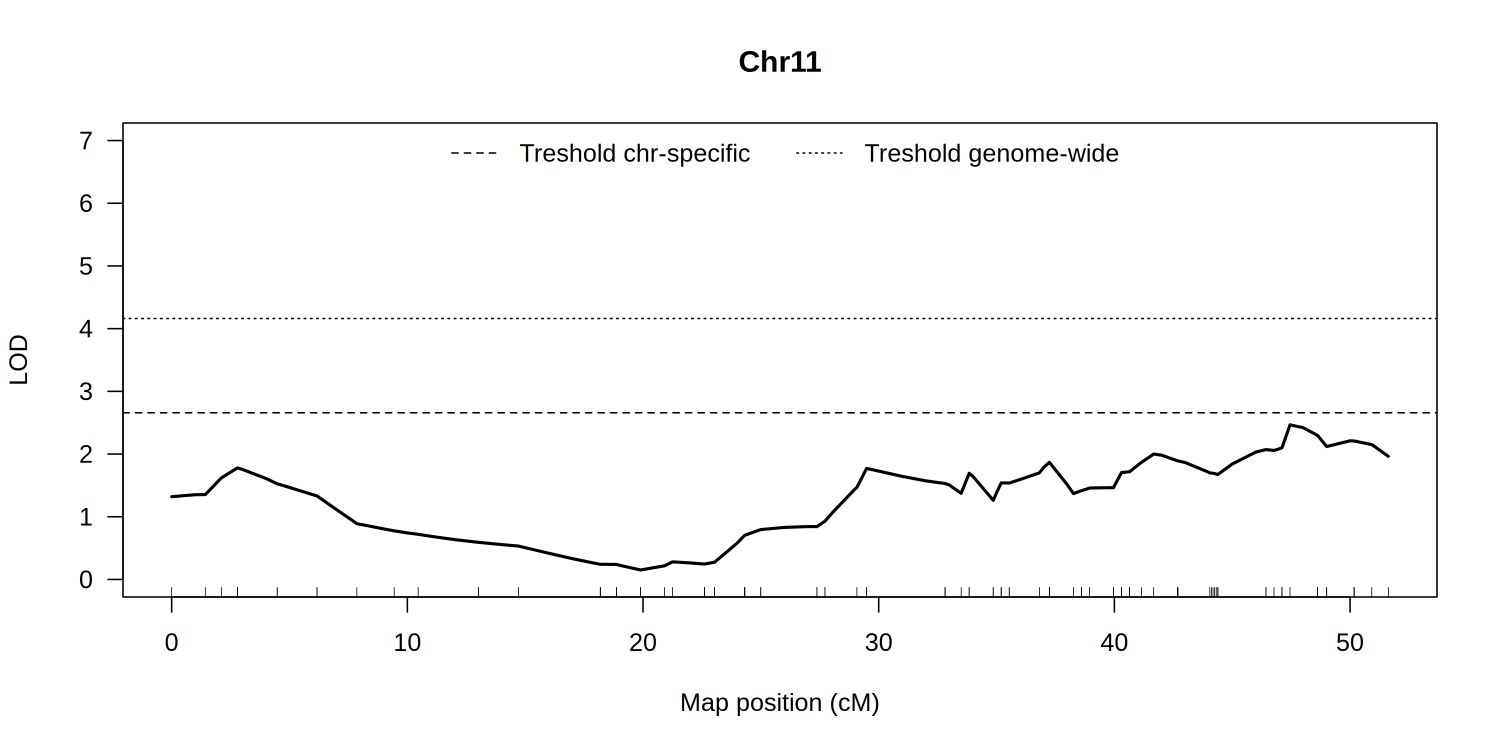

Supplement: Supplementary file 2 [file DataSheet2.zip › Supplementary_Files_4/QTL_analysis/beta_damascenone/beta_damascenone_LODplot_chr11.jpg]

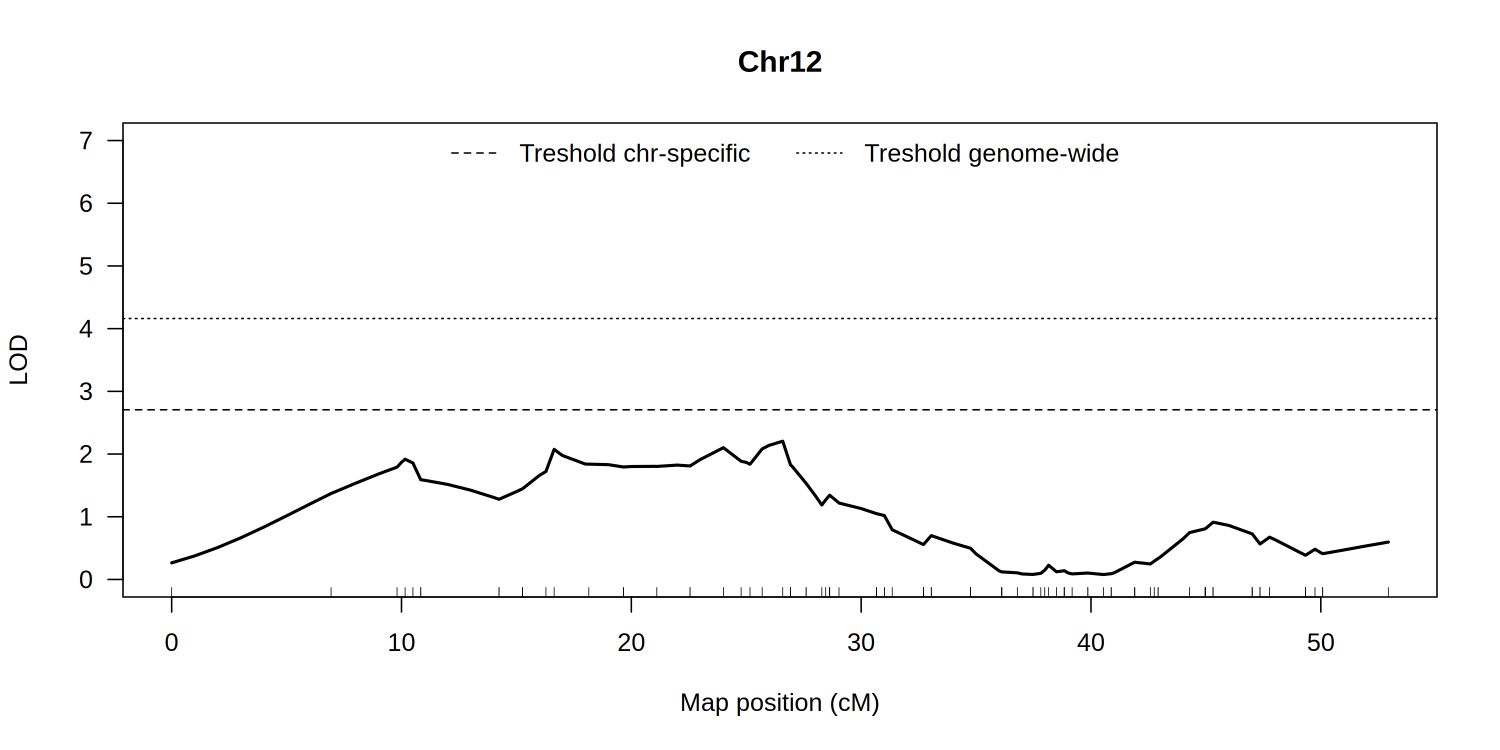

Supplement: Supplementary file 2 [file DataSheet2.zip › Supplementary_Files_4/QTL_analysis/beta_damascenone/beta_damascenone_LODplot_chr12.jpg]

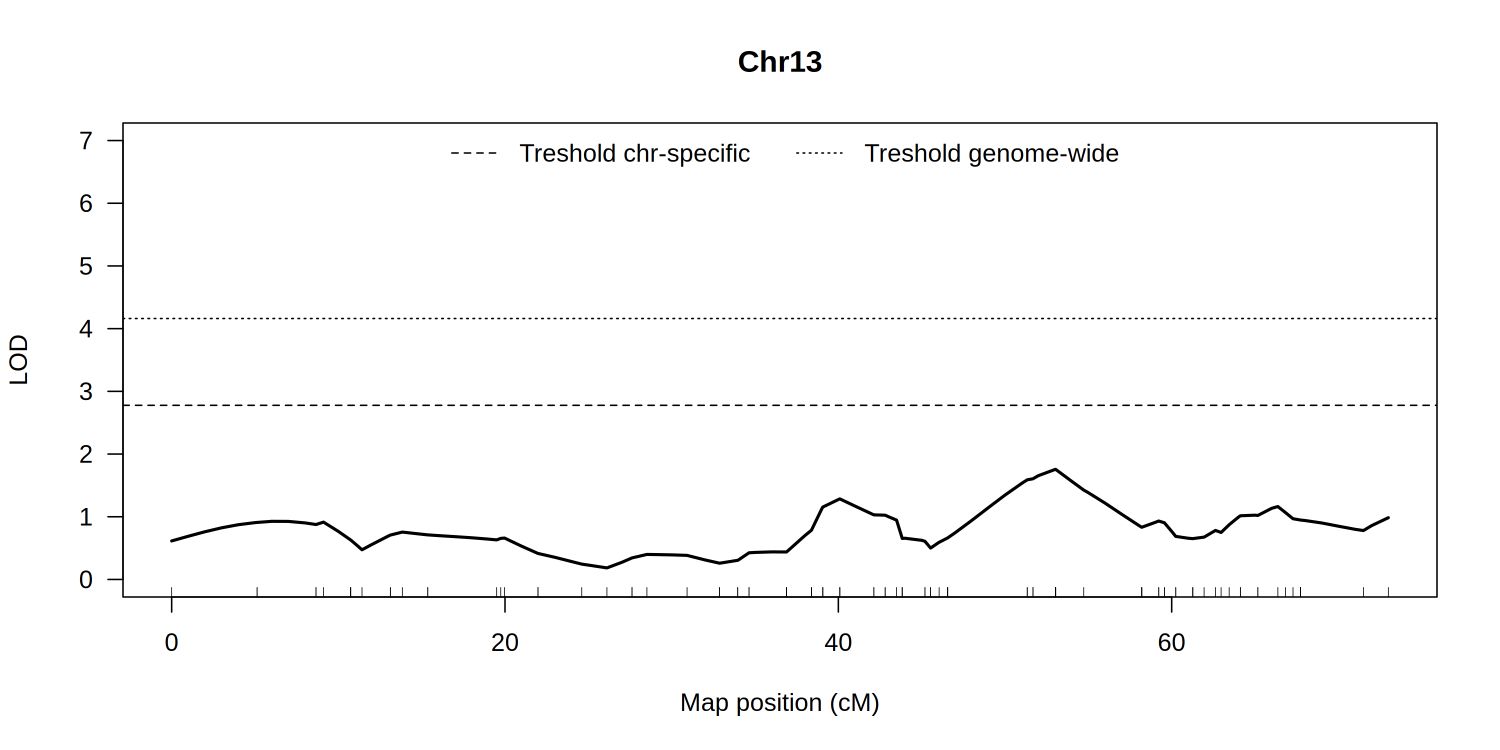

Supplement: Supplementary file 2 [file DataSheet2.zip › Supplementary_Files_4/QTL_analysis/beta_damascenone/beta_damascenone_LODplot_chr13.jpg]

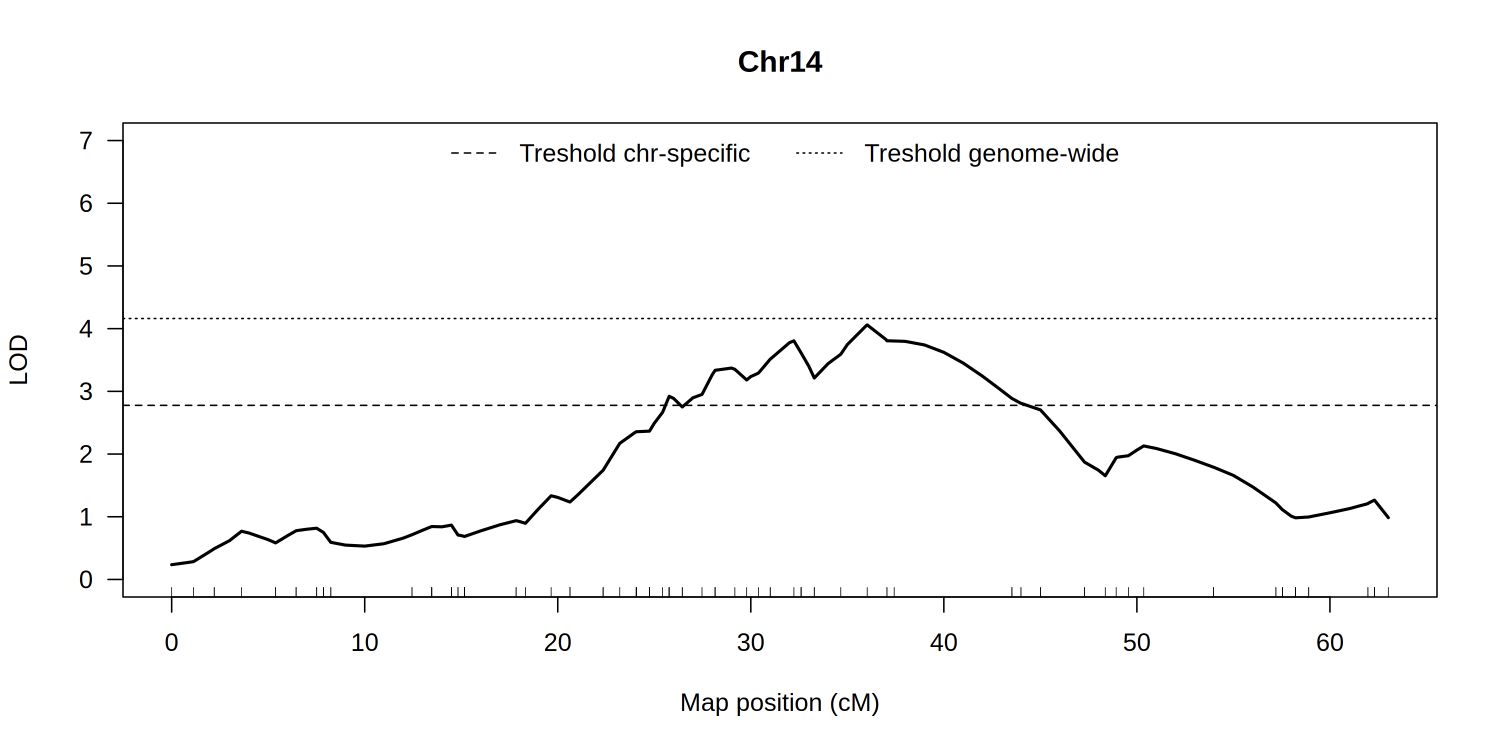

Supplement: Supplementary file 2 [file DataSheet2.zip › Supplementary_Files_4/QTL_analysis/beta_damascenone/beta_damascenone_LODplot_chr14.jpg]

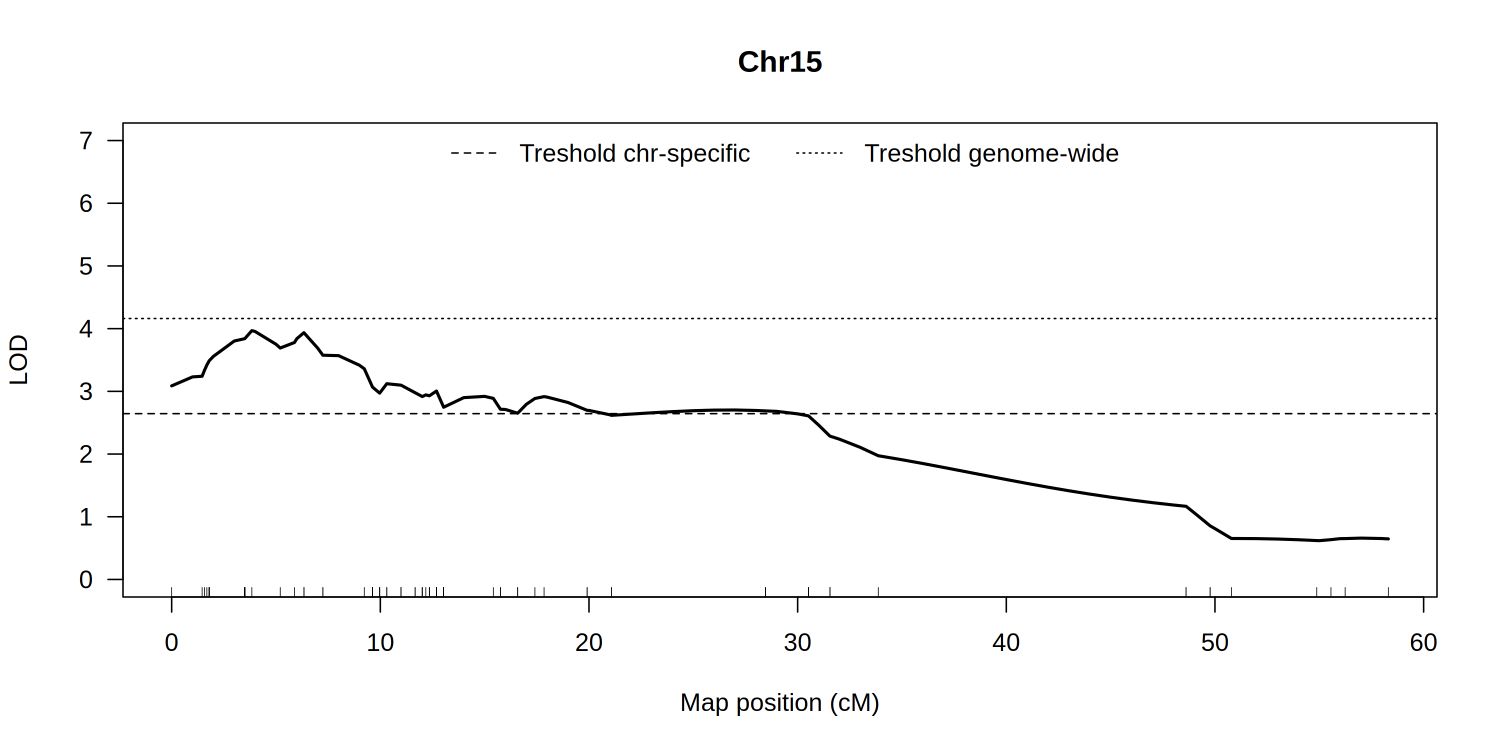

Supplement: Supplementary file 2 [file DataSheet2.zip › Supplementary_Files_4/QTL_analysis/beta_damascenone/beta_damascenone_LODplot_chr15.jpg]

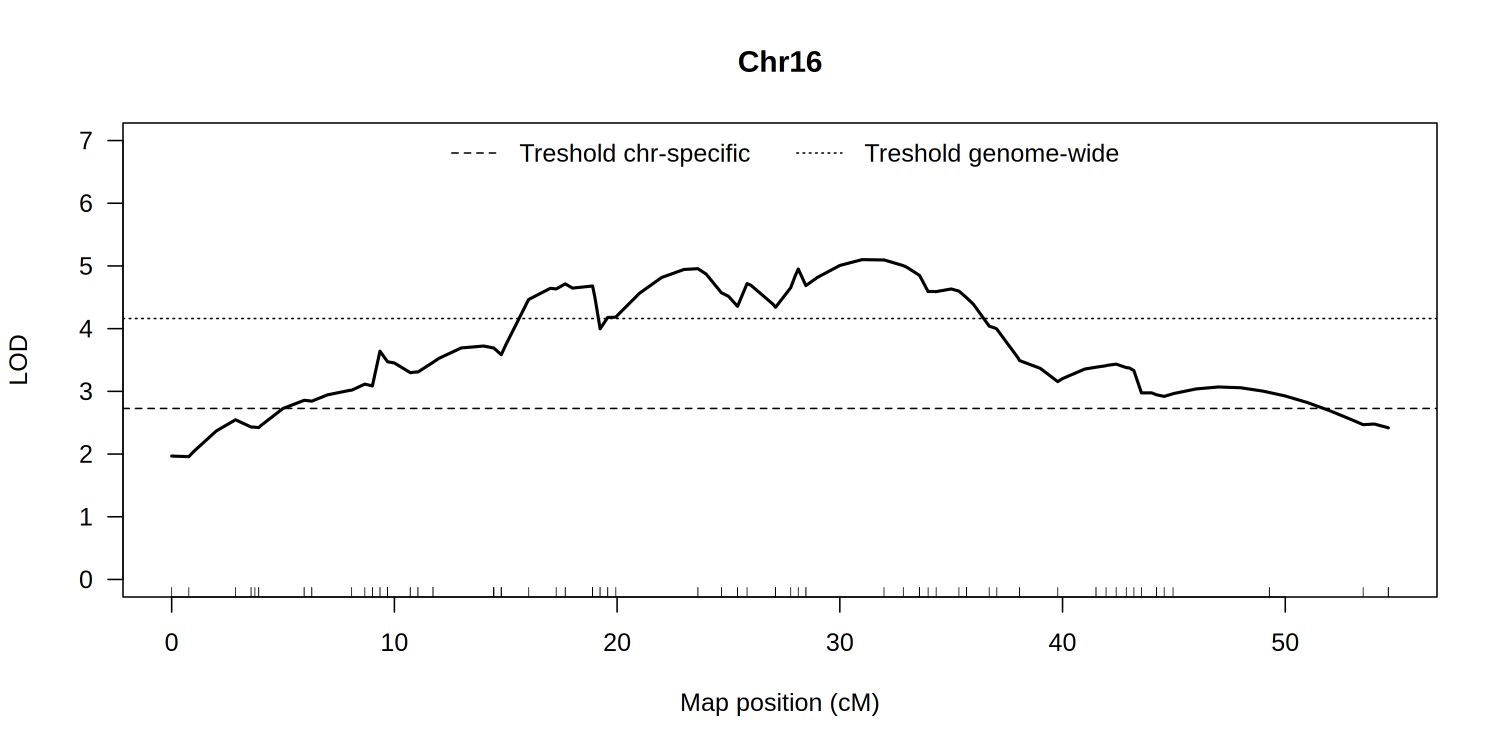

Supplement: Supplementary file 2 [file DataSheet2.zip › Supplementary_Files_4/QTL_analysis/beta_damascenone/beta_damascenone_LODplot_chr16.jpg]

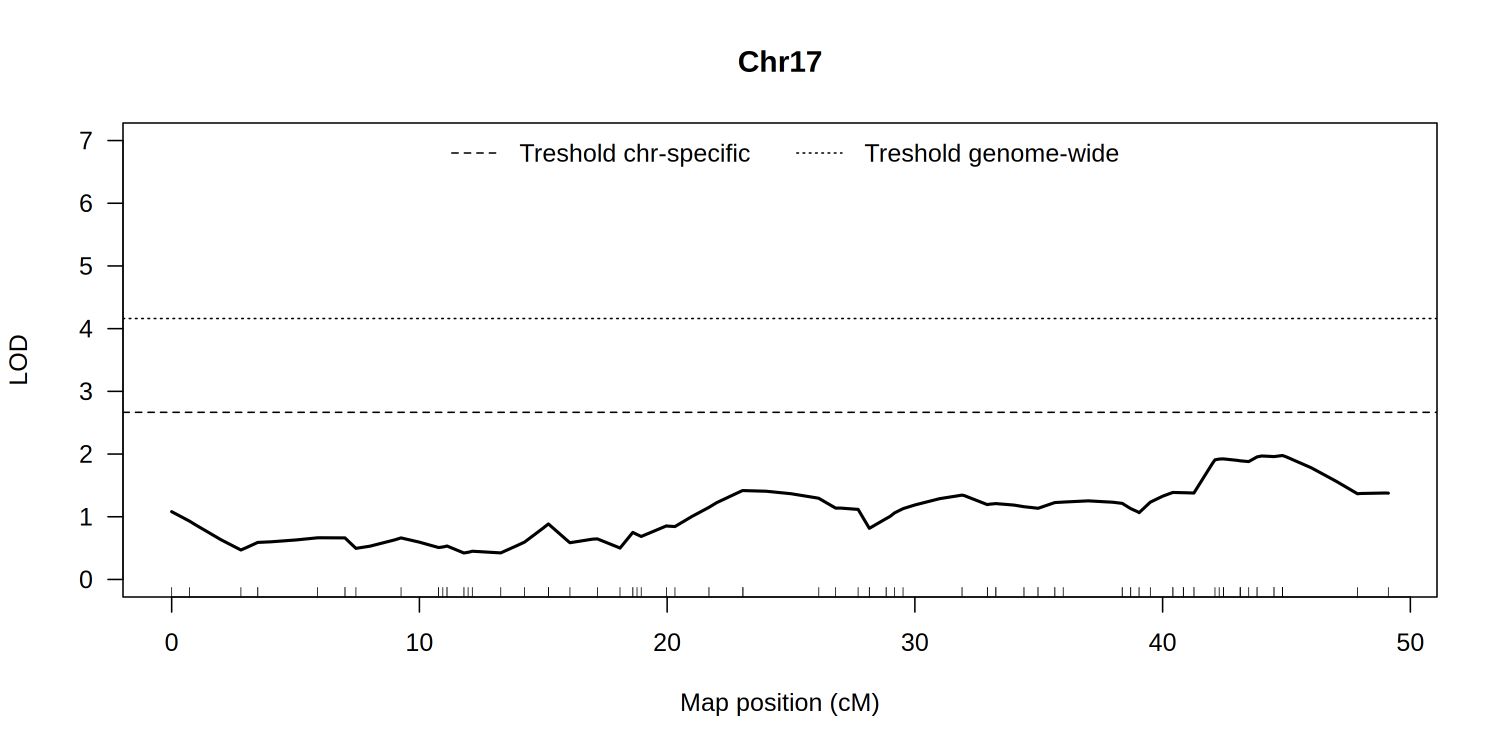

Supplement: Supplementary file 2 [file DataSheet2.zip › Supplementary_Files_4/QTL_analysis/beta_damascenone/beta_damascenone_LODplot_chr17.jpg]

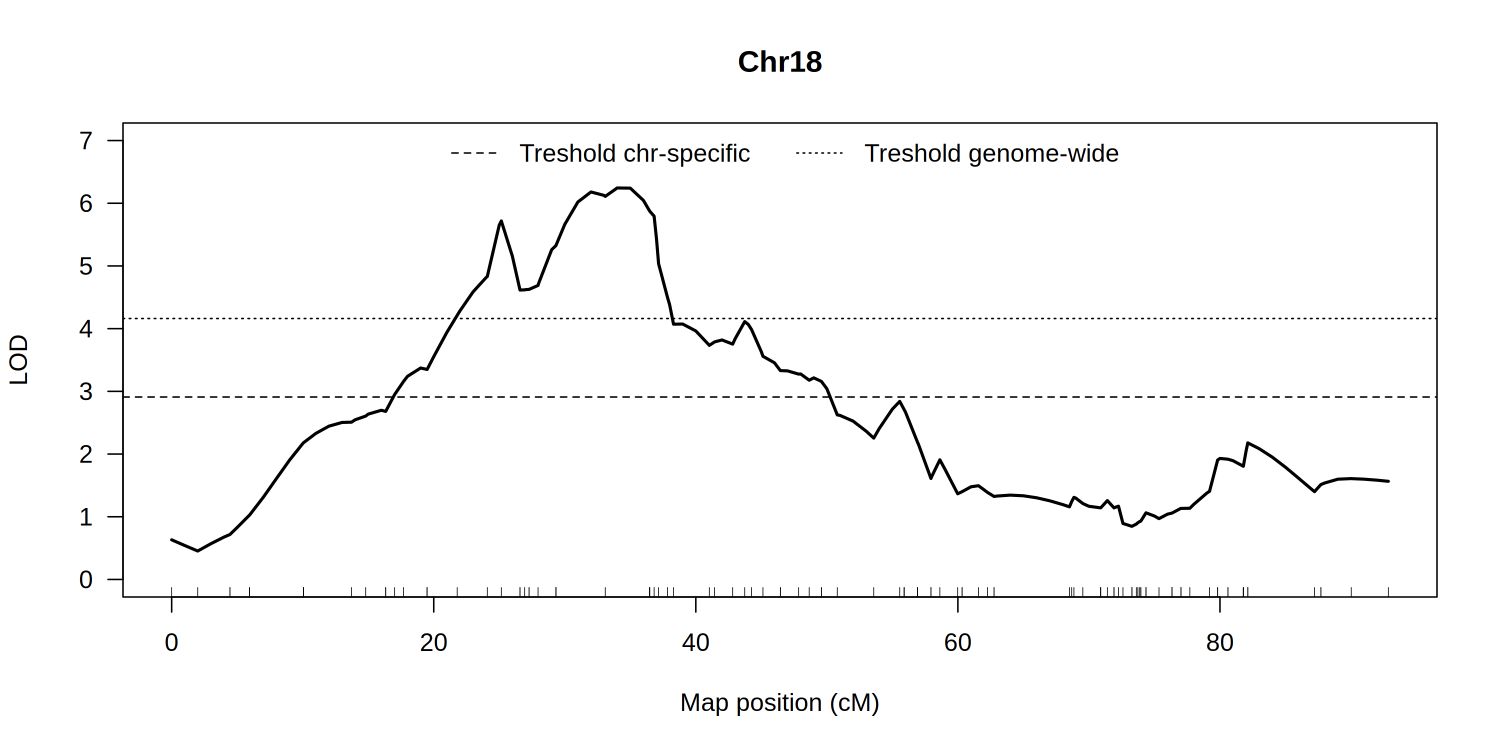

Supplement: Supplementary file 2 [file DataSheet2.zip › Supplementary_Files_4/QTL_analysis/beta_damascenone/beta_damascenone_LODplot_chr18.jpg]

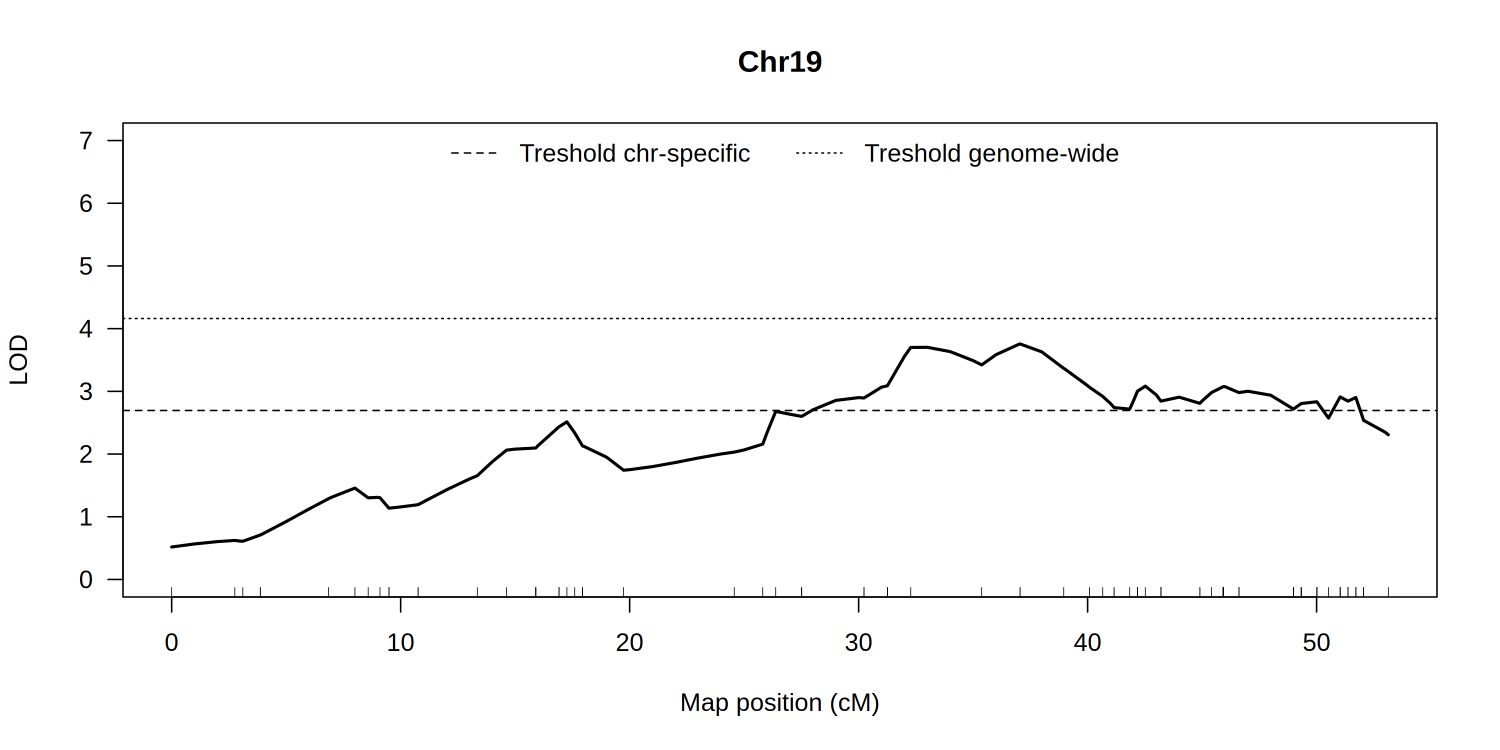

Supplement: Supplementary file 2 [file DataSheet2.zip › Supplementary_Files_4/QTL_analysis/beta_damascenone/beta_damascenone_LODplot_chr19.jpg]

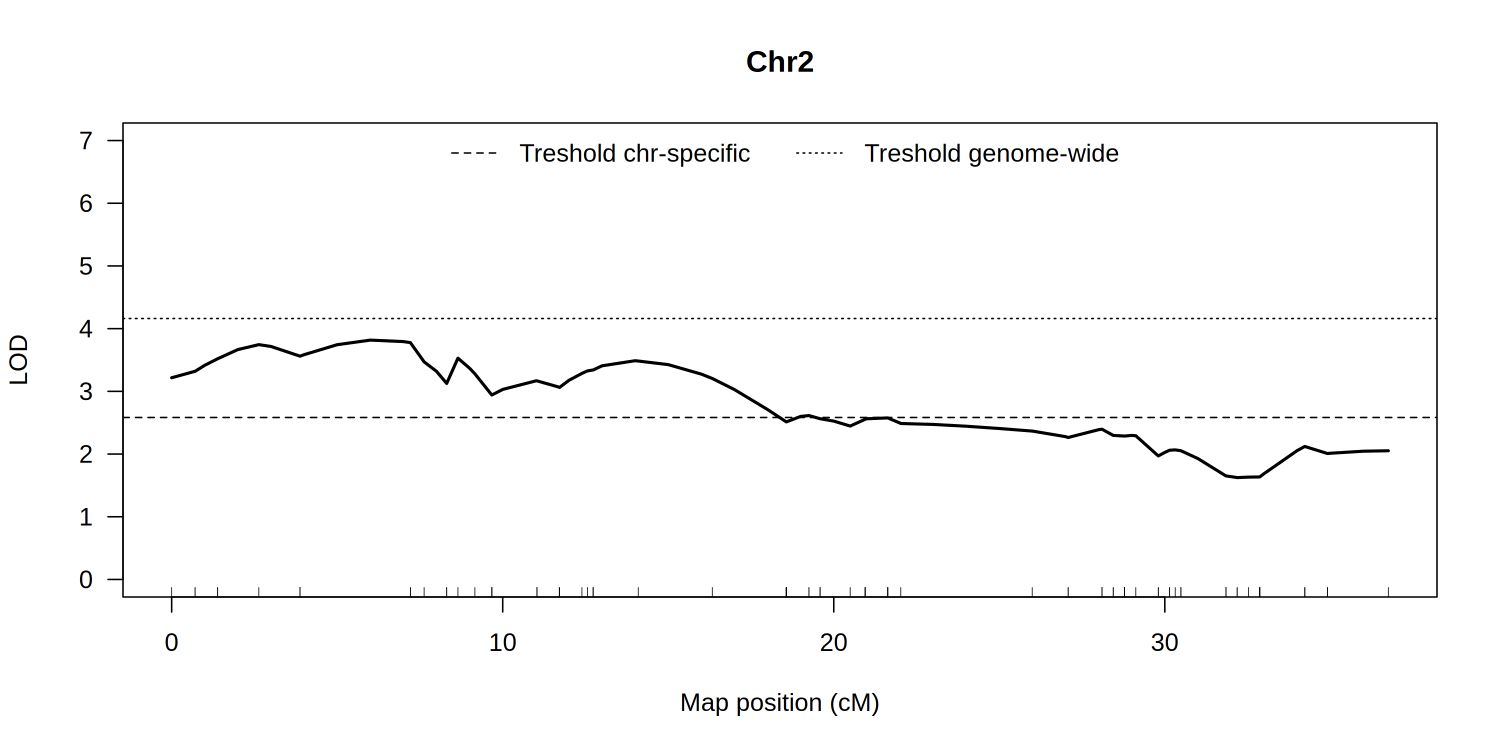

Supplement: Supplementary file 2 [file DataSheet2.zip › Supplementary_Files_4/QTL_analysis/beta_damascenone/beta_damascenone_LODplot_chr2.jpg]

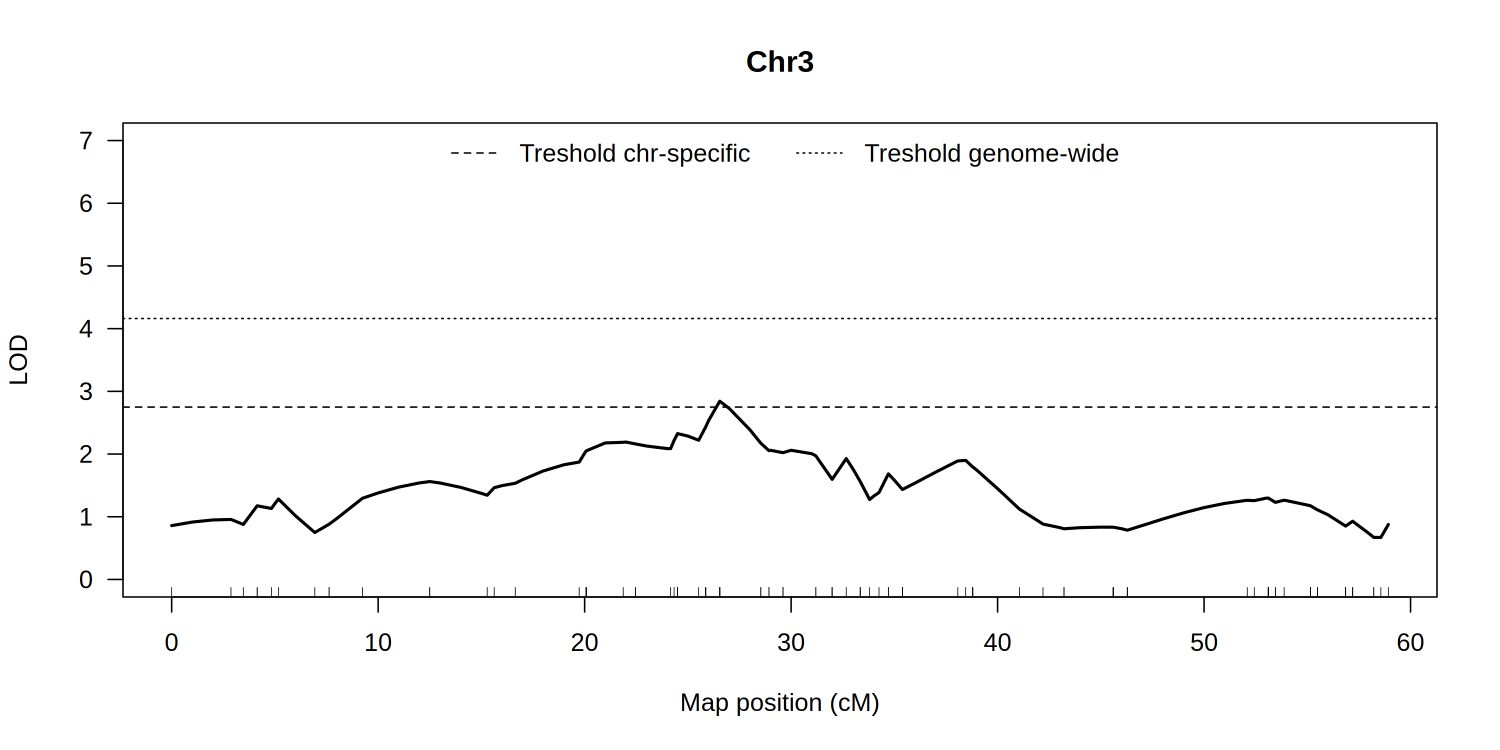

Supplement: Supplementary file 2 [file DataSheet2.zip › Supplementary_Files_4/QTL_analysis/beta_damascenone/beta_damascenone_LODplot_chr3.jpg]

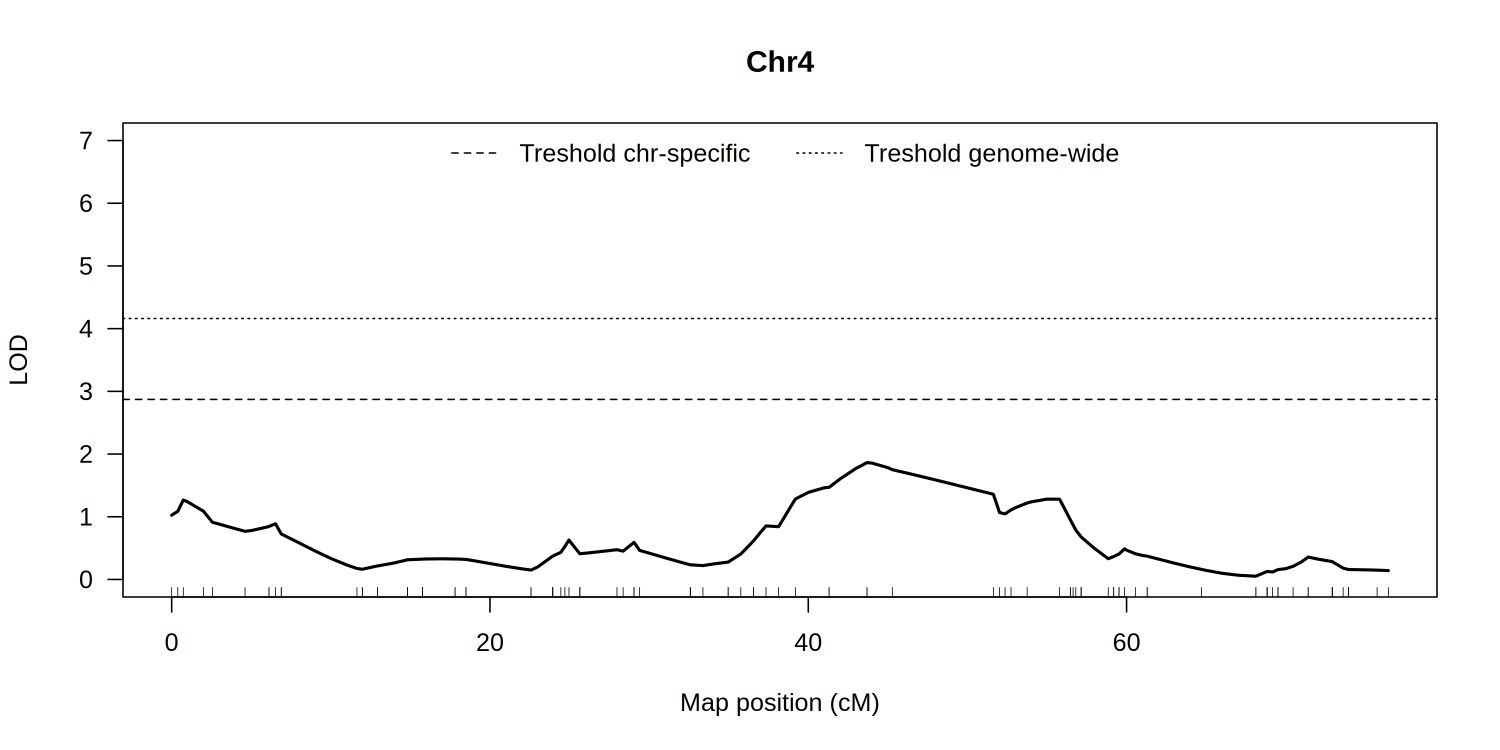

Supplement: Supplementary file 2 [file DataSheet2.zip › Supplementary_Files_4/QTL_analysis/beta_damascenone/beta_damascenone_LODplot_chr4.jpg]

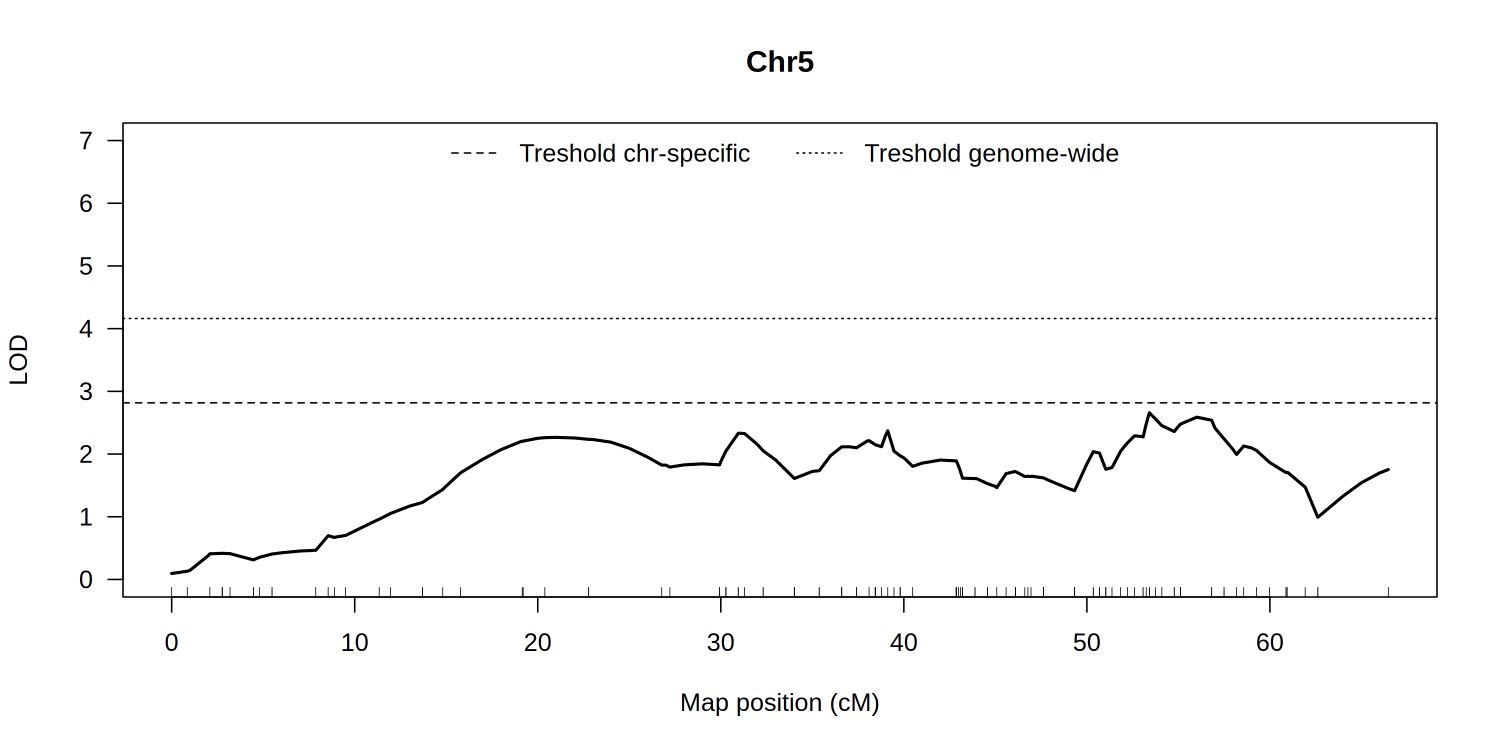

Supplement: Supplementary file 2 [file DataSheet2.zip › Supplementary_Files_4/QTL_analysis/beta_damascenone/beta_damascenone_LODplot_chr5.jpg]

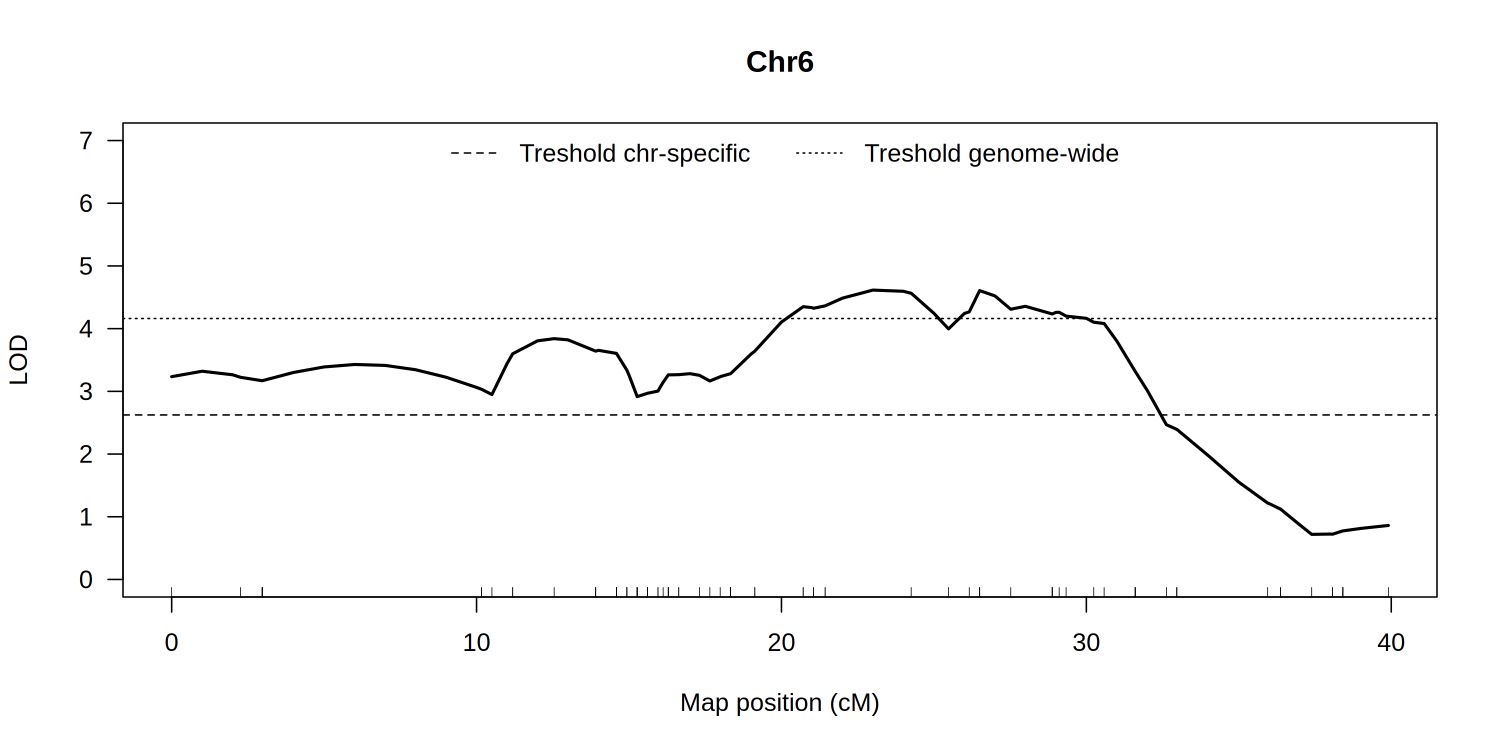

Supplement: Supplementary file 2 [file DataSheet2.zip › Supplementary_Files_4/QTL_analysis/beta_damascenone/beta_damascenone_LODplot_chr6.jpg]

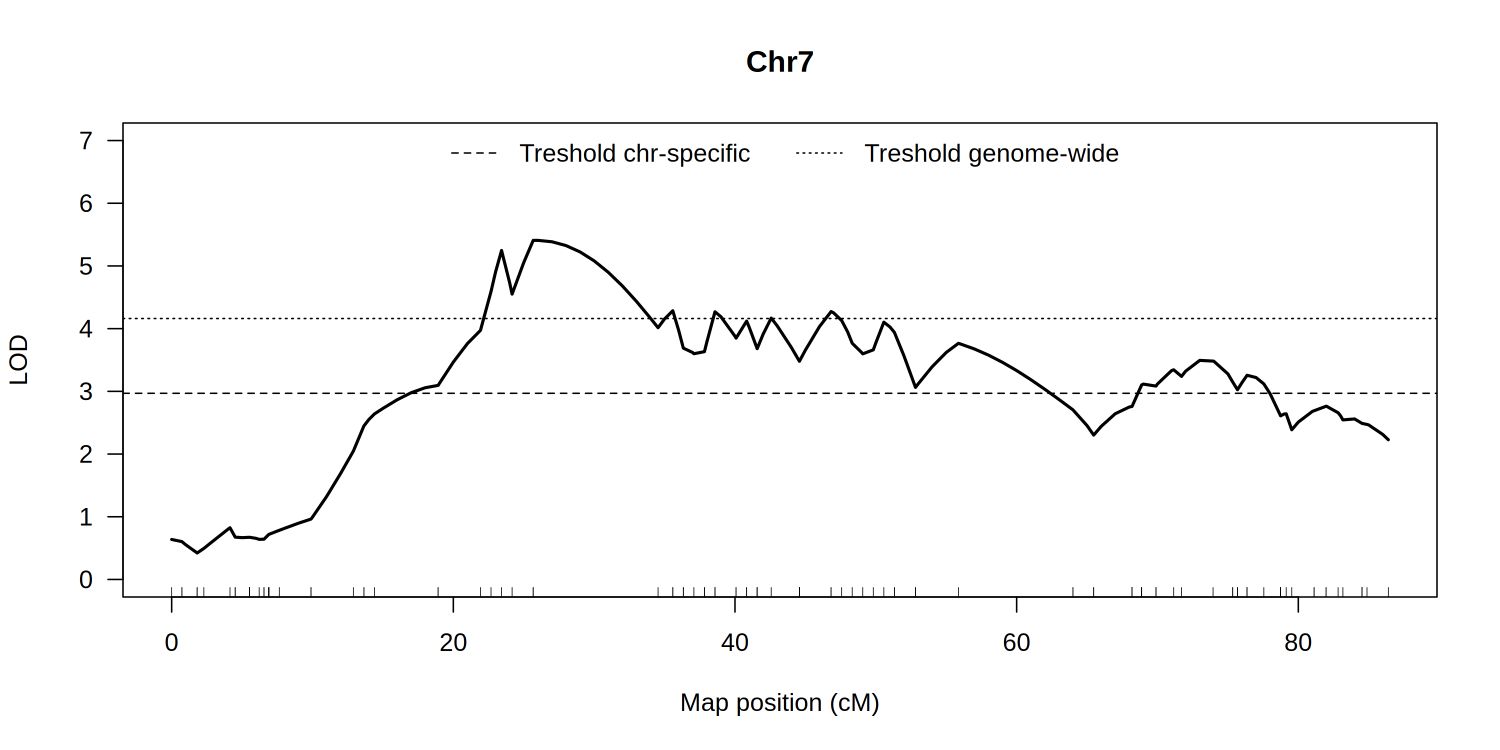

Supplement: Supplementary file 2 [file DataSheet2.zip › Supplementary_Files_4/QTL_analysis/beta_damascenone/beta_damascenone_LODplot_chr7.jpg]

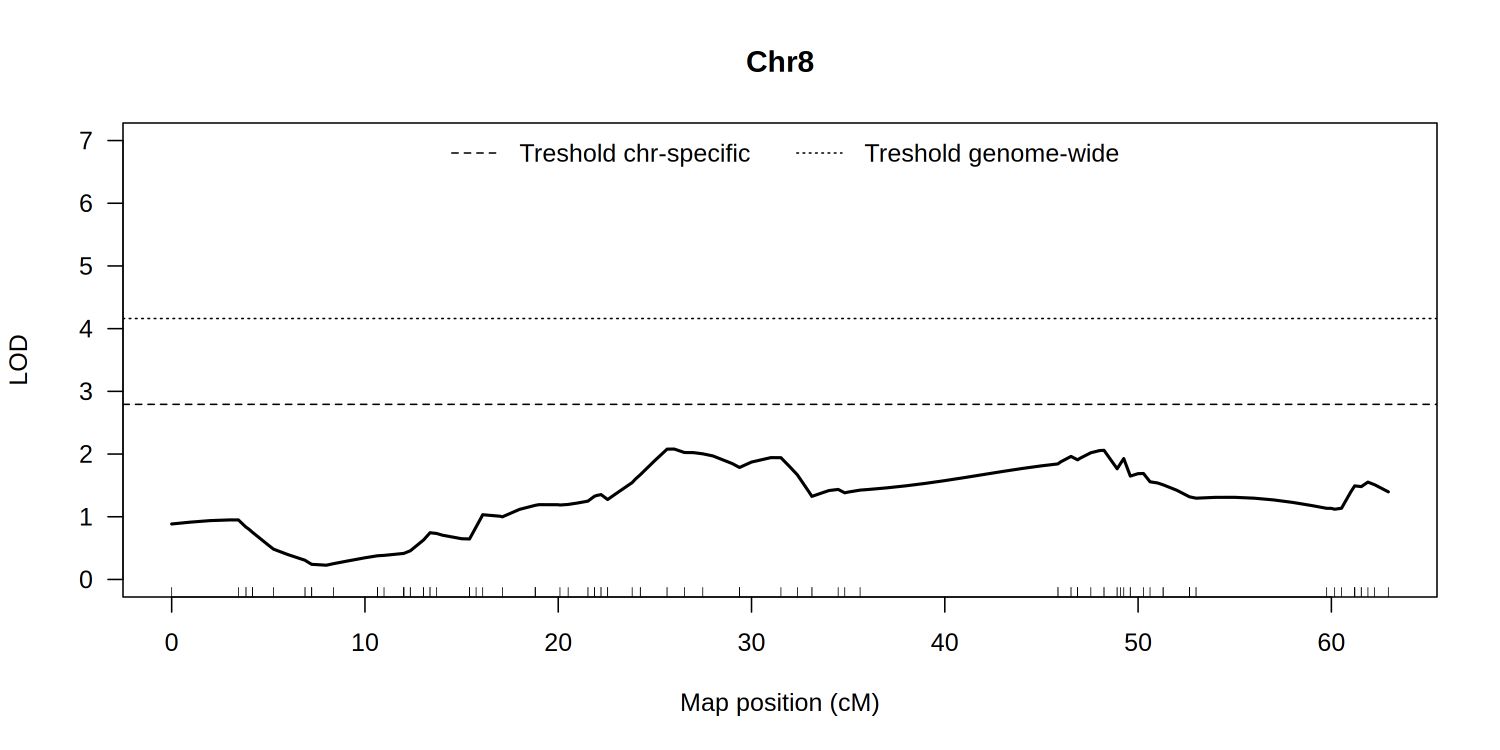

Supplement: Supplementary file 2 [file DataSheet2.zip › Supplementary_Files_4/QTL_analysis/beta_damascenone/beta_damascenone_LODplot_chr8.jpg]

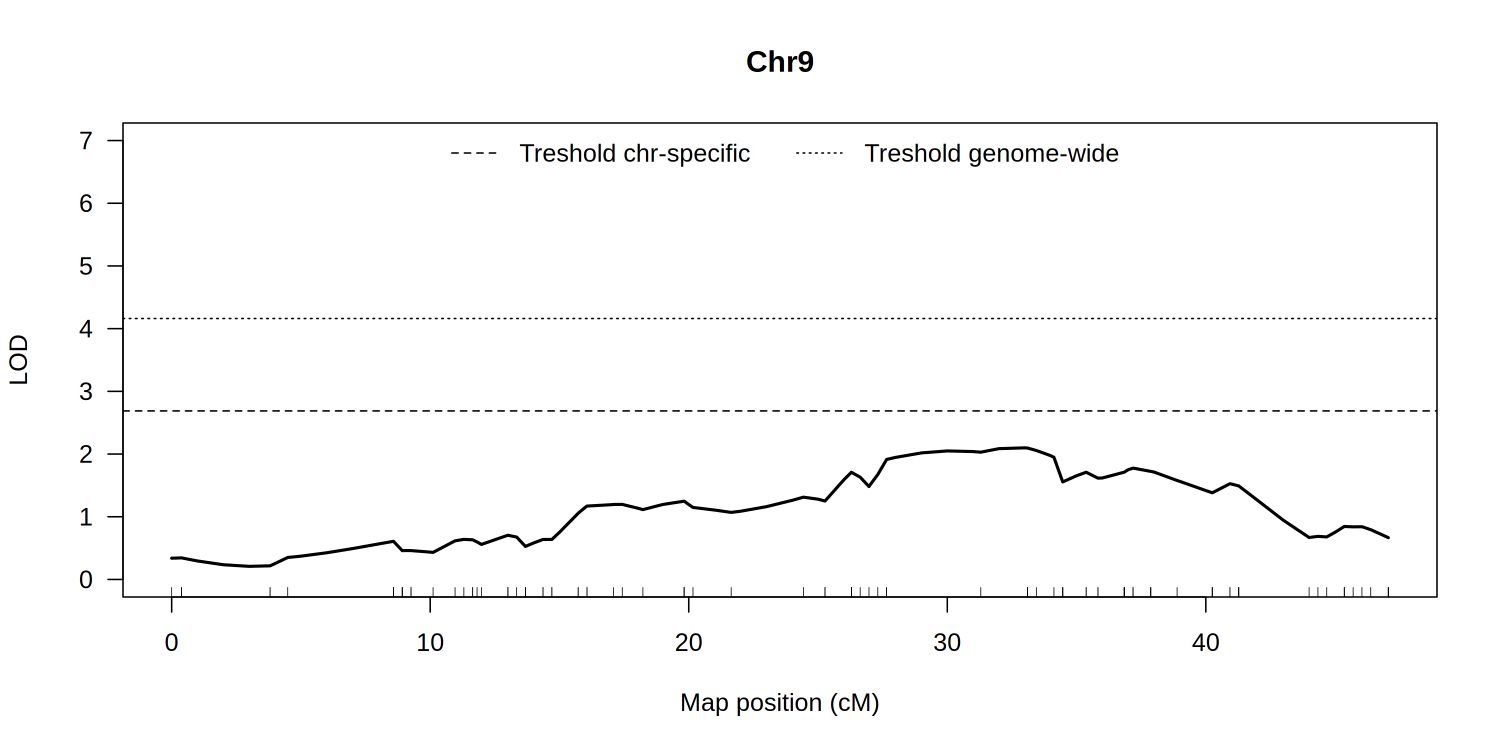

Supplement: Supplementary file 2 [file DataSheet2.zip › Supplementary_Files_4/QTL_analysis/beta_damascenone/beta_damascenone_LODplot_chr9.jpg]

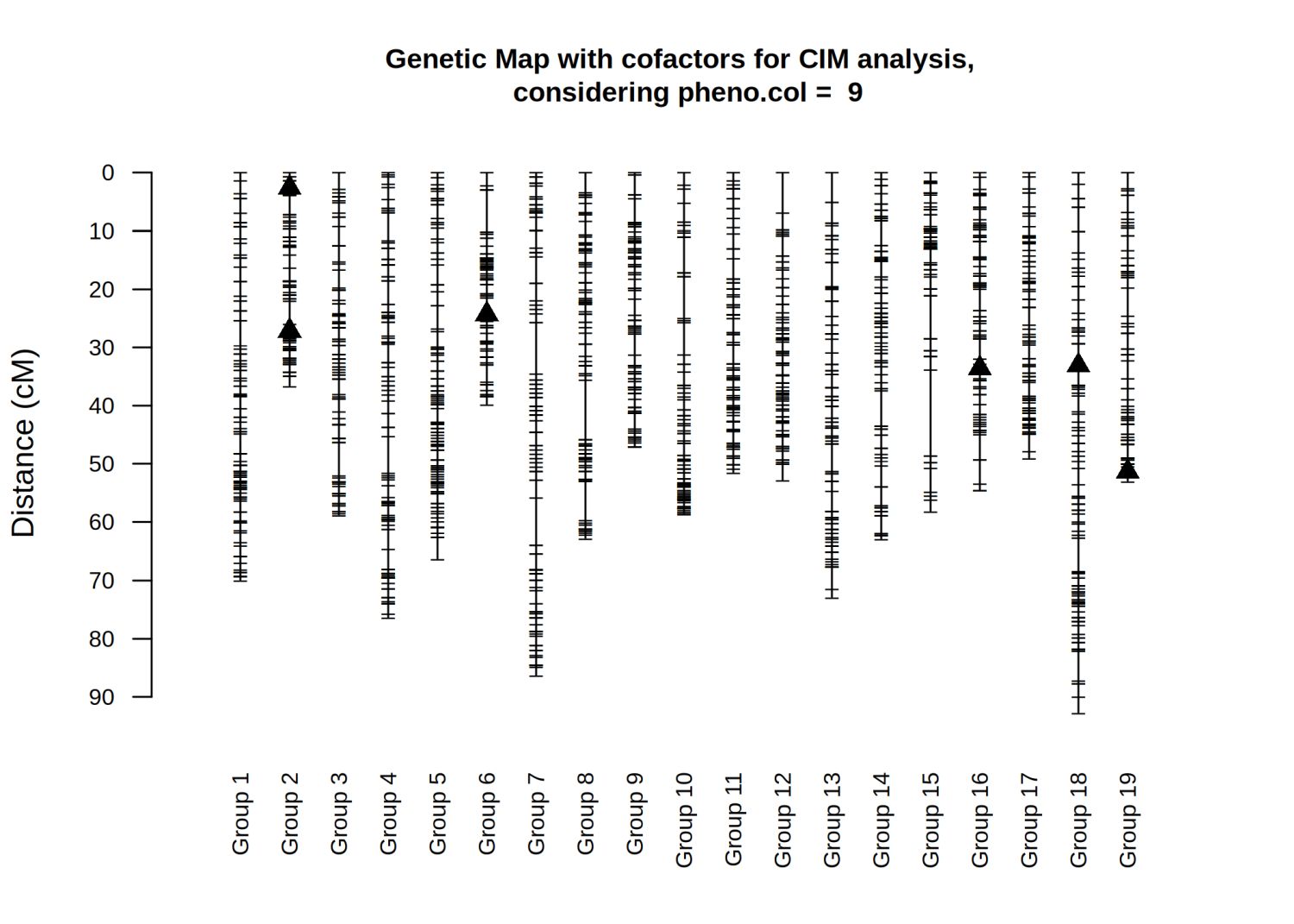

Supplement: Supplementary file 2 [file DataSheet2.zip › Supplementary_Files_4/QTL_analysis/beta_damascenone/CIM analysis/Cofactors_pos.jpg]

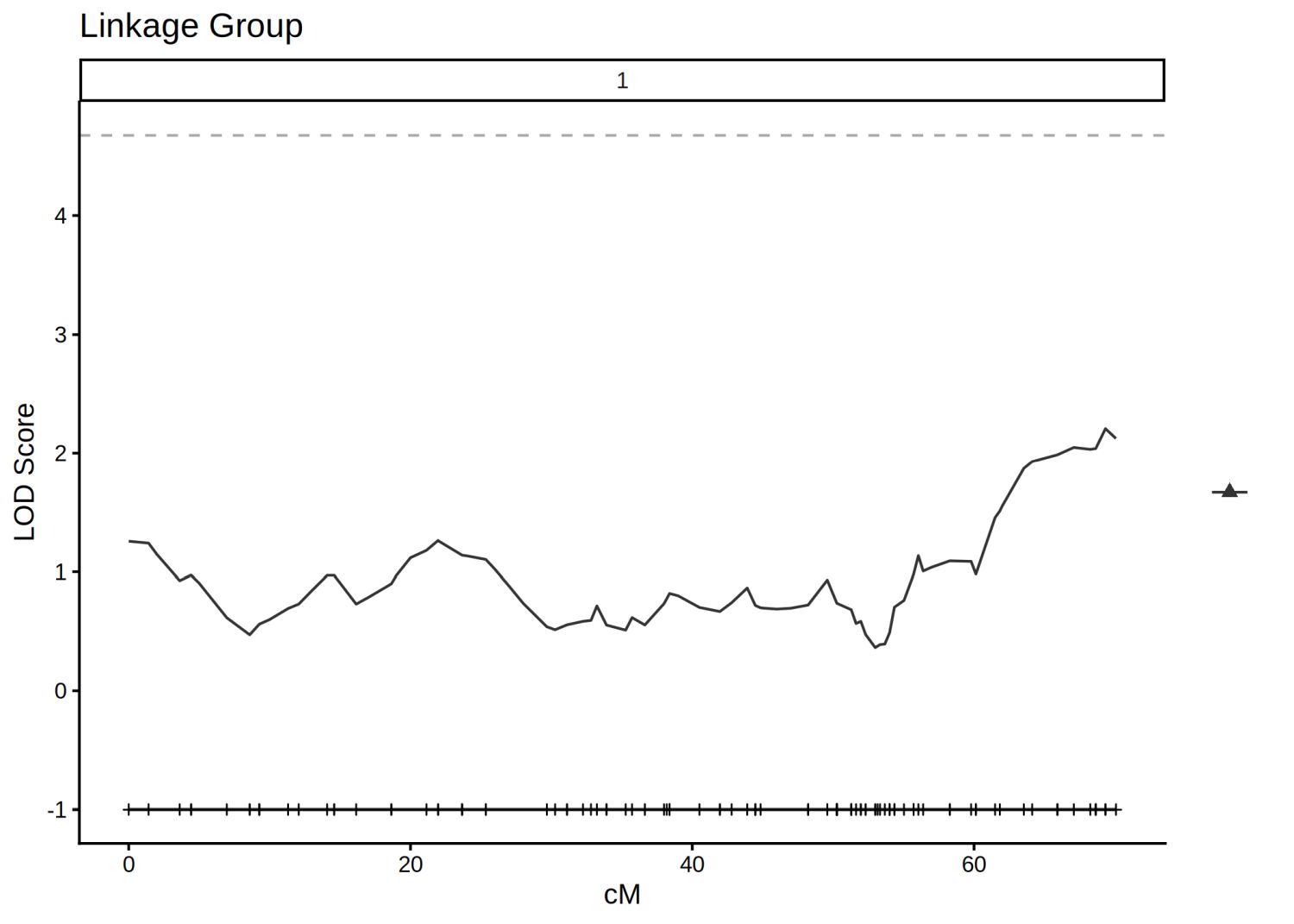

Supplement: Supplementary file 2 [file DataSheet2.zip › Supplementary_Files_4/QTL_analysis/beta_damascenone/CIM analysis/LODplot_chr1.jpg]

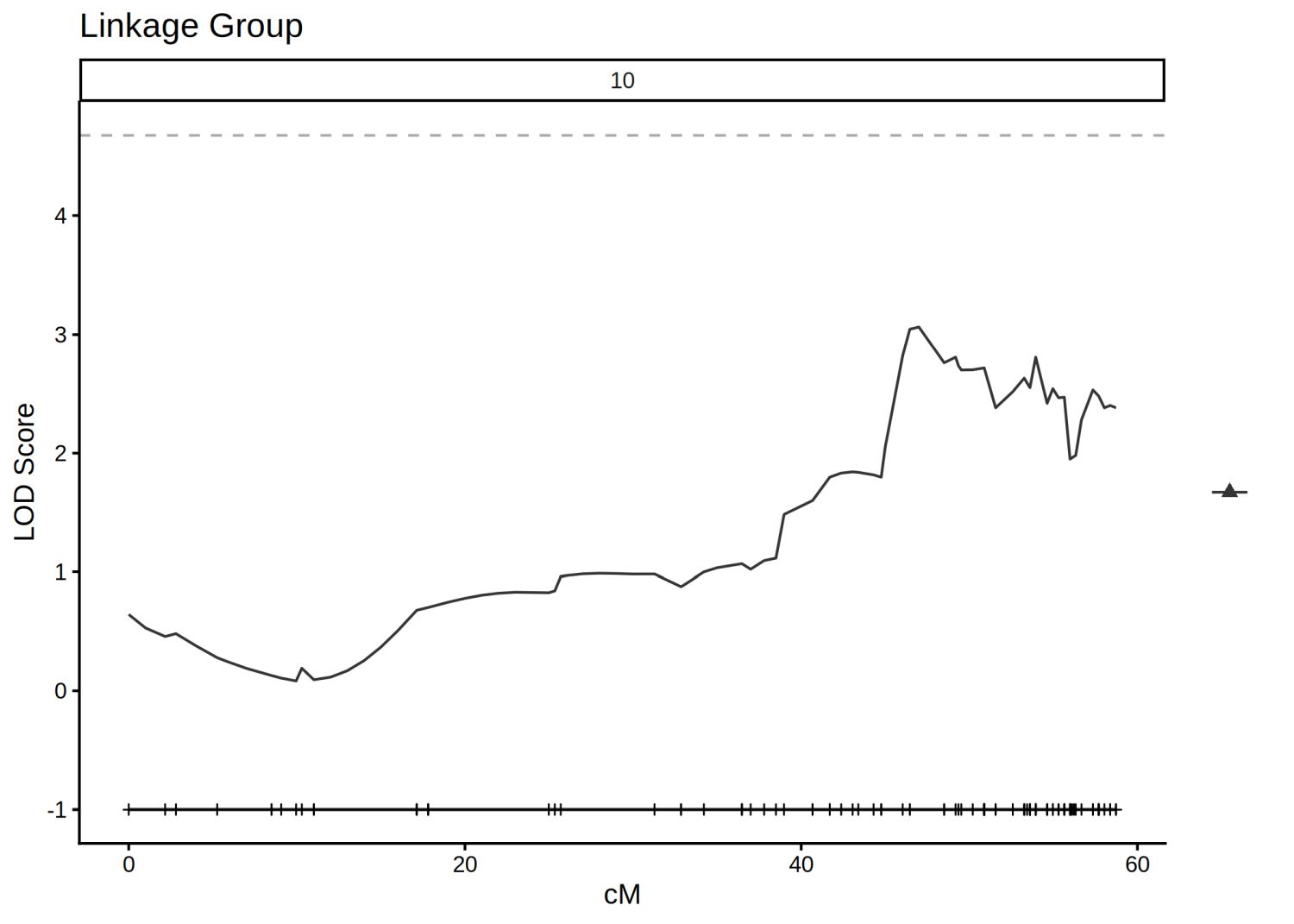

Supplement: Supplementary file 2 [file DataSheet2.zip › Supplementary_Files_4/QTL_analysis/beta_damascenone/CIM analysis/LODplot_chr10.jpg]

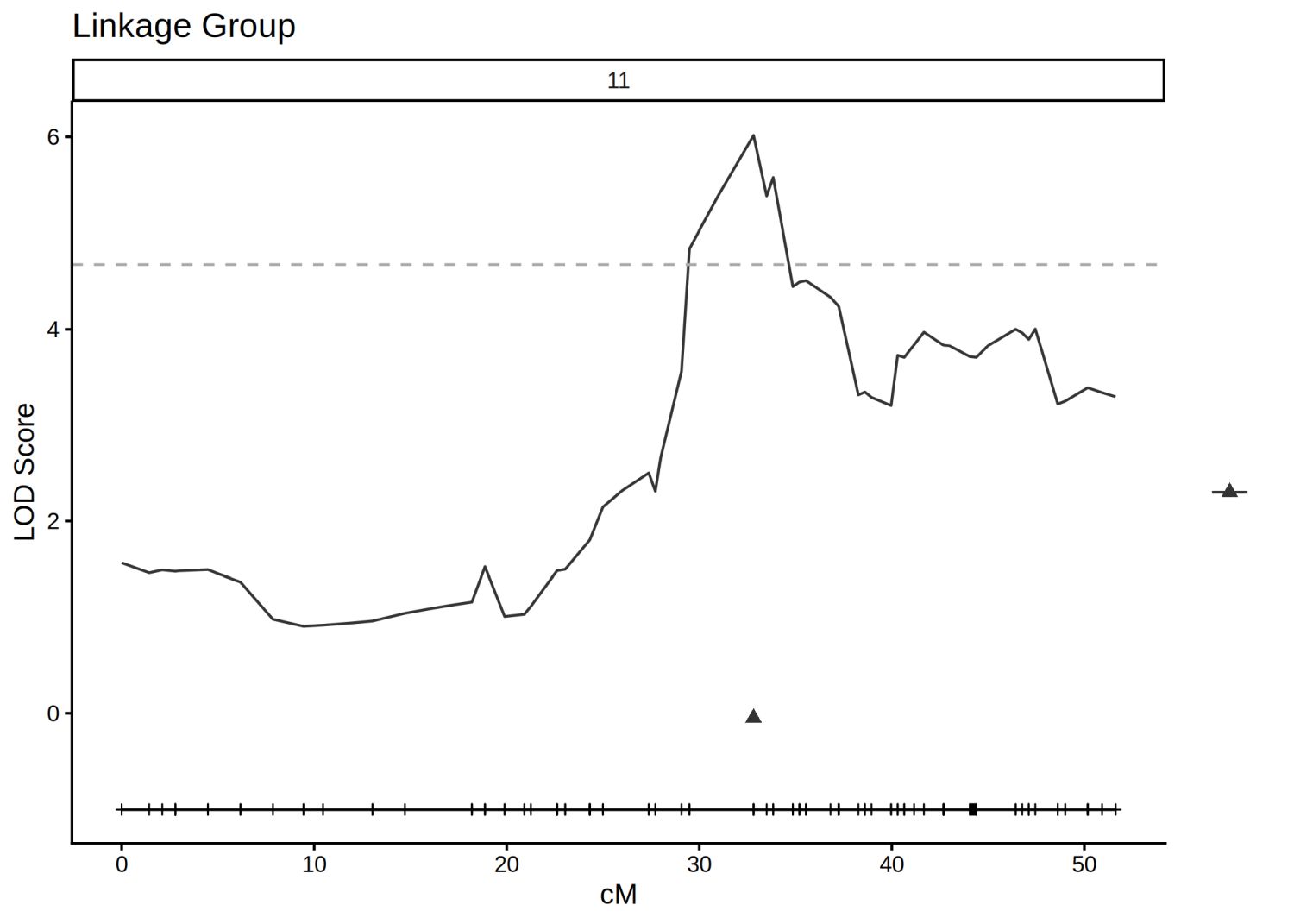

Supplement: Supplementary file 2 [file DataSheet2.zip › Supplementary_Files_4/QTL_analysis/beta_damascenone/CIM analysis/LODplot_chr11.jpg]

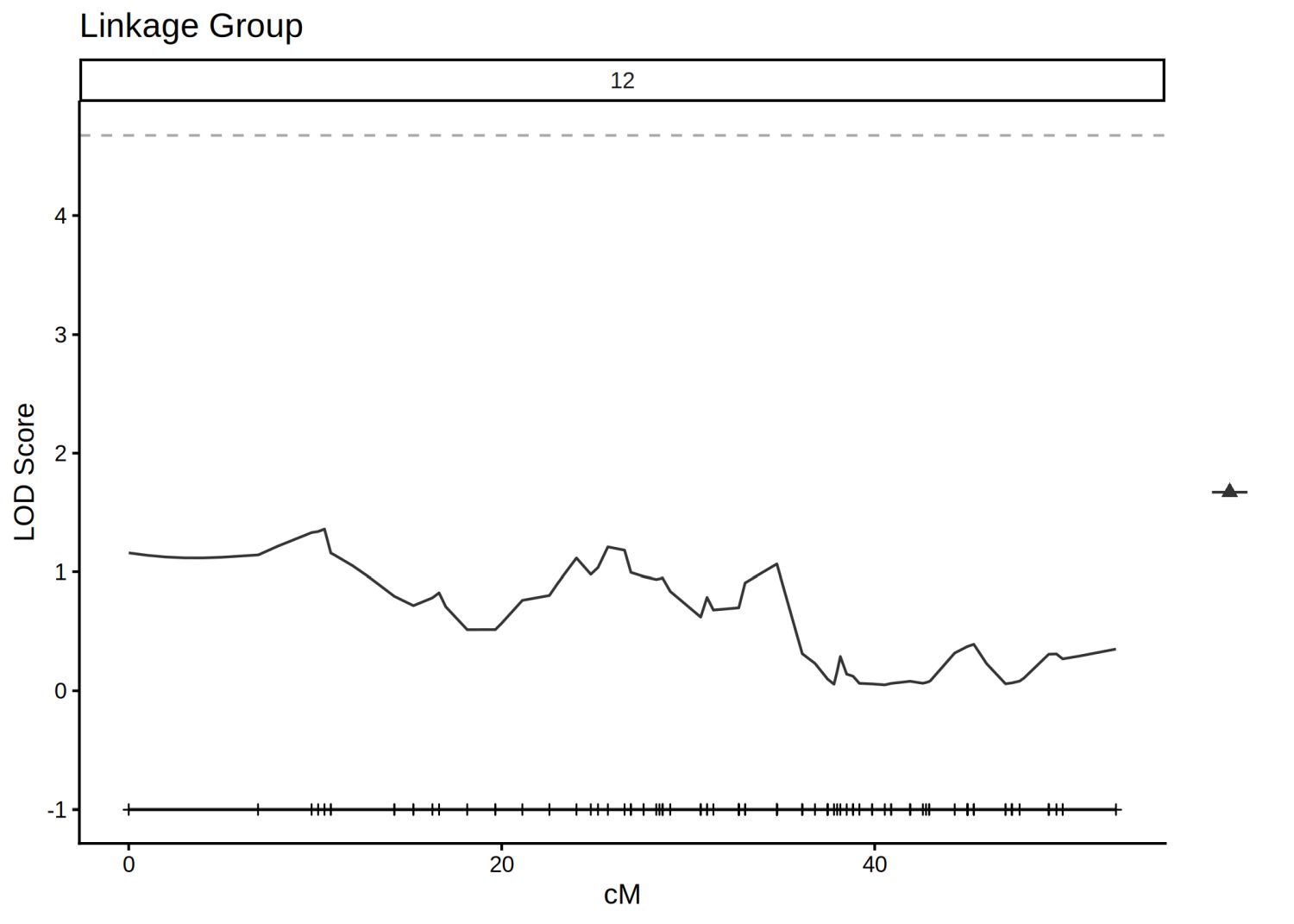

Supplement: Supplementary file 2 [file DataSheet2.zip › Supplementary_Files_4/QTL_analysis/beta_damascenone/CIM analysis/LODplot_chr12.jpg]

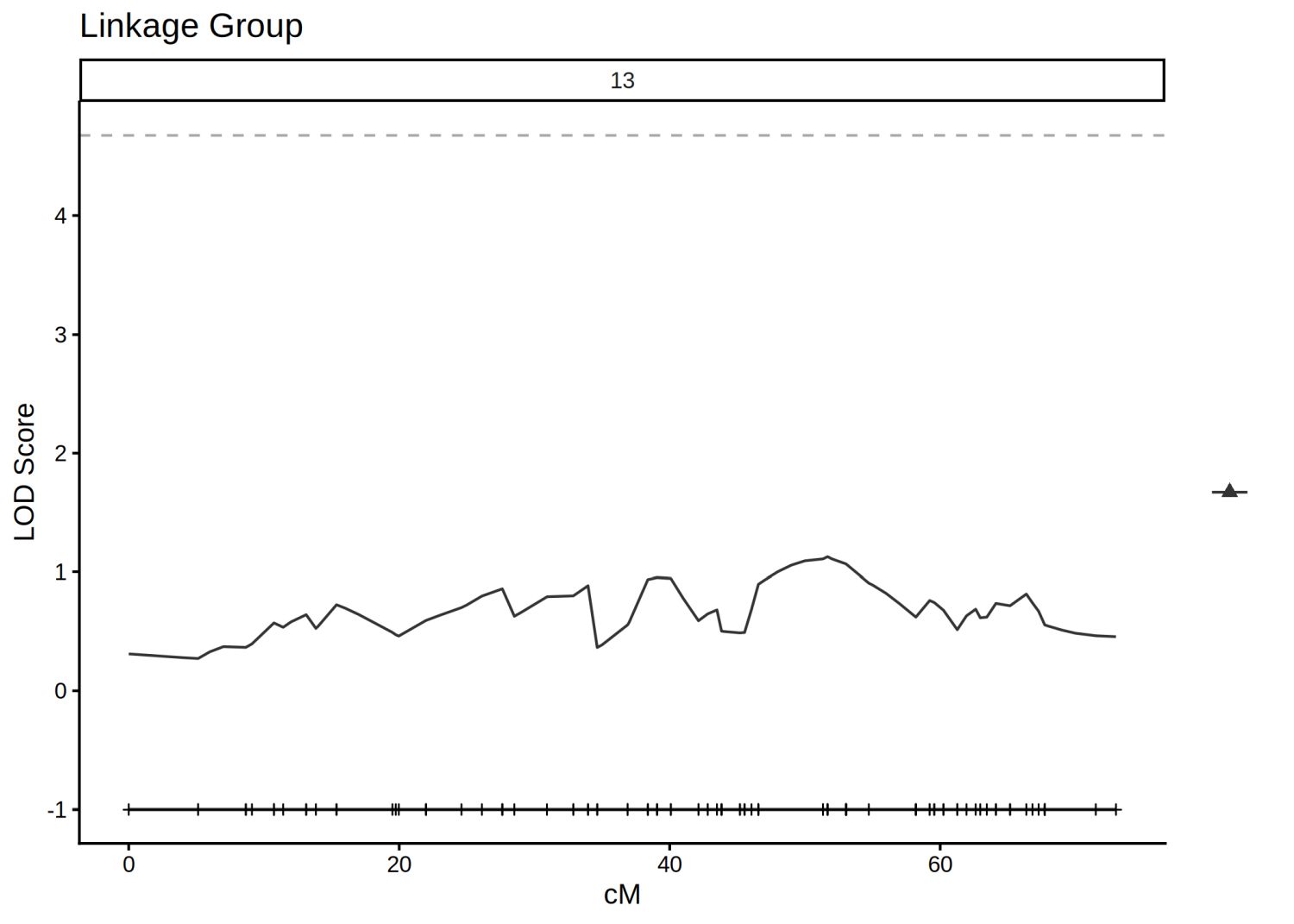

Supplement: Supplementary file 2 [file DataSheet2.zip › Supplementary_Files_4/QTL_analysis/beta_damascenone/CIM analysis/LODplot_chr13.jpg]

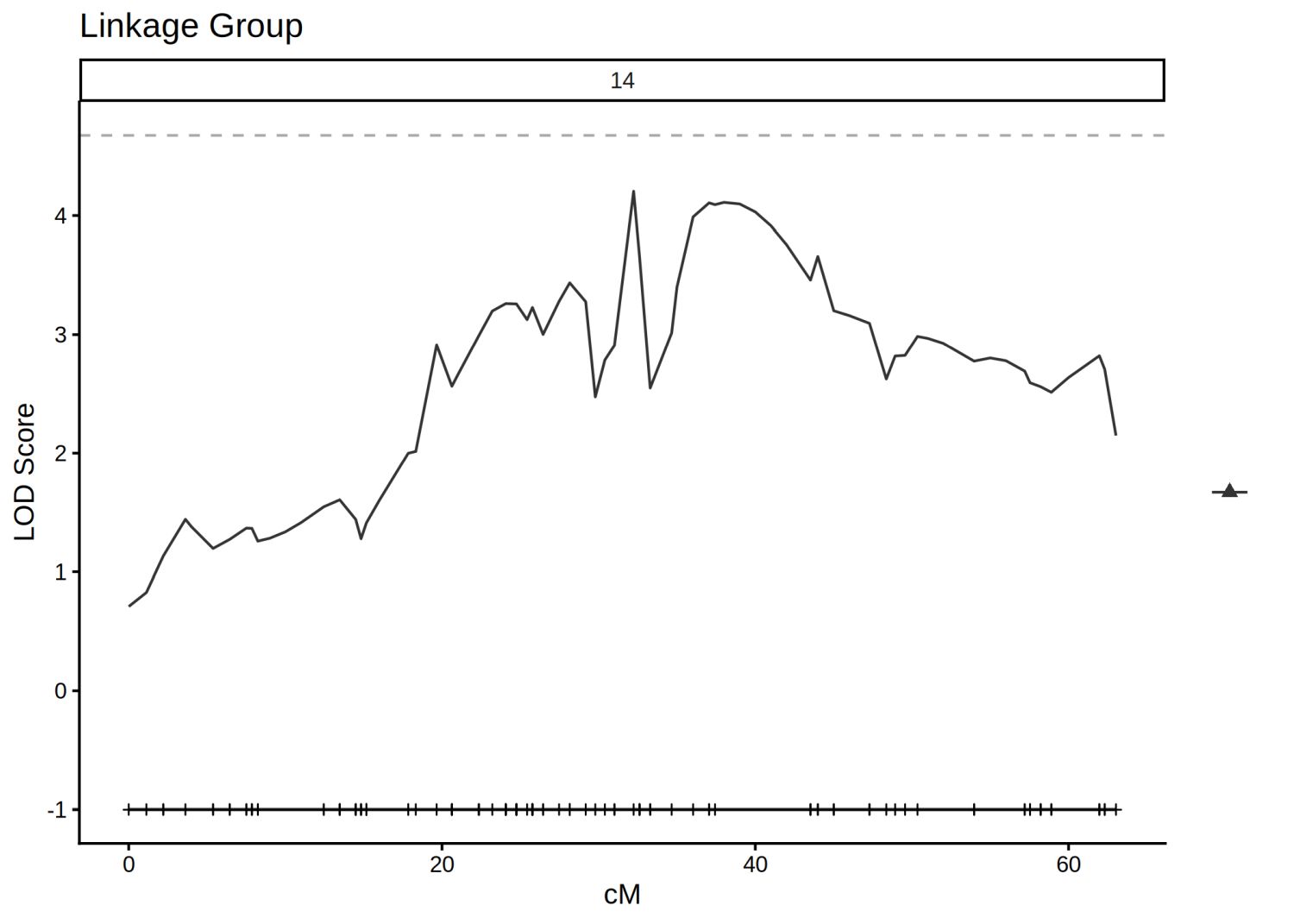

Supplement: Supplementary file 2 [file DataSheet2.zip › Supplementary_Files_4/QTL_analysis/beta_damascenone/CIM analysis/LODplot_chr14.jpg]

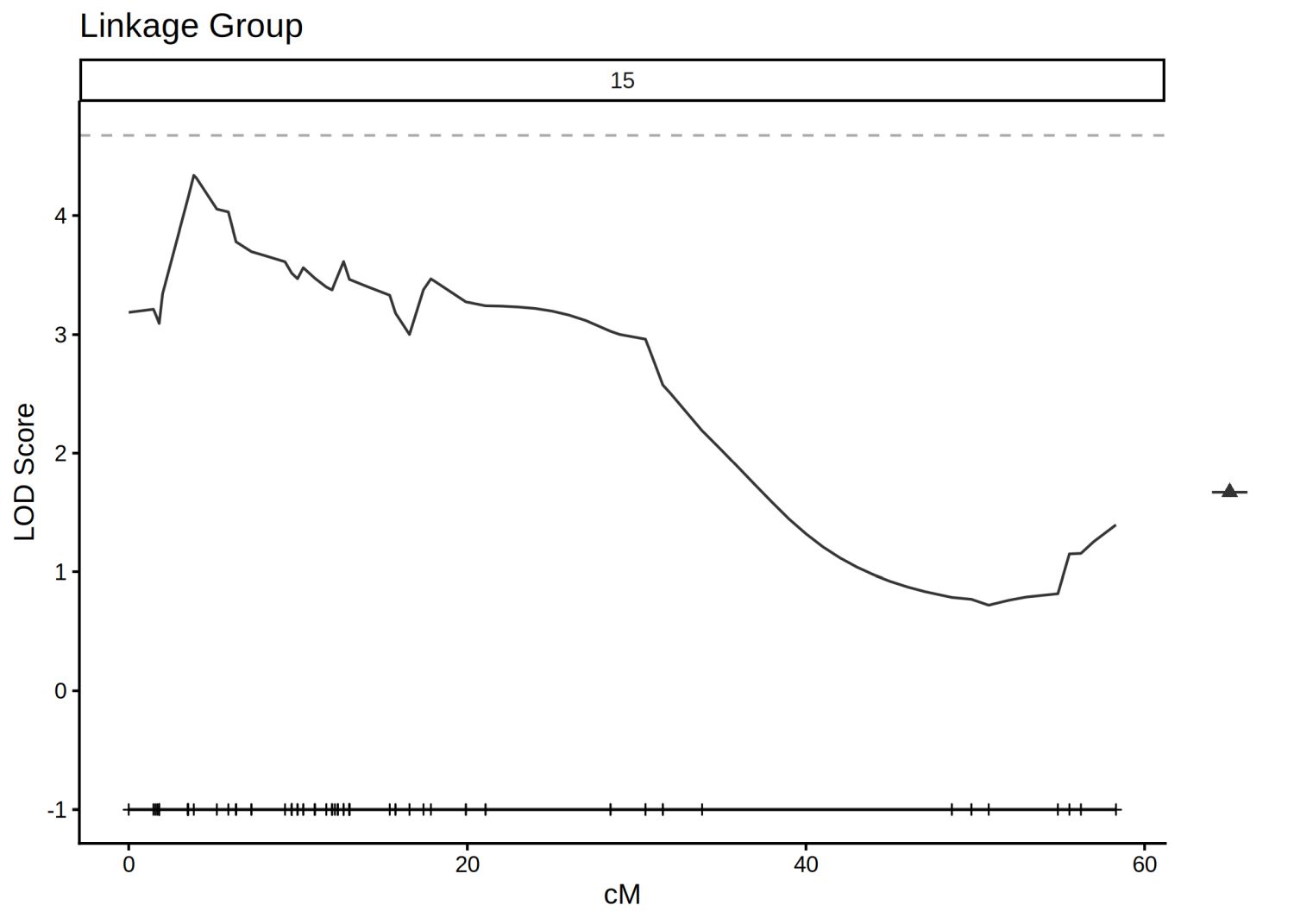

Supplement: Supplementary file 2 [file DataSheet2.zip › Supplementary_Files_4/QTL_analysis/beta_damascenone/CIM analysis/LODplot_chr15.jpg]

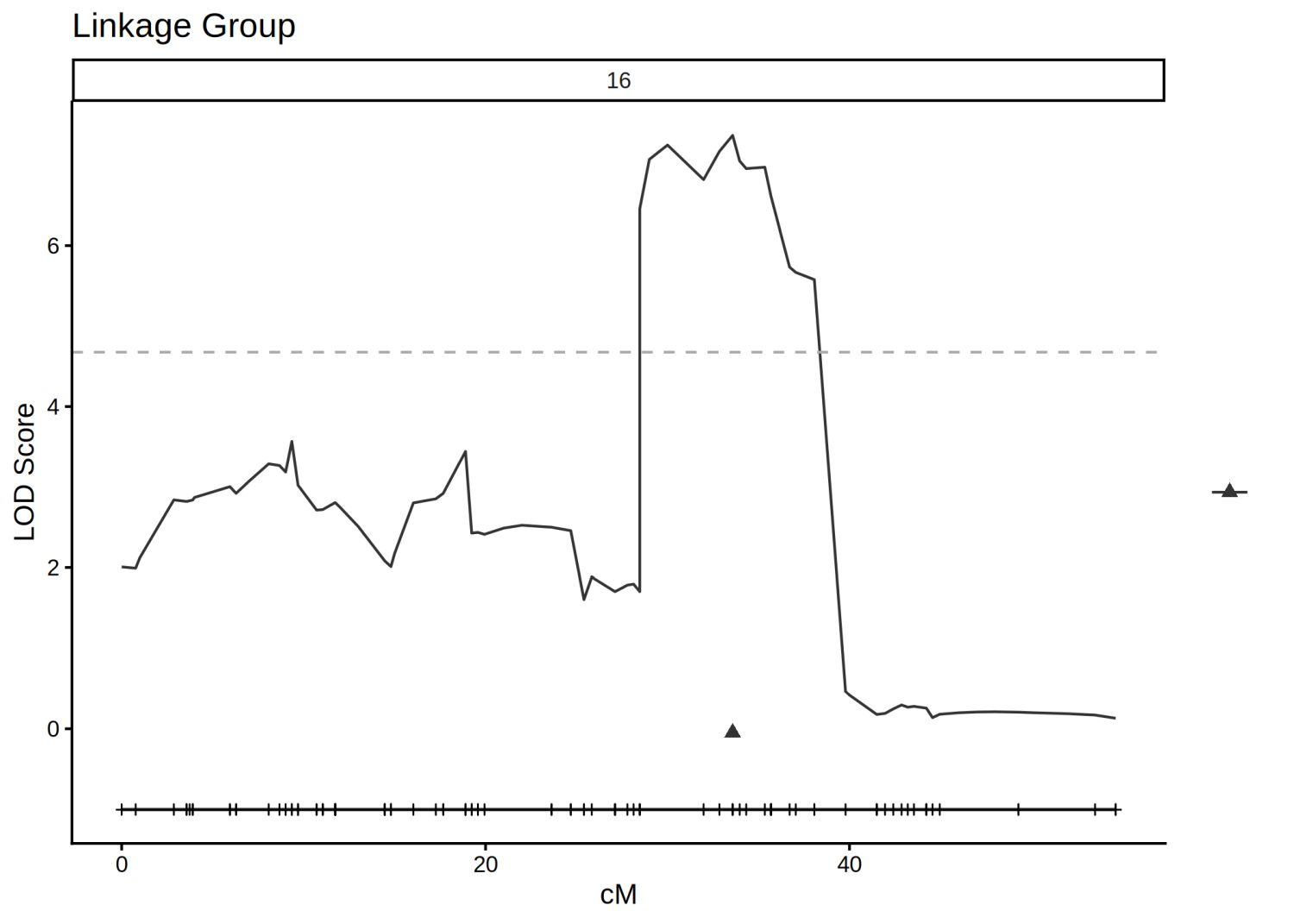

Supplement: Supplementary file 2 [file DataSheet2.zip › Supplementary_Files_4/QTL_analysis/beta_damascenone/CIM analysis/LODplot_chr16.jpg]

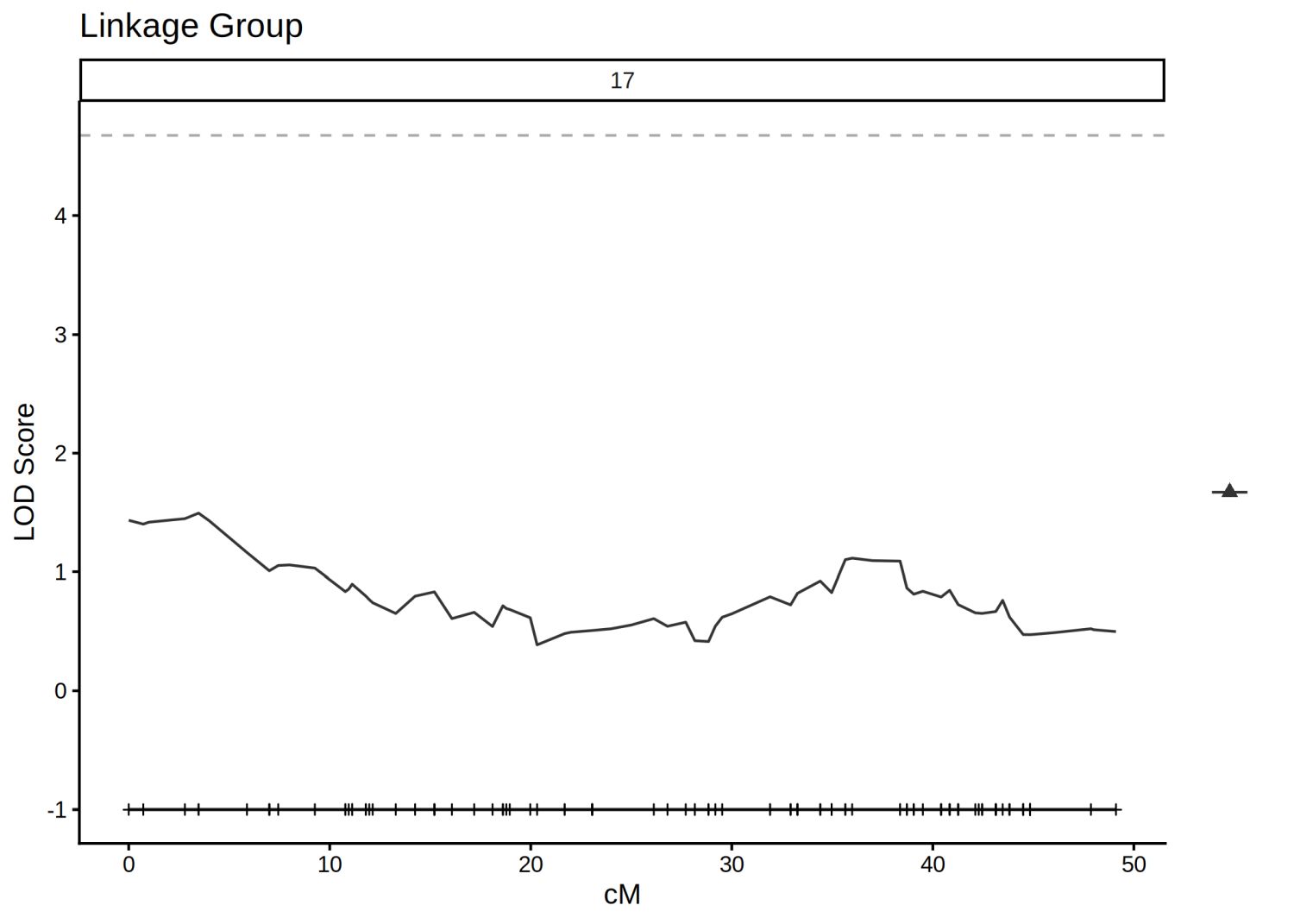

Supplement: Supplementary file 2 [file DataSheet2.zip › Supplementary_Files_4/QTL_analysis/beta_damascenone/CIM analysis/LODplot_chr17.jpg]

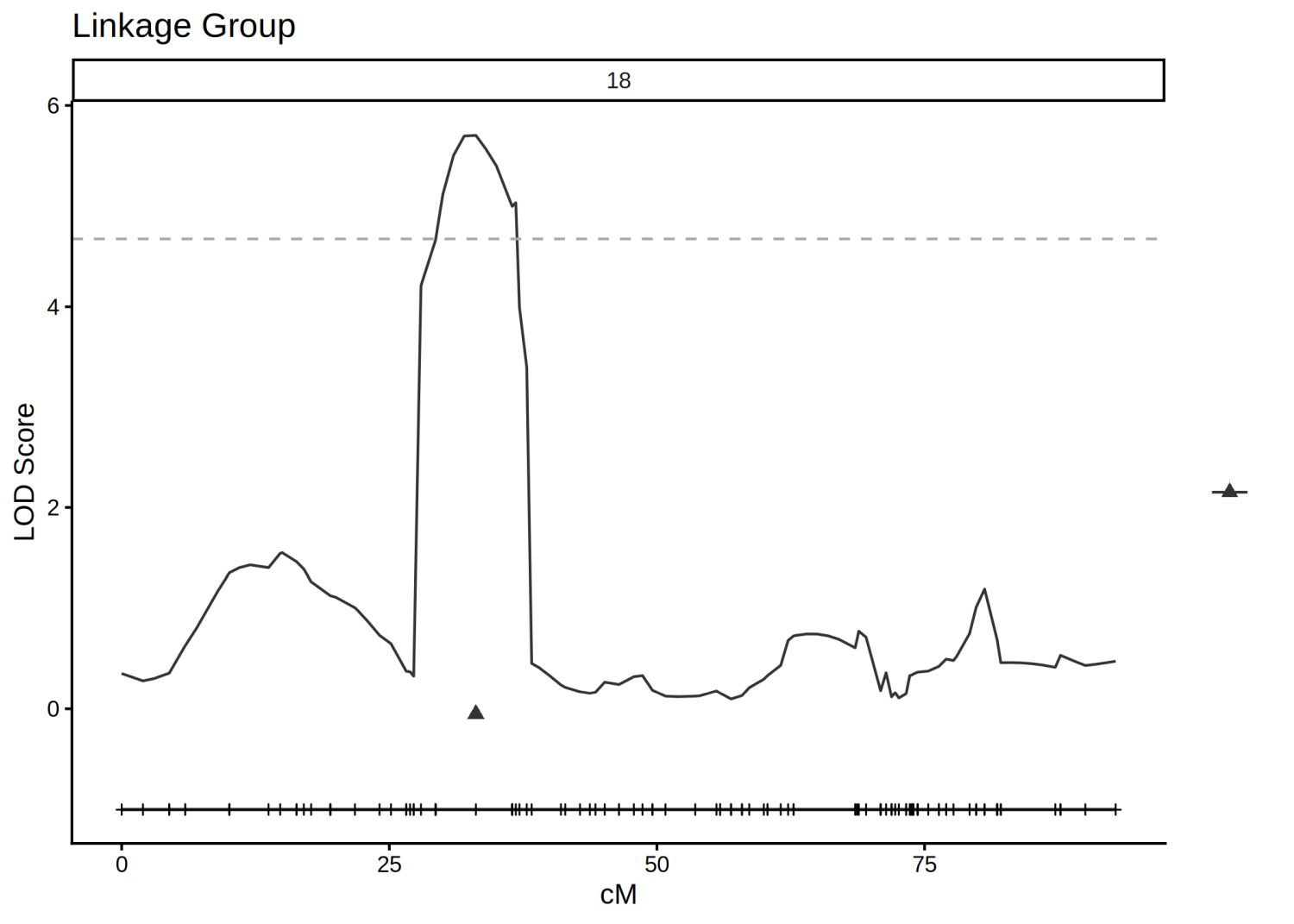

Supplement: Supplementary file 2 [file DataSheet2.zip › Supplementary_Files_4/QTL_analysis/beta_damascenone/CIM analysis/LODplot_chr18.jpg]

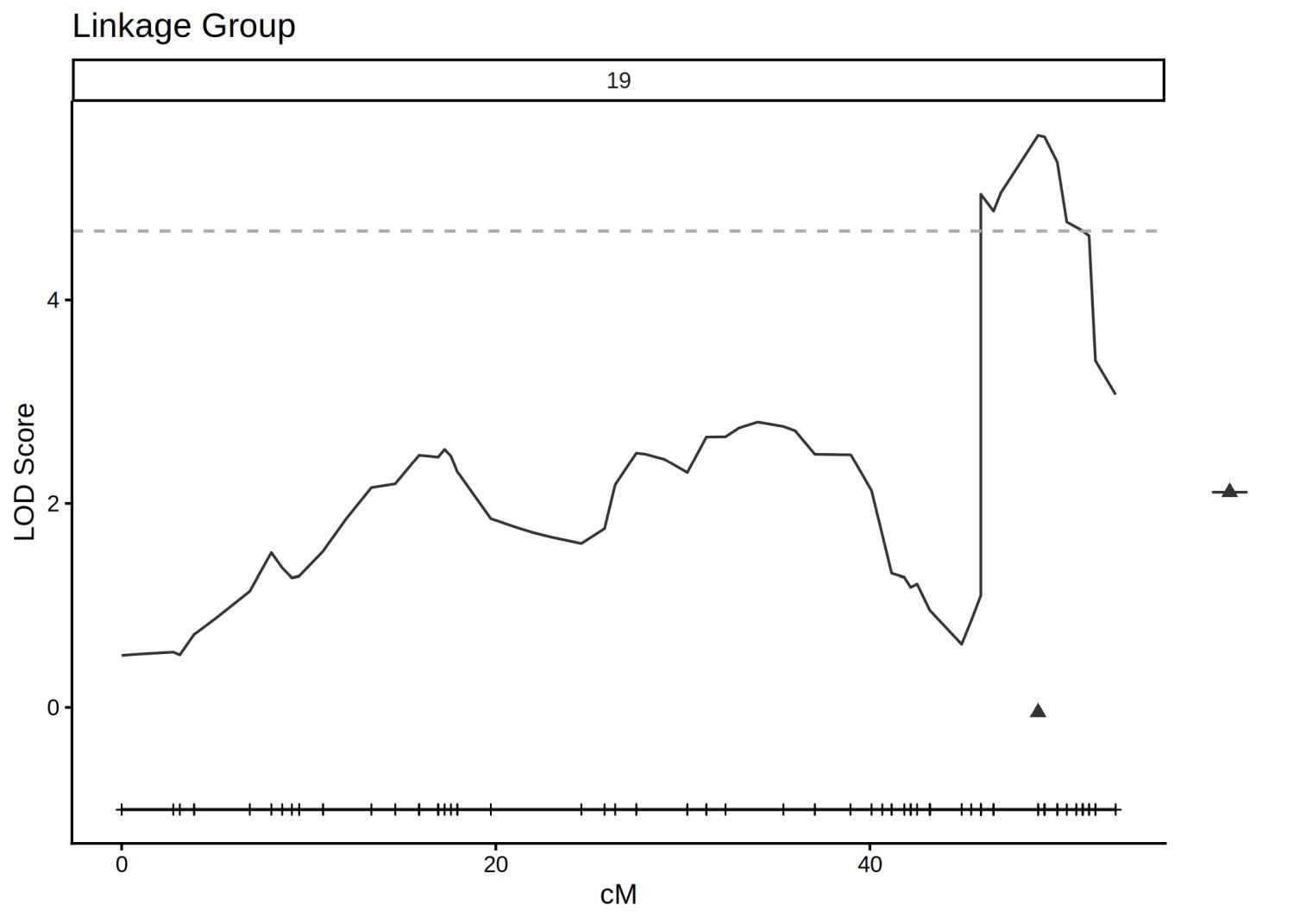

Supplement: Supplementary file 2 [file DataSheet2.zip › Supplementary_Files_4/QTL_analysis/beta_damascenone/CIM analysis/LODplot_chr19.jpg]

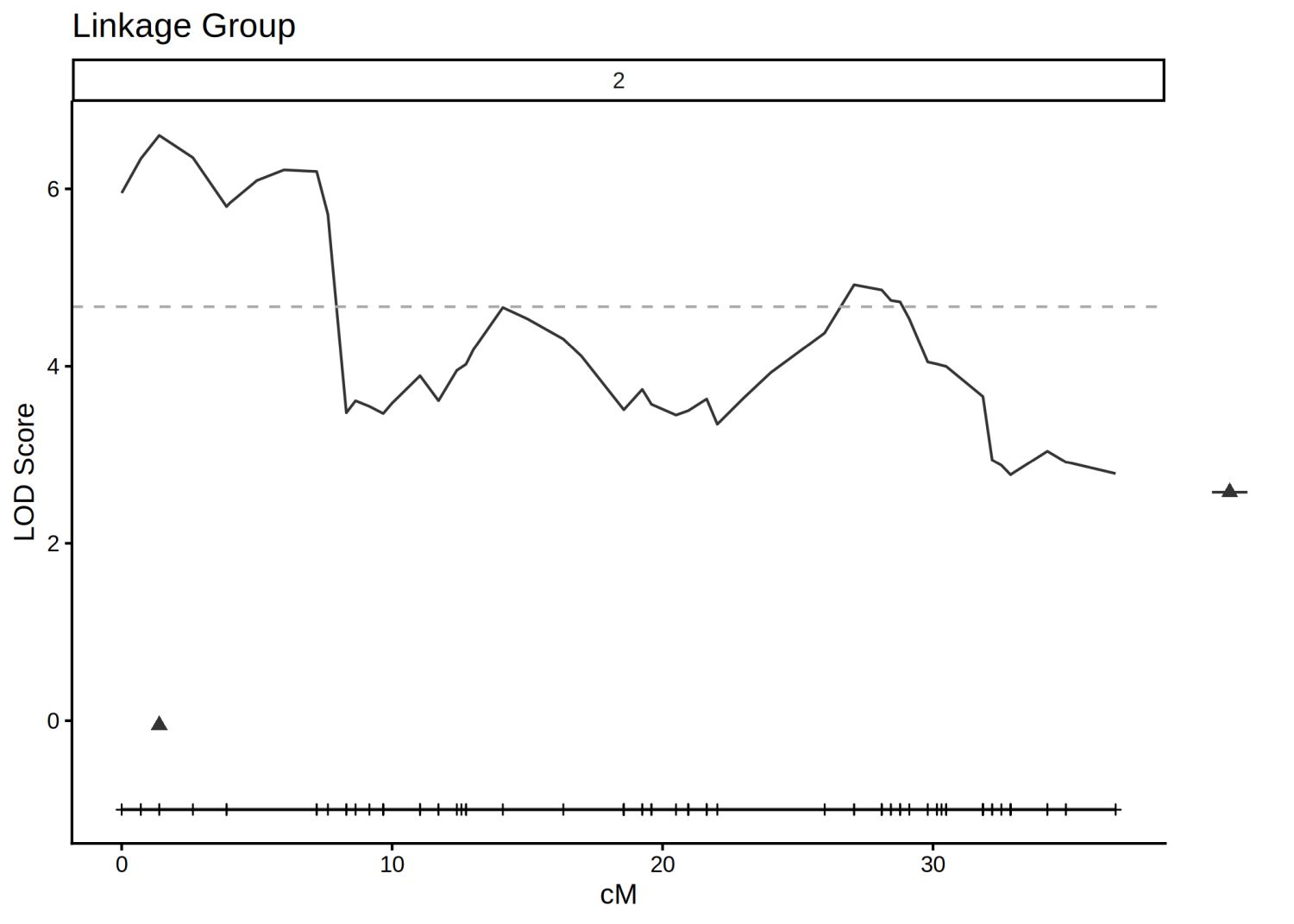

Supplement: Supplementary file 2 [file DataSheet2.zip › Supplementary_Files_4/QTL_analysis/beta_damascenone/CIM analysis/LODplot_chr2.jpg]

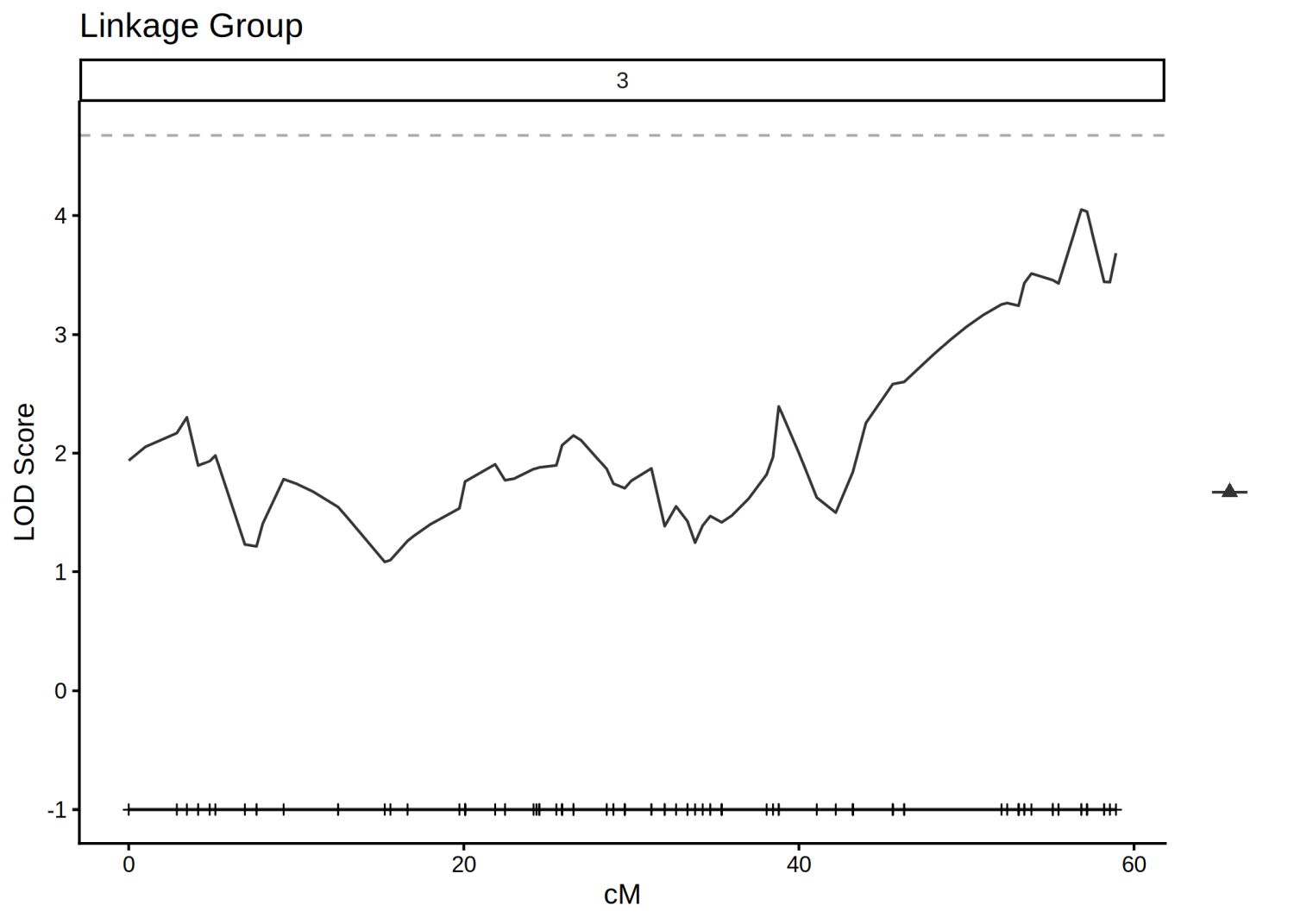

Supplement: Supplementary file 2 [file DataSheet2.zip › Supplementary_Files_4/QTL_analysis/beta_damascenone/CIM analysis/LODplot_chr3.jpg]

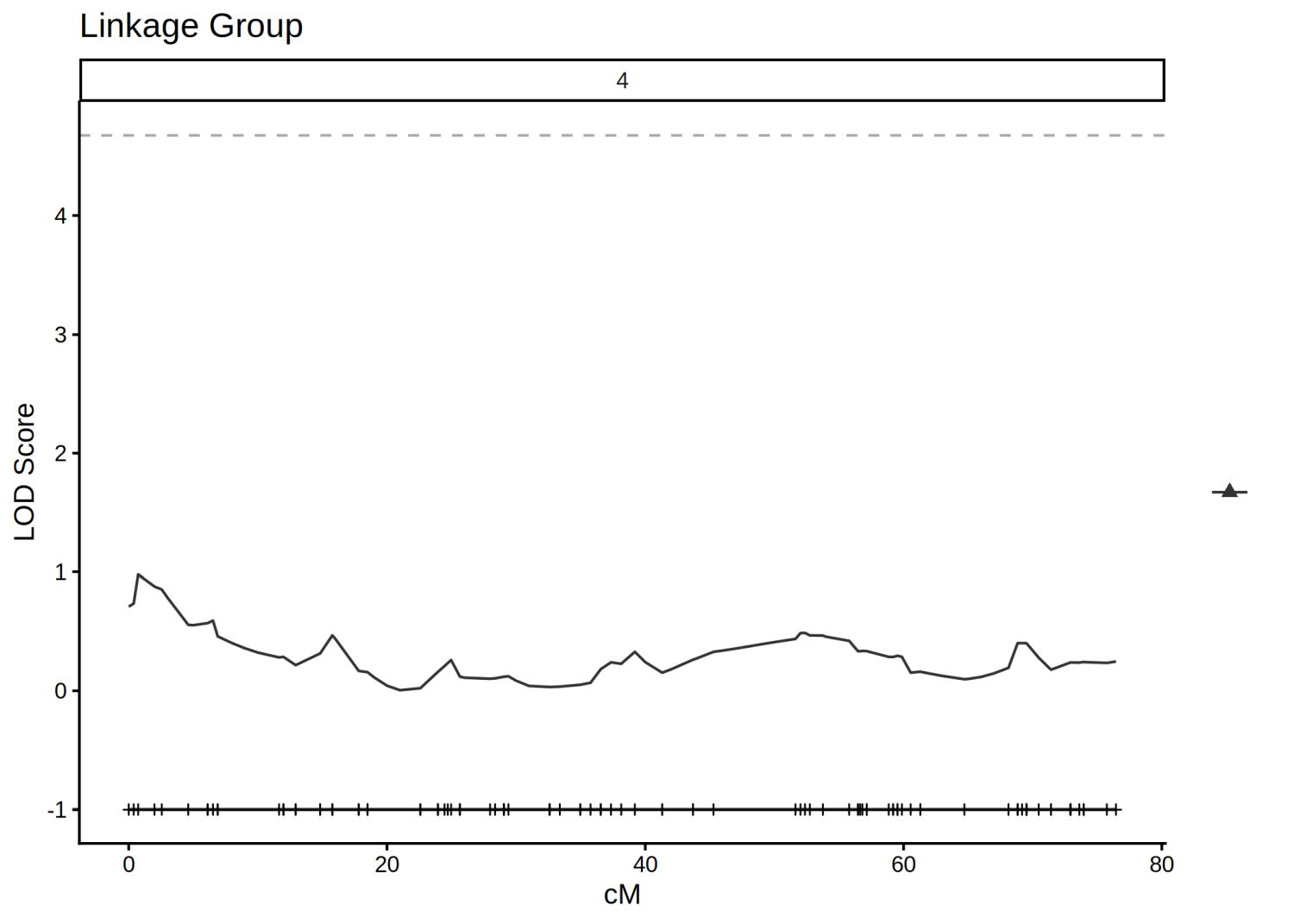

Supplement: Supplementary file 2 [file DataSheet2.zip › Supplementary_Files_4/QTL_analysis/beta_damascenone/CIM analysis/LODplot_chr4.jpg]

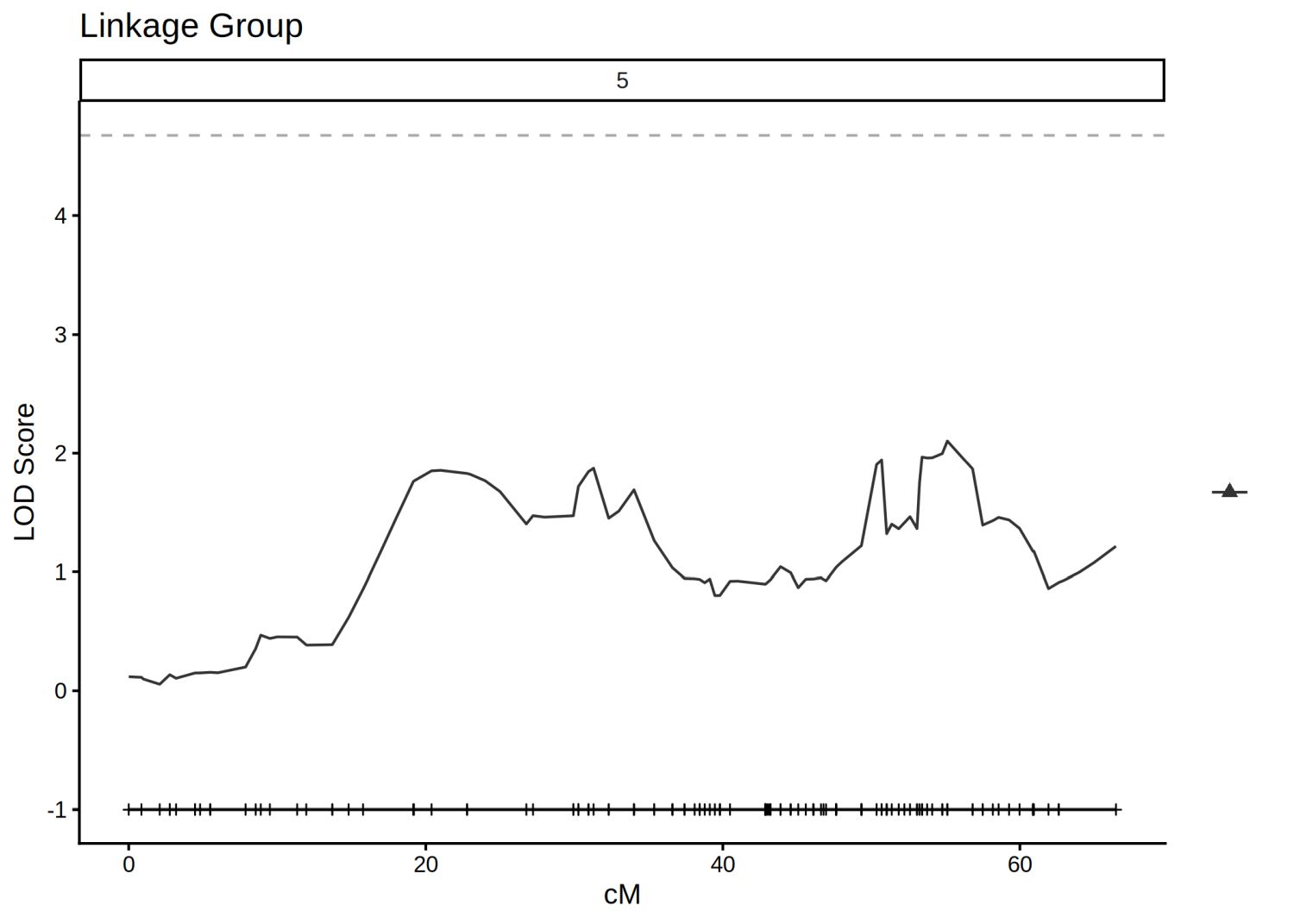

Supplement: Supplementary file 2 [file DataSheet2.zip › Supplementary_Files_4/QTL_analysis/beta_damascenone/CIM analysis/LODplot_chr5.jpg]

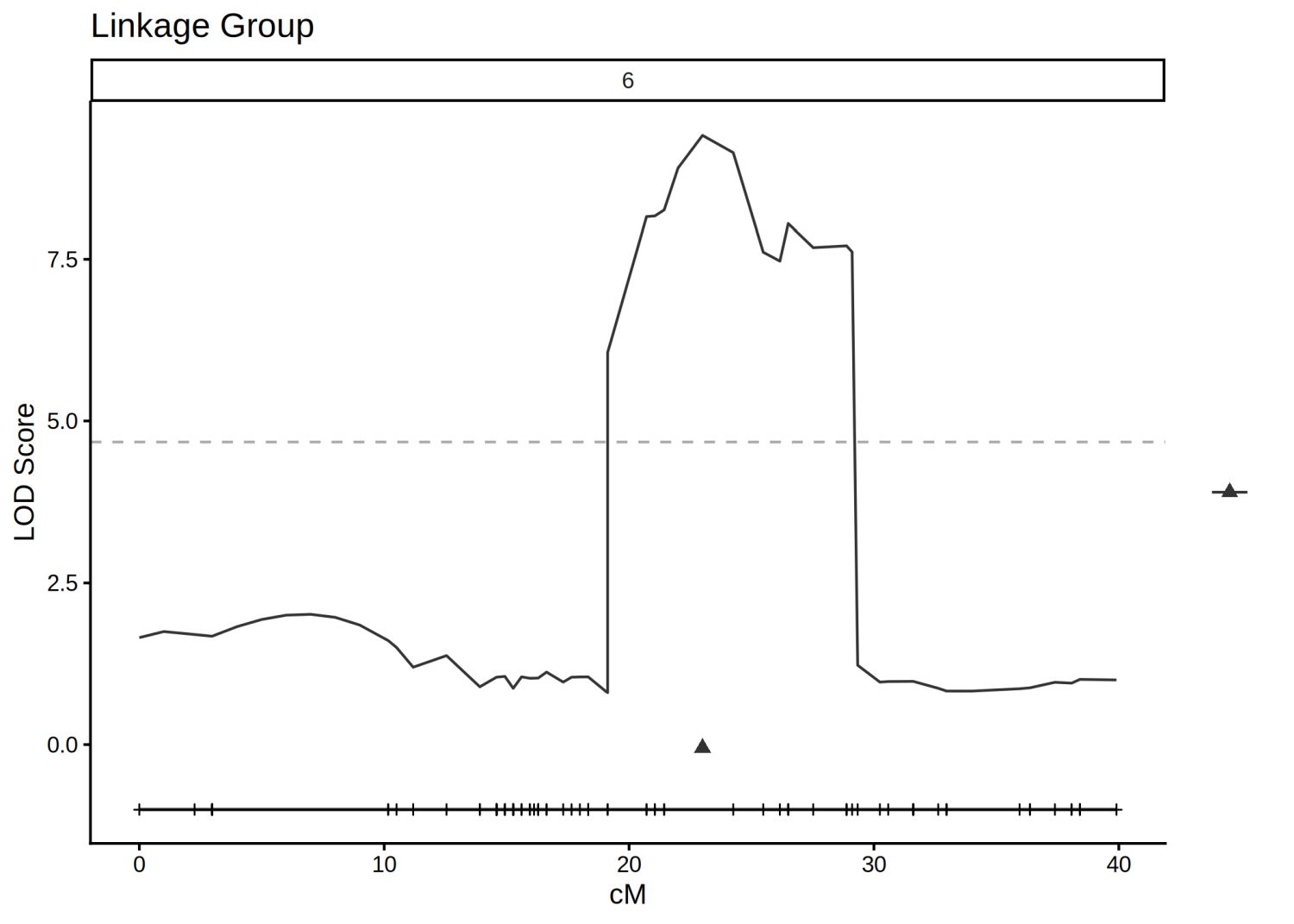

Supplement: Supplementary file 2 [file DataSheet2.zip › Supplementary_Files_4/QTL_analysis/beta_damascenone/CIM analysis/LODplot_chr6.jpg]

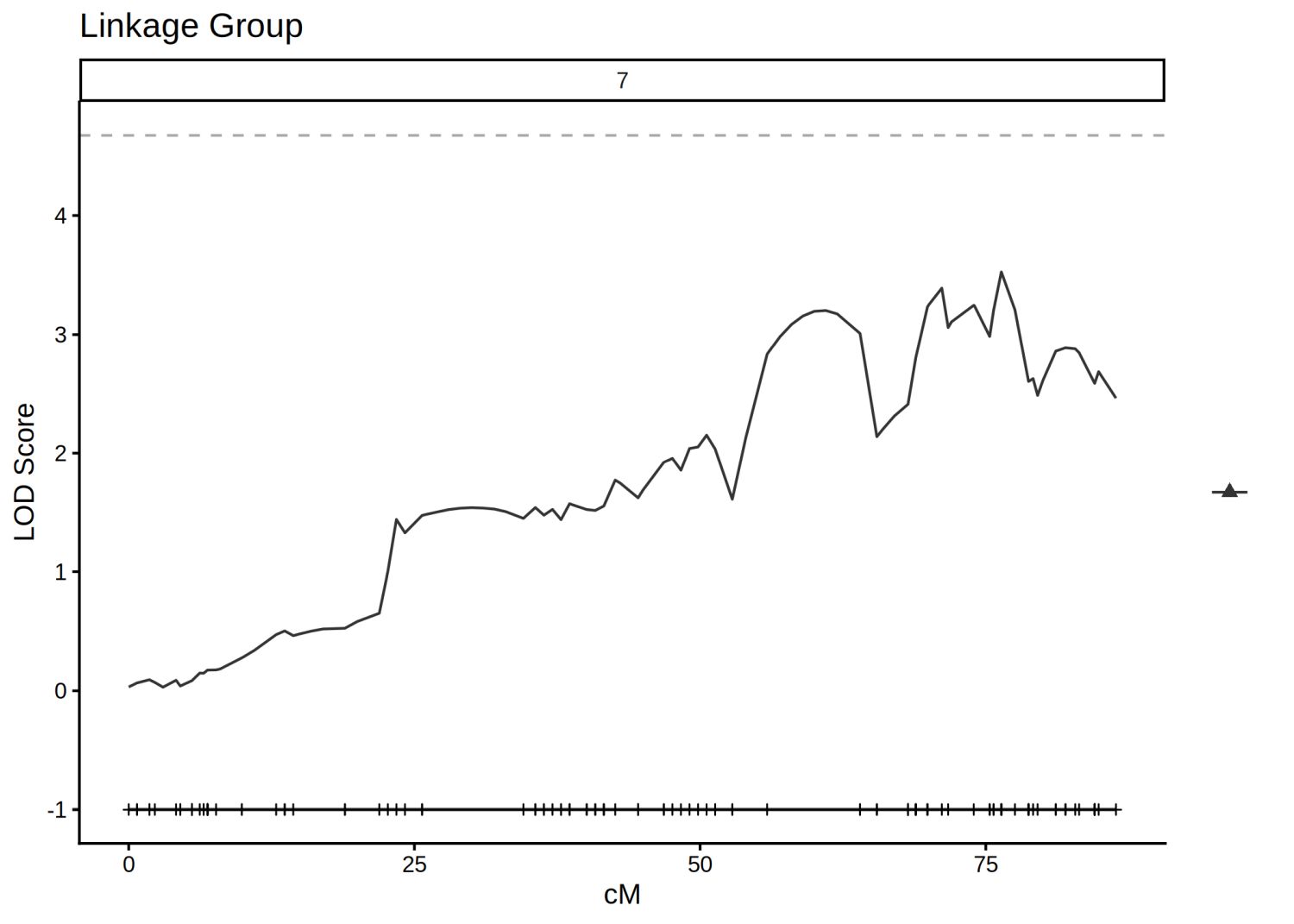

Supplement: Supplementary file 2 [file DataSheet2.zip › Supplementary_Files_4/QTL_analysis/beta_damascenone/CIM analysis/LODplot_chr7.jpg]

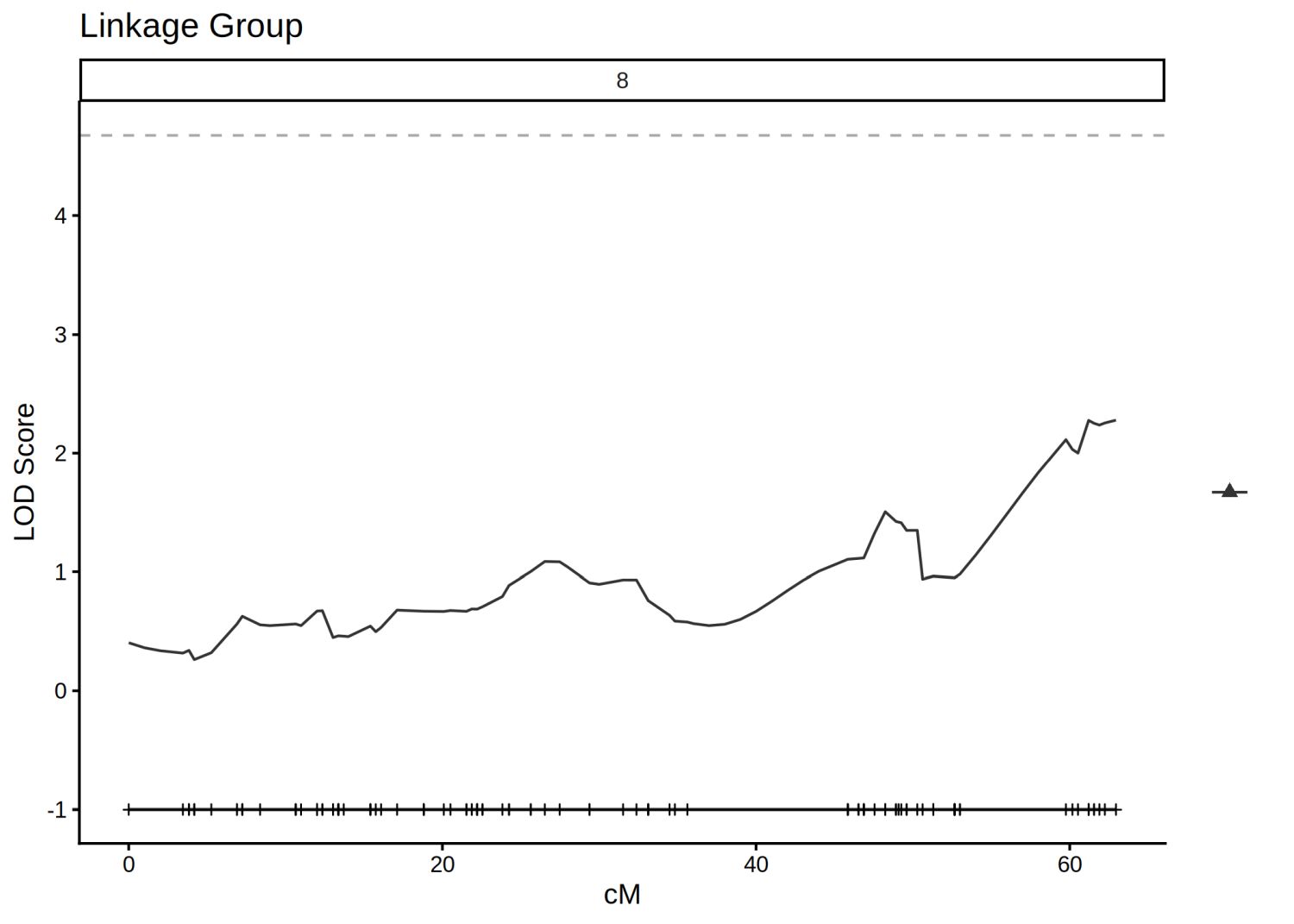

Supplement: Supplementary file 2 [file DataSheet2.zip › Supplementary_Files_4/QTL_analysis/beta_damascenone/CIM analysis/LODplot_chr8.jpg]

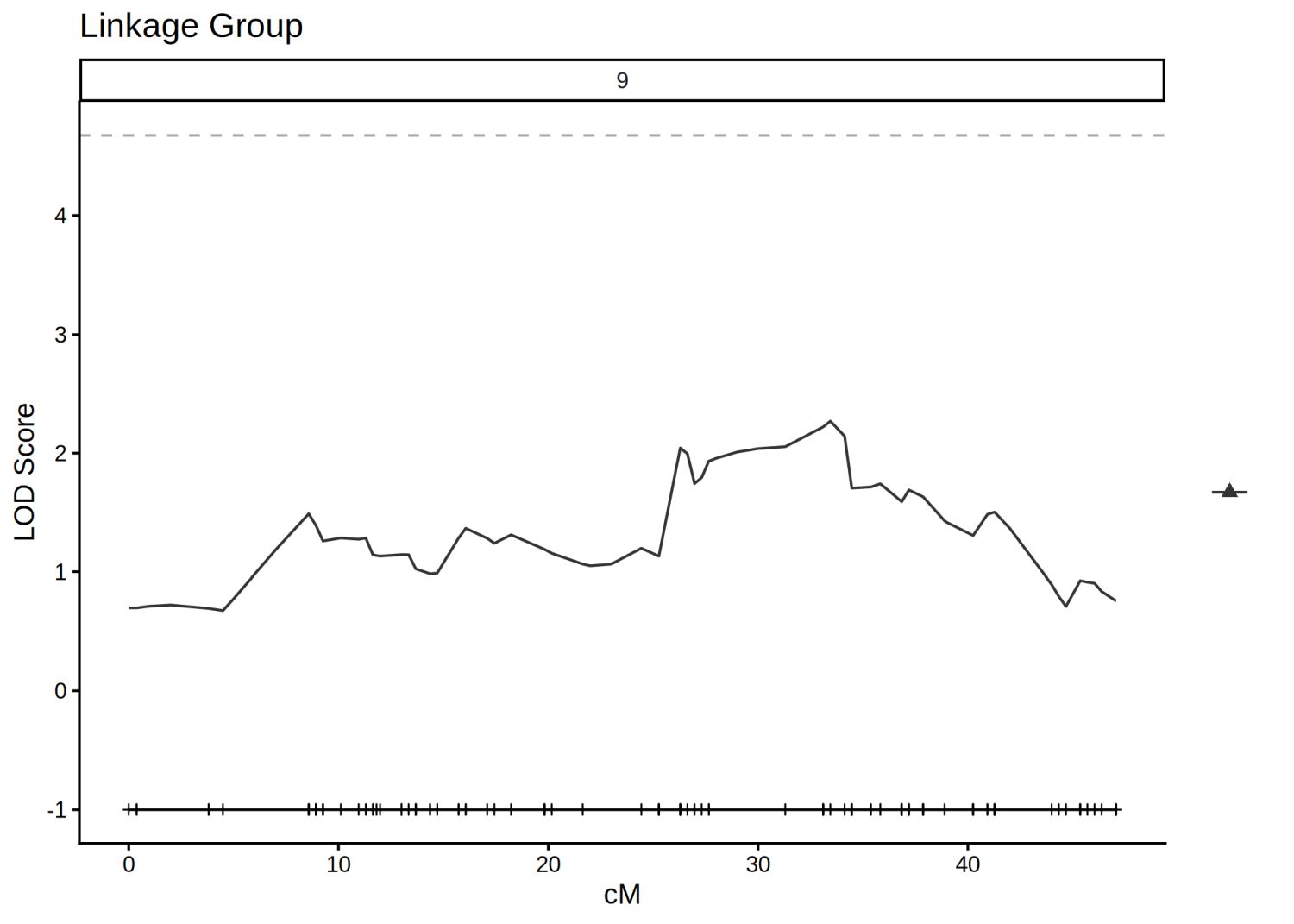

Supplement: Supplementary file 2 [file DataSheet2.zip › Supplementary_Files_4/QTL_analysis/beta_damascenone/CIM analysis/LODplot_chr9.jpg]

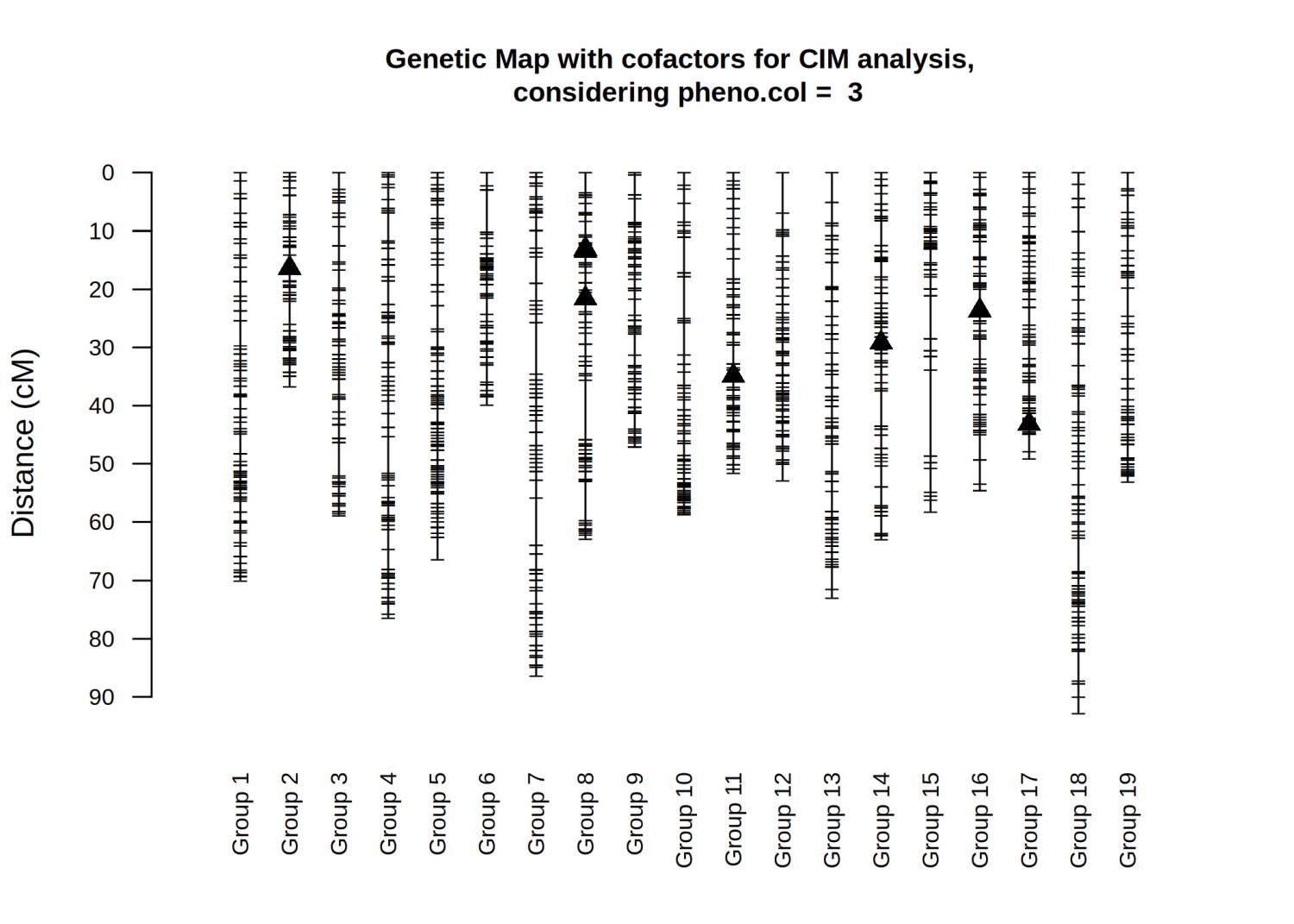

Supplement: Supplementary file 2 [file DataSheet2.zip › Supplementary_Files_4/QTL_analysis/cis_linalool_oxid_furanoid/CIM analysis/Cofactors_pos.jpg]

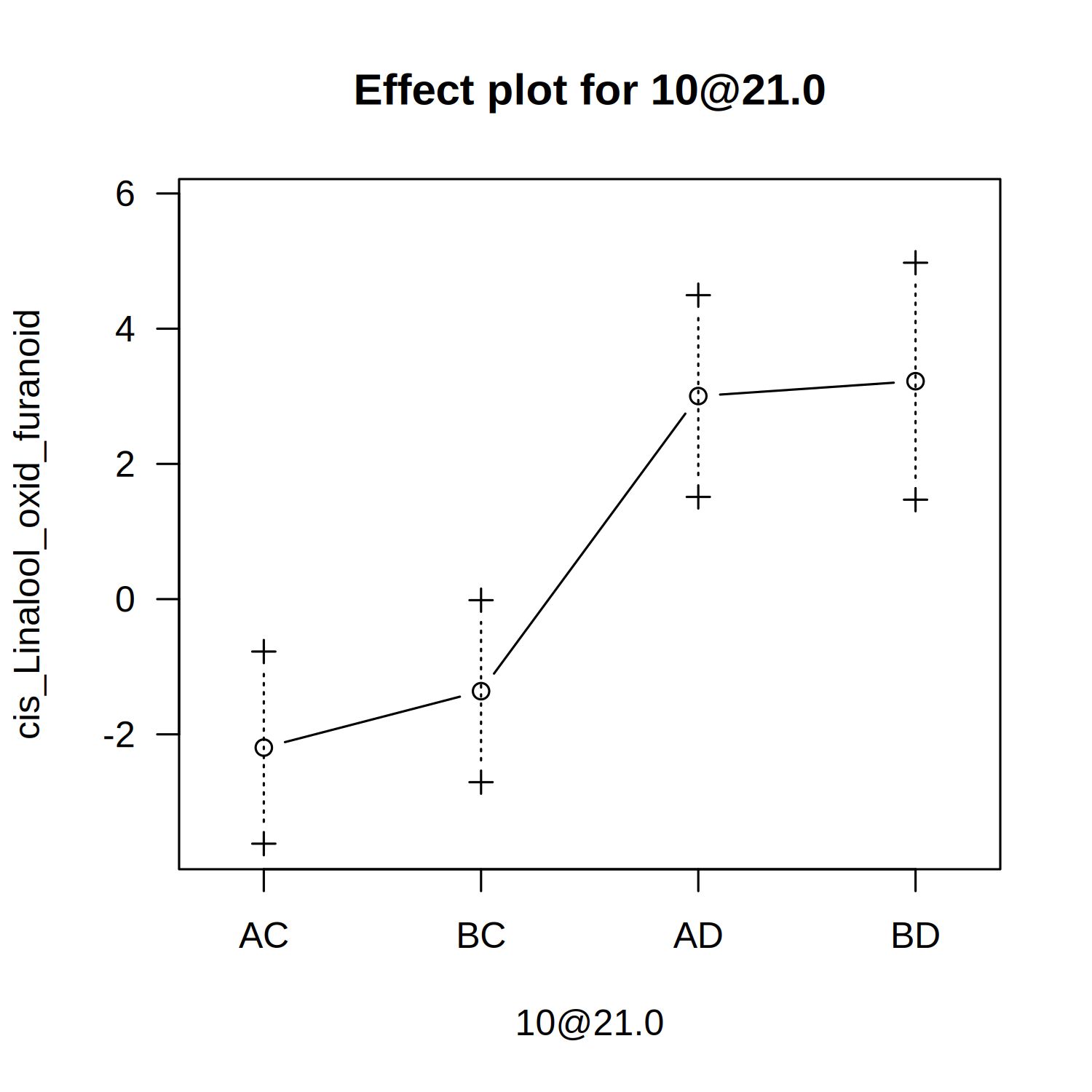

Supplement: Supplementary file 2 [file DataSheet2.zip › Supplementary_Files_4/QTL_analysis/cis_linalool_oxid_furanoid/cis_Linalool_oxid_furanoid_eff_chr10.jpg]
