# Supplementary figures and images for: A multivariate Bayesian modeling strategy coupled with QTL analysis reveals genetic loci linking important sensory wine quality attributes with their corresponding wine aroma compounds (part 3 of 3)
Source: Front Plant Sci. 2026 Jun 26;17:1851889. doi: 10.3389/fpls.2026.1851889 (PMC13350352; doi:10.3389/fpls.2026.1851889)

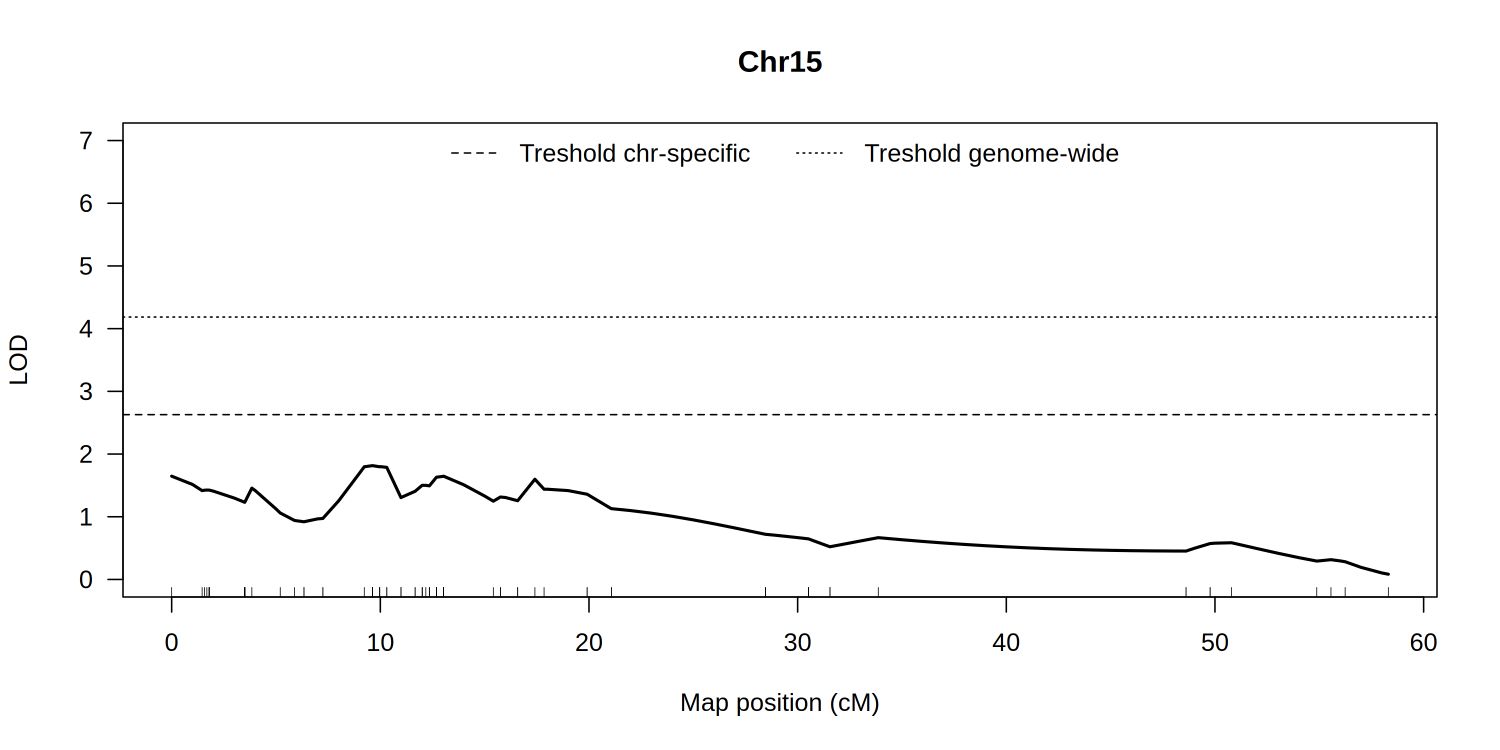

Supplement: Supplementary file 2 [file DataSheet2.zip › Supplementary_Files_4/QTL_analysis/citronellol/citronellol_LODplot_chr15.jpg]
